# The Effect of Dietary Supplements on Male Infertility in Terms of Pregnancy, Live Birth, and Sperm Parameters: A Systematic Review and Meta-Analysis

**DOI:** 10.3390/nu17101710

**Published:** 2025-05-18

**Authors:** Mette Peters Michaelsen, Michelle Poulsen, Anne Ahrendt Bjerregaard, Maria Borgstrøm, Lotte Kraglund Poulsen, Maria Bach Chortsen, Sahra Gatten Henriksen, Ulrik Schiøler Kesmodel

**Affiliations:** 1 Department of Obstetrics and Gynecology, Aalborg University Hospital, 9000 Aalborg, Denmark; mette.michaelsen@rn.dk (M.P.M.); michelle.poulsen@rn.dk (M.P.); 2 Department of Clinical Medicine, Aalborg University, 9000 Aalborg, Denmark; 3 Centre for Clinical Research and Prevention, Copenhagen University Hospital-Bispebjerg and Frederiksberg Hospital, 2000 Frederiksberg, Denmark; anne.ahrendt.bjerregaard@regionh.dk; 4 Department of Epidemiology Research, Statens Serum Institut, 2300 Copenhagen, Denmark; 5 Department of Obstetrics and Gynecology, Herlev and Gentofte University Hospital, 2730 Herlev, Denmark; maria.borgstroem@regionh.dk; 6 Department of Health Science and Technology, Aalborg University, 9220 Aalborg, Denmark; lpou21@student.aau.dk (L.K.P.); mchort21@student.aau.dk (M.B.C.); sghe21@student.aau.dk (S.G.H.)

**Keywords:** male infertility, dietary supplements, live birth, pregnancy, sperm parameters

## Abstract

Background/Objectives: The aim of this systematic review and meta-analysis was to investigate the effect of dietary supplements on male infertility. Methods: PubMed, Embase, and CENTRAL were searched from inception to May 2024. Randomized controlled trials with treatment durations of ≥12 weeks investigating the effect of dietary supplements on male infertility compared to placebo were included. Primary outcomes were pregnancy and live birth, while secondary outcomes were sperm concentration, sperm count, total motility, progressive motility, normal morphology, and DNA Fragmentation Index. Risk of bias was assessed using the revised Cochrane risk of bias (RoB2) tool. Data were meta-analyzed using random effects-restricted maximum likelihood models. Certainty of evidence was evaluated using the grading of recommendations, assessment, development and evaluation (GRADE) approach. Results: Of the 3137 articles identified, 50 were included. No effect on pregnancy and live birth was found. Different supplements improved single sperm parameters: Zinc and folic acid and ≥3 substance dietary supplements improved sperm concentration, selenium, carnitine, and coenzyme Q10 improved motility and alpha-lipoic acid improved normal morphology. Vitamin D, vitamin E, and omega-3 fatty acids showed no improvement in sperm parameters. The majority of studies had some concerns or high risk of bias, and certainty of evidence was generally low or very low. Conclusions: This study found no convincing evidence of an effect of any dietary supplements on male infertility. Larger and more well-conducted randomized controlled trials focusing on specific supplements and considering pregnancy outcomes are needed.

## 1. Introduction

Infertility is a worldwide health problem affecting up to one in six people at some point in their life [1]. Male factor infertility is estimated to be contributory in up to 40–50% of cases [2]. There is an urgent need to identify factors that affect male fertility and to find new treatment strategies to increase the likelihood of successful conception. Modifiable lifestyle factors such as body mass index [3], smoking [4], alcohol consumption [5], and substance use [6] have already been acknowledged as parameters that can affect male fertility. In recent years, an increase in studies investigating the impact of nutrition on male fertility have emerged.

Human sperm contain multiple trace elements essential for male reproductive health, as an imbalance or deficit might lead to reduced sperm quality [7]. In a population-based cohort study including young men, low seminal plasma zinc levels were associated with lower sperm quality [8]. Additionally, in a systematic review and meta-analysis including randomized controlled trials (RCTs), zinc and selenium supplementation was shown to improve some sperm parameters [9].

Spermatozoa are vulnerable to damage induced by oxidative stress (OS) caused by excessive production of reactive oxygen species (ROS). ROS in seminal plasma derives from endogenous factors such as leukocytes and immature spermatozoa, and exogenous factors such as increased age, smoking, alcohol consumption, poor diet, and environmental pollutants [10]. Increased levels of ROS have been associated with aberrations in development, function, fertilization potential, and DNA damage of the spermatozoa [11]. Antioxidants may protect spermatozoa against the negative impact of ROS [12]. Two systematic reviews and meta-analyses on RCTs including infertile men have shown improvements in sperm parameters after L-carnitine (LC) and L-acetyl-carnitine (LAC), and coenzyme Q10 supplementation, respectively. However, neither found improvements in pregnancy rates [13,14].

The effect of dietary supplements on pregnancy-related outcomes has also been investigated. A network meta-analysis assessing the effect of dietary interventions or dietary supplements on male infertility found a statistically significant increase in the chance of pregnancy following LC and micronutrient supplementation, while nearly all other interventions showed non-significant increases in pregnancy chances [15]. Furthermore, a Cochrane review demonstrated that antioxidant supplementation may lead to increased live birth rates and clinical pregnancy rates, although it was highlighted that previous literature is limited by the low number of studies reporting these outcomes [16].

Dietary supplements are readily accessible and affordable [15] and may therefore be favorable in dealing with male infertility if proven effective. Although systematic reviews and meta-analyses have been conducted, important limitations include using only one scientific database, including heterogenous study populations with both fertile and infertile men, or studies affected by female factor infertility, and not considering the duration of spermatogenesis [9,13,14,15,17]. Therefore, the aim of this systematic review and meta-analysis was to evaluate the effect of dietary supplements on male infertility while addressing these limitations.

## 2. Materials and Methods

This study was reported in accordance with the Preferred Reporting Items for Systematic Reviews and Meta-Analyses (PRISMA) 2020 statement [18] and was prospectively registered in PROSPERO (CRD42024544270). Record screenings, data extraction, and evaluations were performed under blinded conditions by two independent reviewers, and disagreements were resolved through discussion or a third party.

### 2.1. Eligibility Criteria, Search Strategy, and Selection Process

Eligibility criteria were formulated using the PICOTS framework (Table 1). With assistance from a medical librarian PubMed, Embase, and CENTRAL were searched in May 2024 with no publication year or geographical restrictions (Table S1A–D). Trial registrations and abstracts from the search were examined and references from included studies were hand-searched. Duplications were removed using EndNote Version 20.1, Clarivate, and screening was conducted in Rayyan [19]. In the case of additional information needed to determine eligibility, the corresponding authors were contacted.

### 2.2. Data Collection

Data were extracted using a standardized extraction form and included author, year, country, study design, blinding, infertility diagnosis, sample size, intervention, comparator, treatment duration, effect estimates, and conclusion. In cross-over studies, only data from the first period were extracted. Data from baseline and all time points from 12 weeks and beyond were extracted. When treatment duration was not clearly defined, the shortest duration was extracted. Data were not extracted if only spontaneous or assisted reproductive technology (ART) pregnancies were reported without rationale. Data on subpopulations and per protocol estimates were not extracted. Corresponding authors were contacted in case of missing data.

### 2.3. Risk of Bias Assessments

Risk of bias was evaluated using the revised Cochrane risk of bias (RoB2) tool for RCTs [20] and presented graphically using robvis [21]. Evaluations were grouped as pregnancy and live birth, and sperm parameters. To ensure the homogeneity of the assessments, two articles were initially evaluated and compared between reviewers. Subsequent evaluations were then performed by two independent reviewers.

### 2.4. Synthesis Methods

Studies were grouped according to the dietary supplement used. Dietary supplements containing ≥3 substances were labelled as multiple substance dietary supplements.

Meta-analyses were performed when ≥2 studies were available. In the case of ≥2 relevant intervention arms in a study, these were pooled to minimize unit of analysis issues. Random effects restricted maximum likelihood models were used. Random effect models were selected based on differences in the characteristics of included studies, e.g., ethnicity and infertility diagnosis. Dichotomous outcomes were compared using risk ratios (RRs) with 95% confidence intervals (95%CI). Due to the limited availability of pregnancy data, the pregnancy parameters biochemical pregnancy, clinical pregnancy, and undefined pregnancy, were simply merged as pregnancy to enable quantitative synthesis. In cases where both biochemical and clinical pregnancy were reported, clinical pregnancy was used. For continuous outcomes, mean differences (MDs) with 95%CI were calculated. *p* < 0.05 was considered statistically significant.

Primary analyses were conducted on follow-up estimates at the longest treatment duration. Secondary analyses on sperm parameters were performed based on generated pseudo individual participant data (IPD) to adjust for baseline imbalances [22] using the two-stage approach when both baseline and follow-up estimates were reported. Sample sizes at follow-up were used. Correlation coefficients were calculated based on articles where baseline, follow-up, and change-from-baseline standard deviations or standard errors (SEs) were obtainable, and a weighted average was used for each outcome (Table S2). Secondary analyses were not performed if correlation coefficients were <0.5 [23]. The pseudo-IPD was used to estimate MDs with SEs using analysis of covariance (ANCOVA), and meta-analyses were performed thereafter. Subgroup analyses were performed on the duration of treatment (3 months, >3 months, ≥6 months) and substance content in terms of single substance or the single substance and an additional substance. Furthermore, explorative subgroup analyses divided into infertility diagnosis regardless of exposure and sensitivity analyses excluding studies with a high risk of bias were conducted.

Heterogeneity was evaluated using the I^2^ and Chi^2^ test. The interpretation of I^2^ was based on thresholds described by Cochrane [24]. For the Chi^2^ test, a significance level of 0.05 was chosen. Funnel plots were conducted when ≥10 studies were available [25].

Analyses were performed in Stata 18 (StataCorp LLC, College Station, TX, USA).

### 2.5. Certainty Assessments

Certainty of evidence of primary meta-analyses was assessed using the grading of recommendations assessment, development and evaluation (GRADE) approach and graded as either high, moderate, low, or very low [26]. The GradePRO 2024 software was used (McMaster University and Evidence Prime).

## 3. Results

### 3.1. Study Selection

**Figure 1:**
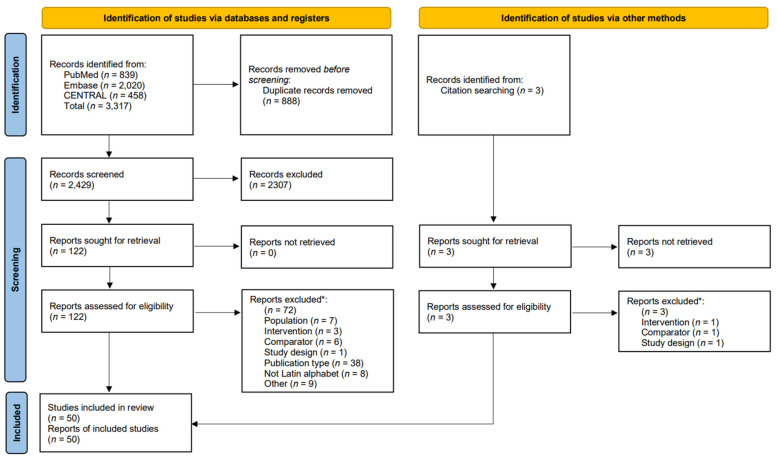
PRISMA flow diagram. Schematic presentation of the selection process. * Some papers might have more than one reason for exclusion; however, the main reason was used for categorization.

Of the 3317 records identified, 50 and 41 articles were eligible for qualitative and quantitative synthesis, respectively (Figure 1). Reasons for exclusion are seen in Table S3. Of the 16 authors contacted, three responded. For studies with non-responsiveness, conservative choices were made (Table S4).

### 3.2. Study Characteristics

Participants (*n* > 4800) were primarily from Europe and Asia and most frequently suffered from asthenzoospermia, oligozoospermia, teratozoospermia, or a combination of these. The supplements assessed were vitamin D [27,28,29,30], LC and LAC [31,32,33,34,35], zinc and folic acid [36,37,38,39,40,41,42,43], coenzyme Q10 [44,45,46,47], alpha-lipoic acid [48,49], omega-3 fatty acids [50,51,52], vitamin E [50,53,54], selenium [54,55], multiple substance dietary supplements [55,56,57,58,59,60,61,62,63,64], and other dietary supplements [46,65,66,67,68,69,70,71,72,73,74,75,76]. Treatment durations varied between 12 and 32 weeks. Information on pregnancy and live birth was obtained from 4 and 11 studies, respectively. All studies reported at least one sperm parameter. Forty studies had a parallel design, eight were factorial, and two were cross-over. Most studies were double-blinded, while six were triple-blinded, three were single-blinded, and in three studies, the blinding was unclear (Table S5).

### 3.3. Risk of Bias in Studies

**Figure 2:**
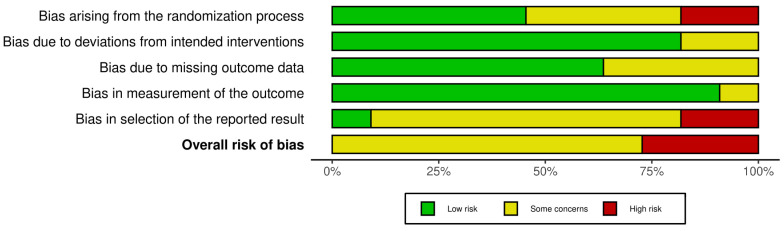
Aggregated risk of bias assessments on the primary outcomes pregnancy and live birth. Overall risk of bias indicates the summary assessment across all bias domains per study. Risk of bias assessments have been visualized using the Robvis tool (2024).

**Figure 3:**
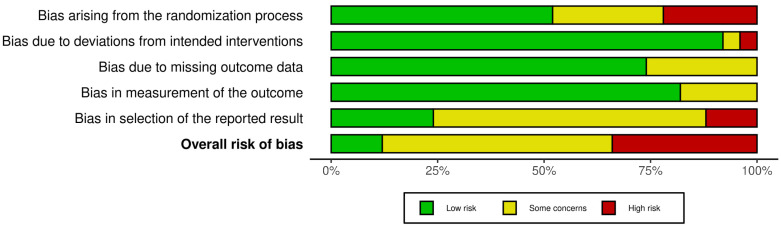
Aggregated risk of bias assessments on the secondary outcomes sperm concentration, sperm count, total motility, progressive motility, normal morphology, and DNA Fragmentation Index. Overall risk of bias indicates the summary assessment across all bias domains per study. Risk of bias assessments have been visualized using the Robvis tool (2024).

Studies assessing pregnancy and live birth all had some concerns or high overall risk of bias. For studies reporting sperm parameters, the overall risk of bias was low in six studies, some concerns in 17, and high in 17. This was mostly due to problems with the randomization process or selection of the reported results (Figure 2, Figure 3, Figures S1 and S2).

### 3.4. Qualitative Synthesis

#### 3.4.1. Vitamin D

Four studies assessed the efficacy of vitamin D for 12–22 weeks administered as either an initial dose of 300,000 IU and hereafter 1400 IU/day, weekly and monthly dosages of 50,000 IU or 400 IU/day [27,28,29,30]. One study reported no improvements in pregnancy and live birth rates [27]. Three studies reported no improvement in sperm parameters [27,28,30], while one found improvements in some [29].

#### 3.4.2. L-carnitine and L-acetyl-carnitine

Five studies evaluated the efficacy of 1–3 g/day L-carnitine and L-acetyl-carnitine for 3–6 months [31,32,33,34,35]. One study evaluated pregnancy rates and found no improvement [34]. Four studies reported improvements in most or all sperm parameters [31,32,33,35], while one reported no improvements [34].

#### 3.4.3. Zinc and Folic Acid

Eight studies investigated efficacy for 3–6 months. The dose of zinc varied between 15 and 220 mg/day, and most studies used 5 mg/day folic acid [36,37,38,39,40,41,42,43]. One study found an increase in live births for men with MTHFR 677 TT genotype, but not for other genotypes [41]. While two studies reported improvements in all measured sperm parameters [39,41], the majority found either no improvements or improvement in only one [36,37,38,40,42,43]

#### 3.4.4. Coenzyme Q10

Four studies assessed the efficacy of 100–200 mg/day coenzyme Q10 for 3–6 months [44,45,46,47]. One study reported an improvement in all measured sperm parameters [46], two reported improvements in some [44,45], and one reported no improvements [46,47].

#### 3.4.5. Alpha-Lipoic Acid

Two studies assessed the efficacy of 600 mg/day alpha-lipoic acid for three months and reported improvements in all measured sperm parameters, except morphology [48,49].

#### 3.4.6. Omega-3 Fatty Acids

Three studies evaluated efficacy for 12–32 weeks of omega-3 fatty acids with dosages of docosahexaenoic acid (DHA) between 400 and 800 mg/day, while 1.12 g/day of eicosatetraenoic acid was given by one study [50,51,52]. Two studies reported improvements in all or most measured sperm parameters [50,52], while one reported no improvements [51].

#### 3.4.7. Vitamin E

Three studies assessed the efficacy of 400–600 IU/day of vitamin E for three months [50,53,54]. One study reported no improvements in pregnancy and live birth rates [53]. One study reported improvements in all sperm parameters [50], while two reported improvement in only one [54] or none [53].

#### 3.4.8. Selenium

Two studies evaluated the effect of 100–200 µg/day of selenium for three months [54,55]. One study reported an aggregated pregnancy rate for two intervention arms and found a higher number of pregnancies [55]. Total motility improved in one study [54], while the other showed no improvements in sperm parameters [55].

#### 3.4.9. Multiple Substance Dietary Supplements

Ten studies evaluated the effect of multiple substance dietary supplements for 3–6 months [55,56,57,58,59,60,61,62,63,64]. Of the five studies reporting pregnancy rate [55,56,57,58,59], three reported improvements [55,56,57] (see Table S6 for the exact substance content). Live birth rate was reported by one study, who found no difference [59]. All sperm parameters improved in four studies [56,57,62,63], some improved in two [61,64], and four studies showed no improvement [55,58,59,60].

#### 3.4.10. Other Dietary Supplements

Thirteen studies evaluated the effects of other supplements than those mentioned above. Arginine did not have an effect on pregnancy rates [69], while more pregnancies were seen following M. sativa supplementation [76]. Myo-inositol [66], glutathione [46], melatonin [70], probiotics [71], Korean red ginseng [75], and M. sativa [76] improved all measured sperm parameters. Lycopene [73], N-acetyl-cysteine [67], and vitamin C [65] improved at least one, and magnesium-orotate [68], arginine [69], astaxanthin [72] and saffron [74] did not improve any sperm parameters.

### 3.5. Quantitative Synthesis

#### 3.5.1. Vitamin D

Primary analyses showed no improvements of vitamin D supplementation on concentration, count, total motility, progressive motility and morphology (Table 2, Figures S3–S7). Secondary analyses were consistent with the primary analyses (Figures S8–S12). Subgroup analyses on treatment duration and substance content were identical and showed no improvements (Figures S13–S20). Sensitivity analyses could not be performed.

#### 3.5.2. L-carnitine and L-acetyl-carnitine

Primary analyses revealed improvement after supplementation with LC and LAC in total motility and no improvements in concentration, progressive motility, and morphology (Table 2, Figures S21–S24). Secondary analysis on total motility changed to a nonsignificant difference, while remaining results were similar to the primary analyses (Figures S25–S28). Subgroup analyses on treatment duration demonstrated no improvement in concentration after three months, while an improvement was seen after ≥6 months. For total motility, an improvement was seen after three months, but not after ≥6 months. For progressive motility and morphology, results were consistent with primary analyses (Figures S29–S35). In the subgroup analyses on substance content, LC and LAC improved concentration, while no improvement was seen for LC given alone. Total motility improved after LC supplementation, while no improvement was seen for LC and LAC given together. Progressive motility showed no improvement after LC and LAC supplementation (Figures S36–S40). Sensitivity analyses were identical to analyses on a treatment duration of ≥6 months (Figures S41–S43).

#### 3.5.3. Zinc and Folic Acid

Primary analyses revealed improvement in concentration and no improvements in total motility, progressive motility, and morphology after zinc and folic acid supplementation (Table 2, Figures S44–S47). Secondary analysis on concentration revealed a nonsignificant difference, while remaining results were similar to the primary analyses (Figures S48–S51). Subgroup analyses on treatment duration showed an improvement in concentration after supplementation for 3 and ≥6 months, but not for >3 months. For total motility, progressive motility and morphology results were similar to the primary analyses (Figures S52–S60). Subgroup analyses on substance content revealed an improvement in concentration and progressive motility for zinc given alone. Remaining analyses showed no improvements for either zinc or folic acid given together or separately (Figures S61–S71). Sensitivity analyses showed an improvement in concentration and no improvements in total motility and morphology (Figures S72–S74).

#### 3.5.4. Coenzyme Q10

Primary analyses revealed improvements in total and progressive motility, and no improvement in concentration after supplementation with coenzyme Q10 (Table 2, Figures S75–S77). Results from secondary analyses were comparable to primary analyses (Figures S78–S80). Subgroup and sensitivity analyses could not be performed.

#### 3.5.5. Alpha-Lipoic Acid

Primary analysis revealed an improvement in morphology, and no improvements in concentration or total and progressive motility following alpha-lipoic acid supplementation (Table 2, Figures S81–S84). Results from secondary analyses were comparable to primary analyses (Figures S85–S88). Subgroup and sensitivity analyses could not be performed.

#### 3.5.6. Omega-3 Fatty Acids

Primary analyses showed no improvements in concentration, count, total motility, or morphology following omega-3 fatty acids supplementation (Table 2, Figures S89–S92). Results from secondary analyses were comparable to primary analyses (Figures S93–S96). Subgroup analyses on three months of treatment and on substance content for DHA given alone revealed no improvements in concentration and total motility (Figures S97–S100). Sensitivity analyses showed no improvements in concentration and total motility (Figures S101 and S102). Sensitivity analyses on count and morphology were identical to primary analyses and therefore not conducted.

#### 3.5.7. Vitamin E

Primary analyses showed no improvements of vitamin E supplementation in concentration, total motility, or morphology (Table 2, Figures S103–S105). Secondary analysis on concentration and morphology revealed improvements, while total motility showed results similar to the primary analysis (Figures S106–S108). Subgroup and sensitivity analyses could not be performed.

#### 3.5.8. Selenium

Primary analyses revealed an improvement in total motility, and no improvement in concentration after selenium supplementation (Table 2, Figures S109 and S110). Secondary analyses showed similar results (Figures S111 and S112). Subgroup and sensitivity analyses could not be performed.

#### 3.5.9. Multiple Substance Dietary Supplements

Primary analyses of multiple substance dietary supplements revealed no increased chance of achieving pregnancy (Table 2, Figure S113). Primary analyses demonstrated an improvement in concentration (Figure 4), and no improvements in count, total motility, progressive motility, morphology, and DNA Fragmentation Index (Table 2, Figures S113–S118). Secondary analyses on progressive motility showed an improvement, while remaining results were similar to the primary analyses (Figures S119–S123). Subgroup analyses on treatment duration showed improvement in concentration after supplementation for >3 and ≥6 months, but not following three months of treatment. Subgroup analyses on count showed a statistically significant difference favoring placebo after three months; however, no difference was observed after ≥6 months. Pregnancy, total motility, progressive motility, morphology, and DNA Fragmentation Index did not differ from primary analyses (Figures S124–S138). Sensitivity analyses on pregnancy and count were identical to primary analyses and therefore not conducted. Sensitivity analyses on concentration, total motility, progressive motility, morphology, and DNA Fragmentation Index showed no improvements (Figures S139–S142).

#### 3.5.10. Explorative Subgroup Analysis

Analyses were divided according to the diagnosis of infertility including any dietary supplement. Analyses of asthenozoospermia revealed an improvement in total and progressive motility and no improvements in concentration, count, or morphology (Figures S143–S147). Analyses of oligozoospermia revealed an improvement in concentration and no improvements in total motility or morphology (Figures S148–S150). Analyses on oligoasthenozoospermia revealed an improvement in total motility, and no improvement in concentration (Figures S151–S152). For oligoasthenoteratozoospermia, concentration, and morphology improved. No improvements were seen in count or total and progressive motility (Figures S153–S157). For oligo- and/or astheno- and/or teratozoospermia, all analyses showed no improvements (Figures S158–S165). For infertile men undergoing varicocelectomy, an improvement was seen in concentration. No improvements were observed in total motility and morphology (Figures S166–S168). Analyses on varicocele revealed an improvement in concentration and morphology (Figures S169–S170).

### 3.6. Reporting Bias

**Figure 5:**
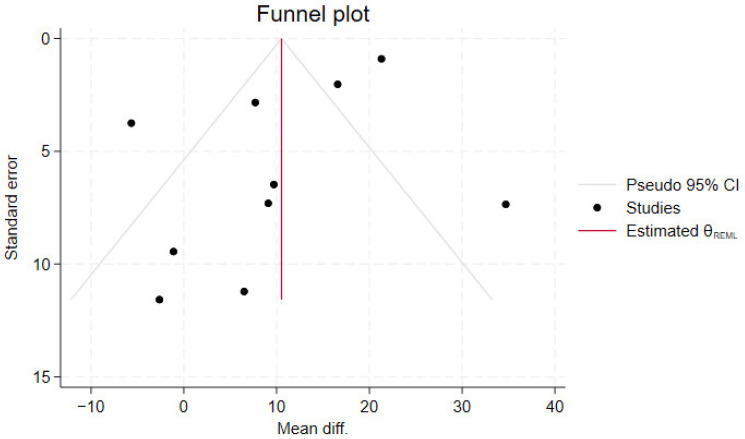
Funnel plot on the primary analysis on the effect of multiple substance dietary supplements on sperm concentration.

Publication bias analysis was performed for the effect of multiple substance dietary supplements on concentration. The funnel plot suggested some risk of publication bias with a skewed distribution of studies (Figure 5).

### 3.7. Certainty of Evidence

Certainty of evidence was predominantly low or very low. Reasons for downgrading were in most cases due to a risk of bias in the randomization process of individual studies, a small number of studies and sample sizes, and inconsistency caused by heterogeneity and differing results between studies (Figures S171–S179).

## 4. Discussion

This systematic review and meta-analysis found no evidence of an effect of dietary supplements on pregnancy and live birth rates in the limited data published. While some supplements showed improvements in sperm parameters, the improvements were limited to a single parameter. Zinc and folic acid and combined substance dietary supplements improved sperm concentration, selenium, carnitine, and coenzyme Q10 improved motility, and alpha-lipoic acid improved normal morphology. Most results were consistent between primary, secondary, subgroup, and sensitivity analyses. Furthermore, the explorative subgroup analyses indicated that sperm parameter improvements might depend on the diagnosis of infertility.

Other meta-analyses have concluded improving effects on sperm parameters following supplements such as LC and LAC, coenzyme Q10, omega-3 fatty acids, selenium, and multiple-substance dietary supplements [9,15,17]. The differing results of some supplements compared to this current study might be attributed to different methodological considerations. In contrast to some of the previous reviews [9,15], this current study only included placebo-controlled trials as participants’ expectations and behaviors might be influenced if they are aware that they are not receiving the intervention aimed to potentially improve their fertility status. Furthermore, while the inclusion of both infertile and fertile males could increase statistical power [9], it would also lead to a heterogeneous study population. Additionally, previous studies have not considered the duration of spermatogenesis in their inclusion criteria [9,15,17], thereby including studies with treatment durations of less than 12 weeks.

Although pregnancy and live birth were the primary outcomes of interest in the current study, the limited number of studies reporting these outcomes hindered a quantitative synthesis. Current research on whether sperm quality can predict the chances of pregnancy and live birth is conflicting [77,78,79,80]. It is therefore unknown whether an improvement in sperm parameters might positively impact these outcomes.

While some of the included studies adhered to the tolerable upper intake level (UL), as established by the European Food Safety Authority (EFSA) and the Institute of Medicine (IOM) [81,82], or were within the range of previous studies with no serious adverse events where no UL exists [32,83,84], others exceeded the UL. For vitamin D, higher initial doses or high weekly doses were applied. Although they exceeded the UL, the administered doses have been found to be safe and effective in treating vitamin D insufficiency [81,85,86]. The administration of zinc and folic acid exceeded UL in most studies [81,87,88], as did vitamin E according to the IOM but not the EFSA [81,82]. The lack of adherence to established ULs could potentially impact the participants negatively and should be considered in future studies.

The current study focused on the isolated effects of dietary supplements. However, it should be emphasized that their efficacy may be influenced by dietary patterns. Adherence to the Mediterranean diet, characterized by a high intake of fruit, vegetables, legumes, whole grains, olive oil, nuts, and a moderate intake of animal products, has demonstrated a positive impact on male fertility, likely due to its antioxidant and anti-inflammatory properties [89,90]. Conversely, the Western diet, characterized by high intakes of processed foods, added sugars, and saturated and trans fatty acids has been associated with reduced sperm quality [90]. This may suggest that individuals with adequate micronutrient intake from a balanced diet will not experience any additional benefit from the possible positive effects of dietary supplements on male infertility, whereas individuals with a poorer dietary quality may benefit due to unmet nutritional needs.

Another factor which may influence the effect of dietary supplements is the gut microbiota. It is well known that dietary patterns may influence gut microbiota composition. Diets rich in fiber, polyphenols, and unsaturated fatty acids have been found to enhance microbial diversity, while diets high in saturated fatty acids and refined carbohydrates have been associated with dysbiosis, defined as an imbalance in the microbial composition [91]. Emerging evidence suggests that the gut microbiota has an impact on male reproductive health [90,92,93]. An imbalance in the gut microbiota can lead to increased OS, which can negatively impact spermatozoa and cause hormonal dysfunction and thereby affect spermatogenesis [93]. The gut microbiota is also involved in modulating the bioavailability of micronutrients, which can affect the effectiveness of dietary supplements [94,95]. Additionally, the relationship between micronutrients and the gut microbiota is bidirectional. While the gut microbiota modulates the absorption of micronutrients, micronutrients can also influence the microbial composition of the gut microbiota [94].

Moreover, genetic variations affecting micronutrient metabolism may also impact male infertility. The 677 TT genotype is a common genetic variation of the MTHFR gene. While this variation is associated with lower levels of circulating folate and a higher risk of male infertility, supplementation has been associated with improved sperm parameters [96]. Genetic variations in other genes are also known to influence micronutrient metabolism, such as variations in the FUT2 gene affecting vitamin B12 levels and variations in the CYP2R1 and GC genes affecting vitamin D levels [96].

Throughout the world, different dietary supplements are marketed as fertility improving, even though there is limited clinical evidence to support their effect on male infertility [97,98]. Individuals with fertility issues are often willing to explore multiple treatment strategies and often seek information on the internet about lifestyle and nutrition including dietary supplements [99]. Furthermore, their commitment to exploring the possibilities of improving their fertility might lead to an excessive use of antioxidants from dietary supplements, which have been suggested to lead to reductive stress and might have the same negative effect on male fertility as OS [100]. Before dietary supplements can be recommended for male infertility, there is a need for more, larger, and well-conducted RCTs, which include pregnancy and live birth as outcome measures and investigate the effect of the same supplements to create high-quality knowledge.

### Strengths and Limitations

The strengths of this study include the use of multiple databases, the hand-searching of references, and the examination of trial registrations and abstracts which contributed to identifying relevant literature. All methodological elements were conducted by two independent reviewers to minimize the subjective judgements. Inclusion criteria were designed to enhance the methodological accuracy, for example, excluding studies with female factor infertility involvement and studies with intervention periods of <12 weeks. Furthermore, multiple analyses were performed to cover multiple aspects of the research question.

Limitations of this study should also be considered. Most studies had some concerns or high risk of bias, and the certainty of evidence was mostly low or very low. Differences in terms of dosages, treatment durations, and infertility diagnoses could have influenced the results. The methodological approach of this review also contains limitations. Pregnancy and live birth data were not extracted from studies where no explanation for only reporting one method of conceiving was available. This may have limited the use of available data; however, the criterion was made to ensure the homogeneity and consistency of the results. Secondary analyses were conducted to adjust for potential baseline imbalances. Although this aspect is valuable, the method is limited, as the ANCOVA assumes a normal distribution which may not be the case for all studies, as some reported data as non-parametric. Furthermore, correlation coefficients used to generate the pseudo-IPD were based on weighted averages which may not be applicable to all studies, although a weighted average would be preferred over only using a single estimate.

## 5. Conclusions

Although this systematic review and meta-analysis suggested the possible positive effects of dietary supplements on some sperm parameters, there is no substantial evidence to support that any specific dietary supplements improve male infertility in terms of pregnancy, live birth, or sperm parameters. This is primarily due to the limited availability of studies assessing the same dietary supplements and outcome measures, the risk of bias in the included studies, and an overall low certainty of evidence. Future studies should aim to conduct larger and methodological robust studies to create evidence-based knowledge on the effect on dietary supplements on male infertility.

## Figures and Tables

**Figure 4:**
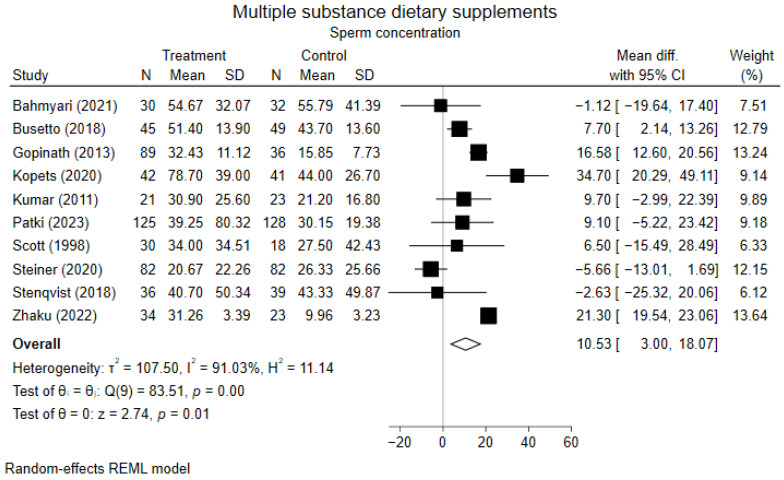
Forest plot of primary analysis on the effect of multiple substance dietary supplements on sperm concentration [55,56,57,58,59,60,61,62,63,64].

**Table 1:** Inclusion and exclusion criteria based on the population–intervention–comparison–outcome–time–study design framework.

	Inclusion Criteria	Exclusion Criteria
Population	Males with confirmed infertility or male partners in couples with unexplained infertility *	Female factor infertility; male infertility caused by obstruction of the genital tract; transgender persons
Intervention	Dietary supplements	Diets; foods; medications
Comparison	Placebo	-
Outcome	Primary outcomes: biochemical pregnancy, clinical pregnancy, undefined pregnancy and live birth rate Secondary outcomes: sperm concentration, sperm count, total and progressive motility, normal morphology and sperm DNA Fragmentation Index	-
Time	Intervention planned for use for a minimum of 12 weeks to account for the length of the spermatogenesis	Intervention planned for use for less than 12 weeks
Study design	Randomized controlled trials	-
Other	-	Duplications; unavailable full texts; abstract-only papers; articles where the journal had issued an expression of concern at the time of the search; article not written in the Latin alphabet; studies conducted on animals or in vitro

* Infertility was defined as being unable to conceive after at least 12 months of regular, unprotected intercourse.

**Table 2:** Summary of the effect measures and 95% confidence intervals for primary analyses on the effect of dietary supplements on male infertility.

	Number of Studies	Effect Measure (95% CI)	I^2^	Chi^2^
Vitamin D				
Pregnancy	1	-	-	-
Live birth	1	-	-	-
Sperm concentration	3	MD −2.09 10^6^/mL (−6.38; 2.19)	0.00%	*p* = 0.77
Sperm count	3	MD 0.06 10^6^/ejaculate (−3.81; 3.94)	0.00%	*p* = 0.68
Total motility	2	MD 1.20% (−8.45; 10.84)	88.26%	*p* = 0.00
Progressive motility	4	MD 0.55% (−4.00; 5.10)	66.42%	*p* = 0.01
Normal morphology	4	MD −0.07% (−0.92; 0.77)	56.84%	*p* = 0.09
DNA Fragmentation Index	1	-	-	-
L-carnitine and L-acetyl-carnitine				
Pregnancy	0	-	-	-
Live birth	0	-	-	-
Sperm concentration	4	MD 2.84 10^6^/mL (−1.79; 7.47)	96.28%	*p* = 0.00
Sperm count	1	-	-	-
Total motility	3	MD 9.80% (0.21; 19.39)	95.60%	*p* = 0.00
Progressive motility	2	MD 8.47% (−6.47; 23.42)	95.21%	*p* = 0.00
Normal morphology	2	MD 5.28% (−0.88; 11.44)	99.90%	*p* = 0.00
DNA Fragmentation Index	0	-	-	-
Zinc and folic acid				
Pregnancy	0	-	-	-
Live birth	0	-	-	-
Sperm concentration	6	MD 7.81 10^6^/mL (1.49; 14.13)	81.95%	*p* = 0.00
Sperm count	1	-	-	-
Total motility	5	MD 1.00% (−1.25; 3.25)	0.00%	*p* = 0.96
Progressive motility	3	MD 4.38% (−3.61; 12.37)	87.41%	*p* = 0.00
Normal morphology	6	MD 0.69% (−0.54; 1.92)	92.22%	*p* = 0.00
DNA Fragmentation Index	1	-	-	-
Coenzyme Q10				
Pregnancy	0	-	-	-
Live birth	0	-	-	-
Sperm concentration	2	MD −0.56 10^6^/mL (−6.53; 5.41)	0.00%	*p* = 0.84
Sperm count	1	-	-	-
Total motility	2	MD 4.35% (0.71; 8.00)	0.00%	*p* = 0.87
Progressive motility	2	MD 4.95% (2.11; 7.79)	0.00%	*p* = 0.92
Normal morphology	1	-	-	-
DNA Fragmentation Index	0	-	-	-
Alpha-lipoic acid				
Pregnancy	0	-	-	-
Live birth	0	-	-	-
Sperm concentration	2	MD 15.46 10^6^/mL (−8.30; 39.21)	98.70%	*p* = 0.00
Sperm count	1	-	-	-
Total motility	2	MD 17.52% (−7.90; 42.94)	98.80%	*p* = 0.00
Progressive motility	2	MD 15.29% (−2.37; 32.94)	98.89%	*p* = 0.00
Normal morphology	2	MD 0.95% (0.27; 1.63)	0.00%	*p* = 0.54
DNA Fragmentation Index	0	-	-	-
Omega-3 fatty acids				
Pregnancy	0	-	-	-
Live birth	0	-	-	-
Sperm concentration	3	MD 5.73 10^6^/mL (−4.51; 15.98)	99.09%	*p* = 0.00
Sperm count	2	MD 10.73 10^6^/ejaculate (−8.39; 29.86)	99.21%	*p* = 0.00
Total motility	3	MD 1.57% (−8.04; 11.18)	98.88%	*p* = 0.00
Progressive motility	1	-	-	-
Normal morphology	2	MD 2.64% (−2.64; 7.91)	98.17%	*p* = 0.00
DNA Fragmentation Index	0	-	-	-
Vitamin E				
Pregnancy	1	-	-	-
Live birth	1	-	-	-
Sperm concentration	2	MD 0.53 10^6^/mL (−0.36; 1.42)	0.00%	*p* = 0.66
Sperm count	1	-	-	-
Total motility	2	MD 8.31% (−5.14; 21.75)	97.87%	*p* = 0.00
Progressive motility	1	-	-	-
Normal morphology	2	MD 0.40% (−0.09; 0.89)	6.76%	*p* = 0.30
DNA Fragmentation Index	0	-	-	-
Selenium				
Pregnancy	1	-	-	-
Live birth	0	-	-	-
Sperm concentration	2	MD 6.32 10^6^/mL (−14.88; 27.51)	60.70%	*p* = 0.11
Sperm count	0	-	-	-
Total motility	2	MD 15.25% (11.71; 18.80)	0.00%	*p* = 0.96
Progressive motility	0	-	-	-
Normal morphology	1	-	-	-
DNA Fragmentation Index	0	-	-	-
Multiple substance dietary supplements				
Pregnancy	4	RR 1.75 (0.58; 5.31)	72.65%	*p* = 0.01
Live birth	1	-	-	-
Sperm concentration	10	MD 10.53 10^6^/mL (3.00; 18.07)	91.03%	*p* = 0.00
Sperm count	3	MD 3.10 10^6^/ejaculate (−38.05; 44.24)	81.22%	*p* = 0.00
Total motility	8	MD 2.73% (−1.75; 7.21)	76.88%	*p* = 0.00
Progressive motility	6	MD 5.55% (−1.31; 12.41)	89.28%	*p* = 0.00
Normal morphology	6	MD −0.04% (−0.54; 0.46)	0.00%	*p* = 0.11
DNA Fragmentation Index	2	MD 1.27% (−2.79; 5.33)	0.00%	*p* = 0.47

-: not performed due to data availability. 95%CI = 95% confidence interval, MD = mean difference, RR = risk ratio.

## Data Availability

No new data were created or analyzed in this study. Data sharing is not applicable to this article.

## Supplementary Materials

The following supporting information can be downloaded at https://www.mdpi.com/article/10.3390/nu17101710/s1, Figure S1: Individual risk of bias assessments on primary outcomes; Figure S2: Individual risk of bias assessments on secondary outcomes; Figures S3–S170: Forest plots; Figures S171–S179: GRADE assessments; Table S1A: Results from the systematic literature searches; Table S1B: Search strategy in PubMed; Table S1C: Search strategy in Embase; Table S1D: Search strategy in Cochrane Central Register of Controlled Trials; Table S2: Calculated correlation coefficients for secondary analyses; Table S3: List of excluded studies and reason for exclusion; Table S4: Author contacts and conservative decisions regarding data extraction for included studies; Table S5: Characteristics of included studies; Table S6: Substance content of the dietary supplements administered by the included studies grouped as multiple substance dietary supplements.

## Abbreviations

The following abbreviations are used in this manuscript:

ANCOVA	Analysis of covariance
ART	Assisted reproductive technology
DHA	Docosahexaenoic acid
EFSA	European Food Safety Authority
GRADE	Grading of recommendations, assessment, development, and evaluation
IOM	Institute of Medicine
LAC	L-acetyl-carnitine
LC	L-carnitine
MD	Mean difference
OS	Oxidative stress
PICOTS	Population–intervention–comparison–outcome–ime–study design
PRISMA	Preferred Reporting Items for Systematic Reviews and Meta-analyses
Pseudo-IDP	Pseudo individual participant data
RCT	Randomized controlled trial
RoB2	Revised Cochrane risk of bias
ROS	Reactive oxygen species
RR	Risk ratio
SE	Standard errors
UL	Tolerable upper intake level
95%CI	95% confidence interval
