# Supplementary material for: The Effect of Dietary Supplements on Male Infertility in Terms of Pregnancy, Live Birth, and Sperm Parameters: A Systematic Review and Meta-Analysis
Source: Nutrients. 2025 May 18;17(10):1710. doi: 10.3390/nu17101710 (PMC12113742; doi:10.3390/nu17101710)
Supplement: Supplementary file 1 [file nutrients-17-01710-s001.zip › nutrients-3643910-supplementary.pdf]

Tables and Figures

The effect of dietary supplements on male infertility in term of live birth, pregnancy and sperm parameters: a systematic review and meta-analysis

Table of contents

Table S1A. Results the from systematic literature searches. 7

Table S1B. Search strategy in PubMed. 7

Table S1C. Search strategy in Embase. 12

Table S1D. Search strategy in Cochrane Central Register of Controlled Trials. 14

Table S2. Calculated correlation coefficients for secondary analyses. 16

Table S3. List of excluded studies and reason for exclusion. 17

Table S4. Author contacts and conservative decisions regarding data extraction for included studies. 26

Table S5. Characteristics of included studies 28

Table S6. Substance content of the dietary supplements administered by the included studies grouped as multiple substance dietary supplements. 44

Figure S1 45

Figure S2 46

Figure S3 47

Figure S4 47

Figure S5 48

Figure S6 48

Figure S7 49

Figure S8 49

Figure S9 50

Figure S10 50

Figure S11 51

Figure S12 51

Figure S13 52

Figure S14 52

Figure S15 53

Figure S16 53

Figure S17 54

Figure S18 54

Figure S19 55

Figure S20 55

Figure S21........ 56
Figure S22........ 56
Figure S23........ 57
Figure S24........ 57
Figure S25........ 58
Figure S26........ 58
Figure S27........ 59
Figure S28........ 59
Figure S29........ 60
Figure S30........ 60
Figure S31........ 61
Figure S32........ 61
Figure S33........ 62
Figure S34........ 62
Figure S35........ 63
Figure S36........ 63
Figure S37........ 64
Figure S38........ 64
Figure S39........ 65
Figure S40........ 65
Figure S41........ 66
Figure S42........ 66
Figure S43........ 67
Figure S44........ 67
Figure S45........ 68
Figure S46........ 68
Figure S47........ 69
Figure S48........ 69
Figure S49........ 70
Figure S50........ 70
Figure S51........ 71
Figure S52........ 71
Figure S53........ 72
Figure S54........ 72

Figure S55.. 73
Figure S56.. 73
Figure S57.. 74
Figure S58.. 74
Figure S59.. 75
Figure S60.. 75
Figure S61.. 76
Figure S62.. 76
Figure S63.. 77
Figure S64.. 77
Figure S65.. 78
Figure S66.. 78
Figure S67.. 79
Figure S68.. 79
Figure S69.. 80
Figure S70.. 80
Figure S71.. 81
Figure S72.. 81
Figure S73.. 82
Figure S74.. 82
Figure S75.. 83
Figure S76.. 83
Figure S77.. 84
Figure S78.. 84
Figure S79.. 85
Figure S80.. 85
Figure S81.. 86
Figure S82.. 86
Figure S83.. 87
Figure S84.. 87
Figure S85.. 88
Figure S86.. 88
Figure S87.. 89
Figure S88.. 89

Figure S89 90
Figure S90 90
Figure S91 91
Figure S92 91
Figure S93 92
Figure S94 92
Figure S95 93
Figure S96 93
Figure S97 94
Figure S98 94
Figure S99 95
Figure S100 95
Figure S101 96
Figure S102 96
Figure S103 97
Figure S104 97
Figure S105 98
Figure S106 98
Figure S107 99
Figure S108 99
Figure S109 100
Figure S110 100
Figure S111 101
Figure S112 101
Figure S113 102
Figure S114 102
Figure S115 103
Figure S116 103
Figure S117 104
Figure S118 104
Figure S119 105
Figure S120 105
Figure S121 106
Figure S122 106

Figure S123........ 107
Figure S124........ 107
Figure S125........ 108
Figure S126........ 108
Figure S127........ 109
Figure S128........ 109
Figure S129........ 110
Figure S130........ 110
Figure S131........ 111
Figure S132........ 111
Figure S133........ 112
Figure S134........ 112
Figure S135........ 113
Figure S136........ 113
Figure S137........ 114
Figure S138........ 114
Figure S139........ 115
Figure S140........ 115
Figure S141........ 116
Figure S142........ 116
Figure S143........ 117
Figure S144........ 117
Figure S145........ 118
Figure S146........ 118
Figure S147........ 119
Figure S148........ 119
Figure S149........ 120
Figure S150........ 120
Figure S151........ 121
Figure S152........ 121
Figure S153........ 122
Figure S154........ 122
Figure S155........ 123
Figure S156........ 123

Figure S157.. 124
Figure S158.. 124
Figure S159.. 125
Figure S160.. 125
Figure S161.. 126
Figure S162.. 126
Figure S163.. 127
Figure S164.. 128
Figure S165.. 128
Figure S166.. 129
Figure S167.. 129
Figure S168.. 130
Figure S169.. 130
Figure S170.. 131
Figure S171.. 132
Figure S172.. 133
Figure S173.. 134
Figure S174.. 135
Figure S175.. 136
Figure S176.. 137
Figure S177.. 138
Figure S178.. 139
Figure S179.. 140

Table S1A. Results the from systematic literature searches.

Database	*Result*	*Date*
PubMed	839	May 15, 2024
Embase	2,020	May 15, 2024
Cochrane	458	May 15, 2024
Total before removing duplicates	3,317	
Total after removing duplicates	2,428 (889 *duplicates removed*)	

Table S1B. Search strategy in PubMed.

Search	*Query*	*Results*
#9	**Search:** ((("Dietary Supplements"[Mesh]) OR (dietary suppl*[Title/Abstract])) OR (food supplement*[Title/Abstract])) OR (nutraceutic*[Title/Abstract])) OR (nutriceutic*[Title/Abstract])) OR (neutraceutic*[Title/Abstract])) OR (herbal supplement*[Title/Abstract])) OR (nutritional supplement*[Title/Abstract])) OR (("Micronutrients" [Pharmacological Action]) OR "Micronutrients"[Mesh])) OR (micronutrien*[Title/Abstract] OR trace element*[Title/Abstract] OR vitamin*[Title/Abstract])) OR (("Antioxidants" [Pharmacological Action]) OR "Antioxidants"[Mesh])) OR (antioxidant*[Title/Abstract])) OR (anti-oxidant*[Title/Abstract])) OR (probiotic*[Title/Abstract])) OR ("Vitamins" [Pharmacological Action])) OR ("Vitamin A"[Mesh])) OR ("Ascorbic Acid"[Mesh])) OR ("Vitamin D"[Mesh])) OR ("Vitamin E"[Mesh])) OR ("Vitamin K"[Mesh])) OR ("Thiamine"[Mesh])) OR ("Riboflavin"[Mesh])) OR ("Niacinamide"[Mesh])) OR ("Pantothenic Acid"[Mesh])) OR ("Vitamin B 6"[Mesh])) OR ("Biotin"[Mesh])) OR ("Folic Acid"[Mesh])) OR ("Vitamin B 12"[Mesh])) OR ("Sodium"[Mesh])) OR ("Potassium"[Mesh] OR "Potassium, Dietary"[Mesh])) OR ("Calcium"[Mesh])) OR ("Magnesium"[Mesh])) OR ("Phosphorus"[Mesh])) OR ("Chlorides"[Mesh])) OR ("Copper"[Mesh])) OR ("Iron, Dietary"[Mesh])) OR ("Chromium"[Mesh])) OR ("Molybdenum"[Mesh])) OR ("Zinc"[Mesh])) OR ("Manganese"[Mesh])) OR ("Selenium"[Mesh])) OR (ascorbic acid*[Title/Abstract] OR thiamin*[Title/Abstract] OR riboflavin*[Title/Abstract] OR niacin*[Title/Abstract] OR pantothenic*[Title/Abstract] OR biotin*[Title/Abstract] OR folic acid*[Title/Abstract] OR folate*[Title/Abstract] OR cobalamin*[Title/Abstract] OR sodium*[Title/Abstract] OR potassium*[Title/Abstract] OR calcium*[Title/Abstract] OR magnesi*[Title/Abstract] OR phosphorus*[Title/Abstract] OR chloride*[Title/Abstract] OR copper*[Title/Abstract] OR iron[Title/Abstract] OR iodine*[Title/Abstract] OR chromium*[Title/Abstract] OR molybdenum*[Title/Abstract] OR zinc*[Title/Abstract] OR manganese*[Title/Abstract] OR selenium*[Title/Abstract])) OR ("Fish Oils"[Mesh])) OR (fish oil*[Title/Abstract] OR omega 3[Title/Abstract] OR docosahexaenoic*[Title/Abstract] OR eicosapentaenoic*[Title/Abstract])) OR ("Carnitine"[Mesh])) OR (carnitine*[Title/Abstract])) OR (acetylcarnitine*[Title/Abstract])) OR ("Acetylcysteine"[Mesh])) OR (acetylcysteine*[Title/Abstract] OR acetyl-L-cysteine*[Title/Abstract])) OR ("Ubiquinone"[Mesh])) OR (ubiquinone[Title/Abstract] OR q10[Title/Abstract] OR coQ 10[Title/Abstract])) OR ((((("Glutathione"[Mesh]) OR "Arginine"[Mesh]) OR "Lycopene"[Mesh]) OR "beta Carotene"[Mesh]) OR "Choline"[Mesh])) OR (glutathione*[Title/Abstract] OR arginine*[Title/Abstract] OR lycopene*[Title/Abstract] OR beta carotene*[Title/Abstract] OR betacarotene*[Title/Abstract] OR choline*[Title/Abstract] OR "Sodium Chloride, Dietary"[Mesh] OR "Phosphorus, Dietary"[Mesh] OR "Sodium, Dietary"[Mesh] OR "Calcium, Dietary"[Mesh])) AND ((("Infertility, Male"[Mesh]) OR (male infertilit*[Title/Abstract] OR male fertilit*[Title/Abstract])) OR (male reproducti*[Title/Abstract])))) AND (((((((("Pregnancy Rate"[Mesh]) OR "Pregnancy"[Mesh]) OR ("Semen Analysis"[Mesh])) OR (semen qualit*[Title/Abstract] OR semen motilit*[Title/Abstract] OR semen morpholog*[Title/Abstract] OR semen count*[Title/Abstract] OR semen concentrat*[Title/Abstract] OR semen parameter*[Title/Abstract] OR semen analys*[Title/Abstract])) OR (sperm qualit*[Title/Abstract] OR sperm motilit*[Title/Abstract] OR sperm morpholog*[Title/Abstract] OR sperm count*[Title/Abstract] OR sperm concentrat*[Title/Abstract] OR sperm parameter*[Title/Abstract] OR sperm analys*[Title/Abstract])) OR ("DNA	839

	Fragmentation"[Mesh])) OR (dna fragmentati*[Text Word])) OR (pregnan*[Text Word] OR live birth*[Text Word]))) AND ("Randomized Controlled Trial"[pt] OR "Controlled Clinical Trial"[pt] OR "Pragmatic Clinical Trial"[pt] OR "Equivalence Trial"[pt] OR "Clinical Trial, Phase III"[pt] OR "Randomized Controlled Trials as Topic"[mh] OR "Controlled Clinical Trials as Topic"[mh] OR "Random Allocation"[mh] OR "Double-Blind Method"[mh] OR "Single-Blind Method"[mh] OR Placebos[Mesh:NoExp] OR "Control Groups"[mh] OR (random*[tiab] OR sham[tiab] OR placebo*[tiab]) OR ((singl*[tiab] OR doubl*[tiab]) AND (blind*[tiab] OR dumm*[tiab] OR mask*[tiab])) OR ((tripl*[tiab] OR trebl*[tiab]) AND (blind*[tiab] OR dumm*[tiab] OR mask*[tiab])) OR (control*[tiab] AND (study[tiab] OR studies[tiab] OR trial*[tiab] OR group*[tiab])) OR (Nonrandom*[tiab] OR "non random*"[tiab] OR "non-random*"[tiab] OR "quasi-random*"[tiab] OR quasirandom*[tiab]) OR allocated[tiab] OR (("open label"[tiab] OR "open-label"[tiab]) AND (study[tiab] OR studies[tiab] OR trial*[tiab])) OR ((equivalence[tiab] OR superiority[tiab] OR "non-inferiority"[tiab] OR noninferiority[tiab]) AND (study[tiab] OR studies[tiab] OR trial*[tiab])) OR ("pragmatic study"[tiab] OR "pragmatic studies"[tiab]) OR ((pragmatic[tiab] OR practical[tiab]) AND trial*[tiab]) OR ((quasiexperimental[tiab] OR "quasi-experimental"[tiab]) AND (study[tiab] OR studies[tiab] OR trial*[tiab])) OR (phase[ti] AND (III[ti] OR 3[ti]) AND (study[ti] OR studies[ti] OR trial*[ti])) OR (phase[ot] AND (III[ot] OR 3[ot]) AND (study[ot] OR studies[ot] OR trial*[ot])))) NOT (((("Animals"[Mesh]) NOT "Humans"[Mesh]) OR rat[Title] OR rats[Title] OR mouse[Title] OR mice[Title])) **Sort by:** Publication Date	
#8	**Search:** ((("Animals"[Mesh]) NOT "Humans"[Mesh]) OR rat[Title] OR rats[Title] OR mouse[Title] OR mice[Title]) **Sort by:** Publication Date	5,473,739
#7	**Search:** ((("Dietary Supplements"[Mesh]) OR (dietary suppl*[Title/Abstract])) OR (food supplement*[Title/Abstract])) OR (nutraceutic*[Title/Abstract])) OR (nutriceutic*[Title/Abstract])) OR (neutraceutic*[Title/Abstract])) OR (herbal supplement*[Title/Abstract])) OR (nutritional supplement*[Title/Abstract])) OR (("Micronutrients" [Pharmacological Action]) OR "Micronutrients"[Mesh])) OR (micronutrien*[Title/Abstract] OR trace element*[Title/Abstract] OR vitamin*[Title/Abstract])) OR (("Antioxidants" [Pharmacological Action]) OR "Antioxidants"[Mesh])) OR (antioxidant*[Title/Abstract])) OR (anti-oxidant*[Title/Abstract])) OR (probiotic*[Title/Abstract])) OR ("Vitamins" [Pharmacological Action])) OR ("Vitamin A"[Mesh])) OR ("Ascorbic Acid"[Mesh])) OR ("Vitamin D"[Mesh])) OR ("Vitamin E"[Mesh])) OR ("Vitamin K"[Mesh])) OR ("Thiamine"[Mesh])) OR ("Riboflavin"[Mesh])) OR ("Niacinamide"[Mesh])) OR ("Pantothenic Acid"[Mesh])) OR ("Vitamin B 6"[Mesh])) OR ("Biotin"[Mesh])) OR ("Folic Acid"[Mesh])) OR ("Vitamin B 12"[Mesh])) OR ("Sodium"[Mesh])) OR ("Potassium"[Mesh] OR "Potassium, Dietary"[Mesh])) OR ("Calcium"[Mesh])) OR ("Magnesium"[Mesh])) OR ("Phosphorus"[Mesh])) OR ("Chlorides"[Mesh])) OR ("Copper"[Mesh])) OR ("Iron, Dietary"[Mesh])) OR ("Chromium"[Mesh])) OR ("Molybdenum"[Mesh])) OR ("Zinc"[Mesh])) OR ("Manganese"[Mesh])) OR ("Selenium"[Mesh])) OR (ascorbic acid*[Title/Abstract] OR thiamin*[Title/Abstract] OR riboflavin*[Title/Abstract] OR niacin*[Title/Abstract] OR pantothenic*[Title/Abstract] OR biotin*[Title/Abstract] OR folic acid*[Title/Abstract] OR folate*[Title/Abstract] OR cobalamin*[Title/Abstract] OR sodium*[Title/Abstract] OR potassium*[Title/Abstract] OR calcium*[Title/Abstract] OR magnesi*[Title/Abstract] OR phosphorus*[Title/Abstract] OR chloride*[Title/Abstract] OR copper*[Title/Abstract] OR iron[Title/Abstract] OR iodine*[Title/Abstract] OR chromium*[Title/Abstract] OR molybdenum*[Title/Abstract] OR zinc*[Title/Abstract] OR manganese*[Title/Abstract] OR selenium*[Title/Abstract])) OR ("Fish Oils"[Mesh])) OR (fish oil*[Title/Abstract] OR omega 3[Title/Abstract] OR docosahexaenoic*[Title/Abstract] OR eicosapentaenoic*[Title/Abstract])) OR ("Carnitine"[Mesh])) OR (carnitine*[Title/Abstract])) OR (acetylcarnitine*[Title/Abstract])) OR ("Acetylcysteine"[Mesh])) OR (acetylcysteine*[Title/Abstract] OR acetyl-L-cysteine*[Title/Abstract])) OR ("Ubiquinone"[Mesh])) OR (ubiquinone[Title/Abstract] OR q10[Title/Abstract] OR coQ 10[Title/Abstract])) OR ((((("Glutathione"[Mesh]) OR "Arginine"[Mesh]) OR "Lycopene"[Mesh]) OR "beta Carotene"[Mesh]) OR "Choline"[Mesh])) OR (glutathione*[Title/Abstract] OR arginine*[Title/Abstract] OR lycopene*[Title/Abstract] OR beta carotene*[Title/Abstract] OR	1,551

	betacarotene*[Title/Abstract] OR choline*[Title/Abstract] OR "Sodium Chloride, Dietary"[Mesh] OR "Phosphorus, Dietary"[Mesh] OR "Sodium, Dietary"[Mesh] OR "Calcium, Dietary"[Mesh])) AND ((("Infertility, Male"[Mesh]) OR (male infertilit*[Title/Abstract] OR male fertilit*[Title/Abstract])) OR (male reproducti*[Title/Abstract]))) AND ((((((("Pregnancy Rate"[Mesh]) OR "Pregnancy"[Mesh]) OR ("Semen Analysis"[Mesh])) OR (semen qualit*[Title/Abstract] OR semen motilit*[Title/Abstract] OR semen morpholog*[Title/Abstract] OR semen count*[Title/Abstract] OR semen concentrat*[Title/Abstract] OR semen parameter*[Title/Abstract] OR semen analys*[Title/Abstract])) OR (sperm qualit*[Title/Abstract] OR sperm motilit*[Title/Abstract] OR sperm morpholog*[Title/Abstract] OR sperm count*[Title/Abstract] OR sperm concentrat*[Title/Abstract] OR sperm parameter*[Title/Abstract] OR sperm analys*[Title/Abstract])) OR ("DNA Fragmentation"[Mesh])) OR (dna fragmentati*[Text Word])) OR (pregnan*[Text Word] OR live birth*[Text Word]))) AND ("Randomized Controlled Trial"[pt] OR "Controlled Clinical Trial"[pt] OR "Pragmatic Clinical Trial"[pt] OR "Equivalence Trial"[pt] OR "Clinical Trial, Phase III"[pt] OR "Randomized Controlled Trials as Topic"[mh] OR "Controlled Clinical Trials as Topic"[mh] OR "Random Allocation"[mh] OR "Double-Blind Method"[mh] OR "Single-Blind Method"[mh] OR Placebos[Mesh:NoExp] OR "Control Groups"[mh] OR (random*[tiab] OR sham[tiab] OR placebo*[tiab]) OR ((singl*[tiab] OR doubl*[tiab]) AND (blind*[tiab] OR dumm*[tiab] OR mask*[tiab])) OR ((tripl*[tiab] OR trebl*[tiab]) AND (blind*[tiab] OR dumm*[tiab] OR mask*[tiab])) OR (control*[tiab] AND (study[tiab] OR studies[tiab] OR trial*[tiab] OR group*[tiab])) OR (Nonrandom*[tiab] OR "non random*"[tiab] OR "non-random*"[tiab] OR "quasi-random*"[tiab] OR quasirandom*[tiab]) OR allocated[tiab] OR (("open label"[tiab] OR "open-label"[tiab]) AND (study[tiab] OR studies[tiab] OR trial*[tiab])) OR ((equivalence[tiab] OR superiority[tiab] OR "non-inferiority"[tiab] OR noninferiority[tiab]) AND (study[tiab] OR studies[tiab] OR trial*[tiab])) OR ("pragmatic study"[tiab] OR "pragmatic studies"[tiab]) OR ((pragmatic[tiab] OR practical[tiab]) AND trial*[tiab]) OR ((quasiexperimental[tiab] OR "quasi-experimental"[tiab]) AND (study[tiab] OR studies[tiab] OR trial*[tiab])) OR (phase[ti] AND (III[ti] OR 3[ti]) AND (study[ti] OR studies[ti] OR trial*[ti])) OR (phase[ot] AND (III[ot] OR 3[ot]) AND (study[ot] OR studies[ot] OR trial*[ot]))) **Sort by:** Publication Date	
#6	**Search:** "Randomized Controlled Trial"[pt] OR "Controlled Clinical Trial"[pt] OR "Pragmatic Clinical Trial"[pt] OR "Equivalence Trial"[pt] OR "Clinical Trial, Phase III"[pt] OR "Randomized Controlled Trials as Topic"[mh] OR "Controlled Clinical Trials as Topic"[mh] OR "Random Allocation"[mh] OR "Double-Blind Method"[mh] OR "Single-Blind Method"[mh] OR Placebos[Mesh:NoExp] OR "Control Groups"[mh] OR (random*[tiab] OR sham[tiab] OR placebo*[tiab]) OR ((singl*[tiab] OR doubl*[tiab]) AND (blind*[tiab] OR dumm*[tiab] OR mask*[tiab])) OR ((tripl*[tiab] OR trebl*[tiab]) AND (blind*[tiab] OR dumm*[tiab] OR mask*[tiab])) OR (control*[tiab] AND (study[tiab] OR studies[tiab] OR trial*[tiab] OR group*[tiab])) OR (Nonrandom*[tiab] OR "non random*"[tiab] OR "non-random*"[tiab] OR "quasi-random*"[tiab] OR quasirandom*[tiab]) OR allocated[tiab] OR (("open label"[tiab] OR "open-label"[tiab]) AND (study[tiab] OR studies[tiab] OR trial*[tiab])) OR ((equivalence[tiab] OR superiority[tiab] OR "non-inferiority"[tiab] OR noninferiority[tiab]) AND (study[tiab] OR studies[tiab] OR trial*[tiab])) OR ("pragmatic study"[tiab] OR "pragmatic studies"[tiab]) OR ((pragmatic[tiab] OR practical[tiab]) AND trial*[tiab]) OR ((quasiexperimental[tiab] OR "quasi-experimental"[tiab]) AND (study[tiab] OR studies[tiab] OR trial*[tiab])) OR (phase[ti] AND (III[ti] OR 3[ti]) AND (study[ti] OR studies[ti] OR trial*[ti])) OR (phase[ot] AND (III[ot] OR 3[ot]) AND (study[ot] OR studies[ot] OR trial*[ot])) **Sort by:** Publication Date	4,561,929
#5	**Search:** ((("Dietary Supplements"[Mesh]) OR (dietary suppl*[Title/Abstract])) OR (food supplement*[Title/Abstract])) OR (nutraceutic*[Title/Abstract])) OR (nutriceutic*[Title/Abstract])) OR (neutraceutic*[Title/Abstract])) OR (herbal supplement*[Title/Abstract])) OR (nutritional supplement*[Title/Abstract])) OR (("Micronutrients" [Pharmacological Action]) OR "Micronutrients"[Mesh])) OR (micronutrien*[Title/Abstract] OR trace element*[Title/Abstract] OR vitamin*[Title/Abstract])) OR (("Antioxidants" [Pharmacological Action]) OR "Antioxidants"[Mesh])) OR (antioxidant*[Title/Abstract])) OR (anti-oxidant*[Title/Abstract]))	3,637

	OR (probiotic*[Title/Abstract])) OR ("Vitamins" [Pharmacological Action])) OR ("Vitamin A"[Mesh])) OR ("Ascorbic Acid"[Mesh])) OR ("Vitamin D"[Mesh])) OR ("Vitamin E"[Mesh])) OR ("Vitamin K"[Mesh])) OR ("Thiamine"[Mesh])) OR ("Riboflavin"[Mesh])) OR ("Niacinamide"[Mesh])) OR ("Pantothenic Acid"[Mesh])) OR ("Vitamin B 6"[Mesh])) OR ("Biotin"[Mesh])) OR ("Folic Acid"[Mesh])) OR ("Vitamin B 12"[Mesh])) OR ("Sodium"[Mesh])) OR ("Potassium"[Mesh] OR "Potassium, Dietary"[Mesh])) OR ("Calcium"[Mesh])) OR ("Magnesium"[Mesh])) OR ("Phosphorus"[Mesh])) OR ("Chlorides"[Mesh])) OR ("Copper"[Mesh])) OR ("Iron, Dietary"[Mesh])) OR ("Chromium"[Mesh])) OR ("Molybdenum"[Mesh])) OR ("Zinc"[Mesh])) OR ("Manganese"[Mesh])) OR ("Selenium"[Mesh])) OR (ascorbic acid*[Title/Abstract] OR thiamin*[Title/Abstract] OR riboflavin*[Title/Abstract] OR niacin*[Title/Abstract] OR pantothenic*[Title/Abstract] OR biotin*[Title/Abstract] OR folic acid*[Title/Abstract] OR folate*[Title/Abstract] OR cobalamin*[Title/Abstract] OR sodium*[Title/Abstract] OR potassium*[Title/Abstract] OR calcium*[Title/Abstract] OR magnesi*[Title/Abstract] OR phosphorus*[Title/Abstract] OR chloride*[Title/Abstract] OR copper*[Title/Abstract] OR iron[Title/Abstract] OR iodine*[Title/Abstract] OR chromium*[Title/Abstract] OR molybdenum*[Title/Abstract] OR zinc*[Title/Abstract] OR manganese*[Title/Abstract] OR selenium*[Title/Abstract])) OR ("Fish Oils"[Mesh])) OR (fish oil*[Title/Abstract] OR omega 3[Title/Abstract] OR docosahexaenoic*[Title/Abstract] OR eicosapentaenoic*[Title/Abstract])) OR ("Carnitine"[Mesh])) OR (carnitine*[Title/Abstract])) OR (acetylcarnitine*[Title/Abstract])) OR ("Acetylcysteine"[Mesh])) OR (acetylcysteine*[Title/Abstract] OR acetyl-L-cysteine*[Title/Abstract])) OR ("Ubiquinone"[Mesh])) OR (ubiquinone[Title/Abstract] OR q10[Title/Abstract] OR coQ 10[Title/Abstract])) OR ((((("Glutathione"[Mesh]) OR "Arginine"[Mesh]) OR "Lycopene"[Mesh]) OR "beta Carotene"[Mesh]) OR "Choline"[Mesh])) OR (glutathione*[Title/Abstract] OR arginine*[Title/Abstract] OR lycopene*[Title/Abstract] OR beta carotene*[Title/Abstract] OR betacarotene*[Title/Abstract] OR choline*[Title/Abstract] OR "Sodium Chloride, Dietary"[Mesh] OR "Phosphorus, Dietary"[Mesh] OR "Sodium, Dietary"[Mesh] OR "Calcium, Dietary"[Mesh])) AND ((("Infertility, Male"[Mesh]) OR (male infertilit*[Title/Abstract] OR male fertilit*[Title/Abstract])) OR (male reproducti*[Title/Abstract]))) AND ((((((("Pregnancy Rate"[Mesh]) OR "Pregnancy"[Mesh]) OR ("Semen Analysis"[Mesh])) OR (semen qualit*[Title/Abstract] OR semen motilit*[Title/Abstract] OR semen morpholog*[Title/Abstract] OR semen count*[Title/Abstract] OR semen concentrat*[Title/Abstract] OR semen parameter*[Title/Abstract] OR semen analys*[Title/Abstract])) OR (sperm qualit*[Title/Abstract] OR sperm motilit*[Title/Abstract] OR sperm morpholog*[Title/Abstract] OR sperm count*[Title/Abstract] OR sperm concentrat*[Title/Abstract] OR sperm parameter*[Title/Abstract] OR sperm analys*[Title/Abstract])) OR ("DNA Fragmentation"[Mesh])) OR (dna fragmentati*[Text Word])) OR (pregnan*[Text Word] OR live birth*[Text Word])) **Sort by:** Publication Date	
#4	**Search:** ((((((("Pregnancy Rate"[Mesh]) OR "Pregnancy"[Mesh]) OR ("Semen Analysis"[Mesh])) OR (semen qualit*[Title/Abstract] OR semen motilit*[Title/Abstract] OR semen morpholog*[Title/Abstract] OR semen count*[Title/Abstract] OR semen concentrat*[Title/Abstract] OR semen parameter*[Title/Abstract] OR semen analys*[Title/Abstract])) OR (sperm qualit*[Title/Abstract] OR sperm motilit*[Title/Abstract] OR sperm morpholog*[Title/Abstract] OR sperm count*[Title/Abstract] OR sperm concentrat*[Title/Abstract] OR sperm parameter*[Title/Abstract] OR sperm analys*[Title/Abstract])) OR ("DNA Fragmentation"[Mesh])) OR (dna fragmentati*[Text Word])) OR (pregnan*[Text Word] OR live birth*[Text Word]) **Sort by:** Publication Date	1,261,807
#3	**Search:** ((("Dietary Supplements"[Mesh]) OR (dietary suppl*[Title/Abstract])) OR (food supplement*[Title/Abstract])) OR (nutraceutic*[Title/Abstract])) OR (nutriceutic*[Title/Abstract])) OR (neutraceutic*[Title/Abstract])) OR (herbal supplement*[Title/Abstract])) OR (nutritional supplement*[Title/Abstract])) OR (("Micronutrients" [Pharmacological Action]) OR "Micronutrients"[Mesh])) OR (micronutrien*[Title/Abstract] OR trace element*[Title/Abstract] OR vitamin*[Title/Abstract])) OR (("Antioxidants" [Pharmacological Action]) OR "Antioxidants"[Mesh])) OR (antioxidant*[Title/Abstract])) OR (anti-oxidant*[Title/Abstract])) OR (probiotic*[Title/Abstract])) OR ("Vitamins" [Pharmacological Action])) OR	6,573

	("Vitamin A"[Mesh])) OR ("Ascorbic Acid"[Mesh])) OR ("Vitamin D"[Mesh])) OR ("Vitamin E"[Mesh])) OR ("Vitamin K"[Mesh])) OR ("Thiamine"[Mesh])) OR ("Riboflavin"[Mesh])) OR ("Niacinamide"[Mesh])) OR ("Pantothenic Acid"[Mesh])) OR ("Vitamin B 6"[Mesh])) OR ("Biotin"[Mesh])) OR ("Folic Acid"[Mesh])) OR ("Vitamin B 12"[Mesh])) OR ("Sodium"[Mesh])) OR ("Potassium"[Mesh] OR "Potassium, Dietary"[Mesh])) OR ("Calcium"[Mesh])) OR ("Magnesium"[Mesh])) OR ("Phosphorus"[Mesh])) OR ("Chlorides"[Mesh])) OR ("Copper"[Mesh])) OR ("Iron, Dietary"[Mesh])) OR ("Chromium"[Mesh])) OR ("Molybdenum"[Mesh])) OR ("Zinc"[Mesh])) OR ("Manganese"[Mesh])) OR ("Selenium"[Mesh])) OR (ascorbic acid*[Title/Abstract] OR thiamin*[Title/Abstract] OR riboflavin*[Title/Abstract] OR niacin*[Title/Abstract] OR pantothenic*[Title/Abstract] OR biotin*[Title/Abstract] OR folic acid*[Title/Abstract] OR folate*[Title/Abstract] OR cobalamin*[Title/Abstract] OR sodium*[Title/Abstract] OR potassium*[Title/Abstract] OR calcium*[Title/Abstract] OR magnesi*[Title/Abstract] OR phosphorus*[Title/Abstract] OR chloride*[Title/Abstract] OR copper*[Title/Abstract] OR iron[Title/Abstract] OR iodine*[Title/Abstract] OR chromium*[Title/Abstract] OR molybdenum*[Title/Abstract] OR zinc*[Title/Abstract] OR manganese*[Title/Abstract] OR selenium*[Title/Abstract])) OR ("Fish Oils"[Mesh])) OR (fish oil*[Title/Abstract] OR omega 3[Title/Abstract] OR docosahexaenoic*[Title/Abstract] OR eicosapentaenoic*[Title/Abstract])) OR ("Carnitine"[Mesh])) OR (carnitine*[Title/Abstract])) OR (acetylcarnitine*[Title/Abstract])) OR ("Acetylcysteine"[Mesh])) OR (acetylcysteine*[Title/Abstract] OR acetyl-L-cysteine*[Title/Abstract])) OR ("Ubiquinone"[Mesh])) OR (ubiquinone[Title/Abstract] OR q10[Title/Abstract] OR coQ 10[Title/Abstract])) OR ((((("Glutathione"[Mesh]) OR "Arginine"[Mesh]) OR "Lycopene"[Mesh]) OR "beta Carotene"[Mesh]) OR "Choline"[Mesh])) OR (glutathione*[Title/Abstract] OR arginine*[Title/Abstract] OR lycopene*[Title/Abstract] OR beta carotene*[Title/Abstract] OR betacarotene*[Title/Abstract] OR choline*[Title/Abstract] OR "Sodium Chloride, Dietary"[Mesh] OR "Phosphorus, Dietary"[Mesh] OR "Sodium, Dietary"[Mesh] OR "Calcium, Dietary"[Mesh])) AND ((("Infertility, Male"[Mesh]) OR (male infertilit*[Title/Abstract] OR male fertilit*[Title/Abstract])) OR (male reproducti*[Title/Abstract])) **Sort by:** Publication Date	
#2	**Search:** (("Infertility, Male"[Mesh]) OR (male infertilit*[Title/Abstract] OR male fertilit*[Title/Abstract])) OR (male reproducti*[Title/Abstract]) **Sort by:** Publication Date	51,349
#1	**Search:** ((("Dietary Supplements"[Mesh]) OR (dietary suppl*[Title/Abstract])) OR (food supplement*[Title/Abstract])) OR (nutraceutic*[Title/Abstract])) OR (nutriceutic*[Title/Abstract])) OR (neutraceutic*[Title/Abstract])) OR (herbal supplement*[Title/Abstract])) OR (nutritional supplement*[Title/Abstract])) OR (("Micronutrients" [Pharmacological Action]) OR "Micronutrients"[Mesh])) OR (micronutrien*[Title/Abstract] OR trace element*[Title/Abstract] OR vitamin*[Title/Abstract])) OR (("Antioxidants" [Pharmacological Action]) OR "Antioxidants"[Mesh])) OR (antioxidant*[Title/Abstract])) OR (anti-oxidant*[Title/Abstract])) OR (probiotic*[Title/Abstract])) OR ("Vitamins" [Pharmacological Action])) OR ("Vitamin A"[Mesh])) OR ("Ascorbic Acid"[Mesh])) OR ("Vitamin D"[Mesh])) OR ("Vitamin E"[Mesh])) OR ("Vitamin K"[Mesh])) OR ("Thiamine"[Mesh])) OR ("Riboflavin"[Mesh])) OR ("Niacinamide"[Mesh])) OR ("Pantothenic Acid"[Mesh])) OR ("Vitamin B 6"[Mesh])) OR ("Biotin"[Mesh])) OR ("Folic Acid"[Mesh])) OR ("Vitamin B 12"[Mesh])) OR ("Sodium"[Mesh])) OR ("Potassium"[Mesh] OR "Potassium, Dietary"[Mesh])) OR ("Calcium"[Mesh])) OR ("Magnesium"[Mesh])) OR ("Phosphorus"[Mesh])) OR ("Chlorides"[Mesh])) OR ("Copper"[Mesh])) OR ("Iron, Dietary"[Mesh])) OR ("Chromium"[Mesh])) OR ("Molybdenum"[Mesh])) OR ("Zinc"[Mesh])) OR ("Manganese"[Mesh])) OR ("Selenium"[Mesh])) OR (ascorbic acid*[Title/Abstract] OR thiamin*[Title/Abstract] OR riboflavin*[Title/Abstract] OR niacin*[Title/Abstract] OR pantothenic*[Title/Abstract] OR biotin*[Title/Abstract] OR folic acid*[Title/Abstract] OR folate*[Title/Abstract] OR cobalamin*[Title/Abstract] OR sodium*[Title/Abstract] OR potassium*[Title/Abstract] OR calcium*[Title/Abstract] OR magnesi*[Title/Abstract] OR phosphorus*[Title/Abstract] OR chloride*[Title/Abstract] OR copper*[Title/Abstract] OR iron[Title/Abstract] OR iodine*[Title/Abstract] OR chromium*[Title/Abstract] OR molybdenum*[Title/Abstract] OR zinc*[Title/Abstract] OR manganese*[Title/Abstract] OR selenium*[Title/Abstract])) OR ("Fish	3,524,443

	Oils"[Mesh])) OR (fish oil*[Title/Abstract] OR omega 3[Title/Abstract] OR docosahexaenoic*[Title/Abstract] OR eicosapentaenoic*[Title/Abstract])) OR ("Carnitine"[Mesh])) OR (carnitine*[Title/Abstract])) OR (acetylcarnitine*[Title/Abstract])) OR ("Acetylcysteine"[Mesh])) OR (acetylcysteine*[Title/Abstract] OR acetyl-L-cysteine*[Title/Abstract])) OR ("Ubiquinone"[Mesh])) OR (ubiquinone[Title/Abstract] OR q10[Title/Abstract] OR coQ 10[Title/Abstract])) OR ((((("Glutathione"[Mesh]) OR "Arginine"[Mesh]) OR "Lycopene"[Mesh]) OR "beta Carotene"[Mesh]) OR "Choline"[Mesh])) OR (glutathione*[Title/Abstract] OR arginine*[Title/Abstract] OR lycopene*[Title/Abstract] OR beta carotene*[Title/Abstract] OR betacarotene*[Title/Abstract] OR choline*[Title/Abstract] OR "Sodium Chloride, Dietary"[Mesh] OR "Phosphorus, Dietary"[Mesh] OR "Sodium, Dietary"[Mesh] OR "Calcium, Dietary"[Mesh]) **Sort by:** Publication Date	

Search filter used: RCT / CCT - PubMed. In: CADTH Search Filters Database. Ottawa: CADTH; 2024: https://searchfilters.cadth.ca/link/108. Accessed 2024-05-15.

Table S1C. Search strategy in Embase.

No.	*Query*	*Results*
#8	((('dietary supplement'/exp OR 'diet supplementation'/exp OR (((diet* OR food OR herbal OR nutrition*) NEAR/1 suppl*):ti,ab,kw) OR neutraceutic*:ti,ab,kw OR nutraceutic*:ti,ab,kw OR nutriceutic*:ti,ab,kw OR 'nutraceutical'/exp OR 'trace element'/exp OR micronutrient*:ti,ab,kw OR 'trace element*':ti,ab,kw OR vitamin*:ti,ab,kw OR 'antioxidant'/exp OR 'vitamin'/exp OR antioxidant*:ti,ab,kw OR 'anti-oxidant*':ti,ab,kw OR probiotic*:ti,ab,kw OR 'probiotic agent'/exp OR 'sodium'/exp OR 'potassium'/exp OR 'potassium intake'/exp OR 'chloride'/exp OR 'mineral intake'/exp OR 'chromium'/exp OR 'molybdenum'/exp OR 'zinc'/exp OR 'manganese'/exp OR 'selenium'/exp OR 'ascorbic acid*':ti,ab,kw OR thiamin*:ti,ab,kw OR riboflavin*:ti,ab,kw OR niacin*:ti,ab,kw OR pantothenic*:ti,ab,kw OR biotin*:ti,ab,kw OR 'folic acid*':ti,ab,kw OR folate*:ti,ab,kw OR cobalamin*:ti,ab,kw OR sodium*:ti,ab,kw OR potassium*:ti,ab,kw OR calcium*:ti,ab,kw OR magnesi*:ti,ab,kw OR phosphorus*:ti,ab,kw OR chloride*:ti,ab,kw OR copper*:ti,ab,kw OR iron:ti,ab,kw OR iodine*:ti,ab,kw OR chromium*:ti,ab,kw OR molybdenum*:ti,ab,kw OR zinc*:ti,ab,kw OR manganese*:ti,ab,kw OR selenium*:ti,ab,kw OR 'fish oil'/exp OR 'fish oil*':ti,ab,kw OR 'omega 3':ti,ab,kw OR docosahexaenoic*:ti,ab,kw OR eicosapentaenoic*:ti,ab,kw OR 'carnitine'/exp OR 'acetylcysteine'/exp OR 'ubiquinone'/exp OR 'glutathione'/exp OR 'arginine'/exp OR 'choline'/exp OR carnitine*:ti,ab,kw OR acetylcarnitine*:ti,ab,kw OR acetylcysteine*:ti,ab,kw OR 'acetyl-l-cysteine*':ti,ab,kw OR ubiquinone:ti,ab,kw OR q10:ti,ab,kw OR 'coq 10':ti,ab,kw OR glutathione*:ti,ab,kw OR arginine*:ti,ab,kw OR lycopene*:ti,ab,kw OR 'beta carotene*':ti,ab,kw OR betacarotene*:ti,ab,kw OR choline*:ti,ab,kw OR 'sodium intake'/de OR 'phosphate intake'/de OR 'salt intake'/de OR 'calcium intake'/de) AND ('male infertility'/exp OR ((male NEAR/1 (infertilit* OR fertilit* OR reproduct*)):ti,ab,kw)) AND ((((sperm OR semen) NEAR/1 (qualit* OR motilit* OR morpholog* OR count* OR concentrat* OR parameter* OR analys*)):ti,ab,kw) OR pregnan*:ti,ab,kw OR 'live birth*':ti,ab,kw OR 'dna fragmentati*':ti,ab,kw OR 'pregnancy'/exp OR 'pregnancy rate'/exp OR 'semen analysis'/exp OR 'sperm count'/exp OR 'sperm quality'/exp OR 'spermatozoon motility'/exp OR 'semen parameters'/de OR 'sperm morphology'/de OR 'spermatozoon density'/de OR 'sperm dna fragmentation'/de OR 'dna fragmentation'/de OR 'live birth'/de OR 'live birth rate'/de)) AND ('controlled clinical trial'/exp OR 'randomized controlled trial'/exp OR (((random* OR controlled* OR crossover OR 'cross over' OR blind* OR mask*) NEAR/3 (trial* OR study OR studies OR analy*)):ti,ab,de) OR rct:ti,ab,de OR (((single OR double OR triple) NEAR/2 (blind* OR mask*)):ti,ab,de) OR placebo:ti,ab,de)) NOT ('animal'/exp NOT 'human'/exp OR rat:ti OR rats:ti OR mouse:ti OR mice:ti)	2020
#7	'animal'/exp NOT 'human'/exp OR rat:ti OR rats:ti OR mouse:ti OR mice:ti	6342984
#6	(('dietary supplement'/exp OR 'diet supplementation'/exp OR (((diet* OR food OR herbal OR nutrition*) NEAR/1 suppl*):ti,ab,kw) OR neutraceutic*:ti,ab,kw OR nutraceutic*:ti,ab,kw OR nutriceutic*:ti,ab,kw OR 'nutraceutical'/exp OR 'trace element'/exp OR micronutrient*:ti,ab,kw OR 'trace element*':ti,ab,kw OR vitamin*:ti,ab,kw OR 'antioxidant'/exp OR 'vitamin'/exp OR antioxidant*:ti,ab,kw OR 'anti-oxidant*':ti,ab,kw OR	3572

	probiotic*:ti,ab,kw OR 'probiotic agent'/exp OR 'sodium'/exp OR 'potassium'/exp OR 'potassium intake'/exp OR 'chloride'/exp OR 'mineral intake'/exp OR 'chromium'/exp OR 'molybdenum'/exp OR 'zinc'/exp OR 'manganese'/exp OR 'selenium'/exp OR 'ascorbic acid*':ti,ab,kw OR thiamin*:ti,ab,kw OR riboflavin*:ti,ab,kw OR niacin*:ti,ab,kw OR pantothenic*:ti,ab,kw OR biotin*:ti,ab,kw OR 'folic acid*':ti,ab,kw OR folate*:ti,ab,kw OR cobalamin*:ti,ab,kw OR sodium*:ti,ab,kw OR potassium*:ti,ab,kw OR calcium*:ti,ab,kw OR magnesi*:ti,ab,kw OR phosphorus*:ti,ab,kw OR chloride*:ti,ab,kw OR copper*:ti,ab,kw OR iron:ti,ab,kw OR iodine*:ti,ab,kw OR chromium*:ti,ab,kw OR molybdenum*:ti,ab,kw OR zinc*:ti,ab,kw OR manganese*:ti,ab,kw OR selenium*:ti,ab,kw OR 'fish oil'/exp OR 'fish oil*':ti,ab,kw OR 'omega 3':ti,ab,kw OR docosahexaenoic*:ti,ab,kw OR eicosapentaenoic*:ti,ab,kw OR 'carnitine'/exp OR 'acetylcysteine'/exp OR 'ubiquinone'/exp OR 'glutathione'/exp OR 'arginine'/exp OR 'choline'/exp OR carnitine*:ti,ab,kw OR acetyl-carnitine*:ti,ab,kw OR acetylcysteine*:ti,ab,kw OR 'acetyl-l-cysteine*':ti,ab,kw OR ubiquinone:ti,ab,kw OR q10:ti,ab,kw OR 'coq 10':ti,ab,kw OR glutathione*:ti,ab,kw OR arginine*:ti,ab,kw OR lycopene*:ti,ab,kw OR 'beta carotene*':ti,ab,kw OR betacarotene*:ti,ab,kw OR choline*:ti,ab,kw OR 'sodium intake'/de OR 'phosphate intake'/de OR 'salt intake'/de OR 'calcium intake'/de) AND ('male infertility'/exp OR ((male NEAR/1 (infertilit* OR fertilit* OR reproduct*)):ti,ab,kw)) AND ((((sperm OR semen) NEAR/1 (qualit* OR motilit* OR morpholog* OR count* OR concentrat* OR parameter* OR analys*)):ti,ab,kw) OR pregnan*:ti,ab,kw OR 'live birth*':ti,ab,kw OR 'dna fragmentati*':ti,ab,kw OR 'pregnancy'/exp OR 'pregnancy rate'/exp OR 'semen analysis'/exp OR 'sperm count'/exp OR 'sperm quality'/exp OR 'spermatozoon motility'/exp OR 'semen parameters'/de OR 'sperm morphology'/de OR 'spermatozoon density'/de OR 'sperm dna fragmentation'/de OR 'dna fragmentation'/de OR 'live birth'/de OR 'live birth rate'/de)) AND ('controlled clinical trial'/exp OR 'randomized controlled trial'/exp OR (((random* OR controlled* OR crossover OR 'cross over' OR blind* OR mask*) NEAR/3 (trial* OR study OR studies OR analy*)):ti,ab,de) OR rct:ti,ab,de OR (((single OR double OR triple) NEAR/2 (blind* OR mask*)):ti,ab,de) OR placebo:ti,ab,de)	
#5	'controlled clinical trial'/exp OR 'randomized controlled trial'/exp OR (((random* OR controlled* OR crossover OR 'cross over' OR blind* OR mask*) NEAR/3 (trial* OR study OR studies OR analy*)):ti,ab,de) OR rct:ti,ab,de OR (((single OR double OR triple) NEAR/2 (blind* OR mask*)):ti,ab,de) OR placebo:ti,ab,de	11296286
#4	('dietary supplement'/exp OR 'diet supplementation'/exp OR (((diet* OR food OR herbal OR nutrition*) NEAR/1 suppl*):ti,ab,kw) OR neutraceutic*:ti,ab,kw OR nutraceutic*:ti,ab,kw OR nutriceutic*:ti,ab,kw OR 'nutraceutical'/exp OR 'trace element'/exp OR micronutrient*:ti,ab,kw OR 'trace element*':ti,ab,kw OR vitamin*:ti,ab,kw OR 'antioxidant'/exp OR 'vitamin'/exp OR antioxidant*:ti,ab,kw OR 'anti-oxidant*':ti,ab,kw OR probiotic*:ti,ab,kw OR 'probiotic agent'/exp OR 'sodium'/exp OR 'potassium'/exp OR 'potassium intake'/exp OR 'chloride'/exp OR 'mineral intake'/exp OR 'chromium'/exp OR 'molybdenum'/exp OR 'zinc'/exp OR 'manganese'/exp OR 'selenium'/exp OR 'ascorbic acid*':ti,ab,kw OR thiamin*:ti,ab,kw OR riboflavin*:ti,ab,kw OR niacin*:ti,ab,kw OR pantothenic*:ti,ab,kw OR biotin*:ti,ab,kw OR 'folic acid*':ti,ab,kw OR folate*:ti,ab,kw OR cobalamin*:ti,ab,kw OR sodium*:ti,ab,kw OR potassium*:ti,ab,kw OR calcium*:ti,ab,kw OR magnesi*:ti,ab,kw OR phosphorus*:ti,ab,kw OR chloride*:ti,ab,kw OR copper*:ti,ab,kw OR iron:ti,ab,kw OR iodine*:ti,ab,kw OR chromium*:ti,ab,kw OR molybdenum*:ti,ab,kw OR zinc*:ti,ab,kw OR manganese*:ti,ab,kw OR selenium*:ti,ab,kw OR 'fish oil'/exp OR 'fish oil*':ti,ab,kw OR 'omega 3':ti,ab,kw OR docosahexaenoic*:ti,ab,kw OR eicosapentaenoic*:ti,ab,kw OR 'carnitine'/exp OR 'acetylcysteine'/exp OR 'ubiquinone'/exp OR 'glutathione'/exp OR 'arginine'/exp OR 'choline'/exp OR carnitine*:ti,ab,kw OR acetyl-carnitine*:ti,ab,kw OR acetylcysteine*:ti,ab,kw OR 'acetyl-l-cysteine*':ti,ab,kw OR ubiquinone:ti,ab,kw OR q10:ti,ab,kw OR 'coq 10':ti,ab,kw OR glutathione*:ti,ab,kw OR arginine*:ti,ab,kw OR lycopene*:ti,ab,kw OR 'beta carotene*':ti,ab,kw OR betacarotene*:ti,ab,kw OR choline*:ti,ab,kw OR 'sodium intake'/de OR 'phosphate intake'/de OR 'salt intake'/de OR 'calcium intake'/de) AND ('male infertility'/exp OR ((male NEAR/1 (infertilit* OR fertilit* OR reproduct*)):ti,ab,kw)) AND ((((sperm OR semen) NEAR/1 (qualit* OR motilit* OR morpholog* OR count* OR concentrat* OR parameter* OR analys*)):ti,ab,kw) OR pregnan*:ti,ab,kw OR 'live birth*':ti,ab,kw OR 'dna fragmentati*':ti,ab,kw OR 'pregnancy'/exp OR 'pregnancy rate'/exp OR 'semen analysis'/exp	5973

	OR 'sperm count'/exp OR 'sperm quality'/exp OR 'spermatozoon motility'/exp OR 'semen parameters'/de OR 'sperm morphology'/de OR 'spermatozoon density'/de OR 'sperm dna fragmentation'/de OR 'dna fragmentation'/de OR 'live birth'/de OR 'live birth rate'/de)	
#3	(((sperm OR semen) NEAR/1 (qualit* OR motilit* OR morpholog* OR count* OR concentrat* OR parameter* OR analys*)):ti,ab,kw) OR pregnan*:ti,ab,kw OR 'live birth*':ti,ab,kw OR 'dna fragmentati*':ti,ab,kw OR 'pregnancy'/exp OR 'pregnancy rate'/exp OR 'semen analysis'/exp OR 'sperm count'/exp OR 'sperm quality'/exp OR 'spermatozoon motility'/exp OR 'semen parameters'/de OR 'sperm morphology'/de OR 'spermatozoon density'/de OR 'sperm dna fragmentation'/de OR 'dna fragmentation'/de OR 'live birth'/de OR 'live birth rate'/de	1331893
#2	'male infertility'/exp OR ((male NEAR/1 (infertilit* OR fertilit* OR reproduct*)):ti,ab,kw)	74097
#1	'dietary supplement'/exp OR 'diet supplementation'/exp OR (((diet* OR food OR herbal OR nutrition*) NEAR/1 suppl*):ti,ab,kw) OR neutraceutic*:ti,ab,kw OR nutraceutic*:ti,ab,kw OR nutriceutic*:ti,ab,kw OR 'nutraceutical'/exp OR 'trace element'/exp OR micronutrient*:ti,ab,kw OR 'trace element*':ti,ab,kw OR vitamin*:ti,ab,kw OR 'antioxidant'/exp OR 'vitamin'/exp OR antioxidant*:ti,ab,kw OR 'anti-oxidant*':ti,ab,kw OR probiotic*:ti,ab,kw OR 'probiotic agent'/exp OR 'sodium'/exp OR 'potassium'/exp OR 'potassium intake'/exp OR 'chloride'/exp OR 'mineral intake'/exp OR 'chromium'/exp OR 'molybdenum'/exp OR 'zinc'/exp OR 'manganese'/exp OR 'selenium'/exp OR 'ascorbic acid*':ti,ab,kw OR thiamin*:ti,ab,kw OR riboflavin*:ti,ab,kw OR niacin*:ti,ab,kw OR pantothenic*:ti,ab,kw OR biotin*:ti,ab,kw OR 'folic acid*':ti,ab,kw OR folate*:ti,ab,kw OR cobalamin*:ti,ab,kw OR sodium*:ti,ab,kw OR potassium*:ti,ab,kw OR calcium*:ti,ab,kw OR magnesi*:ti,ab,kw OR phosphorus*:ti,ab,kw OR chloride*:ti,ab,kw OR copper*:ti,ab,kw OR iron:ti,ab,kw OR iodine*:ti,ab,kw OR chromium*:ti,ab,kw OR molybdenum*:ti,ab,kw OR zinc*:ti,ab,kw OR manganese*:ti,ab,kw OR selenium*:ti,ab,kw OR 'fish oil'/exp OR 'fish oil*':ti,ab,kw OR 'omega 3':ti,ab,kw OR docosahexaenoic*:ti,ab,kw OR eicosapentaenoic*:ti,ab,kw OR 'carnitine'/exp OR 'acetylcysteine'/exp OR 'ubiquinone'/exp OR 'glutathione'/exp OR 'arginine'/exp OR 'choline'/exp OR carnitine*:ti,ab,kw OR acetylcarnitine*:ti,ab,kw OR acetylcysteine*:ti,ab,kw OR 'acetyl-l-cysteine*':ti,ab,kw OR ubiquinone:ti,ab,kw OR q10:ti,ab,kw OR 'coq 10':ti,ab,kw OR glutathione*:ti,ab,kw OR arginine*:ti,ab,kw OR lycopene*:ti,ab,kw OR 'beta carotene*':ti,ab,kw OR betacarotene*:ti,ab,kw OR choline*:ti,ab,kw OR 'sodium intake'/de OR 'phosphate intake'/de OR 'salt intake'/de OR 'calcium intake'/de	4152878

Table S1D. Search strategy in Cochrane Central Register of Controlled Trials.

ID	***Search***	***Hits***
#1	MeSH descriptor: [Infertility, Male] explode all trees	1027
#2	(male NEAR/1 (infertilit* OR fertilit* OR reproduct*)):ti,ab,kw	1732
#3	#1 OR #2	1940
#4	MeSH descriptor: [Dietary Supplements] explode all trees	19990
#5	((diet* OR food OR herbal OR nutrition*) NEAR/1 suppl*):ti,ab,kw	30503
#6	(neutraceutic* OR nutraceutic* OR nutriceutic*):ti,ab,kw	1034
#7	MeSH descriptor: [Micronutrients] explode all trees	7416
#8	(micronutrien* OR (trace NEXT element*) OR vitamin*):ti,ab,kw	41483
#9	MeSH descriptor: [Antioxidants] explode all trees	6600
#10	(antioxidant* OR (anti NEXT oxidant*) OR probiotic*):ti,ab,kw	27223
#11	MeSH descriptor: [Vitamins] explode all trees	5975
#12	MeSH descriptor: [Carotenoids] explode all trees	4707
#13	MeSH descriptor: [Vitamin B 12] explode all trees	1182
#14	MeSH descriptor: [Vitamin B 6] explode all trees	872
#15	MeSH descriptor: [Ascorbic Acid] explode all trees	2814
#16	MeSH descriptor: [Vitamin D] explode all trees	7921
#17	MeSH descriptor: [Vitamin E] explode all trees	3126
#18	MeSH descriptor: [Vitamin K] explode all trees	868
#19	MeSH descriptor: [Thiamine] explode all trees	389

#20	MeSH descriptor: [Riboflavin] explode all trees	563
#21	MeSH descriptor: [Niacinamide] explode all trees	1609
#22	MeSH descriptor: [Pantothenic Acid] explode all trees	122
#23	MeSH descriptor: [Biotin] explode all trees	71
#24	MeSH descriptor: [Folic Acid] explode all trees	4729
#25	MeSH descriptor: [Sodium] explode all trees	2788
#26	MeSH descriptor: [Potassium] explode all trees	2699
#27	MeSH descriptor: [Calcium] explode all trees	4743
#28	MeSH descriptor: [Magnesium] explode all trees	1533
#29	MeSH descriptor: [Phosphorus] explode all trees	966
#30	MeSH descriptor: [Chlorides] explode all trees	3558
#31	MeSH descriptor: [Copper] explode all trees	631
#32	MeSH descriptor: [Iron, Dietary] explode all trees	486
#33	MeSH descriptor: [Chromium] explode all trees	442
#34	MeSH descriptor: [Molybdenum] explode all trees	36
#35	MeSH descriptor: [Zinc] explode all trees	2063
#36	MeSH descriptor: [Manganese] explode all trees	99
#37	MeSH descriptor: [Selenium] explode all trees	975
#38	((ascorbic NEXT acid*) OR thiamin* OR riboflavin* OR niacin* OR pantothenic* OR biotin* OR (folic NEXT acid*) OR folate* OR cobalamin* OR sodium* OR potassium* OR calcium* OR magnesi* OR phosphorus* OR chloride* OR copper* OR iron OR iodine* OR chromium* OR molybdenum* OR zinc* OR manganese* OR selenium*):ti,ab,kw	132806
#39	MeSH descriptor: [Fish Oils] explode all trees	4909
#40	((fish NEXT oil*) OR "omega 3" OR docosahexaenoic* OR eicosapentaenoic*):ti,ab,kw	10808
#41	MeSH descriptor: [Carnitine] explode all trees	825
#42	MeSH descriptor: [Acetylcysteine] explode all trees	1487
#43	MeSH descriptor: [Ubiquinone] explode all trees	735
#44	MeSH descriptor: [Glutathione] explode all trees	874
#45	MeSH descriptor: [Arginine] explode all trees	1853
#46	MeSH descriptor: [Choline] explode all trees	1763
#47	(carnitine* OR acetylcarnitine* OR acetylcysteine* OR "acetyl-L-cysteine" OR ubiquinone OR q10 OR "coQ 10" OR glutathione* OR arginine* OR lycopene* OR (beta NEXT carotene*) OR betacarotene* OR choline*):ti,ab,kw	24430
#48	MeSH descriptor: [Sodium, Dietary] explode all trees	1028
#49	MeSH descriptor: [Phosphorus, Dietary] explode all trees	102
#50	MeSH descriptor: [Calcium, Dietary] explode all trees	1749
#51	{OR #4-#50}	219013
#52	MeSH descriptor: [Pregnancy] explode all trees	34005
#53	MeSH descriptor: [Pregnancy Rate] explode all trees	2414
#54	MeSH descriptor: [Semen Analysis] explode all trees	775
#55	((sperm OR semen) NEAR/1 (qualit* OR motilit* OR morpholog* OR count* OR concentrat* OR parameter* OR analys*)):ti,ab,kw	2602
#56	(pregnan* OR (live NEXT birth*)):ti,ab,kw	89887
#57	MeSH descriptor: [DNA Fragmentation] explode all trees	76
#58	(DNA NEXT fragment*):ti,ab,kw	533
#59	{OR #52-#58}	91929
#60	#3 AND #51 AND #59	467
#61	MeSH descriptor: [Animals] explode all trees	891396
#62	MeSH descriptor: [Humans] explode all trees	887732
#63	#61 NOT #62	3664
#64	#60 NOT #63	460
#65	(rat OR rats OR mouse OR mice):ti	957
#66	#64 NOT #65	458

Table S2. Calculated correlation coefficients for secondary analyses.

Intervention group		Placebo group	
**Author**	**Correlation coefficient**	**Author**	**Correlation coefficient**
**Sperm concentration**			
Nouri (2019)	0.49	Nouri (2019)	0.43
Bahmyari (2021)	0.58	Bahmyari (2021)	0.84
Eslamian (2020)	0.83	Eslamian (2020)	0.98
Gheflati (2021)	0.81	Gheflati (2021)	0.81
Steiner 2020)	0.83	Steiner (2020)	0.73
Cyrus (2015)	0.55	Cyrus (2015)	0.56
Weighted average	***0.75***	***Weighted average***	***0.73***
**Sperm count**			
Maghsoumi-Norouzabad (2022)	0.83	Maghsoumi-Norouzabad (2022)	0.86
Nouri (2019)	0.54	Nouri (2019)	0.55
Eslamian (2020)	0.78	Eslamian (2020)	0.95
Steiner (2020)	0.80	Steiner (2020)	0.72
Weighted average	***0.78***	***Weighted average***	***0.79***
**Total sperm motility**			
Maghsoumi-Norouzabad (2022)	0.75	Maghsoumi-Norouzabad (2022)	0.39
Nouri (2019)	0.43	Nouri (2019)	0.48
Bahmyari (2021)	0.52	Bahmyari (2021)	0.43
Eslamian (2020)	0.81	Eslamian (2020)	0.58
Steiner (2020)	0.55	Steiner (2020)	0.63
Weighted average	***0.68***	***Weighted average***	***0.53***
**Progressive sperm motility**			
Maghsoumi-Norouzabad (2022)	0.73	Maghsoumi-Norouzabad (2022)	0.80
Nouri (2019)	0.03	Nouri (2019)	0.52
Bahmyari (2021)	0.50	Bahmyari (2021)	0.47
Eslamian (2020)	0.74	Eslamian (2020)	0.73
Gheflati (2021)	0.48	Gheflati (2021)	0.48
Cyrus (2015)	0.21	Cyrus (2015)	0.40
Weighted average	***0.57***	***Weighted average***	***0.57***
**Normal sperm morphology**			
Maghsoumi-Norouzabad (2022)	0.91	Maghsoumi-Norouzabad (2022)	0.94
Nouri (2019)	0.69	Nouri (2019)	0.70
Bahmyari (2021)	0.43	Bahmyari (2021)	0.25
Eslamian (2020)	0.88	Eslamian (2020)	0.82
Gheflati (2021)	0.71	Gheflati (2021)	0.71
Cyrus (2015)	0.19	Cyrus (2015)	0.59
Weighted average	***0.71***	***Weighted average***	***0.67***
**DNA Fragmentation Index**			
Maghsoumi-Norouzabad (2022)	0.20	Maghsoumi-Norouzabad (2022)	0.28
Weighted average	***0.20***	***Weighted average***	***0.28***

Table S3. List of excluded studies and reason for exclusion.

Authors	***Title***	***Year***	***Population***	***Intervention***	***Comparator group***	***Outcome***	***Study design***	***Publication type***	***Not Latin alphabet***	***Other***	***Specified reason for exclusion***
Abel BJ, Carswell G, Elton R, Hargreave TB, Kyle K, Orr S, Rogers A, Baxby K, Yates A	Randomised trial of clomiphene citrate treatment and vitamin C for male infertility	1982			X						Vitamin E compared to clomiphene citrate; no placebo comparator group.
Ardestani Zadeh A, Arab D, Kia NS, Heshmati S, Amirkhalili SN.	The role of Vitamin E - Selenium - Folic Acid Supplementation in Improving Sperm Parameters After Varicocelectomy: A Randomized Clinical Trial.	2019			X						Identified from hand-searching of included articles. Not included as the study did not use placebo.
Banks N, Sun F, Krawetz SA, Coward RM, Masson P, Smith JF, Trussell JC, Santoro N, Zhang H, Steiner AZ.	Male vitamin D status and male factor infertility.	2021						X			Secondary analyses based on data from the article "The effects of antioxidants on male factor infertility: the Males, Antioxidants, and Infertility (MOXI) randomized clinical trial" by Steiner et al. (2020).
Busetto, G. M., Agarwal, A., Virmani, A., Del Giudice, F., Micic, S., Gentile, V., De Berardinis, E.	Pregnancy rate and infertility in patients with varicocele and/or oligoasthenoteratozoospermia: evaluation of antioxidant supplementation effect on sperm parameters	2018						X			Abstract based on data from the article "Effect of metabolic and antioxidant supplementation on sperm parameters in oligo-astheno-teratozoospermia, with and without varicocele: A double-blind placebo-controlled study" by Busetto et al. (2018)
Busetto, G. M., Del Giudice, F., Agarwal, A., Micic, S., Virmani, A., De Berardinis, E.	Body mass index and age correlate with antioxidants supplementation effect on sperm quality: a double blind place controlled trial on patients with varicocele & oligoasthenoteratozoospermia	2019						X			Abstract based on data from the article "Effect of metabolic and antioxidant supplementation on sperm parameters in oligo-astheno-teratozoospermia, with and without varicocele: A double-blind placebo-controlled study" by Busetto et al. (2018)
Busetto, G. M., Del Giudice, F., Sciarra, A., Maggi, M., Porreca, A., de Berardinis, E.	Body mass index and age correlate with antioxidant supplementation effects on sperm quality: Post-hoc analyses from a double-blind placebo-controlled trial	2020						X			Abstract based on data from the article "Effect of metabolic and antioxidant supplementation on sperm parameters in oligo-astheno-teratozoospermia, with and without varicocele: A double-blind placebo-controlled study" by Busetto et al. (2018)

Busetto, G. M., Del Giudice, F., Virmani, A., De Berardinis, E., Agarwal, A.	Body mass index and age correlate with antioxidant supplementation effects on sperm quality: post-hoc analyses from a double-blind placebo-controlled trial	2020						X			Abstract based on data from the article "Effect of metabolic and antioxidant supplementation on sperm parameters in oligo-astheno-teratozoospermia, with and without varicocele: A double-blind placebo-controlled study" by Busetto et al. (2018)
Busetto, G. M., Rodrigues, B. F., Virmani, A., Checchia, A., Ninivaggi, A., Ricapito, A., Barbieri, G., Fischetti, P., Falagario, U. G., Annese, P., d'Altilia, N., Mancini, V., Ferro, M., Crocetto, F., Porreca, A., Bettocchi, C., Cormio, L., Agarwal, A., Carrieri, G.	Antioxidant treatment for oligoasthenoteratozoospermia and varicocele: a DBPC trial to evaluate the impact of age and body mass index	2024						X			Abstract based on data from the article "Effect of metabolic and antioxidant supplementation on sperm parameters in oligo-astheno-teratozoospermia, with and without varicocele: A double-blind placebo-controlled study" by Busetto et al. (2018)
Busetto, G. M., Virmani, A., Agarwal, A., Antonini, G., Del Giudice, F., Micic, S., De Berardinis, E	Varicocele and oligoasthenoteratozoospermia: evaluation of antioxidant supplementation effect on pregnancy rate and sperm quality	2018						X			Abstract based on data from the article "Effect of metabolic and antioxidant supplementation on sperm parameters in oligo-astheno-teratozoospermia, with and without varicocele: A double-blind placebo-controlled study" by Busetto et al. (2018)
Busetto, G. M., Virmani, A., Antonini, G., Ragonesi, G., Del Giudice, F., Agarwal, A., Gentile, V.	Effect of antioxidant supplementation on sperm parameters in oligo-astheno-teratozoospermia, with and without varicocele: A double blind place controlled (DBPC) study	2017						X			Abstract based on data from the article "Effect of metabolic and antioxidant supplementation on sperm parameters in oligo-astheno-teratozoospermia, with and without varicocele: A double-blind placebo-controlled study" by Busetto et al. (2018)
Busetto, G. M., Virmani, A., Antonini, G., Ragonesi, G., Del Giudice, F., Gentile, V., De Berardinis, E.	Effect of antioxidant supplementation on sperm parameters in oligoastheno-teratozoospermia, with and without varicocele: a double blind place controlled (DBPC) study	2017						X			Abstract based on data from the article "Effect of metabolic and antioxidant supplementation on sperm parameters in oligo-astheno-teratozoospermia, with and without varicocele: A double-blind placebo-controlled study" by Busetto et al. (2018)
Busetto, G., Virmani, A., Del Giudice, F., Micic, S., Agarwal, A., De Berardinis, E.	Varicocele and oligoasthenoteratozoo-spermia: evaluation of antioxidant supplementation effect on pregnancy rate and sperm quality	2017						X			Abstract based on data from the article "Effect of metabolic and antioxidant supplementation on sperm parameters in oligo-astheno-teratozoospermia, with and without varicocele: A double-blind placebo-controlled study" by Busetto et al. (2018)

Busetto, G., Virmani, M. A., Antonini, G., Ragonesi, G., De Berardinis, E., Agarwal, A., Gentile, V.	Effect of antioxidant supplementation on sperm parameters in oligoasthenoteratozoospermia, with and without varicocele: a DBPC study	2016						X			Abstract based on data from the article "Effect of metabolic and antioxidant supplementation on sperm parameters in oligo-astheno-teratozoo-spermia, with and without varicocele: A double-blind placebo-controlled study" by Busetto et al. (2018)
Busetto, G., Virmani, M. A., Del Giudice, F., Micic, S., Agarwal, A., De Berardinis, E.	Body mass index & age correlate with antioxidants supplementation effect on sperm quality: a double-blind placebo controlled trial on patients with varicocele & oligoasthenoteratozoospermia	2018						X			Abstract based on data from the article "Effect of metabolic and antioxidant supplementation on sperm parameters in oligo-astheno-teratozoo-spermia, with and without varicocele: A double-blind placebo-controlled study" by Busetto et al. (2018)
Comhaire FH, El Garem Y, Mahmoud A, Eertmans F, Schoonjans F.	Combined conventional/antioxidant "Astaxanthin" treatment for male infertility: a double blind, randomized trial	2005								X	Antibiotics and tamoxifen were administered to some patients.
Ctri (https://www.cochranelibrary.com/es/central/doi/10.1002/central/CN-01798094/full)	A clinical study to evaluate efficacy and safety of â??TLPL/AY/04/2009â?? in comparison with placebo in patients suffering from Male Infertility	2012						X			Abstract. No article has been identified.
Ctri (https://www.cochranelibrary.com/central/doi/10.1002/central/CN-01973333/full)	A clinical trial to study the association between reactive oxygen species and semen quality and the effect of vitamin C therapy in male infertility patients	2019						X			Abstract. No article has been identified.
Dawson EB, Harris WA, Powell LC.	Relationship between ascorbic acid and male fertility	1990	X	X							Identified from hand-searching of included articles. Not included as the study had a treatment duration of one month. Further, it has not been stated whether participants were infertile.
De Rosa, M., Boggia, B., Amalfi, B., Zarrilli, S., Vita, A., Colao, A., Lombardi, G.	Correlation between seminal carnitine and functional spermatozoal characteristics in men with semen dysfunction of various origins	2005			X					X	Only a subgroup of patients were given L-carnitine and L-acetyl-carnitine (asthenzoospermic males without azoospermia). No comparator group.
Ebisch IM, Pierik FH, DE Jong FH, Thomas CM, Steegers-Theunissen RP.	Does folic acid and zinc sulphate intervention affect endocrine parameters and sperm characteristics in men?	2006						X			Analyses are based on data from the study "Effects of folic acid and zinc sulfate on male factor subfertility: a double-blind, randomized, placebo-controlled trial" by Wong et al. (2002)
Ebisch, I. M., van Heerde, W. L., Thomas, C. M., van der Put, N., Wong, W. Y., Steegers-Theunissen, R. P.	C677T methylenetetrahydrofolate reductase polymorphism interferes with the effects of folic acid and zinc sulfate on sperm concentration	2003						X			Analyses are based on data from the study "Effects of folic acid and zinc sulfate on male factor subfertility: a double-blind, randomized, placebo-controlled trial" by Wong et al. (2002)

Eslamian, G., Sadeghi, M. R., Rashidkhani, B., Amirjannati, N., Hekmatdoost, A.	The effects of combined docosahexaenoic acid and vitamin E supplementation on spermatogram in asthenozoospermic men	2012						X			Abstract based on data from the article ""Effects of coadministration of DHA and vitamin E on spermatogram, seminal oxidative stress, and sperm phospholipids in asthenozoospermic men: a randomized controlled trial" by Eslamian et al. (2012).
Euctr, D. K. (https://www.cochranelibrary.com/central/doi/10.1002/central/CN-01850938/full)	Vitamin D supplementation and male infertility: a randomized double blinded clinical trial	2011						X			Trial registry record based on the article "Effects of Vitamin D Supplementation on Semen Quality, Reproductive Hormones, and Live Birth Rate: A Randomized Clinical Trial" by Blomberg Jensen et al. (2018)
Exposito A, Perez-Sanz J, Crisol L, Aspichueta F, Quevedo S, Diaz-Nunez M, Mendoza R, Ruiz-Sanz JI, Martinez-Astorquiza T, Matorras R	A prospective double-blind randomized placebo-controlled study of the effect of vitamin E on semen parameters in infertile men	2016	X								Abstract. The article "Effect of vitamin E administered to men in infertile couples on sperm and assisted reproduction outcomes: a double-blind randomized study" by Matorras et al. (2020) with the same EudraCT registration number includes couples with female factor infertility.
Gamidov CI, Ovchinnikov RI, Popova AIu, Tkhagapsoeva RA, Izhbaev SKh.	[Current approach to therapy for male infertility in patients with varicocele]	2012							X		Article not written with the Latin alphabet
Gamidov SI, Ovchinnikov RI, Popova AY.	[Double-blind, randomized placebo-controlled study of efficiency and safety of complex acetyl-L-carnitine, L-carnitine fumarate and alpha-lipoic acid (Spermactin Forte) for treatment of male infertility].	2019							X		Article not written with the Latin alphabet
Hosseini J, Mardi Mamaghani A, Hosseinifar H, Sadighi Gilani MA, Dadkhah F, Sepidarkish M.	The influence of ginger (Zingiber officinale) on human sperm quality and DNA fragmentation: A double-blind randomized clinical trial	2016								X	Infertility drugs, testosterone and vitamins where used by some patients in addition to the given intervention/placebo.
Ianniello B, Gambardella V, Gianno F.	"Support Therapy with Integrators after Varicocelectomy."	2004			X						Comparator group received no treatment
Irct201010054010N (https://www.cochranelibrary.com/central/doi/10.1002/central/CN-01832287/full)	The effect of Omega-3 fatty acids plus vitamin E supplements on spermatogram and oxidative stress in infertile men with asthenozospermia	2010						X			Trial registry record based on the article "Effects of coadministration of DHA and vitamin E on spermatogram, seminal oxidative stress, and sperm phospholipids in asthenozoospermic men: a randomized controlled trial" by Eslamian et al. (2012)
Irct2015020120895N (https://www.cochranelibrary.com/central/doi/10.1002/central/CN-01811690/full)	Efficacy of phonix powder capsule on quality of sperm parameters of infertile men	2015						X		X	Abstract. All participants were given a standard medical treatment drug in addition to intervention or placebo.

Javadi M, Gholaminejad F, Haghighian HK, Karami AA, Alizadeh F.	Effect of propolis oral supplements on sperm parameters and oxidative stress indicator in idiopathic infertile men: A double-blind randomized clinical trial	2018							X		Article not written with the Latin alphabet
Jensen, M. B., Lawaetz, J. G., Andersson, A. M., Juul, A., Jorgensen, N.	Vitamin D and calcium as novel regulators of reproductive hormones and sex steroids in copenhagen bone gonadal study: a randomized clinical trial	2016						X			Abstract based on data from the article "Effects of Vitamin D Supplementation on Semen Quality, Reproductive Hormones, and Live Birth Rate: A Randomized Clinical Trial" by Blomberg Jensen et al. (2018)
Kumamoto Y, Maruta H, Ishigami J, Kamidono S, Orikasa S, Kimura M, Yamanaka H, Kurihara H, Koiso K, Okada K, et al.	[Clinical efficacy of mecobalamin in the treatment of oligozoospermia--results of double-blind comparative clinical study]	1988							X		Article not written with the Latin alphabet
Kuzmenko AV, Kuzmenko VV, Gyaurgiev TA.	[The effectiveness of the Speroton complex in the management of male factor infertility]	2018							X		Article not written with the Latin alphabet
Liang, M., Yang, H. L., Meng, F. W., Wang, X., Shi, B. K.	[Compound Amino Acid Capsule (8-11) combined with L-carnitine for the treatment of asthenospermia]	2023							X		Article not written with the Latin alphabet
Maghsoumi-Norouzabad L, Zare Javid A, Mansoori A, Dadfar M, Serajian A.	The effects of Vitamin D3 supplementation on Spermatogram and endocrine factors in asthenozoospermia infertile men: a randomized, triple blind, placebo-controlled clinical trial	2021								X	Same data published in "Vitamin D3 Supplementation Effects on Spermatogram and Oxidative Stress Biomarkers in Asthenozoospermia Infertile Men: a Randomized, Triple-Blind, Placebo-Controlled Clinical Trial" by Maghsoumi-Norouzabad et al. (2022)
Maghsoumi-Norouzabad, L., Zare Javid, A., Mansoori, A., Dadfar, M., Serajian, A.	Evaluation of the effect of vitamin D supplementation on spermatogram, seminal and serum levels of oxidative stress indices in asthenospermia infertile men: a study protocol for a triple-blind, randomized controlled trial	2021						X			Protocol for the study "Vitamin D3 Supplementation Effects on Spermatogram and Oxidative Stress Biomarkers in Asthenozoospermia Infertile Men: a Randomized, Triple-Blind, Placebo-Controlled Clinical Trial" by Maghsoumi-Norouzabad et al. (2022)
Mahdiani E, Highighian HK, Javadi M, Karami AA, Kavianpour M.	Effect of Carob (Ceratonia Siliqua L.) Oral supplementation on changes of semen parameters, oxidative stress, inflammatory biomarkers and reproductive hormones in infertil men	2018							X		Article not written with the Latin alphabet
Mancini, A., Balercia, G.	Coenzyme Q(10) in male infertility: physiopathology and therapy	2011					X				Review article.
Mathieu d'Argent E, Ravel C, Rousseau A, Morcel K, Massin N, Sussfeld J, Simon T, Antoine JM, Mandelbaume J, Daraï E, Kolanska K.	High-Dose Supplementation of Folic Acid in Infertile Men Improves IVF-ICSI Outcomes: A Randomized Controlled Trial (FOLFIV Trial)	2021	X								Study population includes male partners where the etiology of infertility is male factor or combined male and female factor.

Micic S, Lalic N, Djordjevic D, Bojanic N, Bogavac-Stanojevic N, Busetto GM, Virmani A, Agarwal A.	Double-blind, randomised, placebo-controlled trial on the effect of L-carnitine and L-acetyl-carnitine on sperm parameters in men with idiopathic oligoasthenozoospermia	2019	X								Study population includes males with female partners diagnosed with female infertility.
Micic, S., Lalaic, N., Djordjevic, D., Bojanic, N., Busetto, G. M., Virmani, A., Agarwal, A.	Sperm DNA fragmentation index (DFI) and alpha-glucosidase are good predictors for prognosis of sperm motility in oligoasthenozzospermic men, treated with carnitine and essential nutrients	2019						X			Abstract based on data from the article "Double-blind, randomized, placebo-controlled trial on the effect of L-carnitine and L-acetyl-carnitine on sperm parameters in men with idiopathic oligoasthenozoospermia" by Micic et al. (2019).
Micic, S., Lalic, N., Bojanic, N., Djordjevic, D., Virmani, A., Agarwal, A.	Assessment of sperm motility in oligoasthenospermic men, treated with metabolic and essential nutrients, in a randomized, double blind, placebo study	2016						X			Abstract based on data from the article "Double-blind, randomised, placebo-controlled trial on the effect of L-carnitine and L-acetyl-carnitine on sperm parameters in men with idiopathic oligoasthenozoospermia" by Micic et al. (2019).
Micic, S., Lalic, N., Bojanic, N., Djordjevic, D., Virmani, A., Agarwal, A.	DBPC study showed significant correlation of DNA fragmetation index (DFI) and seminal carnitine with progressive sperm motility in oligospermic men treated with metabolic and essential nutrients	2017						X			Abstract based on data from the article "Double-blind, randomized, placebo-controlled trial on the effect of L-carnitine and L-acetyl-carnitine on sperm parameters in men with idiopathic oligoasthenozoospermia" by Micic et al. (2019).
Micic, S., Lalic, N., Bojanic, N., Djordjevic, D., Virmani, A., Agarwal, A.	Oligoastenospermic men treated with proxeed plus showed correlation between sperm motility and seminal carnitine	2016						X			Abstract based on data from the article "Double-blind, randomized, placebo-controlled trial on the effect of L-carnitine and L-acetyl-carnitine on sperm parameters in men with idiopathic oligoasthenozoospermia" by Micic et al. (2019).
Micic, S., Lalic, N., Bojanic, N., Djordjevic, D., Virmani, A., Agarwal, A.	DBPC study in oligoasthenospermic men treated with metabolic and essential nutrients showed that progressive sperm motility was correlated to seminal carnitine levels	2016						X			Abstract based on data from the article "Double-blind, randomized, placebo-controlled trial on the effect of L-carnitine and L-acetyl-carnitine on sperm parameters in men with idiopathic oligoasthenozoospermia" by Micic et al. (2019).
Micic, S., Lalic, N., Djordjevic, D., Bogavac-Stanojevic, N., Virmani, A., Agarwal, A.	Seminal carnitine and DNA fragmentation index (DFI) impact progressive sperm motility in oligoasthenospermic men treated with metabolic and essential nutrients, with moderate accuracy	2017						X			Abstract based on data from the article "Double-blind, randomized, placebo-controlled trial on the effect of L-carnitine and L-acetyl-carnitine on sperm parameters in men with idiopathic oligoasthenozoospermia" by Micic et al. (2019)
Micic, S., Lalic, N., Djordjevic, D., Bojanic, N., Virmani, A., Agarwal, A.	Sperm vitality and DNA fragmentation index (DFI) are good predictors of progressive sperm motility in oligozooasthenospermic men treated with metabolic and essential nutrients	2018						X			Abstract based on data from the article "Double-blind, randomized, placebo-controlled trial on the effect of L-carnitine and L-acetyl-carnitine on sperm parameters in men with idiopathic oligoasthenozoospermia" by Micic et al. (2019)

Micic, S., Lalic, N., Djordjevic, D., Bojanic, N., Virmani, A., Busetto, G., Agarwal, A.	Carnitines and essential nutrients ameliorate sperm vitality and DNA fragmentation index which also predict improvement in progressive sperm motility	2018						X			Abstract based on data from the article "Double-blind, randomized, placebo-controlled trial on the effect of L-carnitine and L-acetyl-carnitine on sperm parameters in men with idiopathic oligoasthenozoospermia" by Micic et al. (2019).
Modarresi R, Aminsharifi A, Foroughinia F.	Impact of Spirulina Supplementation on Semen Parameters in Patients with Idiopathic Male Infertility: A Pilot Randomized Trial	2019								X	All participants were given conventional treatment including medical treatment with clomiphene in addition to intervention or placebo
Moilanen J, Hovatta O, Lindroth L.	Vitamin E levels in seminal plasma can be elevated by oral administration of vitamin E in infertile men	1993		X							Uncertainty as to whether the treatment duration and the time of evaluating follow-up for sperm parameters was 3 months. Author was contacted and was not able to provide additional information as data was no longer available.
Nadjarzadeh A, Shidfar F, Amirjannati N, Vafa MR, Motevalian SA, Gohari MR, Nazeri Kakhki SA, Akhondi MM, Sadeghi MR.	Effect of Coenzyme Q10 supplementation on antioxidant enzymes activity and oxidative stress of seminal plasma: a double-blind randomised clinical trial.	2014								X	Same data published in "Coenzyme Q10 improves seminal oxidative defense but does not affect on semen parameters in idiopathic oligoasthenoteratozoospermia: a randomized double-blind, placebo controlled trial" by Nadjarzadeh et al. (2011). The other publication was preferred as relevant data was reported at baseline and follow-up whereas the excluded article reported relevant outcomes as change from baseline.
Nasurullah F, Shams-Ul-Islam, Zaidi K, Hussain W, Haider M	Role of Coenzyme Q10 Supplementation on Semen Parameters in Infertile Men: A Quasi Experimental Study	2020			X						Ferrous sulphate was used as placebo
Nct. (https://www.cochranelibrary.com/central/doi/10.1002/central/CN-02004301/full)	Body Mass Index and Age Correlate With Antioxidant Supplementation Effects on Sperm Quality	2019						X			Trial registry record based on the article "Effect of metabolic and antioxidant supplementation on sperm parameters in oligo-astheno-teratozoospermia, with and without varicocele: A double-blind placebo-controlled study" by Busetto et al. (2018)
Nct. (https://www.cochranelibrary.com/central/doi/10.1002/central/CN-02053060/full)	Impact of A Nutritional Supplements' Combination (FERTILIS) on Male Infertility	2019	X								Trial registry record based on the article "Effect of Micronutrients and L-Carnitine as Antioxidant on Sperm Parameters, Genome Integrity, and ICSI Outcomes: Randomized, Double-Blind, and Placebo-Controlled Clinical Trial " by Lahimer et al. (2023). Study population includes males with female partners diagnosed with female infertility.

Nct. https://www.cochranelibrary.com/central/doi/10.1002/central/CN-02683070/full	Evaluation of Treatment With Coenzyme Q10 and L-Carnitine on Semen Parameters in Infertile Men With Idiopathic Oligoasthenoteratospermia	2024						X			Trial Registry record. No article has been identified.
Nct. https://www.cochranelibrary.com/central/doi/10.1002/central/CN-02681943/full	Study to Verify the Efficacy of a Product Containing 125 mg of TetraSOD® for the Treatment of Male Infertility	2024						X			Trial Registry record. No article has been identified.
Oumaima, G., Fadoua, B., Amira, D., Narjes, T., Arij, E., Henda, M., Mounir, A.	Sperm DNA integrity following a three months oral antioxidant supplementation: outcomes of a double blind, randomised clinical trial	2024						X			Abstract. No article has been identified.
Pactr. https://www.cochranelibrary.com/central/doi/10.1002/central/CN-02555050/full	The effect of zinc supplementation on semen parameters, serum and seminal zinc levels of oligozoospermic men attending University of Ilorin Teaching Hospital (UITH): a randomized double - blind trial	2022						X			Abstract. No article has been identified.
Raigani, M., Sadeghi, M. R., Akhondi, M. A., Amir Jannati, N., Soleimani Badia, M.	Impacts of MTHFR polymorphism on the effects of folic acid and zinc sulfate supplementations in OAT men	2010						X			Abstract, possibly based on data from the article "The micronutrient supplements, zinc sulphate and folic acid, did not ameliorate sperm functional parameters in oligoasthenoteratozoospermic men" by Raigani et al. (2014)
Raaia, Mohamed F.; Atyeah, Ahmed A.; Elkhiat, Yasser I.; Elenany, Hossam G..	Treatment of idiopathic asthenozoospermia, either isolated or oligoasthenozoospermia, with α-lipoic acid: a placebo-controlled, double-blind study.	2012					X				Identified from hand-searching of included articles. Not included as the study is not randomized.
Safarinejad MR, Safarinejad S, Shafiei N, Safarinejad S.	Effects of the reduced form of coenzyme Q10 (ubiquinol) on semen parameters in men with idiopathic infertility: a double-blind, placebo controlled, randomized study	2012								X	Journal has issued expression of concern
Safarinejad MR, Safarinejad S.	Efficacy of selenium and/or N-acetyl-cysteine for improving semen parameters in infertile men: a double-blind, placebo controlled, randomized study	2009								X	Journal has issued expression of concern
Safarinejad MR.	Efficacy of coenzyme Q10 on semen parameters, sperm function and reproductive hormones in infertile men	2009								X	Journal has issued expression of concern
Safarinejad, M. R.	Efficacy of selenium or N-acetyl-cysteine, separately or in combination in improving semen parameters in infertile men: A double blind, placebo-controlled, randomized study	2009						X			Abstract based on data from the article "Efficacy of Selenium and/or N-acetyl-Cysteine for Improving Semen Parameters in Infertile Men: A Double-Blind, Placebo Controlled, Randomized Study" By Safarinejad M.R. and Safarinejad S. (2009).

Safarinejad, M. R.	Efficacy of coenzyme Q10 supplementation on semen parameters, sperm function and reproductive hormone profiles in infertile men: A double blind, placebo-controlled, randomized study	2009						X			Abstract based on data from the article "Efficacy of Coenzyme Q10 on Semen Parameters, Sperm Function and Reproductive Hormones in Infertile Men" by Safarinejad M. R. (2009)
Schisterman EF, Sjaarda LA, Clemons T, Carrell DT, Perkins NJ, Johnstone E, Lamb D, Chaney K, Van Voorhis BJ, Ryan G, Summers K, Hotaling J, Robins J, Mills JL, Mendola P, Chen Z, DeVilbiss EA, Peterson CM, Mumford SL.	Effect of Folic Acid and Zinc Supplementation in Men on Semen Quality and Live Birth Among Couples Undergoing Infertility Treatment: A Randomized Clinical Trial.	2020	X								Male partners of women with female factor infertility were included.
Shukla, K. K., Mahdi, A. A., Mishra, V., Rajender, S., Sankhwar, S. N., Patel, D., Das, M.	Withania somnifera improves semen quality by combating oxidative stress and cell death and improving essential metal concentrations	2011			X	X					No comparator group, all groups received treatment. Not relevant outcomes.
Sigman M, Glass S, Campagnone J, Pryor JL.	Carnitine for the treatment of idiopathic asthenospermia: a randomized, double-blind, placebo-controlled trial	2006	X								The article includes males with infertility of at least six months.
Steiner, A., Hansen, K., Diamond, M. P., Coutifaris, C., Cedars, M., Legro, R., Usadi, R., Baker, V., Coward, R., Santoro, N., et al.	Antioxidants in the treatment of male factor infertility: results from the double blind. multi-center, randomised controlled males, antioxidants, and infertility	2018						X			Abstract based on data from the article "The effect of antioxidants on male factor infertility: the Males, Antioxidants, and Infertility (MOXI) randomized clinical trial" by Steiner et al. (2020)
Suleiman SA, Ali ME, Zaki ZM, el-Malik EM, Nasr MA.	Lipid peroxidation and human sperm motility: protective role of vitamin E.	1996		X							Time of use of intervention was defined as 6 months or until semen sample improve and partner obtained pregnancy; one participant obtaining pregnancy only used intervention for 2 months.
Tremellen K, Miari G, Froiland D, Thompson J.	A randomised control trial examining the effect of an antioxidant (Menevit) on pregnancy outcome during IVF-ICSI treatment	2007	X								Study population includes male partners where the etiology of infertility is male factor or combined male and female factor.
Török L.	Die Wirkung von Karnitin auf die Motilität der Spermien [The effect of carnitine on the motility of sperm]	1983		X							Duration of exposure is 8 weeks.
Vicari E, Rubino C, De Palma A, Longo G, Lauretta M, Consoli S, Arancio A.	[Antioxidant therapeutic efficiency after the use of carnitine in infertile patients with bacterial or non bacterial prostato-vesiculo-epididymitis]	2001			X						Placebo was not used.
Vinogradov IV, Gamidov SI, Gabliya MY, Zhukov OB, Ovchinnikov RI, Malinina OY, Popova AY, Chalyi ME, Bragina EE, Zhivulko AR.	[Docosahexaenoic acid in the treatment of idiopathic male infertility]	2019							X		Article not written with the Latin alphabet

Table S4. Author contacts and conservative decisions regarding data extraction for included studies.

Article	Author contact	Response/decision
Balercia (2005)	Author was contacted on June 5, 2024 (g.balercia@ao-umbertoprimo.marche.it). Due to an error in email, author was contacted again on June 6, 2024 and June 27, 2024 (g.balercia@univpm.it). Author was contacted to request information on the number of participants in each group at each reported time point. One participant dropped out of the study from the LC+LAC group but it was unclear whether this participant was included in the reported estimates.	Due to non-responsiveness, it was decided that n=14 was specified for the LC+LAC group at all time points.
Balercia (2009)	Author was contacted on June 5, 2024 (g.balercia@ao-umbertoprimo.marche.it). Due to an error in email, author was contacted again on June 6, 2024 and June 27, 2024 (g.balercia@univpm.it). Author was contacted to request information on the number of participants in each group at each reported time point. Two and three participants dropped out of the study from the intervention and placebo group, respectively. However, it was unclear whether these participants were included in the reported estimates.	Due to non-responsiveness, it was decided that n=28 and N=27 was specified at all time points for the intervention and placebo group, respectively.
Ciftci (2009)	Author was contacted on June 5, 2024 and on June 27, 2024 (halilciftci63@hotmail.com) to request baseline values for sperm parameters.	No response from author.
Cyrus (2015)	Author was contacted on June 5, 2024 (aikabir@yahoo.com) to request information on how lost to follow-up cases were substituted with matching new cases.	Author replied on June 5, 2024:<br>"We mean that, there were some other cases who were eligible but were not included in study. However, because of loss to follow up, such eligible cases were randomized to be substituted as 5 patients in intervention or 8 cases in control group. Matching here means that new enrolled cases were matched for age and sperm quality characteristics; not exactly match but considering an interval."
Gharakhani Bahar (2023)	Author was contacted on June 5, 2024 and on June 27, 2024 (zahramid2001@gmail.com) to request sperm parameters reported as means and standard deviations instead of dichotomized values.	No response from author.
Haje (2015)	Author contacted on June 5, 2024 and on June 27, 2024 (milathaji@yahoo.com) to request the number of pregnancies in each group.	No response from author.
Huang (2020)	Author contacted on June 5, 2024 and on June 27,2024 (jmzwfhl@163.com) to request information on the number of participants in each group and the possibility of obtaining aggregated baseline and follow-up effect estimates, so that the number of patients does not exceed the included 769 patients.	No response from author.
Kessopoulou (1995)	Author was contacted on June 5, 2024 and on June 27, 2024 (C.Barratt@dundee.ac.uk) to request follow-up values for sperm parameters after the first treatment phase in the cross-over study.	No response from author.
Moilanen (1993)	Author was contacted on June 6, 2024 (jarna.moilanen@gmail.com) to request information on the treatment duration, the time for which the sperm parameters were analyzed and to request baseline and follow-up values for sperm parameters.	Author replied on June 6, 2024 that the data was no longer available. As no information on the treatment duration could be obtained, the article was excluded.
Moslemi Mehni (2014)	Author contacted on June 5, 2024 and on June 27, 2024 (dr.ketabchi@gmail.com) to request baseline and follow-up values for sperm parameters, as reported values look like change-from-baseline.	No response from author.

Park (2016)	Author contacted June 5, 2024 and on June 27, 2024 (joon501@pusan.ac.kr) to request follow-up values for sperm parameters.	No response from author.
Pryor (1978)	Intention to contact author to request baseline and follow-up values for sperm parameters as well as the number of participants in each group.	Author not contacted, as the email (jpryor@andrology.co.uk) does not exist. Unable to obtain contact information on co-authors.
Saeed Alkumait (2020)	Author was contacted on June 5, 2024 and on June 27, 2024 (malkumait@yahoo.com) to request sperm parameters reported as means and standard deviations for baseline and follow-up instead of percentage improvement.	No response from author.
Safarinejad (2011a)	Author was contacted on June 5, 2024 (safarinejad@urologist.md). Due to an error in email, author was contacted again on June 6, 2024 and June 27, 2024 (info@safarinejad.com). Author was contacted to request information regarding the number of participants in each group at baseline and follow-up.	Due to non-responsiveness, it was decided that n=113 and n=114 were specified at all time points for the intervention and placebo group, respectively.
Safarinejad (2011b)	Author was contacted on June 5, 2024 (safarinejad@urologist.md). Due to an error in email, author was contacted again on June 6, 2024 and June 27, 2024 (info@safarinejad.com). Author was contacted to request information regarding the number of participants in each group at follow-up.	Due to non-responsiveness, it was decided that n=114 and n=116 were specified at each time point for the intervention and placebo group, respectively.
Scott (1998)	Intention to contact author to request the number of pregnancies in each intervention arm.	Author not contacted, as we were unable to obtain contact information.
Steiner (2020)	Author contacted on June 5, 2024 (anne.steiner@duke.edu; error in email) and on June 27, 2024 (heping.zhang@yale.edu) to request follow-up values for sperm parameters.	Author replied on June 27, 2024 and sent the requested data.
Stenqvist (2018)	Author was contacted on June 5, 2024 and on June 27, 2024 (giwercman@med.lu.se) to request information regarding the number of participants in each group at each reported time point, as two participants missed each visit. Further, the number of participants for which the pregnancy outcome values are based on was requested.	Due to non-responsiveness, it was decided that n=36 and n=39 were specified at all time points for the intervention and placebo group, respectively.

Table S5. Characteristics of included studies

Author (year) Country	*Study design*	*Blinding*	*Infertility diagnosis or criteria*	*Intervention (n allocated)*	*Comparator (n allocated)*	*Treatment duration*	*Outcome, pregnancy and live birth parameters*	*Outcome, sperm parameters*	*Conclusion*
Amini (2020) Iran	Parallel	Triple-blinded	Infertile men undergoing infertility treatment and with idiopathic impaired spermograms (low sperm count, mobility impairment or abnormal morphology). and serum vitamin D3 levels ≤30 ng/L	50,000 IU vitamin D3 once a week for 8 weeks, hereafter 50,000 IU vitamin D3 once monthly for the last 4 weeks. (n=35)	Placebo (oral paraffin) (n=37)	12 weeks		Sperm concentration (mil/mL)<br>Sperm count (mil/ejaculate)<br>Progressive motility (%)<br>Normal morphology (%)	Sperm parameters did not improve after vitamin D3 supplementation.
Azizollahi (2013) Iran	Factorial	Double-blinded	Infertile male patients with grade III varicocele and a planned varicocelectomy.<br>Patients underwent varicocelectomy and hereafter 6 months on intervention/placebo.	Intervention 1: 66 mg zinc sulphate per day (n=32)<br>Intervention 2: 5 mg folic acid per day (n=26)<br>Intervention 3: 66 mg zinc sulphate and 5 mg folic acid per day (n=29)	Placebo (n=25)	6 months (26 weeks)		Sperm concentration (mil/mL)<br>Total motility (%)<br>Progressive motility (%)<br>Normal morphology (%)	Folic acid significantly improved sperm concentration 3 and 6 months after varicocelectomy compared to baseline. No changes were seen in total motility, progressive motility, and normal morphology.<br>Zinc sulphate significantly improved morphology at 3 and 6 months compared to baseline. No changes were seen in concentration, total- and progressive motility.<br>Zinc sulphate and folic acid significantly improved sperm concentration, progressive motility, and normal morphology at 3 and 6 months compared to baseline. No improvement was seen in total motility.

Bahmyari (2021)<br>Iran	Parallel	Single-blinded	Idiopathic infertile males with oligo astheno-, terato- or oligo-asthenoteratozoospermia and a history of infertility of one year or more	Multiple substance dietary supplement: 200 ug selenium, 400 IU vitamin E and 5mg folic acid daily (n=35)	Placebo (n=35)	3 months (13 weeks)		Sperm concentration (mil/mL)<br>Total motility (%)<br>Progressive motility (%)<br>Normal morphology (%)	Sperm parameters did not improve after three months of selenium, vitamin E and folic acid supplementation compared to baseline and placebo.
Balercia (2005)<br>Italy	Factorial	Double-blinded	Infertile men with idiopathic asthenozoospermia with a history of infertility of 2 years or more and having a fertile female partner.	Intervention 1: 3 g L-carnitine per day (n=15)<br>Intervention 2: 3 g L-acetyl-carnitine per day (n=15)<br>Intervention 3: 2 g L-carnitine and 1 g L-acetyl-carnitine per day (n=15)	Placebo (n=15)	6 months (26 weeks)	Spontaneous pregnancies, undefined[1]	Sperm concentration (mil/mL)<br>Total motility (%)<br>Progressive motility (%)	L-acetyl-carnitine alone or in combination with L-carnitine significantly improved total and progressive motility after 6 months of treatment compared to baseline. Sperm concentration significantly improved after 6 months administration of carnitines alone or in combination. Overall L-carnitine and L-acetyl-carnitine improved sperm parameters in infertile asthenozoospermic men.
Balercia (2009)<br>Italy	Parallel	Double-blinded	Infertile men with idiopathic asthenozoospermia with a history of infertility of 2 years or more and having a fertile female partner.	200 mg coenzyme Q10, 2 softgels each containing 100 mg coenzyme Q10 per day (n=30)	Placebo (n=30)	6 months (26 weeks)	Spontaneous pregnancies, undefined[1]	Sperm concentration (mil/mL)<br>Total motility (%)<br>Progressive motility (%)	A statistically significant improvement in total and progressive motility was seen in the intervention group compared to baseline and placebo. No improvement was seen in sperm concentration.
Blomberg Jensen (2018)<br>Denmark	Parallel	Triple-blinded	Infertile men with impaired semen quality and vitamin D insufficiency (25ODH levels ≤ 50 nmol/L).	Vitamin D, an initial single dose of 300,000 IU orally, hereafter 1400 IU vitamin D + 500 mg calcium daily (n=164)	An initial single dose of oil orally, hereafter placebo tablets daily (n=166)	150 days (22 weeks)	Clinical pregnancy<br><br>Live birth rate	Sperm count (mil/ejaculate)<br>Sperm concentration (mil/mL)<br>Total motility (%)<br>Progressive motility (%)<br>Normal morphology (%)<br>DNA Fragmentation Index (%)[2]	Vitamin D supplementation did not improve semen quality in infertile men with vitamin D insufficiency. Pregnancy and live birth rate did not improve compared to the placebo group.

Boonyarangkul (2015) Thailand	Factorial	Double-blinded	Infertile men with at least one abnormal sperm parameter (oligo-astheno-teratozoo-spermia) with a history of infertility for one year of more	Intervention 1: Tamoxifen citrate 20 mg per day (n=15)[3] Intervention 2: Folate 5 mg per day (n=15) Intervention 3: Tamoxifen citrate 20 mg and folate 5 mg per day (n=15)[3]	Placebo (n=15)	3 months (13 weeks)		Sperm concentration (mil/mL) Total motility (%) Normal morphology (%)	Sperm motility significantly improved in infertile men after folate supplementation. No changes were seen in sperm concentration and normal morphology after treatment.
Busetto (2018) Italy	Parallel	Double-blinded	Men with oligo- astheno- and/or teratozoo-spermia with or without varicocele having a history of infertility for more than 12 months and a fertile female partner.	Multiple substance dietary supplement: Proxeed Plus, two sachets per day with each sachet consisting of 1,000 mg L-carnitine, 725 mg fumarate, 500 mg acetyl-L-carnitine, 1,000 mg fructose 20 mg CoQ10, 90 mg vitamin C, 10 mg zinc, 200 ug folic acid and 1.5 ug vitamin B12 (n=52)	Placebo containing excipients: sucrose, silica, lemon flavor, acesulfame K (E950) sweetener (n=52)	6 months (26 weeks)	Pregnancies, undefined	Sperm concentration (mil/mL Sperm count (mil/ejaculate) Total motility (%) Progressive motility (%) Normal morphology (%)	Sperm concentration, count, total-, and progressive motility significantly improved after 6 months of supplementation compared to baseline and placebo. Normal morphology significantly improved compared to baseline. For patients with varicocele, sperm concentration, progressive- and total motility significantly improved compared to baseline, with sperm count significantly improved compared to both baseline and placebo group. For non-varicocele patients, concentration, count and progressive motility improved significantly compared to baseline, and total motility significantly improved compared to both baseline and placebo group. Ten pregnancies occurred in the intervention group and two in the placebo group.
Calogero (2015) Italy	Parallel	Double-blinded	Idiopathic infertile men	Inofolic, sachet containing 2 g of myo-inositol and 200 ug folic acid (per day 4 g myo-	Placebo (400 ug folic acid per day) (n=96)	3 months (13 weeks)		Sperm concentration (mil/mL) Sperm count (mil/ejaculate)	Myoinositol significantly improved sperm concentration, sperm count and progressive motility compared to placebo.

				inositol and 400 ug folic acid) (n=98)				Progressive motility (%)	
Cavallini (2004) Italy	Parallel	Double-blinded	Infertile oligoasthe-noteratozoospermic men with or without varicocele with a history of infertility of 12 months or more having a female partner with no identifiable cause of infertility.	Intervention 1: L-carnitine 2 g + L-acetyl-carnitine 1 g per day + glycerine suppositories every 4 days (n=101)<br>Intervention 2: L-carnitine 2 g + L-carnitine 1 g per day + 30 mg cinnoxicam suppository every 4 days (n=106)[3]	Placebo (starch tablets and glycerine suppositories) (n=116)	6 months (26 weeks)	Spontaneous pregnancy, undefined[1]	Sperm concentration (mil/mL)<br>Normal morphology (%)	Carnitine supplementation improved sperm parameters in infertile men with idiopathic infertility and infertile men with grade I, II and III varicocele following varicocelectomy, but not in grade IV and V.<br>Overall a significant difference was seen in the carnitine supplementation group compared to baseline.
Ciftci (2009) Turkey	Parallel	Single-blinded	Idiopathic male infertility with normal sperm parameters	N-acetylcysteine, 600 mg per day (n=60)	Placebo, pills containing sugar (n=60)	3 months (12 weeks)		Sperm concentration (mil/mL)<br>Total motility (%)<br>Normal morphology (%)	Total motility significantly improved compared to the placebo group. No significant differences were seen in normal morphology and sperm concentration between the two groups.
Conquer (2000) Canada	Parallel	Double-blinded	Asthenozoospermic men who were patients of the Reproductive Endocrinology and Infertility Program	Intervention 1: Docosahexaenoic acid 400 mg per day (n=9)<br>Intervention 2: Docosahexaenoic acid 800 mg per day (n=10)	Placebo (corn oil/soy oil) (n=9)	3 months (13 weeks)		Sperm concentration (mil/mL)<br>Total motility (%)	Docosahexaenoic acid did not improve sperm concentration or total motility in asthenozoospermic men compared to baseline and the placebo group.
Cyrus (2015) Iran	Parallel	Double-blinded	Infertile males with palpable varicocele grade II and III, oligo- astheno- or teratozoospermia, who underwent varicocelectomy and received intervention/placebo hereafter.	500 mg vitamin C per day (n=46)	Placebo (n=69)	3 months (13 weeks)		Sperm concentration (mil/mL)<br>Progressive motility (%)<br>Normal morphology (%)	Vitamin C significantly improved progressive motility and normal morphology after varicocelectomy compared to placebo. No improvement was seen in terms of sperm concentration.

da Silva (2013)<br>Brazil	Parallel	Double-blinded	Infertile men with oligo-, astheno-, terato-, or oligoasthenoteratozoospermia with a history of infertility for one year or more	5mg folic acid per day (n=34)	Placebo (n=36)	3 months (13 weeks)		Sperm concentration (mil/mL)<br>Progressive motility (%)<br>Normal morphology (%)	Folic acid did not improve sperm concentration, progressive motility or normal morphology in infertile men compared to placebo.
Dadgar (2023)<br>Iran	Factorial	Double-blinded	Infertile men with idiopathic oligoasthenozoospermia, asthenozoospermia, asthenoteratozoospermia or oligoasthenoteratozoospermia with a history of infertility for one year or more	Intervention 1: pentoxyfylline 800 mg per day (administration two times per day) (n=35)[3]<br>Intervention 2: Zinc 15 mg per day (administration one time per day) (n=35)<br>Intervention 3: 800 mg pentoxyfylline and 15 mg zinc per day (administration of pentoxyfylline twice daily, zinc once daily) (n=35)[3]	Placebo, administered twice daily. (n=35)	3 months (13 weeks)		Sperm count (mil/ejaculate)<br>Progressive motility (%)<br>Normal morphology (%)<br>DNA fragmentation (%)	Sperm count, morphology, progressive motility and DNA fragmentation significantly improved after zinc supplementation in idiopathic infertile men compared to baseline and placebo.
Eslamian (2020)<br>Iran	Factorial	Double-blinded	Idiopathic asthenozoospermic males with a history of infertility for one year or more.	Intervention 1: 465 mg Docosahexaenoic acid + 600 IU vitamin E (n=45)<br>Intervention 2: 465 mg Docosahexaenoic acid + placebo (n=45)<br>Intervention 3: 600 IU vitamin E + placebo (n=45)	Placebo shaped similarly to Docosahexaenoic acid or vitamin E capsules containing medium-chain triglycerides (n=45)	12 weeks		Sperm concentration (mil/mL)<br>Sperm count (mil/ejaculate)<br>Total motility (%)<br>Progressive motility (%)<br>Normal morphology (%)	Sperm concentration, count, total and progressive motility improved in infertile idiopathic asthenozoospermic men after 12 weeks of Docosahexaenoic acid and vitamin E supplementation either alone or in combination. No significant changes occurred in terms of normal morphology.
Gharakhani Bahar (2023)<br>Iran	Parallel	Double-blinded	Men with primary infertility and abnormality of ≥1 semen parameters.	Coenzyme Q10, one capsule of 100 mg per day (n=30)	Placebo, 100 mg of lactose per day (n=30)	12 weeks		Sperm count per cc (million)<br>Progressive motility (%)<br>Normal morphology (%)	A statistically significant higher chance of a normal morphology of ≥4% was seen after CoQ10 supplementation compared to placebo. No differences were seen in terms of sperm count and progressive motility.

Gheflati (2021)<br>Iran	Parallel	Double-blinded	Infertile asthenozoospermic men with vitamin D3 levels < 30 ng/mL	Vitamin D3; Nine pearls containing 50,000 IU vitamin D3 once every 8 weeks, and one pearl in the third month for maintaining the dose. (n=22)	Placebo (n=22)	12 weeks		Sperm concentration (mil/mL)<br>Progressive motility (%)<br>Normal morphology (%)	Vitamin D supplement did not improve semen parameters in vitamin D-deficient infertile men with asthenozoospermia
Gopinath (2013)<br>India	Parallel	Double-blinded	Idiopathic infertile males with oligoasthenozoospermia	Multiple substance dietary supplements<br>Intervention 1: 2 tablets of fixed dose combination of antioxidants two times daily (Coenzyme Q10 200 mg, L-carnitine 2000 mg, lycopene 10 mg, zinc 60 mg daily)<br>(n=46)<br>Intervention 2: 1 tablet of fixed dose combination of antioxidants (Coenzyme Q10 100 mg, L-carnitine 1000 mg, lycopene 5 mg, zinc 30 mg daily) + placebo two times daily (n=46)	2x Placebo two times daily (n=46)	180 days (26 weeks)	Spontaneous pregnancies, undefined[1]	Sperm concentration (mil/mL)<br>Total motility (%)	Antioxidant supplementation significantly improved sperm concentration and total motility in idiopathic infertile men with oligoasthenozoospermia. No intergroup differences were seen between the two intervention groups.
Haghighian (2015)<br>Iran	Parallel	Triple-blinded	Infertile men with idiopathic asthenozoospermia, a history of infertility of 2 years or more and having a fertile female partner.	600 mg alpha-lipoic acid daily (n=24)	Placebo (n=24)	12 weeks		Sperm concentration (mil/mL)<br>Total sperm count (mil/ejaculate)<br>Total motility (%)<br>Progressive motility (%)<br>Normal morphology (%)	12 weeks of alpha-lipoic acid supplementation significantly improved sperm concentration, sperm count, and total and progressive motility compared to baseline and placebo. Morphology did not significantly improve.

Haje (2015)<br>Iraq	Factorial	Unclear	Infertile men with idiopathic oligoasthenozoospermia.	Intervention 1: Tamoxifen 20 mg per day (n=45)[3]<br>Intervention 2: L-carnitine 1000 mg per day (n=20)<br>Intervention 3: Tamoxifen 20 mg and L-carnitine 1000 mg per day (n=34)[3]	Placebo (n=29)	3 to 6 months (13-26 weeks)[4]	Pregnancy rate after ICSI, undefined[5]	Sperm concentration (mil/mL)<br>Total motility (%)<br>Normal morphology (%)	L-carnitine did not improve sperm parameters or pregnancy rate in infertile oligoasthenozoospermic men.
Hodeeb (2022)<br>Egypt	Parallel	Triple-blinded	Infertile men with idiopathic asthenozoospermia	600 mg alpha-lipoic acid daily (n=40)	Placebo (n=40)	90 days (13 weeks)		Sperm concentration (mil/mL)<br>Total motility (%)<br>Progressive motility (%)<br>Normal morphology (%)	Sperm concentration, total and progressive motility improved after alpha-lipoic acid supplementation in infertile asthenozoospermic men compared to baseline values and placebo group, respectively. No change was seen in sperm morphology.
Huang (2020)<br>China	Parallel	Double-blinded	Infertile oligozoospermic men with a history of infertility for at least one year and having a female partner with no female factors of infertility.	0.8 mg folic acid per day (n = unclear)	Placebo, starch-filled capsules (n= unclear)	3 months (13 weeks)	Spontaneous pregnancy, undefined[1]<br>Live birth rate[6]	Sperm concentration (mil/mL)<br>Sperm count (mil/ejaculate)<br>Normal morphology (%)<br>Progressive motility (%)<br>DNA fragmentation (%)	Semen parameters significantly improved in infertile men with MTHFR 677 TT genotype compared to placebo. Further, a significantly higher chance of live birth was seen within the same genotype compared to placebo. No improvements were seen in the other genotypes.
Kessopoulou (1995)<br>United Kingdom	Cross-over	Double-blinded	Men attending infertility clinic for semen analysis as part of infertility investigations, with high levels of reactive oxygen species in semen. Ovulating female partner with tubal patency.	Vitamin E (600 mg alpha-tocopheryl acetate per day) (n=15)	Placebo (n=15)	3 months (12 weeks)	Pregnancy rate, undefined<br><br>Live birth rate	Sperm concentration (mil/mL)<br>Total motility (%)	Semen parameters did not improve after vitamin E supplementation. The same number of pregnancies and live births were achieved in both groups (1/15 intervention group, 1/15 placebo group) during the first treatment period.

Kopets (2020) Ukraine	Parallel	Double-blinded	Idiopathic infertile men with oligo-, astheno- and/or teratozoospermia with a history of infertility or 12 months or more and having a female partner with no pathological cause of infertility.	Multiple substance dietary supplement: One daily dose of L-carnitine/L-acetyl-carnitine 1990 mg, L-arginine 250 mg, glutathione 100 mg, coenzyme Q10 40 mg, zinc 7.5 mg, vitamin B9 234 ug, vitamin B12 2 ug, selenium 50 ug (n=42)	Placebo (n=41)	6 months (Sperm parameters were analyzed after 4 months follow-up (18 weeks))	Pregnancy rate (confirmed with ultrasound)	Sperm concentration (mil/mL)<br>Progressive motility (%)<br>Normal morphology (%)	Sperm concentration, progressive motility and normal morphology significantly improved after multi-component nutrient dietary supplement use compared to the placebo group.<br>A significantly higher pregnancy rate was seen in the intervention group.
Kumalic (2020) Slovenia	Parallel	Double-blinded	Infertile men with oligo-, and/or astheno- and/or teratozoospermia. At least 12 months of infertility	Astasxanthin, 16 mg per day (n=40)	Placebo (n=40)	3 months (13 weeks)		Sperm concentration (mil/mL)<br>Sperm count (mil/ejaculate)<br>Total motility (%)<br>Progressive motility (%)<br>Normal morphology (%)<br>DNA fragmentation (%)	Semen parameters did not improve in infertile men with oligo-, and/or astheno-, and/or teratozoospermia after astaxanthin supplementation.
Kumar (2011) India	Parallel	Triple-blinded	Infertile men with idiopathic oligo-, astheno-, terato-, or oligoasthenoteratozoospermia	Multiple substance dietary supplement: Addyzoa®, two capsules per day (n=25)	Placebo (n=25)	3 months (13 weeks)		Sperm concentration (mil/mL)<br>Total motility (%)<br>Progressive motility (%)<br>Normal morphology (%)<br>DNA Fragmentation Index (%)	Total and progressive motility were significantly improved after three months of Addyzoa® treatment. No changes were observed in sperm concentration, normal morphology and DNA Fragmentation Index.
Lenzi (2004) Italy	Parallel	Double-blinded	Infertile oligoasthenoteratozoospermic men with a history of infertility >2 years and a fertile female partner.	L-carnitine 2 g and L-acetyl-carnitine 1 g per day (n=30)	Placebo (n=30)	6 months (26 weeks)	Spontaneous pregnancy, undefined[1]	Sperm concentration (mil/mL)<br>Total motility (%)<br>Progressive motility (%)	Supplementation with L-carnitine and L-acetyl-carnitine significantly improved total and progressive motility in infertile men with OAT compared to placebo. No improvement was

									observed in terms of sperm concentration.
Lu (2018) China	Parallel	Double-blinded	Infertile males with left-sided clinical varicocele and mild oligozoospermia scheduled for varicocelectomy. Males underwent varicocelectomy and received intervention/placebo hereafter.	400 mg melatonin per day (n=27)	Placebo (n=27)	3 months (13 weeks)		Sperm concentration (mil/mL)<br>Total motility (%)<br>Normal morphology (%)	Melatonin significantly improved post-operative sperm parameters (sperm concentration, total motility and normal morphology) in infertile men who had undergone varicocelectomy compared to the placebo group.
Maghsoumi-Norouzabad (2022) Iran	Parallel	Triple-blinded	Infertile asthenozoospermic men with vitamin D3 levels < 30 ng/mL, a history of infertility of minimum 1 year and having a fertile female partner.	4000 IU vitamin D3 (cholecalciferol) (n=43)	Placebo (n=43)	12 weeks		Sperm count (mil/ejaculate)<br>Total motility (%)<br>Progressive motility (%)<br>Normal morphology (%)<br>DNA Fragmentation Index (%)	Vitamin D3 supplementation did not significantly improve sperm count, normal sperm morphology and DFI. A significant increase was seen in total sperm motility and progressive sperm motility compared to baseline and placebo.

Maretti (2017) Italy	Parallel	Double-blinded	Infertile men with idiopathic oligoasthenoteratozoospermia	Flortec (Bracco; probiotic+prebiotic) once sachet per day containing Arabinogalactan 1243 mg, fructose 500 mg, fructo-oligosaccharides 700 mg, L-glutamine 500 mg, raspberry flavoring, non-soluble alimentary fiber 500 mg, Lactobacillus paracasei 86 B21060 <5 9 109 colony forming units (CFUs), silicium dioxide, vanilla flavoring, black carrot amphocianines, Carthamus extract, citric acid, and sucralose(n=22)	Placebo (n=24)	6 months (26 weeks)		Sperm concentration (mil/mL)<br>Sperm count (mil/ejaculate)<br>Progressive motility (%)<br>Normal morphology (%)	Flortec significantly improved sperm concentration, count, progressive motility, and normal morphology in infertile men with idiopathic OAT compared to baseline and placebo.
Moslemi Mehni (2014) Iran	Parallel	Double-blinded	Infertile men with idiopathic oligoasthenoteratozoospermia	Intervention 1: Pentoxifylline 400 mg + 500 mg L-carnitine twice daily (n=58)[3]<br>Intervention 2: Pentoxifylline 400 mg + placebo twice daily (n=59)[3]<br>Intervention 3: 500 mg L-carnitine + placebo twice daily (n=59)	Placebo + placebo twice daily (n=59)	3 months (13 weeks)		Sperm count (mil/ejaculate)<br>Total motility (%)<br>Normal morphology (%)	Sperm count and total motility improved after L-carnitine supplementation in infertile men with idiopathic OAT compared to baseline. No statistically significant improvement was seen in terms of normal morphology.
Nadjarzadeh (2011) Iran	Parallel	Double-blinded	Infertile men with idiopathic oligoasthenoteratozoospermia	200 mg Coenzyme Q10 per day (n=23)	Placebo (n=24)	12 weeks		Sperm concentration (mil/mL)<br>Total motility (%)<br>Progressive motility (%)<br>Normal morphology (%)	Coenzyme Q10 did not significantly improve sperm concentration, total and progressive motility or normal morphology.

Nouri (2019)<br>Iran	Parallel	Double-blinded	Infertile men with oligozoospermia with primary or secondary infertility<br>for at least 5 years.	25 mg lycopene once a day (n=22)	Placebo (n=22)	12 weeks		Sperm concentration (mil/mL)<br>Sperm count (mil/ejaculate)<br>Total motility (%)<br>Progressive motility (%)<br>Normal morphology (%)	Sperm count and sperm concentration improved in infertile men following lycopene supplementation compared to baseline and placebo group. Total motility improved in both groups compared to baseline but not between groups. No changes were observed in terms of progressive motility and normal morphology.
Park (2016)<br>South Korea	Parallel	Double-blinded	Infertile men with varicocele	Non-varicocelectomy + KRG group: 1500 mg Korean Red ginseng per day (n=20)<br><br>Varicocelectomy + KRG group: 1500 mg Korean Red ginseng per day (n=20)	Non-varicocelectomy + placebo (n=20)<br><br>Varicocelectomy + placebo (n=20)	12 weeks		Sperm concentration (mil/mL)<br>Total motility (%)<br>Normal morphology (%)	All groups except the non-varicocelectomy + placebo group significantly improved sperm concentration, total motility and normal morphology compared to baseline.<br>The non-varicocelectomy+ KRG group, varicocelectomy+ KRG group and varicocelectomy group significantly improved sperm parameters compared to the non-varicocelectomy + placebo group.

Patki (2023) India	Parallel	Double-blinded	Infertile men with oligo-asthenoteratozoo-spermia with a healthy female partner	Multiple substance dietary supplement: Antioxidant blend (50 mg coenzyme Q10, 50 mg L-carnitine, 40 mg vitamin C, 10 mg vitamin E, 10 mg ginseng extract, 10 mg L-arginine, 20.588 mg zinc sulphate, 15.21 mg ferrous fumarate, 2.5 mg L-glutathione, 2 mg vitamin B6, 6.152 mg manganese sulphate, 2 mg lycopene, 1.4 mg vitamin B1, 2.795 mg cupric sulphate, 1250 IU vitamin A, 100 ug folic acid, 95.716 ug sodium selenate, 400 IU vitamin D, 1 ug vitamin B12) (n=150)	Placebo (n=150)	3 months (13 weeks)		Sperm concentration (mil/mL)<br>Total motility (%)<br>Normal morphology (%)<br>DNA fragmentation index (%)[2]	Total motility significantly improved in both groups compared to their respective baseline. Normal morphology significantly improved in the intervention group compared to baseline and the placebo group. No significant changes were seen in terms of sperm concentration.
Pryor (1978) United Kingdom	Cross-over	Double-blinded	Infertile men with total motile sperm count < 10 million/ejaculate	4 g arginine per day (n=35)	Placebo (n=29)	12 weeks	Pregnancy, undefined	Sperm concentration (mil/mL)<br>Sperm motility (%)	Arginine did not have an effect on sperm parameters or pregnancy rates.
Raigani (2014) Iran	Factorial	Double-blinded	Infertile oligoasthe-noteratozoospermic men from infertile couples referred to infertility clinic due to proven male factor infertility.	Intervention 1: Folic acid 5 mg per day + placebo (n=20)<br>Intervention 2: Folic acid 5 mg + zinc sulphate 220 mg per day (n=21)<br>Intervention 3: Zinc sulphate 220 mg per day + placebo (n=24)	Placebo + placebo (n=18)	16 weeks		Sperm concentration (mil/mL)<br>Total motility (%)	Supplementation with folic acid and zinc sulphate alone or in combination did not have an effect on sperm concentration and total motility in infertile men with oligoasthenoteratozoo-spermia.

Sabeti (2021) Iran	Parallel	Double-blinded	Infertile men with asthenoteratozoospermia	400 IU vitamin E and 200 ug selenium per day (n=30)	Placebo (n=30)	3 months (13 weeks)		Sperm concentration (mil/mL) Total motility (%) Normal morphology (%)	Total motility significantly improved in infertile asthenoteratozoospermic men after vitamin E and selenium supplementation compared to baseline. No changes were seen in terms of sperm concentration and normal morphology.
Saeed Alkumait (2020) Iraq	Parallel	Unclear	Infertile men with idiopathic oligoasthenoteratozoospermia having a fertile female	Intervention 1: glutathione, 250 mg, frequency not reported (n=51) Intervention2: Coenzyme Q10, 200 mg, frequency not reported (n=50)	Placebo, sugar sachets (n=50)	6 months (26 weeks)		% of progressive motility improvement % of normal morphology improvement % of concentration improvement	Both glutathione and Coenzyme Q10 significantly improved progressive motility, normal morphology and sperm concentration compared to placebo.
Safarinejad (2011a) Iran	Parallel	Double-blinded	Infertile men with primary infertility and idiopathic oligoasthenoteratozoospermia with a history of infertility of 2 years or more having a female partner diagnosed as normal.	Omega-3 fatty acids (Eicosapentaenoic acid 1.12 g per day, Docosahexaenoic acid 0.72 g per day) (n=119)	Placebo (corn oil and 1% fish oil) (n=119)	32 weeks		Sperm count (mil/ejaculate) Sperm concentration (mil/mL) Total motility (%) Normal morphology (%)	Sperm count, concentration, total motility and normal morphology significantly improved in infertile oligoasthenoteratozoospermic men after omega-3 fatty acid supplementation compared to baseline. No improvement was seen in the placebo group.
Safarinejad (2011b) Iran	Parallel	Double-blinded	Oligoasthenoteratozoospermic men with primary infertility for 2 years or more and a normal fertile female partner.	Saffron, 60mg per day (n=130)	Placebo (starch) (n=130)	26 weeks		Sperm concentration (mil/mL) Sperm count (mil/ejaculate) Total motility (%) Normal morphology (%)	Sperm parameters did not improve in infertile men after saffron supplementation compared to the placebo group.

Scott (1998) United Kingdom	Parallel	Double-blinded	Infertile men with reduced motility	Intervention 1: selenium 100 ug pr day (n=17)<br>Intervention 2: Multiple substance dietary supplement - selenium 100 ug, vitamin A 1 mg, vitamin C 10 mg and vitamin E 15 mg (n=34)	Placebo (n=18)	3 months (13 weeks)	Pregnancy, undefined	Sperm concentration (mil/mL)<br>Total motility (%)	No significant differences in sperm concentration and total motility were seen between the three groups. When the two interventions were combined, total motility was significantly improved after intervention compared to the placebo group. A total of five pregnancies were reported in the two intervention groups combined, whereas no pregnancies were reported in the placebo group.
Shahmirzadi (2024) Iran	Parallel	Double-blinded	Idiopathic infertile men with oligo-, astheno- or teratozoospermia.	6 g M. sativa seed powder per day and 100 IU vitamin E per day (n=30)	Placebo and 100 IU vitamin E per day (n=30)	90 days (13 weeks)	Pregnancy rate, undefined	Sperm count (mil/ejaculate)<br>Total motility (%)<br>Normal morphology (%)	Sperm count, total motility and normal morphology significantly increased compared to baseline and comparator group after 3 months of M. sativa supplementation as add-on to vitamin E. More pregnancies occurred in the intervention group.
Sharifzadeh (2022) Iran	Parallel	Double-blinded	infertile men with idiopathic oligozoospermia	30mL 0,5% zinc solution daily (n=61)	Placebo, syrup (n=53)	12 weeks		Sperm concentration (mil/mL)<br>Total motility (%)<br>Normal morphology (%)	Sperm concentration and normal morphology significantly improved after zinc supplementation in idiopathic oligozoospermic infertile men compared to baseline. No improvement were observed in motility.
Steiner (2020) USA	Parallel	Double-blinded	Infertile males with oligo-, astheno-, teratozoospermia or DNA fragmentation ≥25% in a heterosexual couple with a history of infertility of 12 months or more, and ovulatory female partners with	Multiple substance dietary supplement: Antioxidant formulation containing a daily dose of 500 mg Vitamin C (ascorbic acid), 400 mg vitamin E (d-alpha tocopheryl) 0.20 mg selenium (L-	Placebo (n=86)	At least 3 months, up to 6 months. Semen samples were provided at baseline and after 90 days (13 weeks)	Pregnancy (positive home pregnancy test)<br><br>Clinical pregnancy<br><br>Live birth (delivery of live infant	Sperm concentration (mil/mL)<br>Sperm count (mil/ejaculate)<br>Total motility (%)<br>Normal morphology (%)[3]	A significant change between baseline and 3 months of treatment was seen in sperm concentration and sperm count, where an increase was seen in the placebo group while a decline was seen in the intervention group. No significant differences were seen in

			normal uterine cavity and tubal patency.	selenomethionine), 1000 mg L-carnitine, 20 mg zinc, 1000 ug folic acid, 10 mg lycopene, 2000 IU vitamin D (n=85)			after 20 weeks' gestation)	DNA fragmentation (%)[3]	pregnancy and live birth rates compared to the placebo group.
Stenqvist (2018) Sweden	Parallel	Double-blinded	Infertile men with DFI of 25% or higher	Multiple substance dietary supplement: Combined antioxidant treatment (vitamin C 30 mg, vitamin E 5 mg, vitamin B12 0.5 ug, L-carnitine 750 mg, Coenzyme Q10 10 mg, folic acid 100 ug, zinc 5 mg and selenium 25 ug) (n=37)	Placebo (n=40)	6 months (26 weeks)	Pregnancies, undefined	Sperm concentration (mil/mL)<br>Sperm count (mil/ejaculate)<br>Total motility (%)<br>Progressive motility (%)<br>Normal morphology (%)<br>DNA fragmentation index (%)	Sperm concentration significantly improved after 3 months of intervention compared to baseline, however not after 6 months of intervention. No changes were seen in sperm count, total- and progressive motility, normal morphology and DNA fragmentation index compared to baseline. 3 pregnancies occurred in the intervention group and 4 in the placebo group.
Wong (2002) The Netherlands	Factorial	Double-blinded	Idiopathic infertile males, sperm concentration 5-20 million cells/mL with a history of not being able to conceive with a female partner after one year and fertile males[7]	Intervention 1: 5 mg folic acid pr day + placebo (n=22)<br>Intervention 2: 66 mg zinc sulphate pr day + placebo (n=23)<br>Intervention 3: 66 mg zinc sulphate + 5 mg folic acid per day (n=24)	Placebo + placebo (n=25)	26 weeks		Sperm concentration (mil/mL)<br>Total motility (%)<br>Normal morphology (%)	Sperm concentration was significantly improved after 26 weeks of zinc sulphate + folic acid supplementation in infertile men compared to baseline. No changes were seen in motility and normal morphology. Zinc + placebo as well as folic acid + placebo significantly improved normal sperm morphology compared to their respective baselines. No changes were observed in concentration and motility.

Závaczki (2003) Hungary	Parallel	Unclear	Infertile males with a history of infertility of 12 months or more having a healthy female partner + pathospermia according to WHO 1992 manual (ejaculate volume < 2 mL and/or sperm concentration <20 mil/mL and/or motility < 50 % and/or normal morphology ratio < 30%).	3000 mg magnesium-orotate per day (Magnerot tablets) (n=12)	Placebo (n=14)	90 days (13 weeks)	Clinical pregnancy, spontaneous (confirmed by ultrasound)[1]	Sperm concentration (mil/mL)<br>Total motility (%)<br>Normal morphology (%)	Magnesium-orotate supplement did not significantly improve sperm parameters compared to initial baseline values and the placebo group.
Zhaku (2022) North Macedonia	Parallel	Single-blinded	Infertile men with idiopathic oligoasthenozoospermia and a healthy female partner	Multiple substance dietary supplement: 500 mg maca substance three times daily, and a tablet consisting of 60 mg Korean ginseng extract. 100 mg vitamin C, 67 mg vitamin E, 15 mg zinc, 200 mg selenium, 250 mg L-arginine, 50 L-carnitine, 50 mg L-methionine and 50 L-phenylalanine two times a day (n=37)	Placebo (n=31)	6 months (26 weeks)		Sperm concentration (mil/mL)<br>Progressive motility (%)	26 weeks of antioxidant treatment resulted in a significant improvement in sperm concentration and progressive motility compared to baseline. No changes were seen in the placebo group.

[1]Not extracted as no reason for only reporting spontaneous pregnancies were stated. [2]Effect estimates not extracted as they were only based on a subpopulation. [3]Not extracted as these intervention arms were not relevant for this study. [4]Treatment duration ranged from 3 months and up to 6 months. The shortest treatment duration was extracted for quantitative synthesis. [5]Not extracted for quantitative synthesis as the percentages were unclear. [6]Not extracted for quantitative synthesis as the number of participants was unclear. [7]Only information on infertile males was extracted.

Table S6. Substance content of the dietary supplements administered by the included studies grouped as multiple substance dietary supplements.

**Author (year)**	**Substance content of multiple substance dietary supplement interventions**
Bahmyari (2021)	200 ug selenium, 400 IU vitamin E and 5mg folic acid per day
Busetto (2018)	2,000 mg L-carnitine, 1,450 mg fumarate, 1,000 mg acetyl-L-carnitine, 2,000 fructose, 40 mg Coenzyme Q10, 180 mg vitamin C, 20 mg zinc, 400 ug folic acid and 3 ug vitamin B12 per day
Gopinath (2013)	Intervention arm 1: 200 mg Coenzyme Q10, 2,000 mg L-carnitine, 10 mg lycopene and 60 mg zinc per day<br>Intervention arm 2: 100 mg Coenzyme Q10, 1,000 mg L-carnitine, 5 mg lycopene, 30 mg zinc per day
Kopets (2020)	1,990 mg L-carnitine/L-acetyl-carnitine, 250 mg L-arginine, 100 mg glutathione, 40 mg coenzyme Q10, 7.5 mg zinc, 234 ug vitamin B9, 2 ug vitamin B12 and 50 ug selenium per day
Kumar (2011)	Addyzoa ® consisting of powders 90 mg Purnachandrodaya rasa, 60 mg Suvarnavang, 60 mg Muktashukti bhasma, 60 mg Suvarnamakshik bhasma, 60 mg Shilajit shuddha, 30 mg Abhrak Bhasma, 30 mg Makardhwaj rasa, 10 mg Rasa sindur, and extracts 400 mg Gokshur, 400 mg Ashtavarga, 300 mg Shwet musli, 300 mg Kapikachchhu Shuddha, 300 mg Guduchi, 300 mg Ashwagandha, 150 mg Amalaki, 150 mg Balamool, 150 mg Vridhadharuk, 150 mg Shatavari, 60 mg Varahikand, 60 mg Chopchini, 60 mg Vidarikand and 30 mg Munjatak per day.
Patki (2023)	50 mg coenzyme Q10, 50 mg L-carnitine, 40 mg vitamin C, 10 mg vitamin E, 10 mg ginseng extract, 10 mg L-arginine, 20.588 mg zinc sulphate, 15.21 mg ferrous fumarate, 2.5 mg L-glutathione, 2 mg vitamin B6, 6.152 mg manganese sulphate, 2 mg lycopene, 1.4 mg vitamin B1, 2.795 mg cupric sulphate, 1250 IU vitamin A, 100 ug folic acid, 95.716 ug sodium selenate, 400 IU vitamin D, 1 ug vitamin B12 per day.
Scott (1998)	Intervention arm 2: 100 ug selenium, 1mg vitamin, 10 mg vitamin C and 15 mg vitamin E per day.
Steiner (2020)	500 mg Vitamin C, 400 mg vitamin E, 0.20 mg selenium, 1,000 mg L-carnitine, 20 mg zinc, 1,000 ug folic acid, 10 mg lycopene, 2,000 IU vitamin D per day.
Stenqvist (2018)	30 mg vitamin C, 5 mg vitamin E, 0.5 ug vitamin B12, 750 mg L-carnitine, 10 mg Coenzyme Q10, 100 ug folic acid, 5 mg zinc and 25 ug selenium per day.
Zhaku (2022)	1500 mg maca, 120 mg Korean ginseng extract, 200 mg vitamin C, 134 mg vitamin E, 30 mg zinc, 400 mg selenium, 500 mg L-arginine, 100 L-carnitine, 100 mg L-methionine and 100 L-phenylalanine per day.

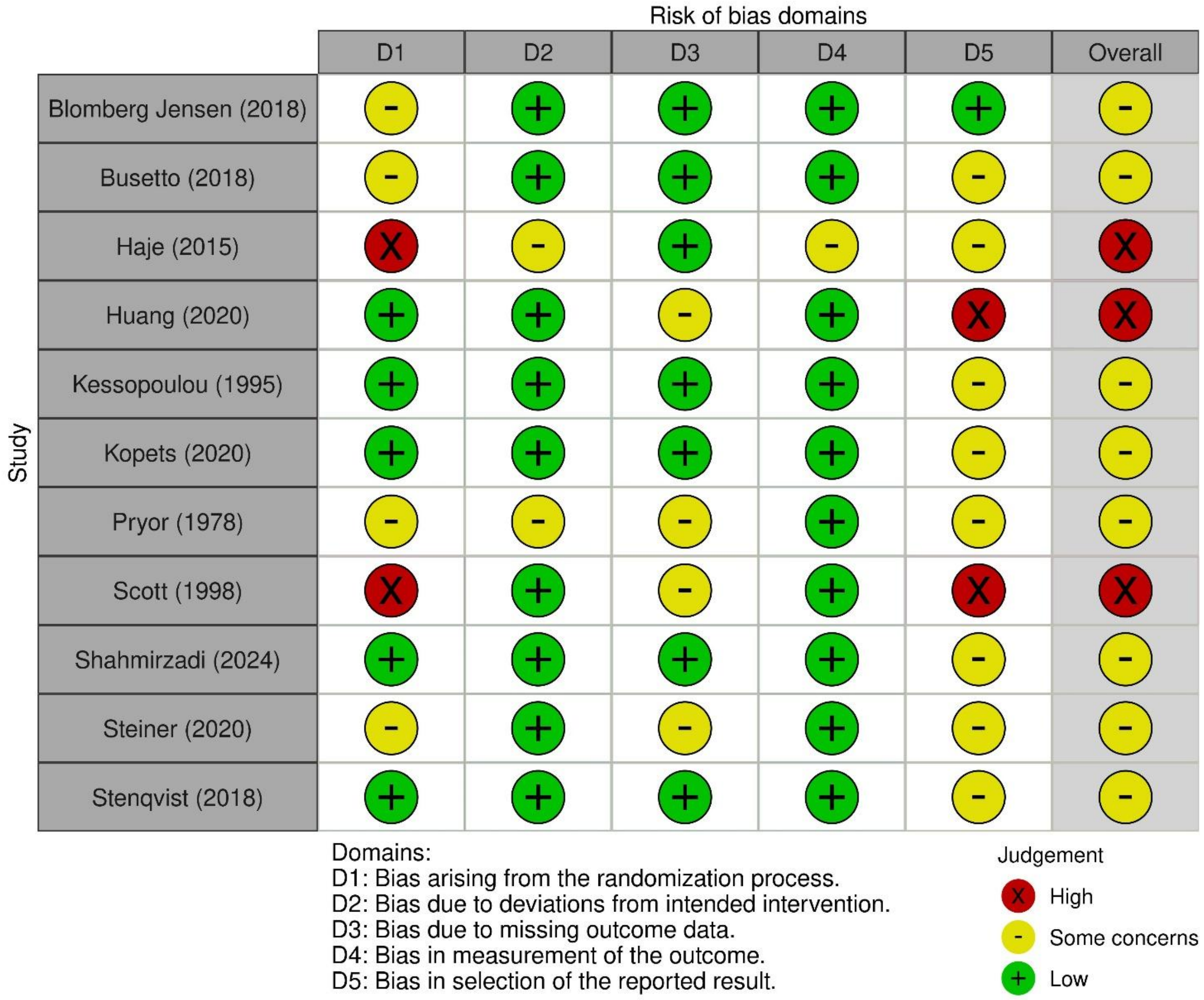

Figure S1. Individual risk of bias assessments on primary outcomes. Risk of bias assessments on the primary outcomes live birth and pregnancy. Risk of bias assessments have been visualized using the Robvis tool.

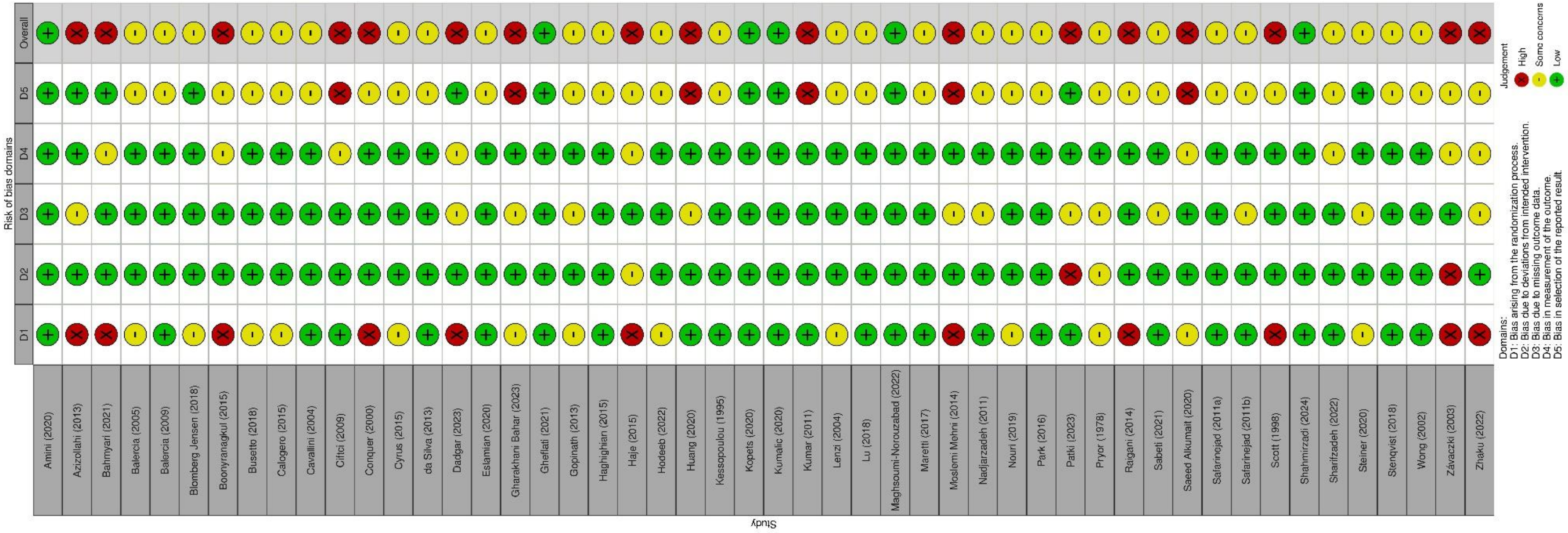

Figure S2. Individual risk of bias assessments on secondary outcome. Risk of bias assessments on the secondary outcomes sperm concentration, sperm count, total motility, progressive motility, normal morphology and DNA Fragmentation Index. Risk of bias assessments have been visualized using the Robvis tool.

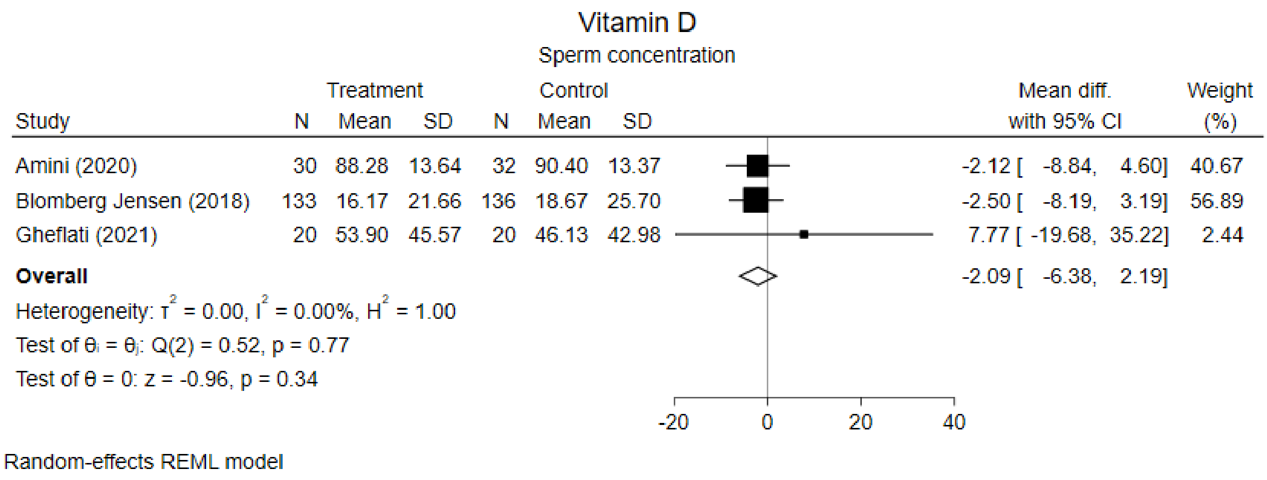

Figure S3. Forest plot of primary analysis on the effect of vitamin D on sperm concentration.

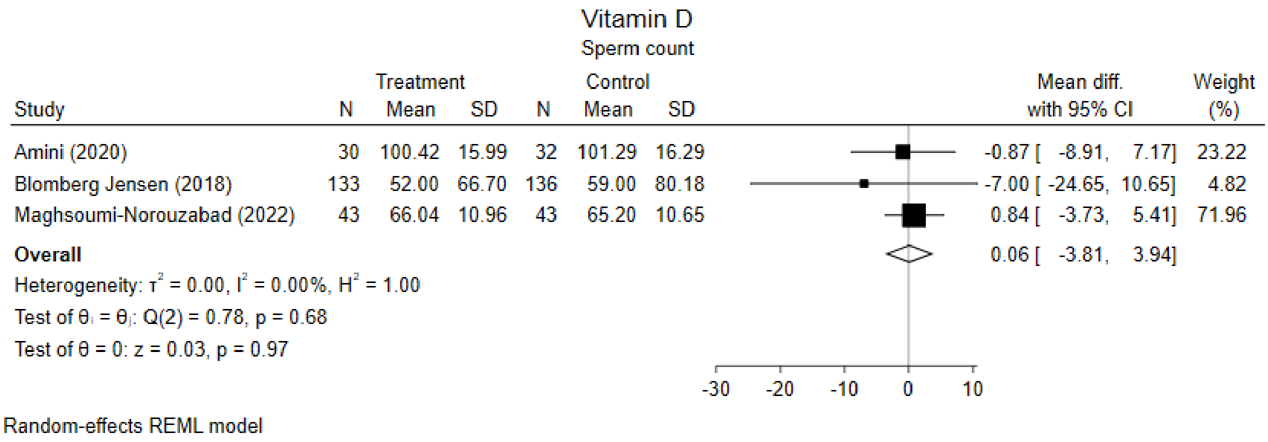

Figure S4. Forest plot of primary analysis on the effect of vitamin D on sperm count.

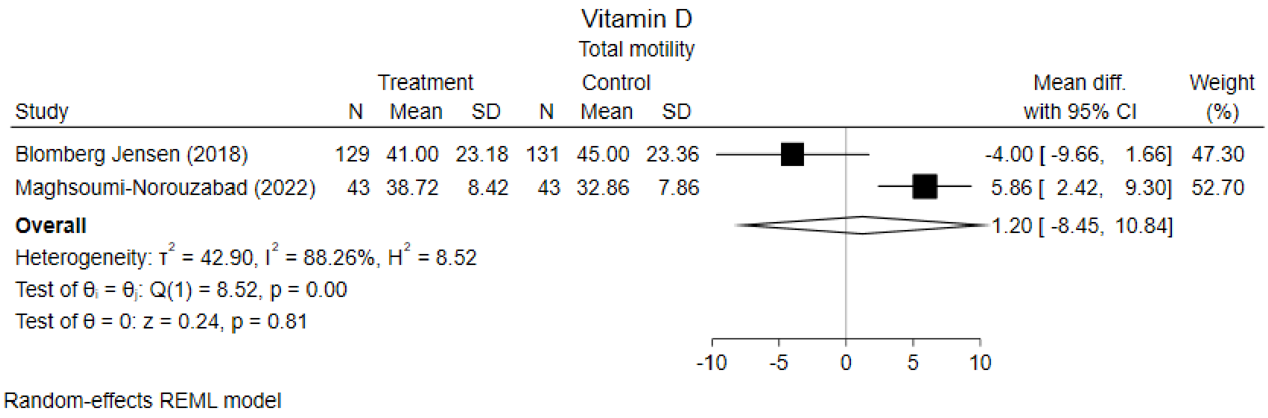

Figure S5. Forest plot of primary analysis on the effect of vitamin D on total motility.

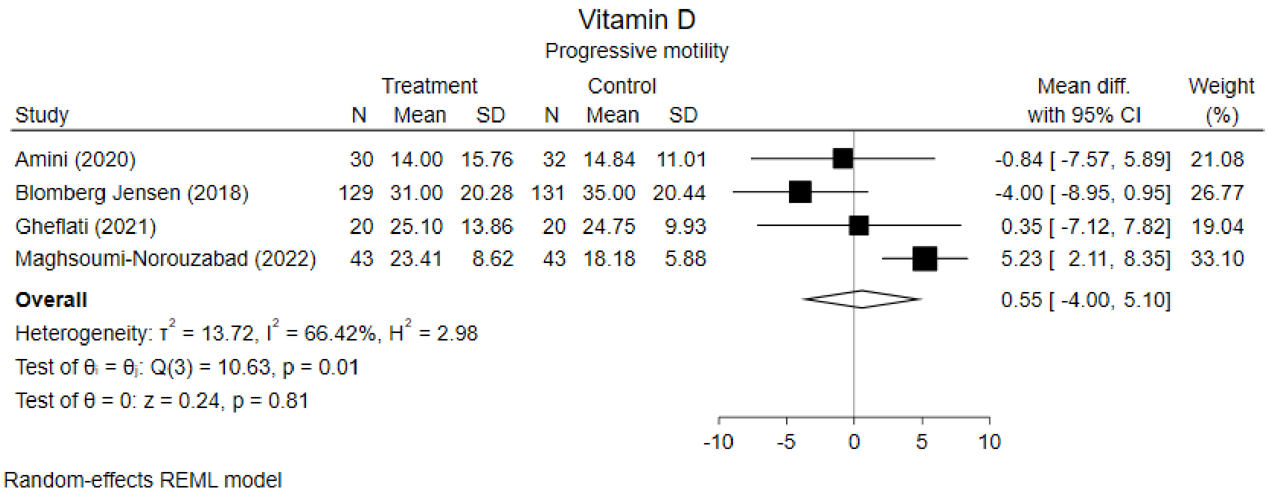

Figure S6. Forest plot of primary analysis on the effect of vitamin D on progressive motility.

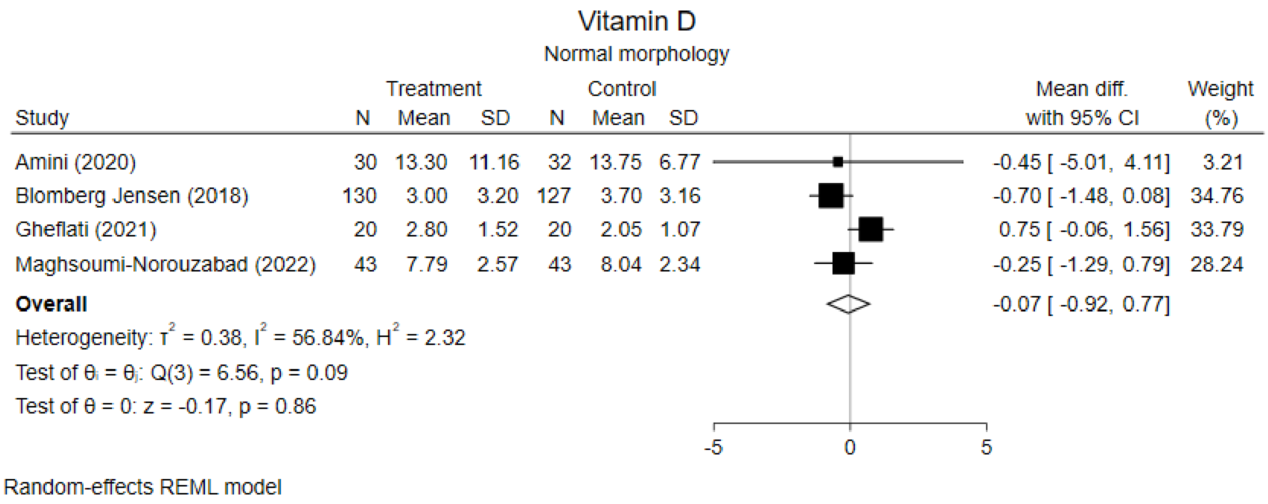

Figure S7. Forest plot of primary analysis on the effect of vitamin D on normal morphology.

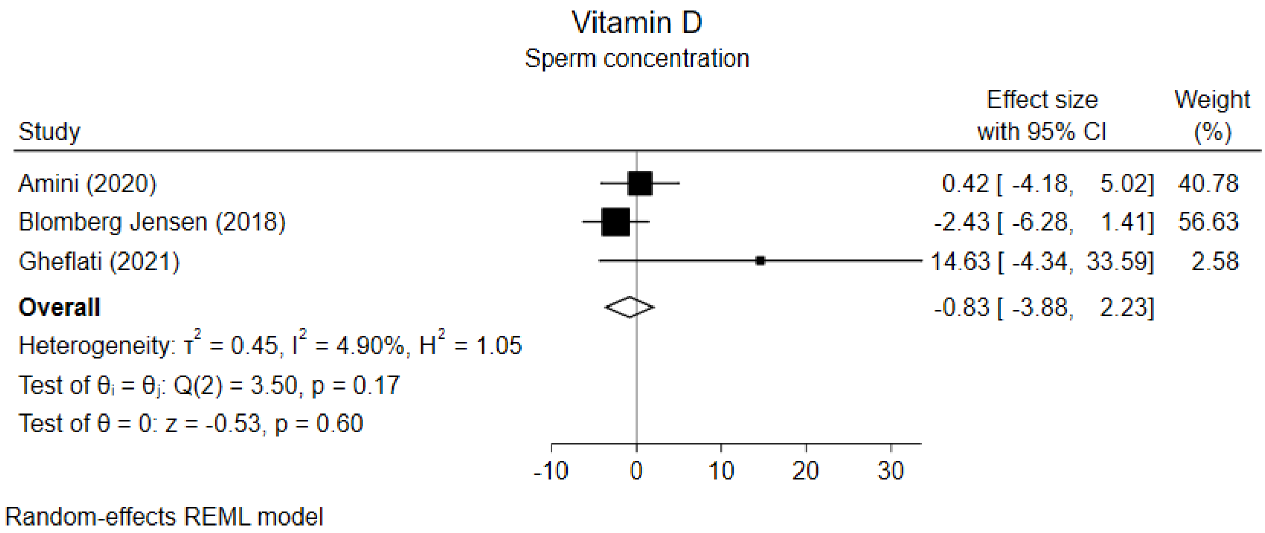

Figure S8. Forest plot of secondary analysis on the effect of vitamin D on sperm concentration. The analysis is adjusted for baseline using pseudo-individual participant data.

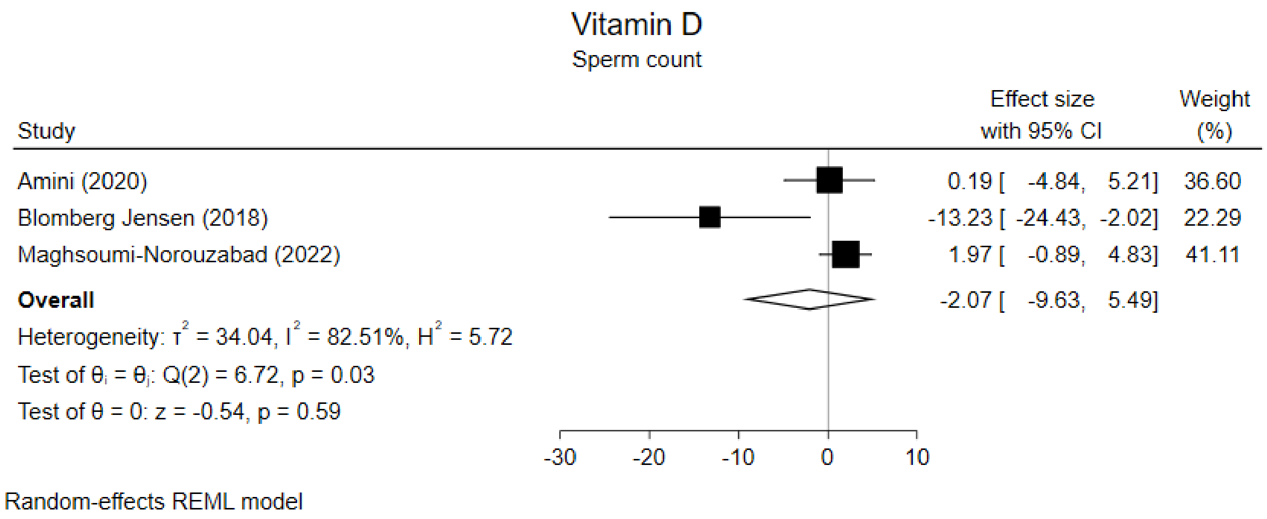

Figure S9. Forest plot of secondary analysis on the effect of vitamin D on sperm count. The analysis is adjusted for baseline using pseudo-individual participant data.

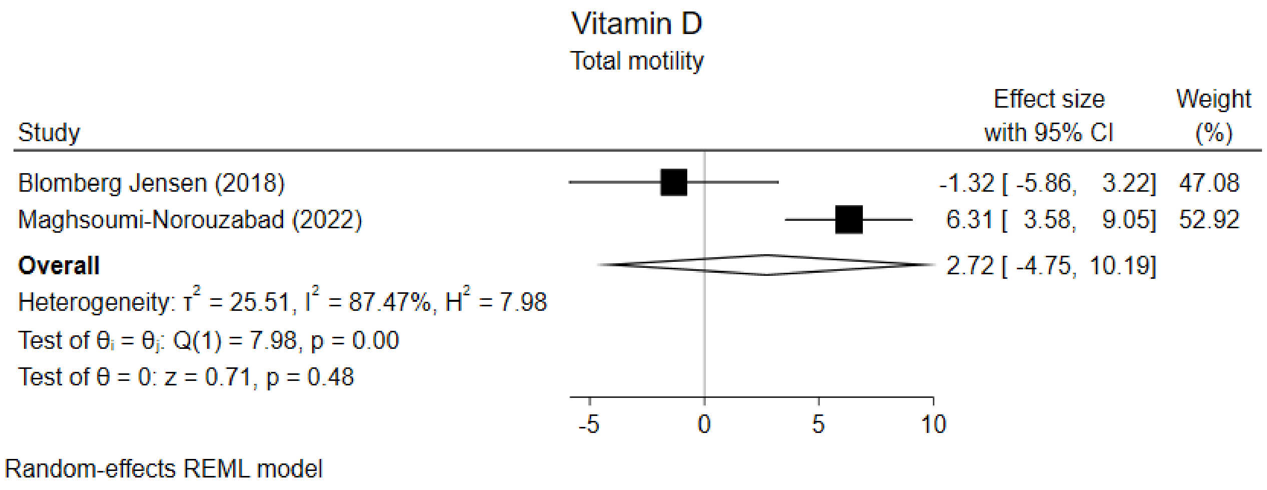

Figure S10. Forest plot of secondary analysis on the effect of vitamin D on total motility. The analysis is adjusted for baseline using pseudo-individual participant data.

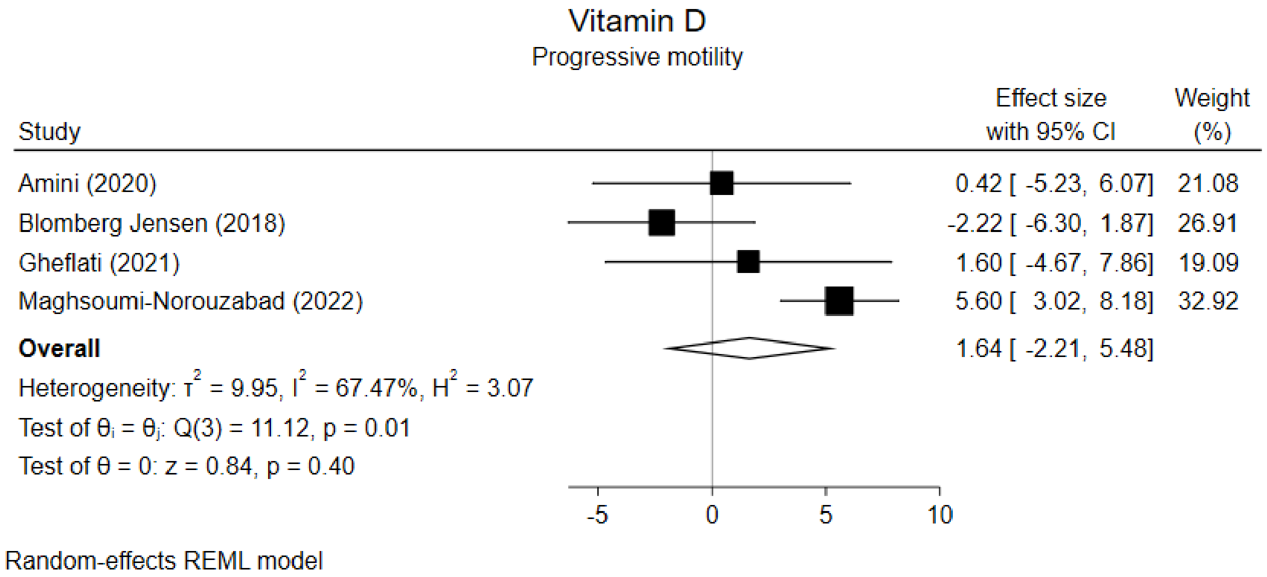

Figure S11. Forest plot of secondary analysis on the effect of vitamin D on progressive motility. The analysis is adjusted for baseline using pseudo-individual participant data.

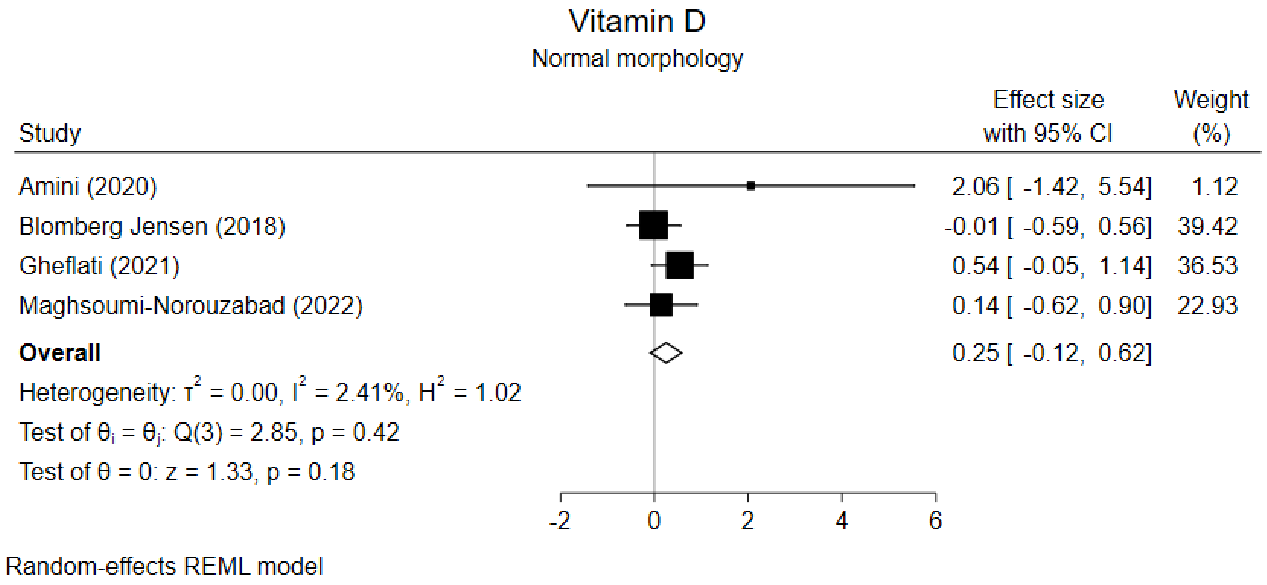

Figure S12. Forest plot of secondary analysis on the effect of vitamin D on normal morphology. The analysis is adjusted for baseline using pseudo-individual participant data.

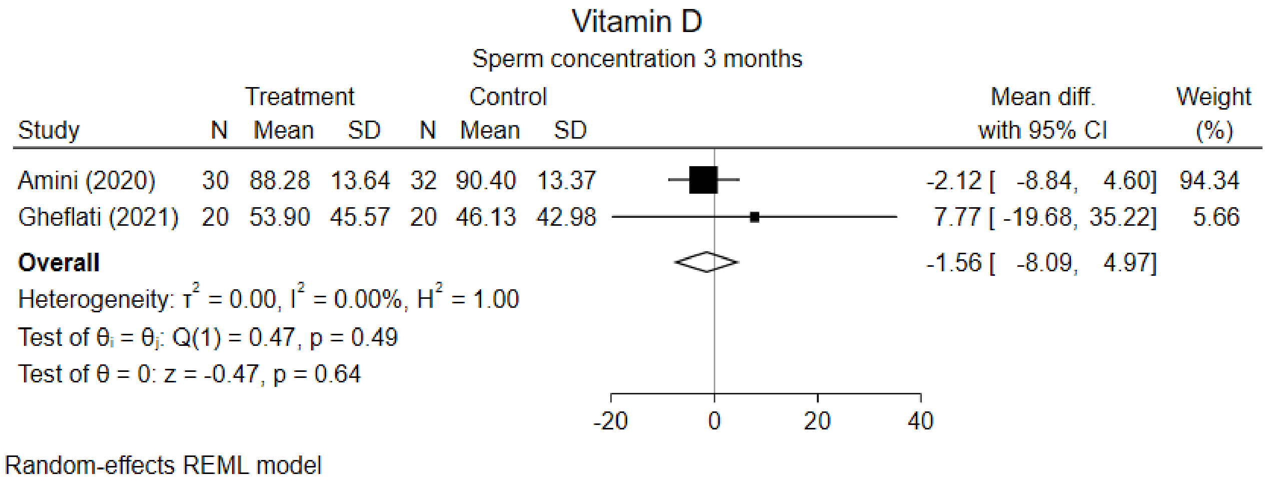

Figure S13. Forest plot of subgroup analysis on the effect of three months of vitamin D use on sperm concentration.

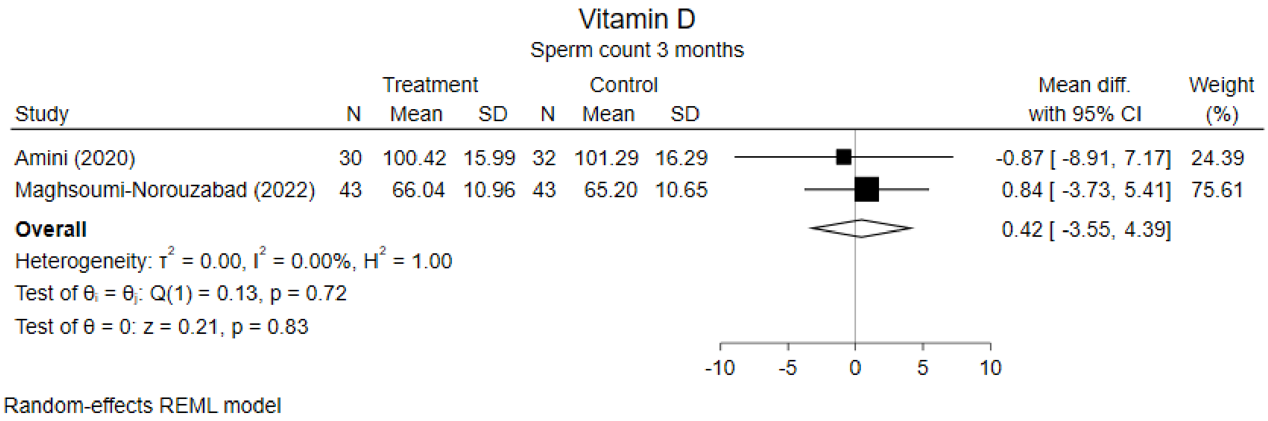

Figure S14. Forest plot of subgroup analysis on the effect of three months of vitamin D use on sperm count.

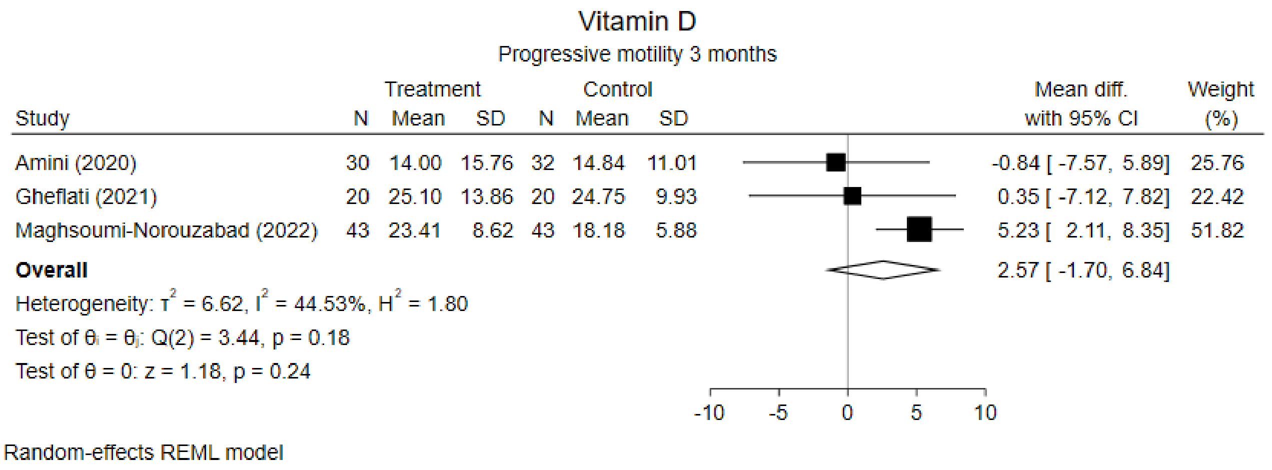

Figure S15. Forest plot of subgroup analysis on the effect of three months of vitamin D use on progressive motility.

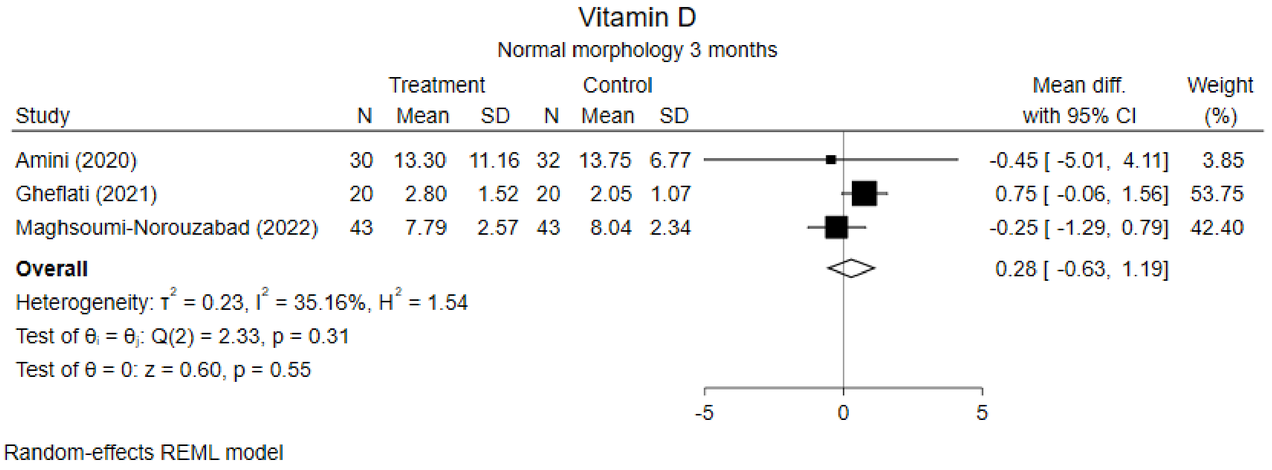

Figure S16. Forest plot of subgroup analysis on the effect of three months of vitamin D use on normal morphology.

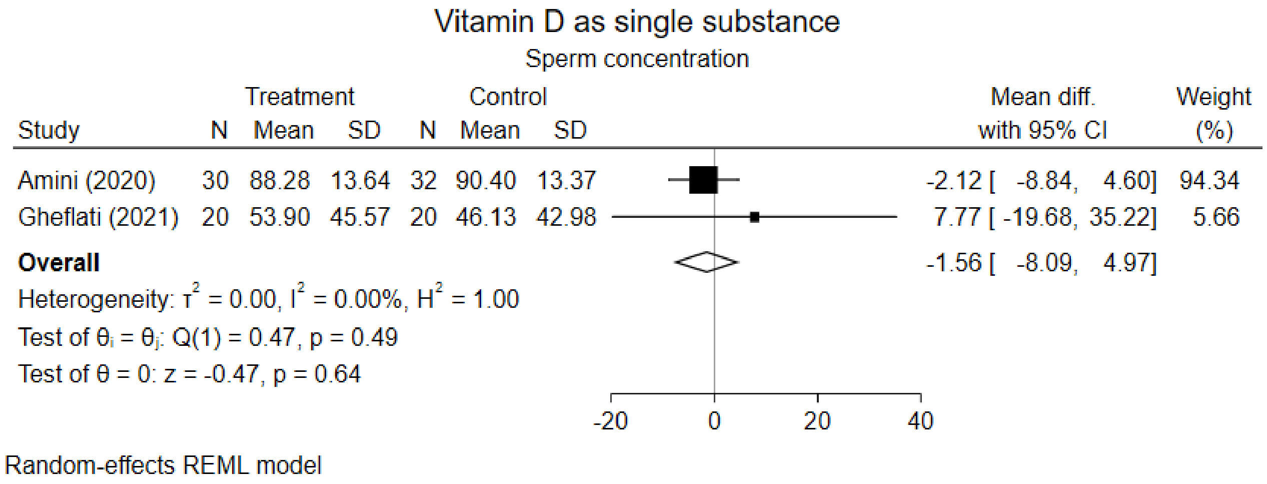

Figure S17. Forest plot of subgroup analysis on the effect of vitamin D as a single substance on sperm concentration.

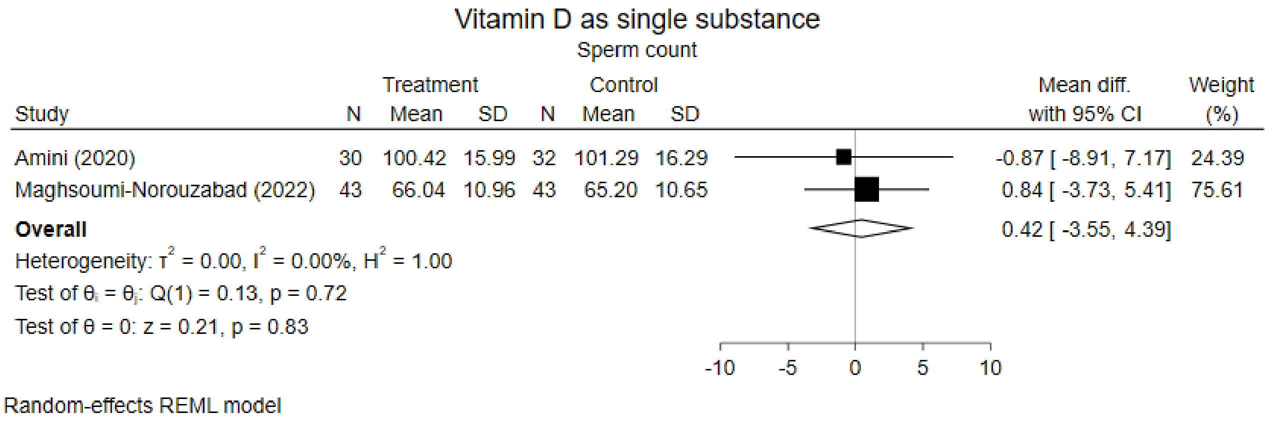

Figure S18. Forest plot of subgroup analysis on the effect of vitamin D as a single substance on sperm count.

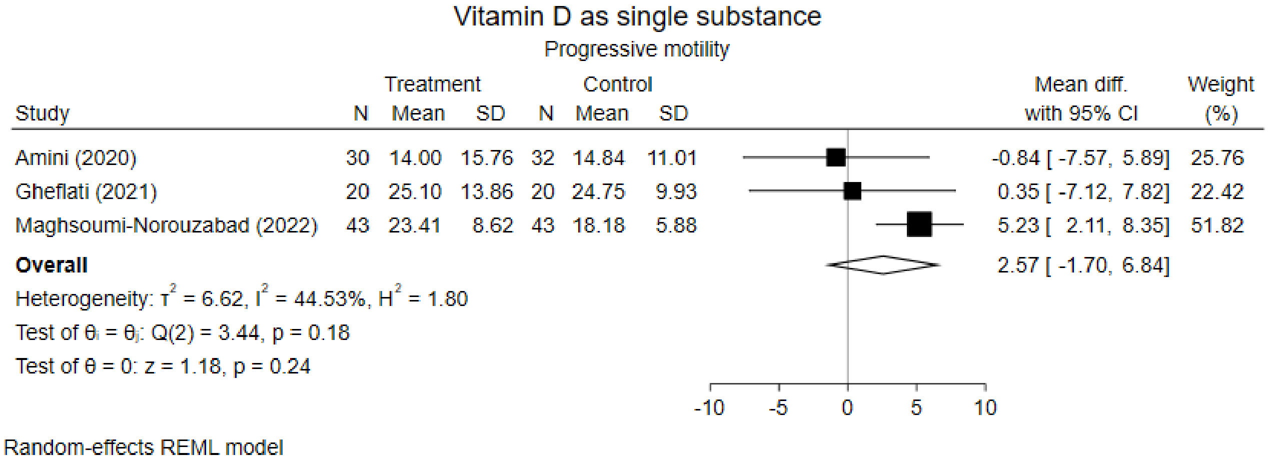

Figure S19. Forest plot of subgroup analysis on the effect of vitamin D as a single substance on progressive motility.

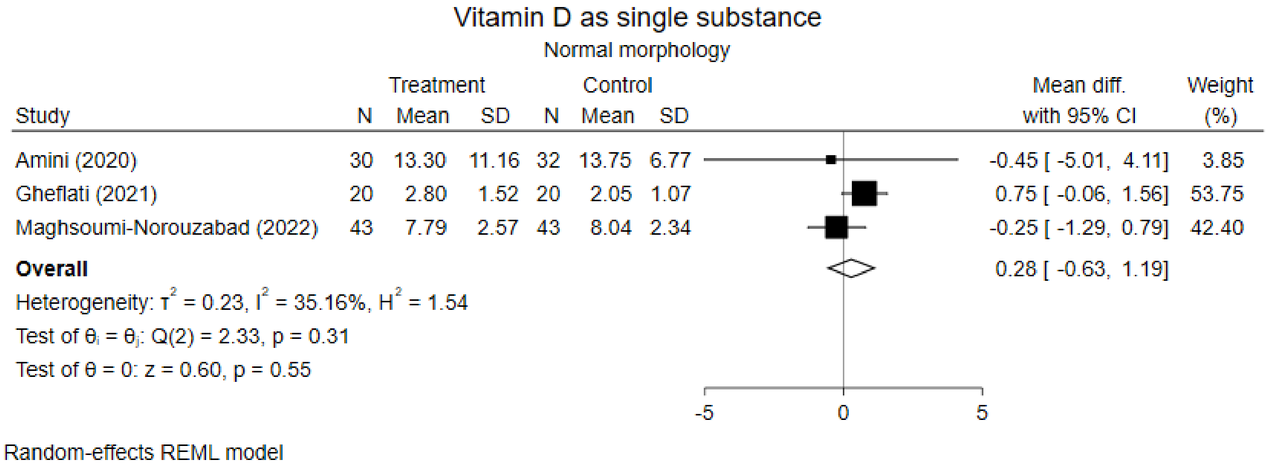

Figure S20. Forest plot of subgroup analysis on the effect of vitamin D as a single substance on normal morphology.

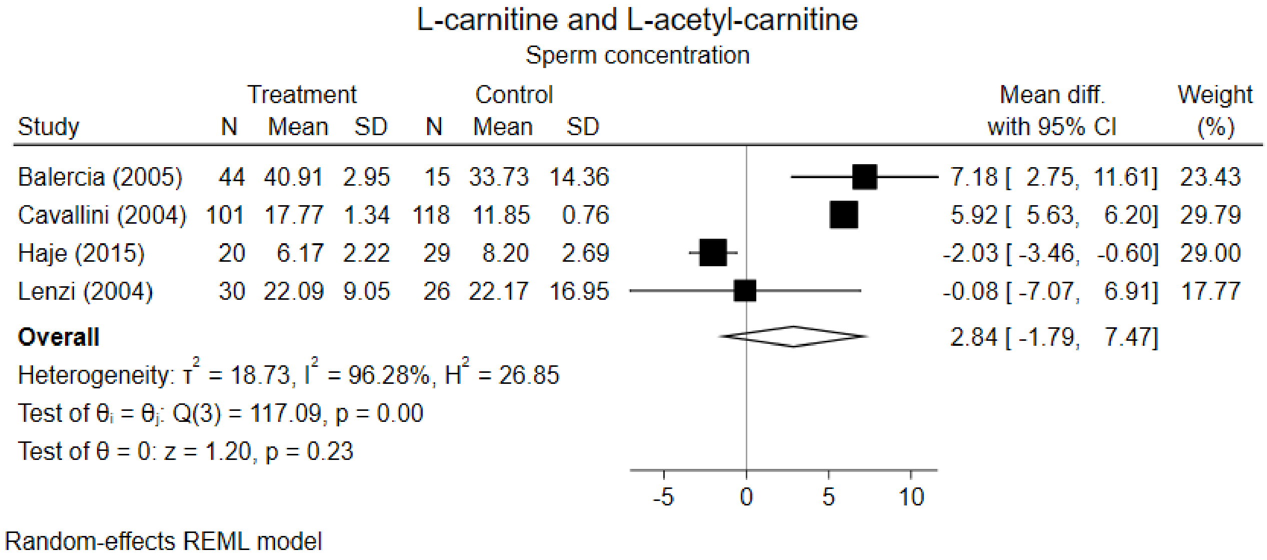

Figure S21. Forest plot of primary analysis on the effect of L-carnitine and L-acetyl-carnitine on sperm concentration.

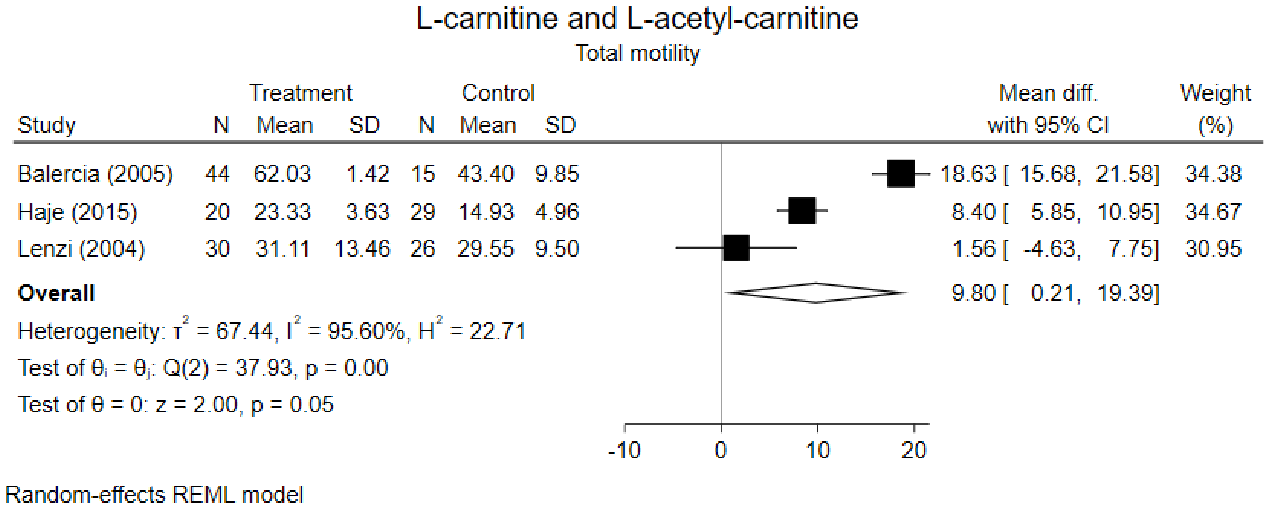

Figure S22. Forest plot of primary analysis on the effect of L-carnitine and L-acetyl-carnitine on total motility.

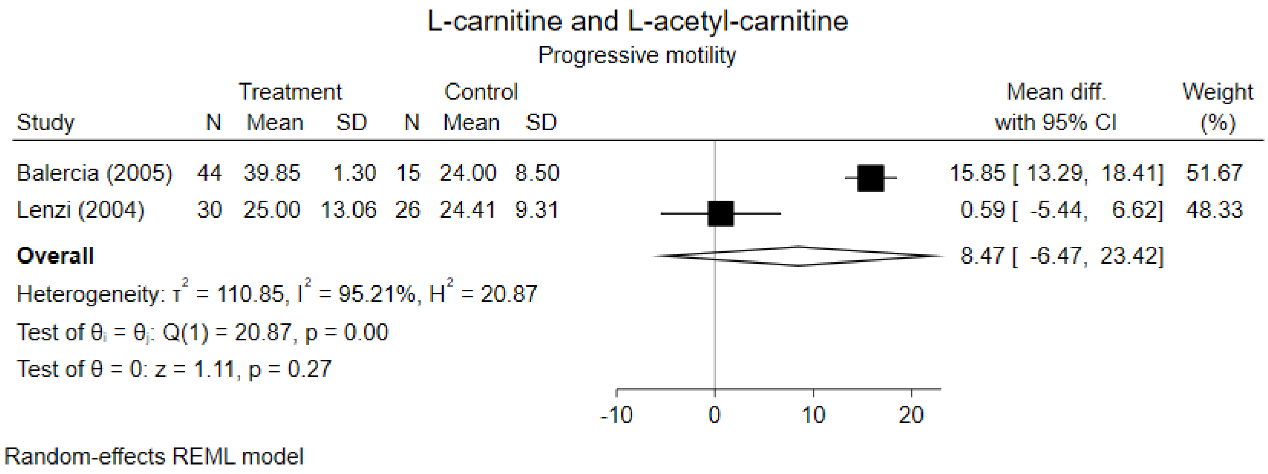

Figure S23. Forest plot of primary analysis on the effect of L-carnitine and L-acetyl-carnitine on progressive motility.

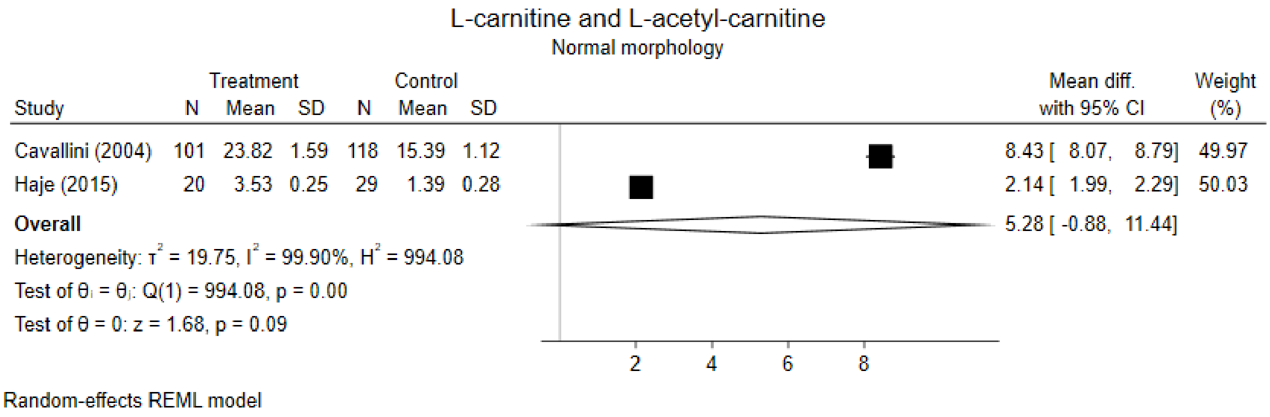

Figure S24. Forest plot of primary analysis on the effect of L-carnitine and L-acetyl-carnitine on normal morphology.

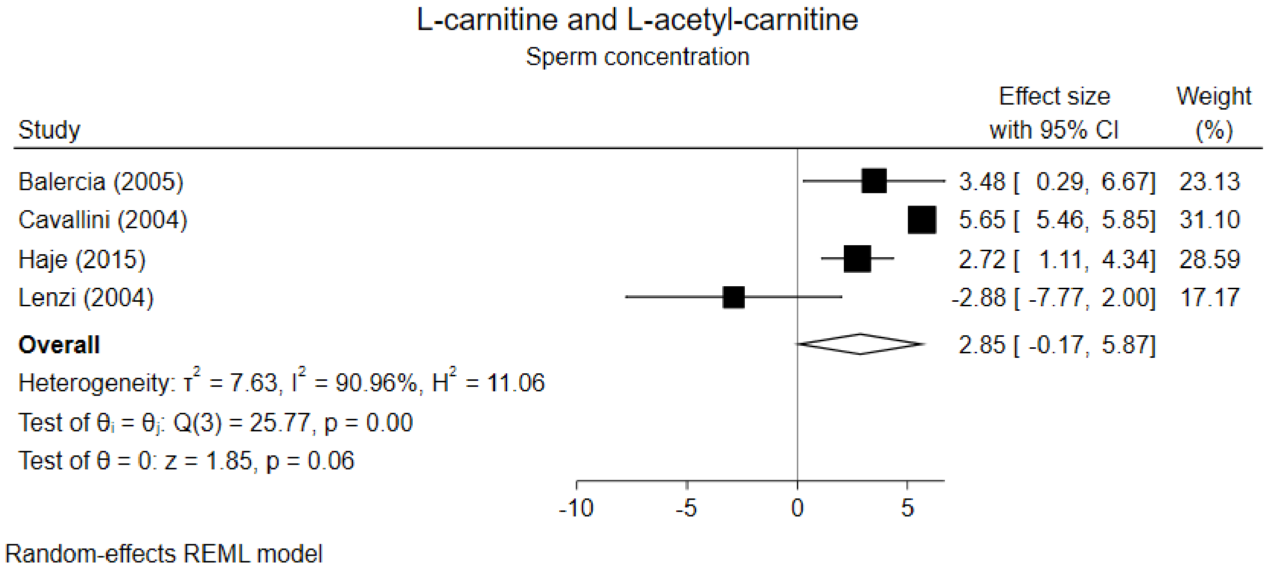

Figure S25. Forest plot of secondary analysis on the effect of L-carnitine and L-acetyl-carnitine on sperm concentration. The analysis is adjusted for baseline using pseudo-individual participant data.

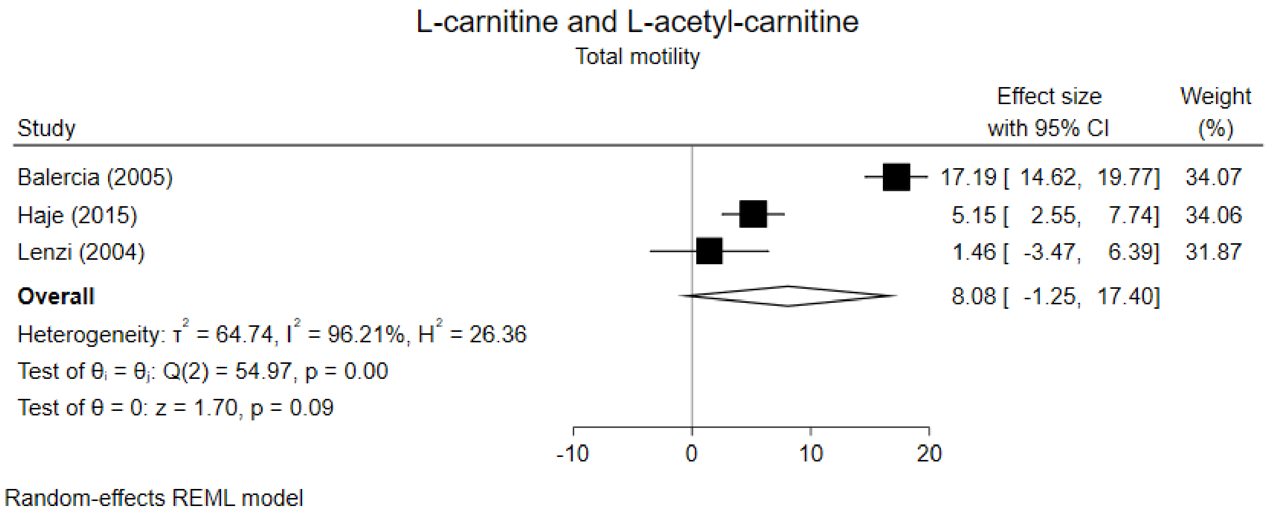

Figure S26. Forest plot of secondary analysis on the effect of L-carnitine and L-acetyl-carnitine on total motility. The analysis is adjusted for baseline using pseudo-individual participant data.

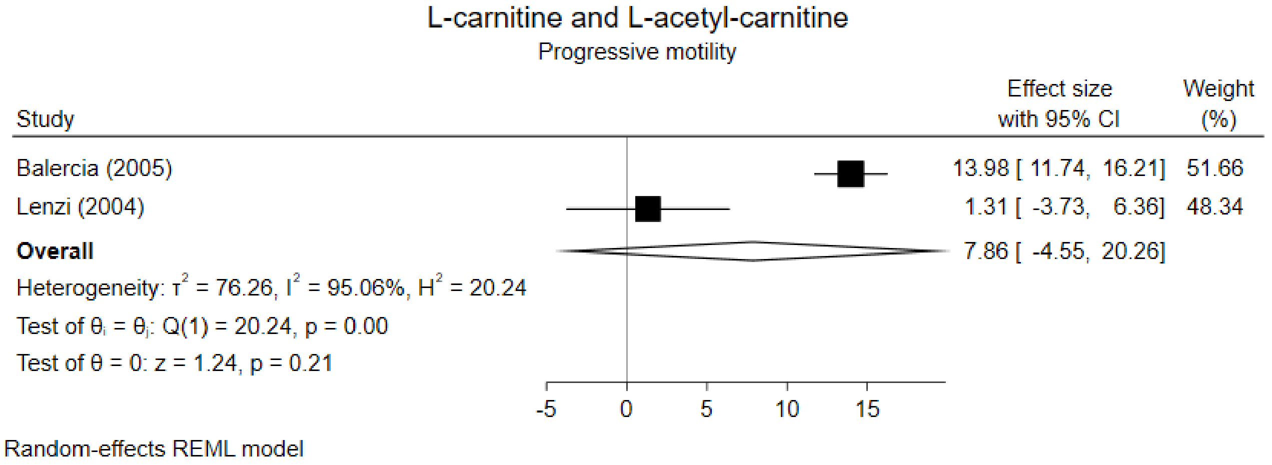

Figure S27. Forest plot of secondary analysis on the effect of L-carnitine and L-acetyl-carnitine on progressive motility. The analysis is adjusted for baseline using pseudo-individual participant data.

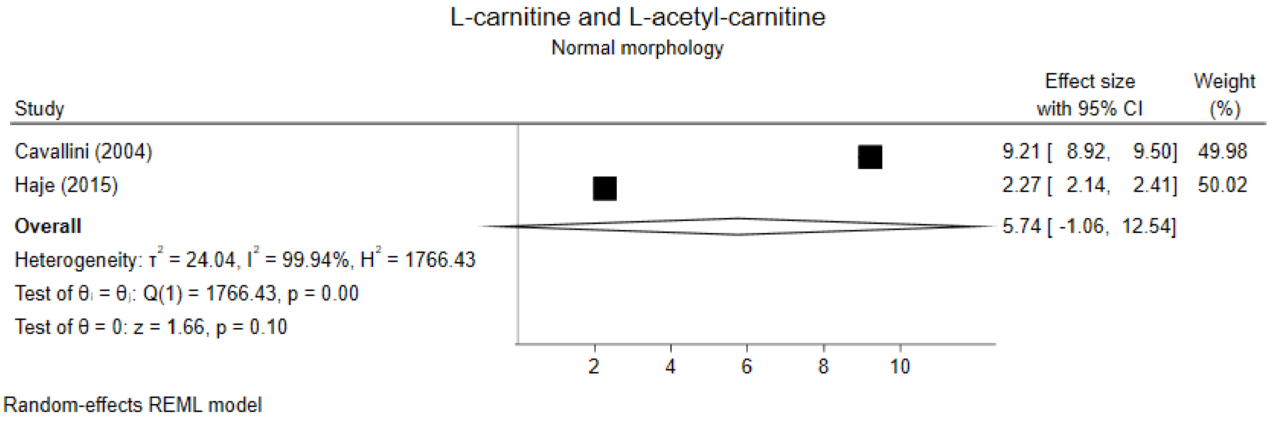

Figure S28. Forest plot of secondary analysis on the effect of L-carnitine and L-acetyl-carnitine on normal morphology. The analysis is adjusted for baseline using pseudo-individual participant data.

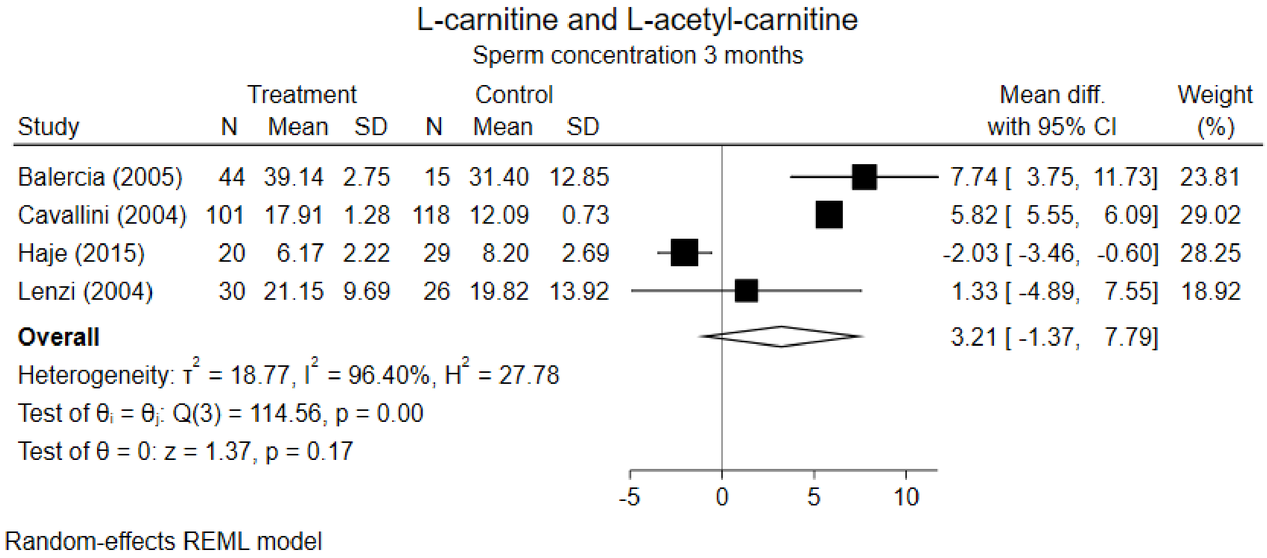

Figure S29. Forest plot of subgroup analysis on the effect of three months of L-carnitine and L-acetyl-carnitine use on sperm concentration.

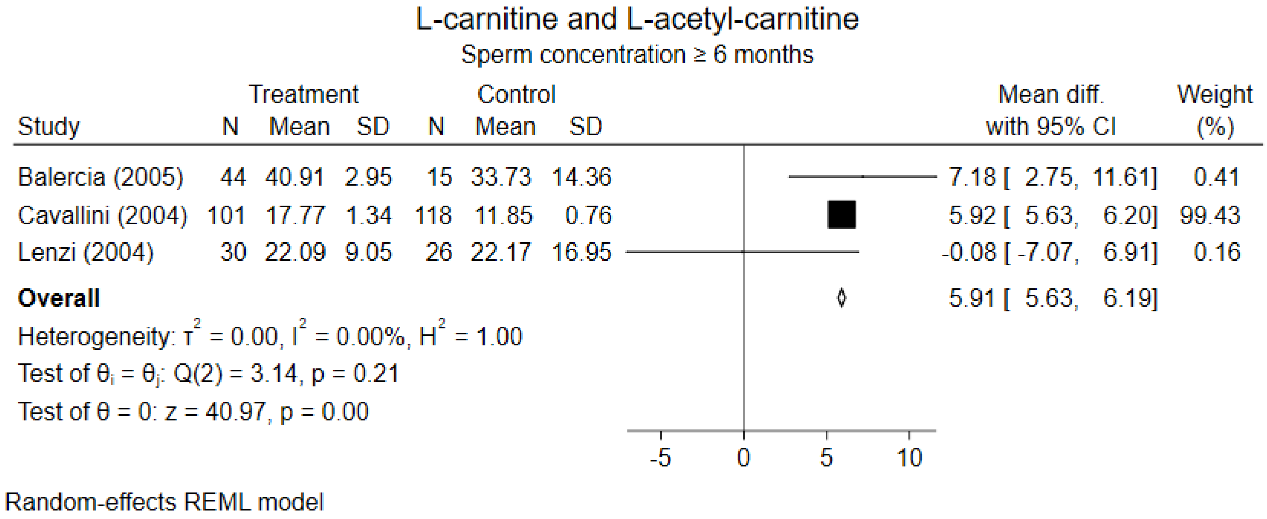

Figure S30. Forest plot of subgroup analysis on the effect of six or more months of L-carnitine and L-acetyl-carnitine use on sperm concentration.

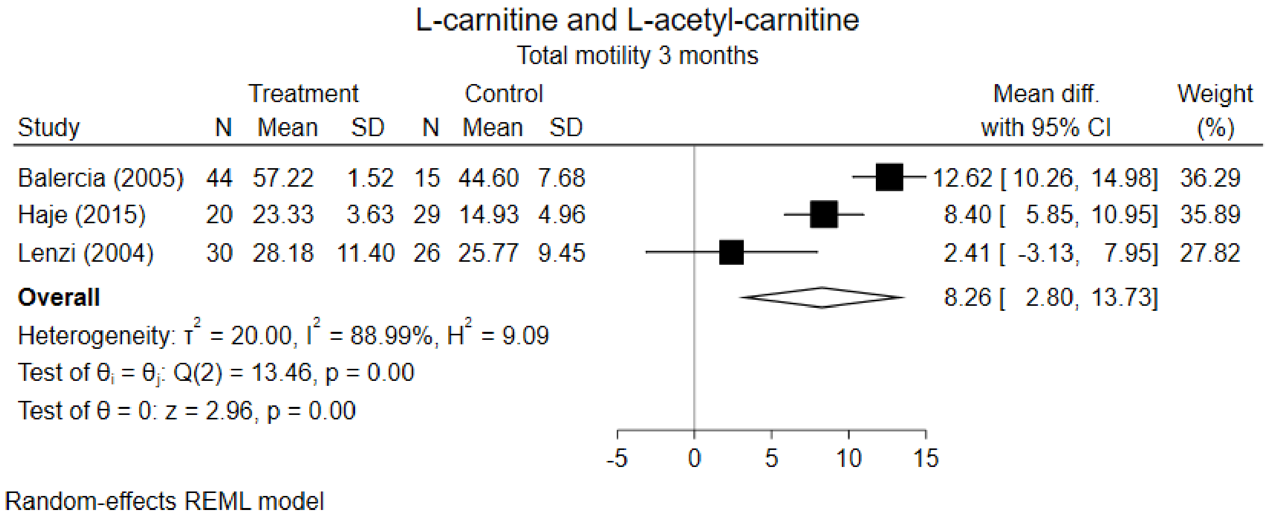

Figure S31. Forest plot of subgroup analysis on the effect of three months of L-carnitine and L-acetyl-carnitine use on total motility.

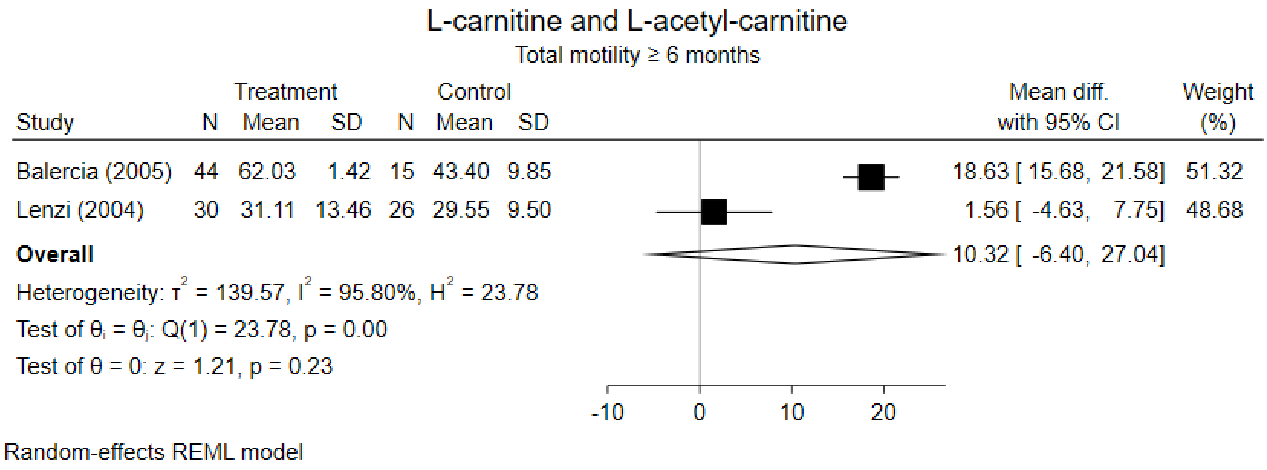

Figure S32. Forest plot of subgroup analysis on the effect of six or more months of L-carnitine and L-acetyl-carnitine use on total motility.

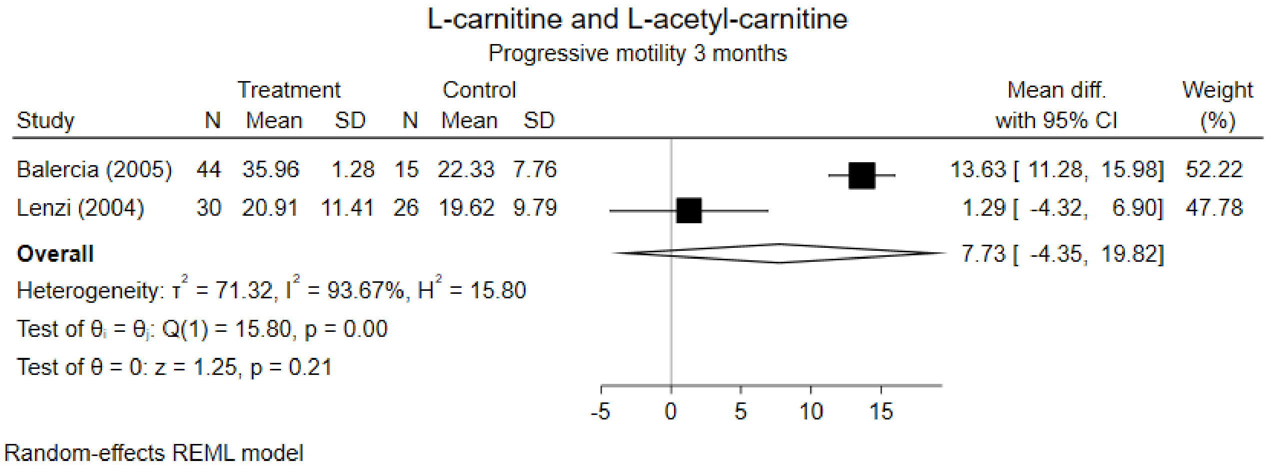

Figure S33. Forest plot of subgroup analysis on the effect of three months of L-carnitine and L-acetyl-carnitine use on progressive motility.

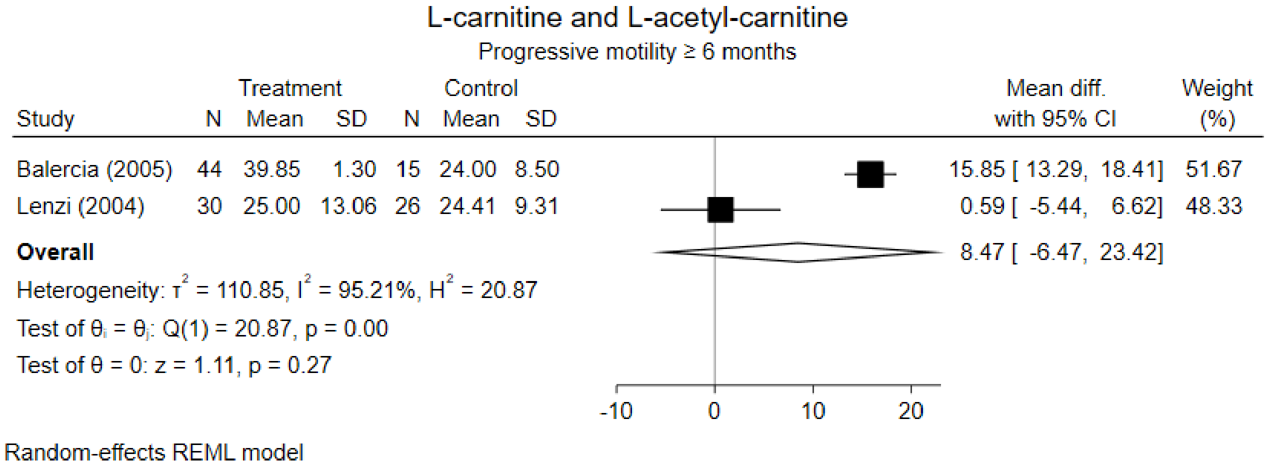

Figure S34. Forest plot of subgroup analysis on the effect of six or more months of L-carnitine and L-acetyl-carnitine use on progressive motility.

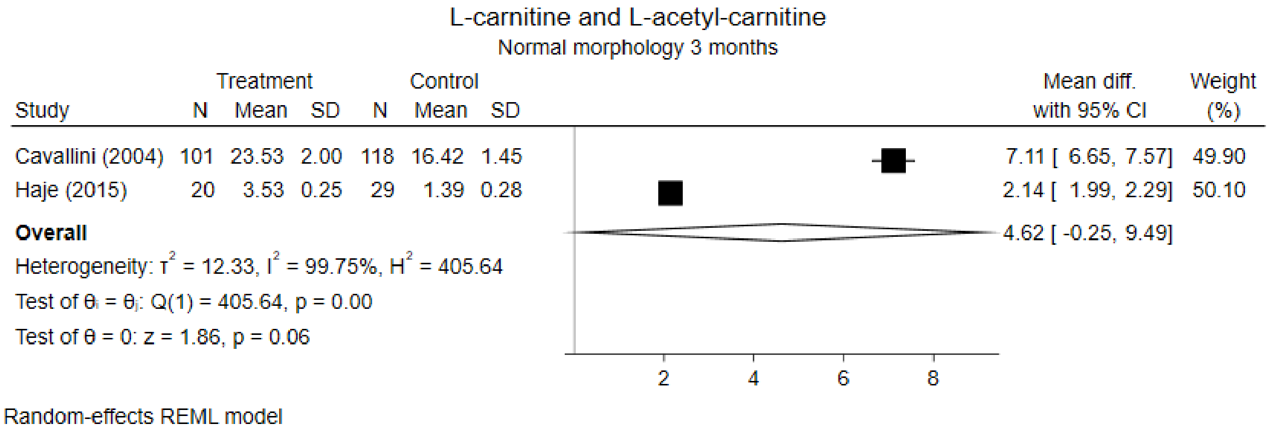

Figure S35. Forest plot of subgroup analysis on the effect of three months of L-carnitine and L-acetyl-carnitine use on normal morphology.

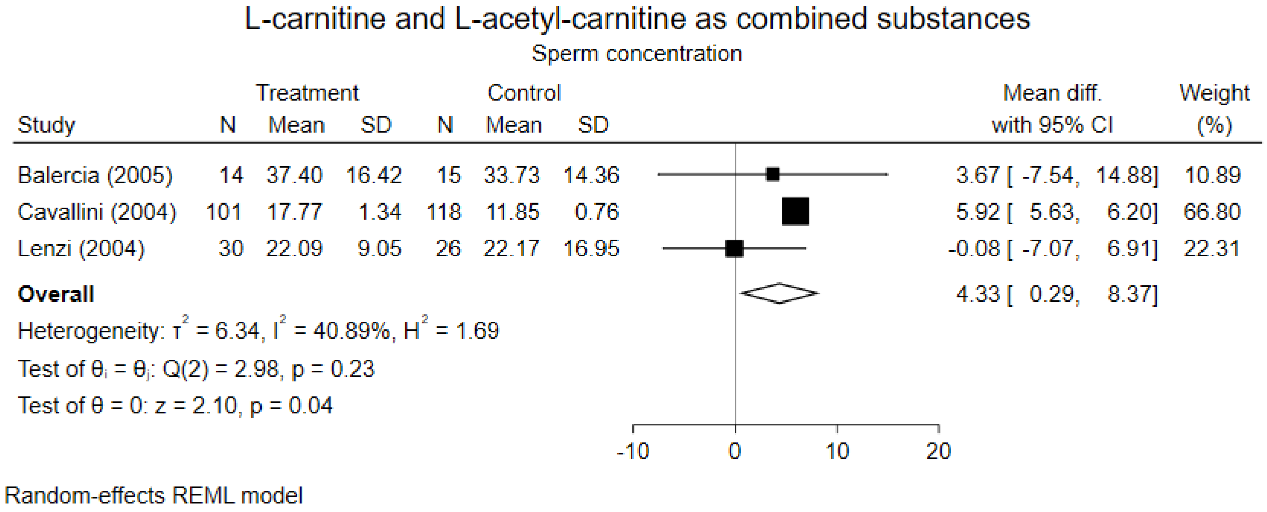

Figure S36. Forest plot of subgroup analysis on the effect of L-carnitine and L-acetyl-carnitine as combined substances on sperm concentration.

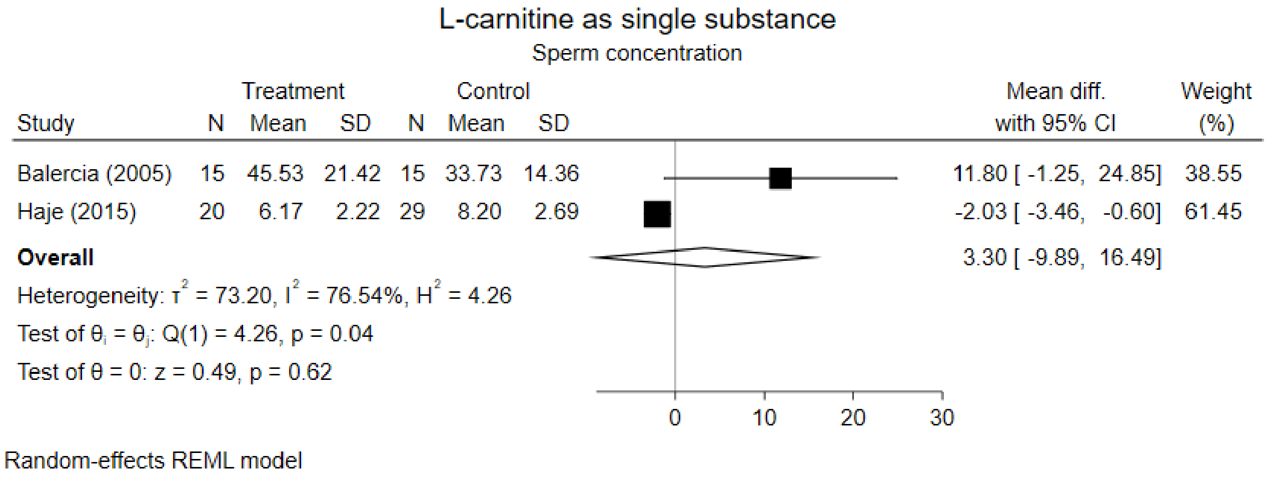

Figure S37. Forest plot of subgroup analysis on the effect of L-carnitine as a single substance on sperm concentration.

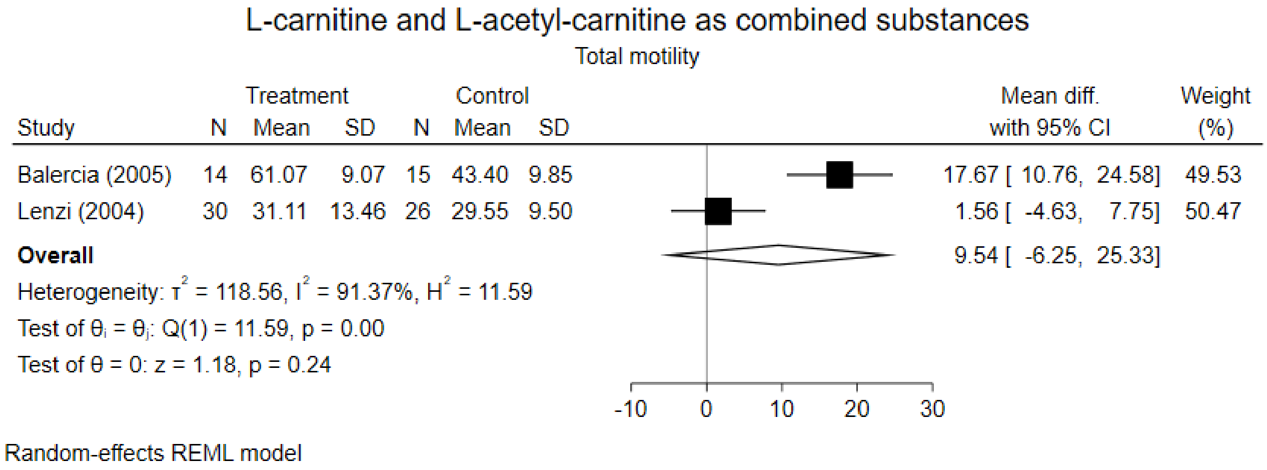

Figure S38. Forest plot of subgroup analysis on the effect of L-carnitine and L-acetyl-carnitine as combined substances on total motility.

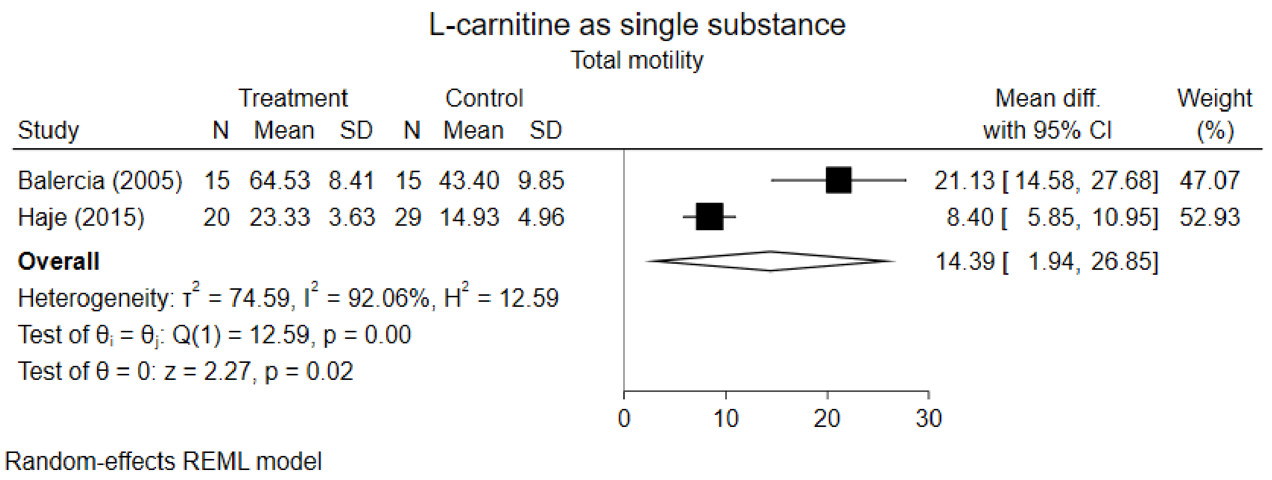

Figure S39. Forest plot of subgroup analysis on the effect of L-carnitine as a single substance on total motility.

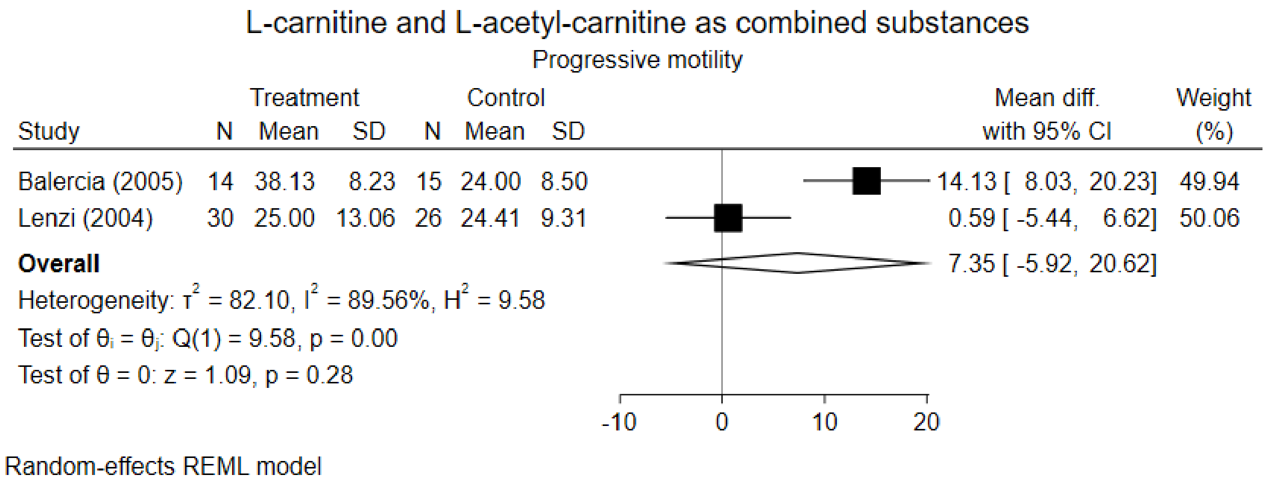

Figure S40. Forest plot of subgroup analysis on the effect of L-carnitine and L-acetyl-carnitine as combined substances on progressive motility.

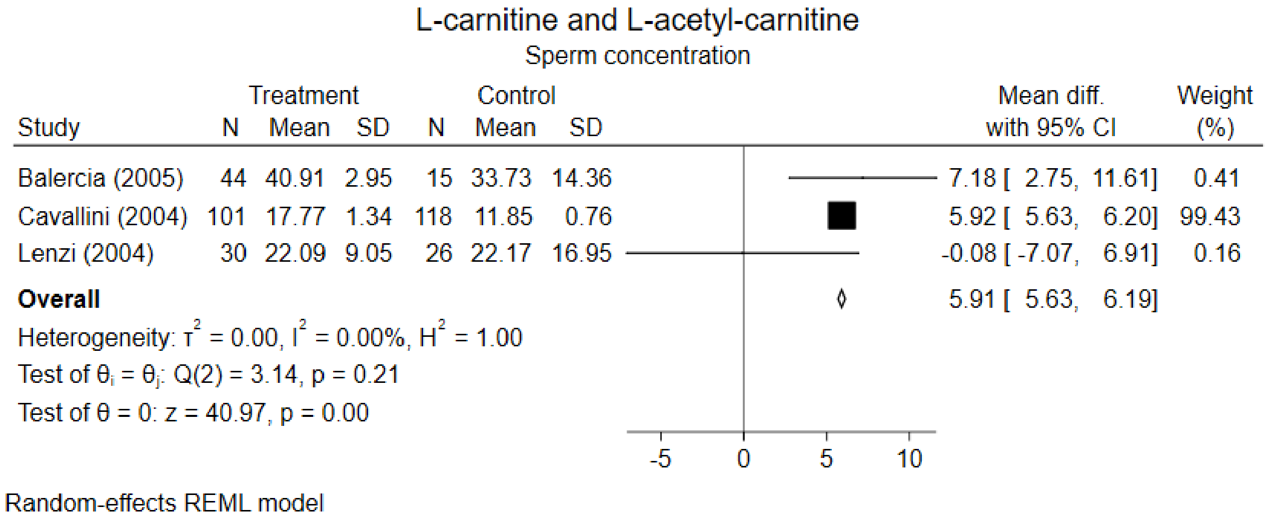

Figure S41. Forest plot on sensitivity analysis on the effect of L-carnitine and L-acetyl-carnitine on sperm concentration. Studies evaluated as having a high risk of bias have been excluded from the analysis.

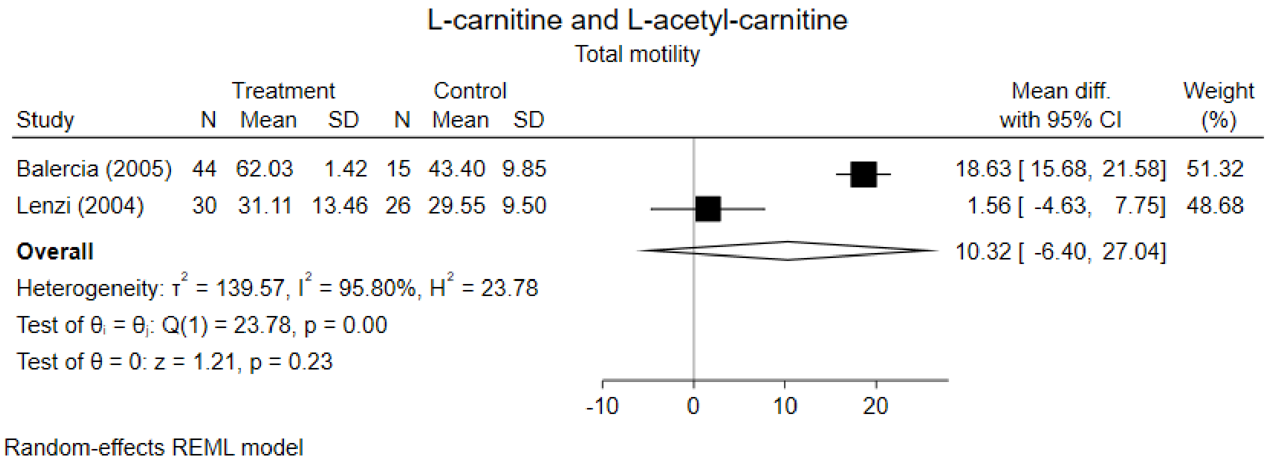

Figure S42. Forest plot on sensitivity analysis on the effect of L-carnitine and L-acetyl-carnitine on total motility. Studies evaluated as having a high risk of bias have been excluded from the analysis.

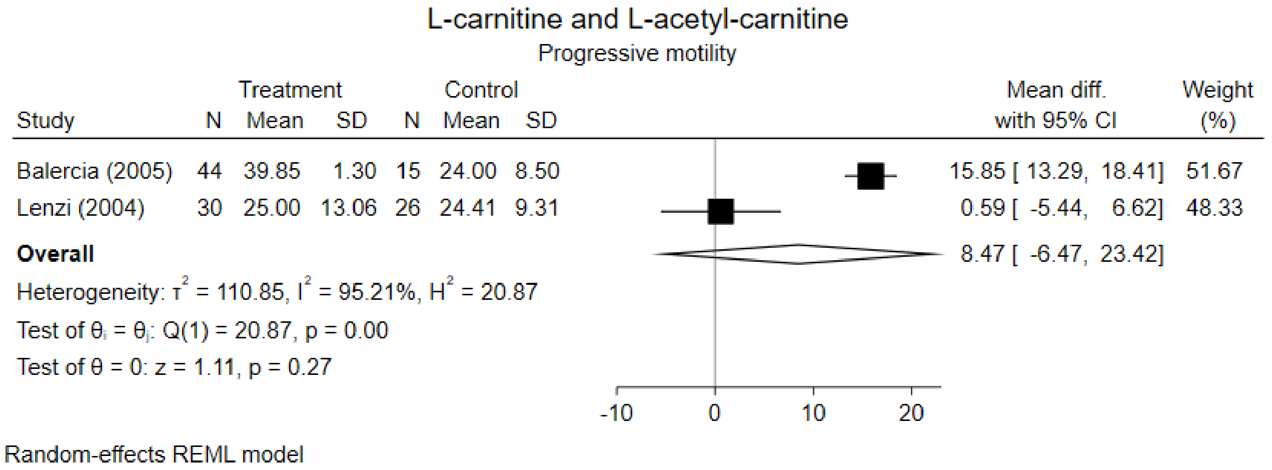

Figure S43. Forest plot on sensitivity analysis on the effect of L-carnitine and L-acetyl-carnitine on progressive motility. Studies evaluated as having a high risk of bias have been excluded from the analysis.

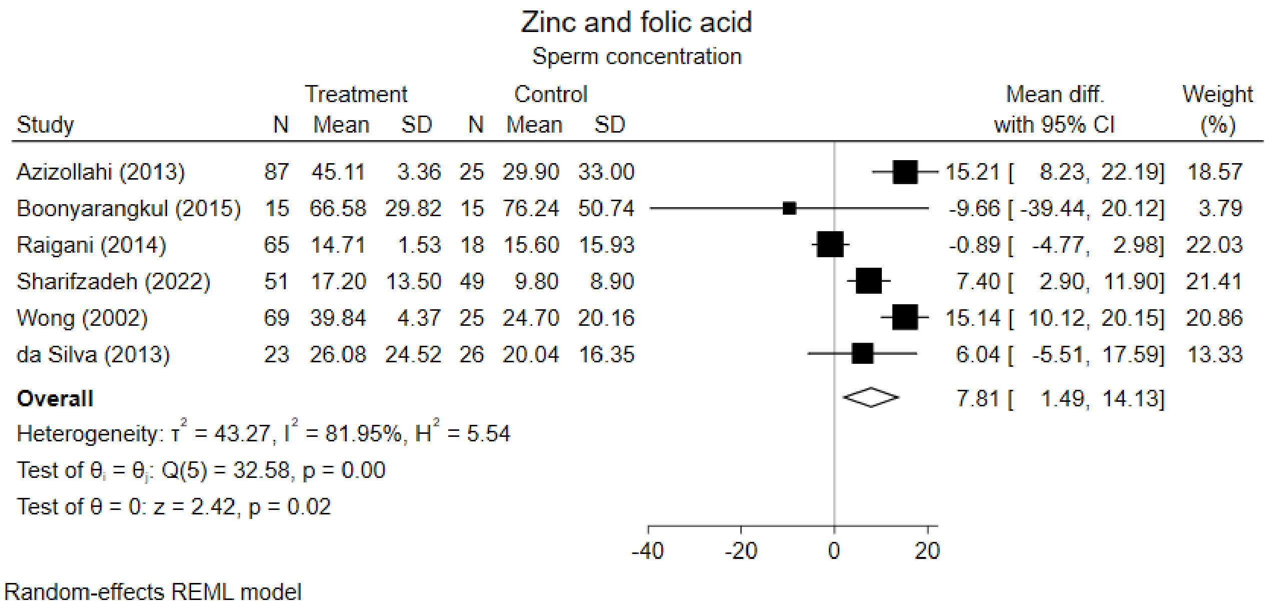

Figure S44. Forest plot of primary analysis on the effect of zinc and folic acid on sperm concentration.

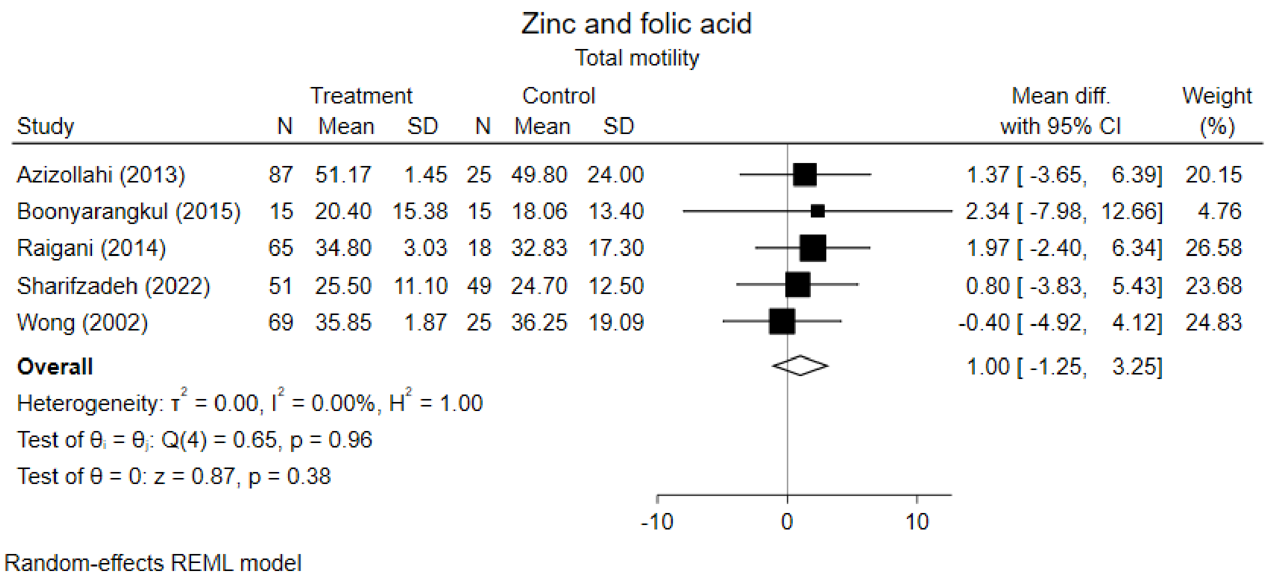

Figure S45. Forest plot of primary analysis on the effect of zinc and folic acid on total motility.

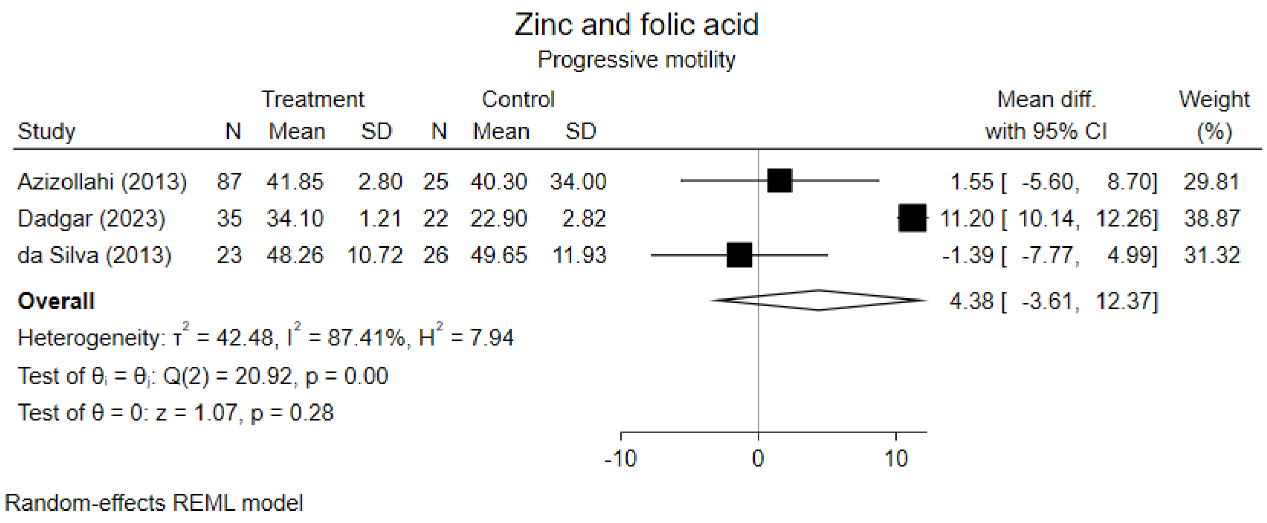

Figure S46. Forest plot of primary analysis on the effect of zinc and folic acid on progressive motility.

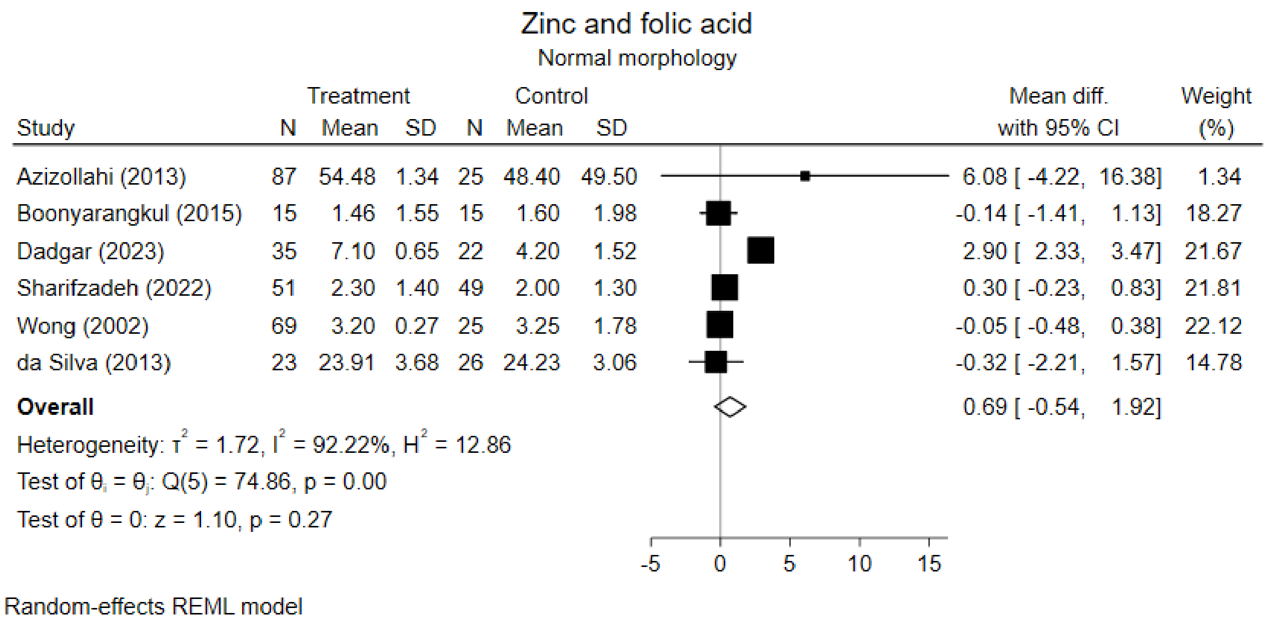

Figure S47. Forest plot of primary analysis on the effect of zinc and folic acid on normal morphology.

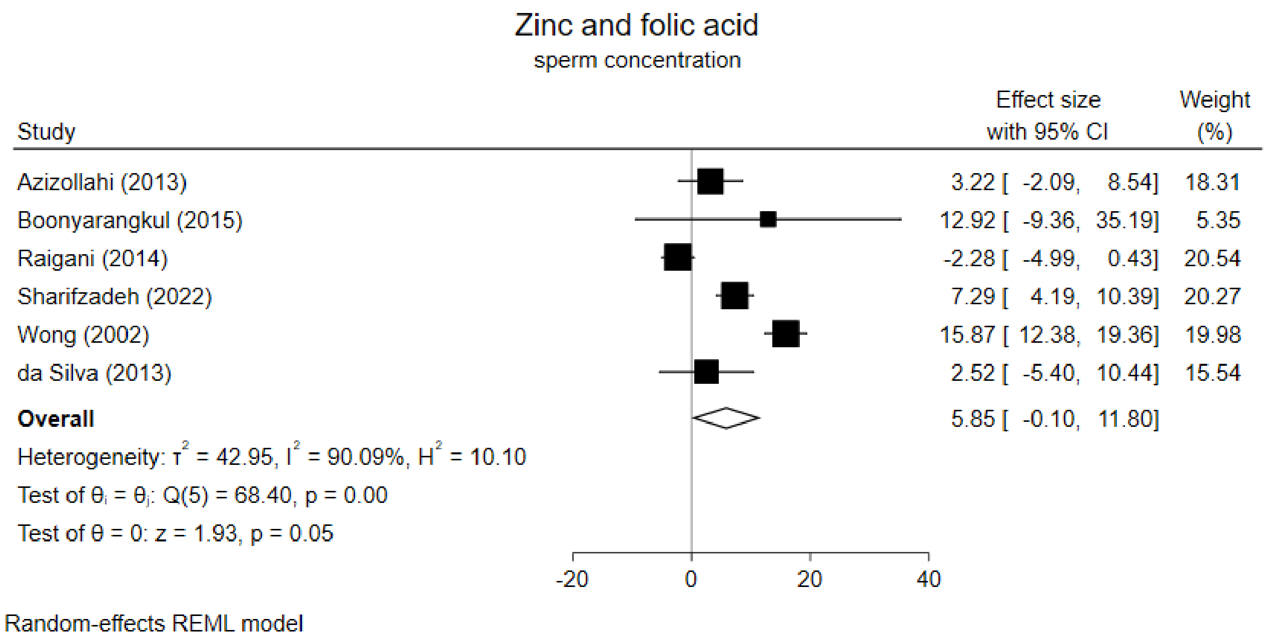

Figure S48. Forest plot of secondary analysis on the effect of zinc and folic acid on sperm concentration. The analysis is adjusted for baseline using pseudo-individual participant data.

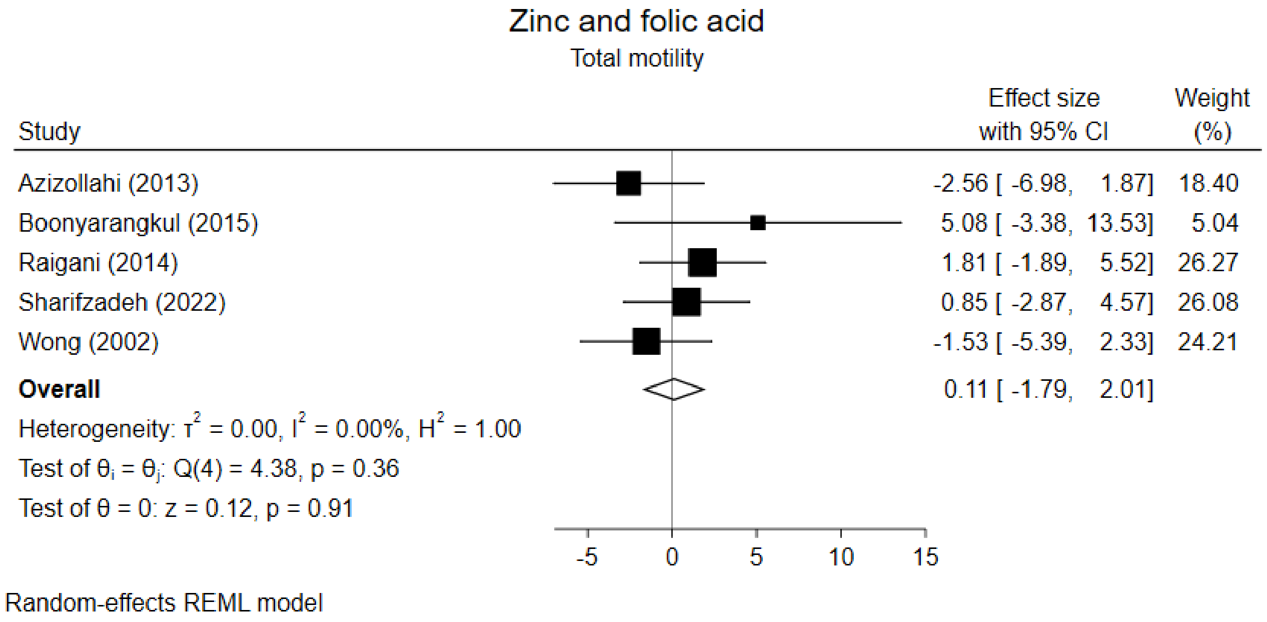

Figure S49. Forest plot of secondary analysis on the effect of zinc and folic acid on total motility. The analysis is adjusted for baseline using pseudo-individual participant data.

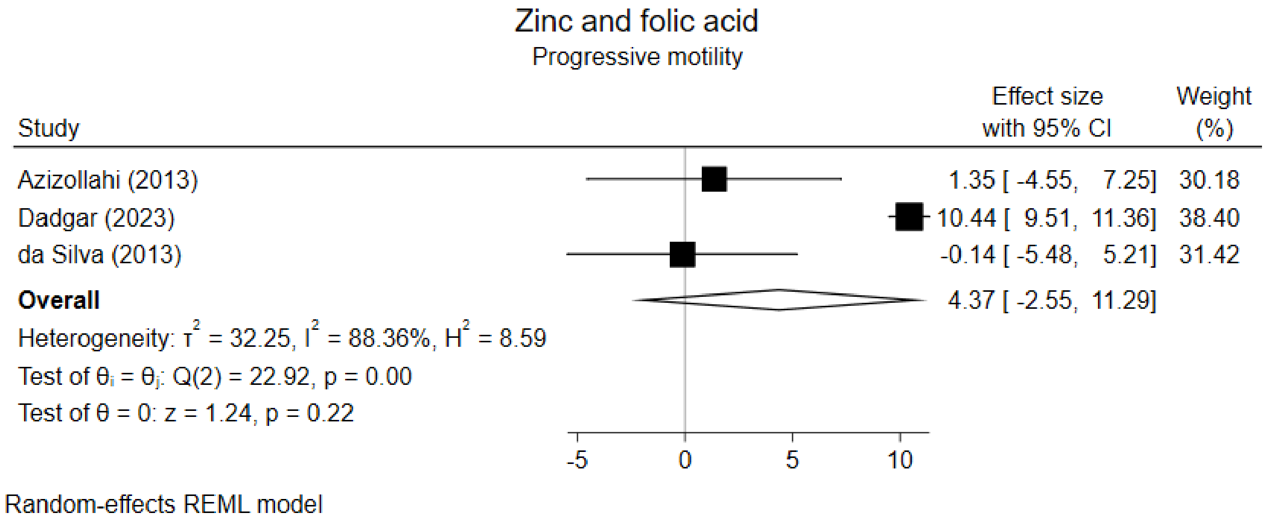

Figure S50. Forest plot of secondary analysis on the effect of zinc and folic acid on progressive motility. The analysis is adjusted for baseline using pseudo-individual participant data.

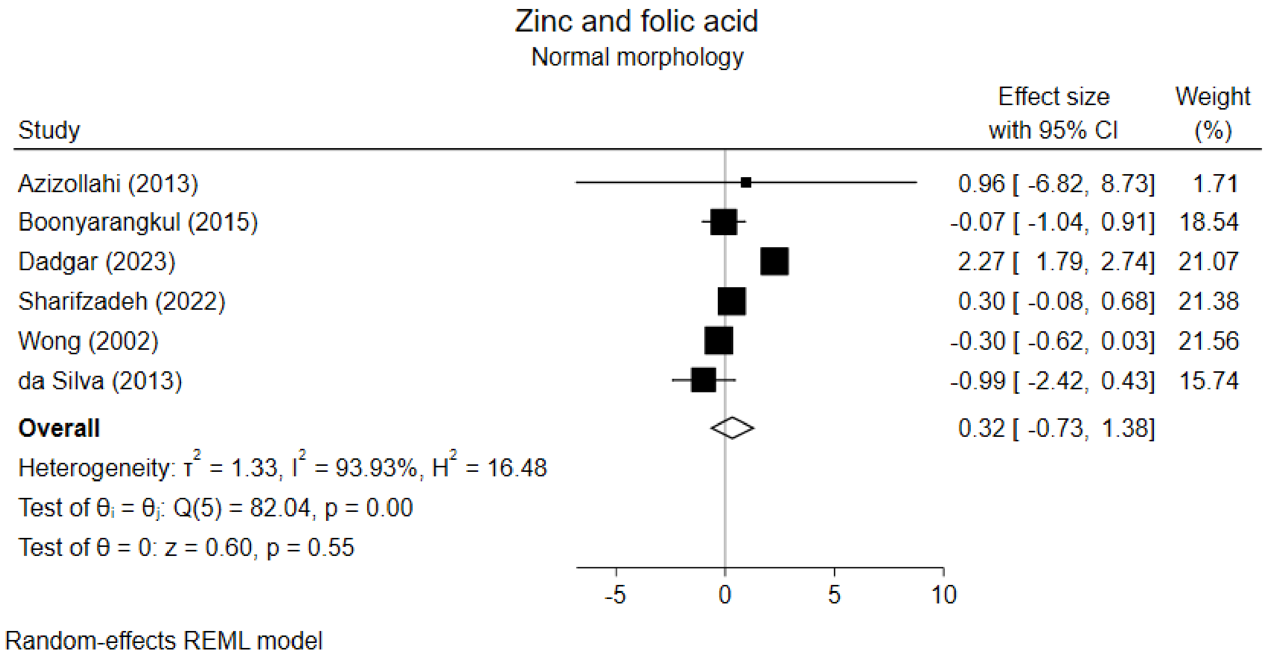

Figure S51. Forest plot of secondary analysis on the effect of zinc and folic acid on normal morphology. The analysis is adjusted for baseline using pseudo-individual participant data.

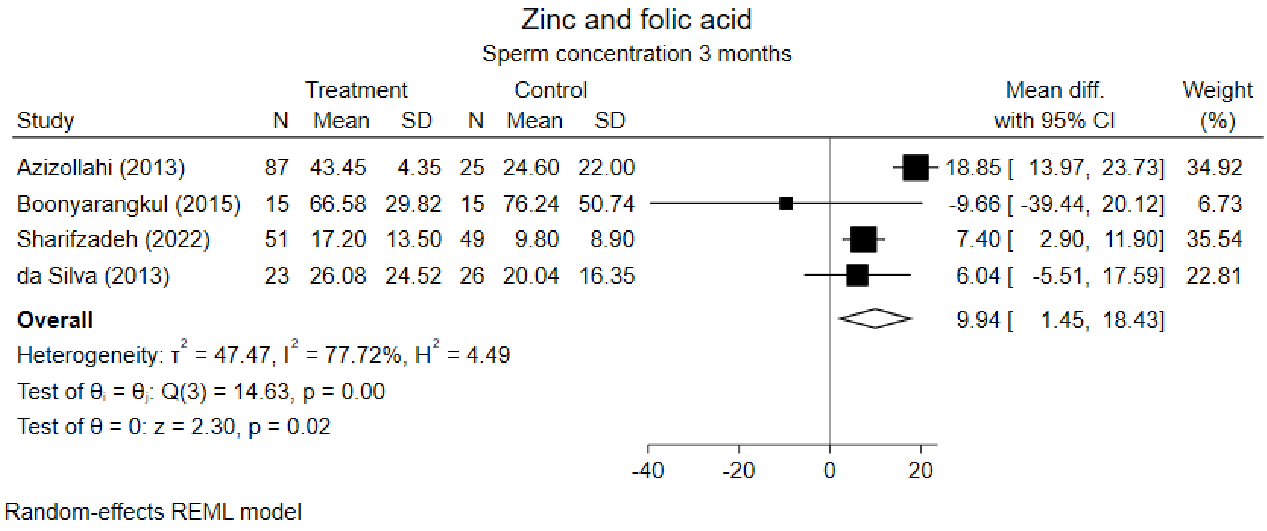

Figure S52. Forest plot of subgroup analysis on the effect of three months of zinc and folic acid use on sperm concentration.

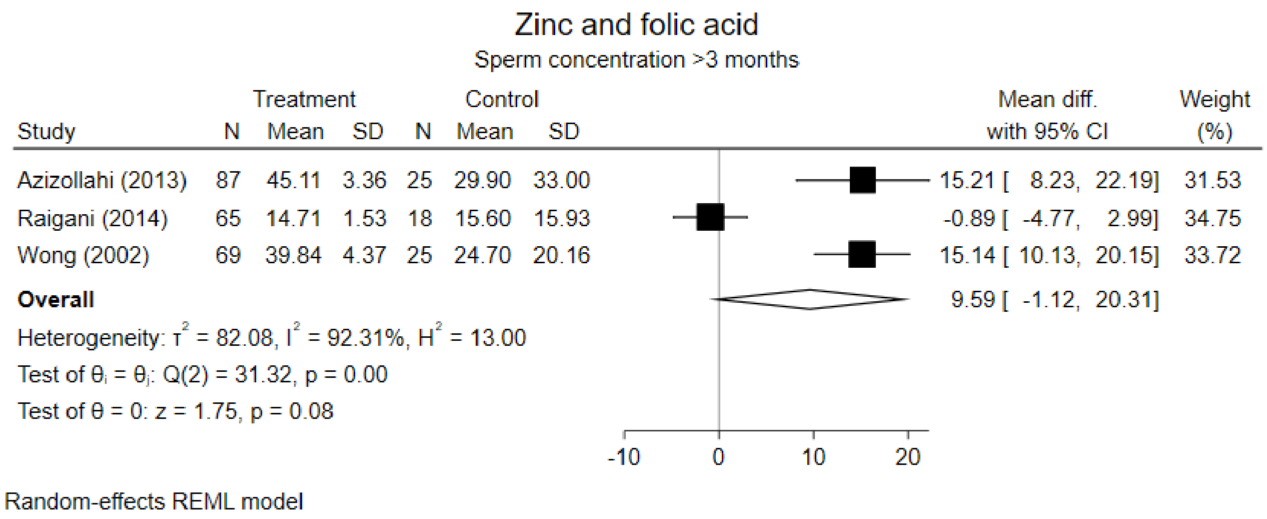

Figure S53. Forest plot of subgroup analysis on the effect of more than three months of zinc and folic acid use on sperm concentration.

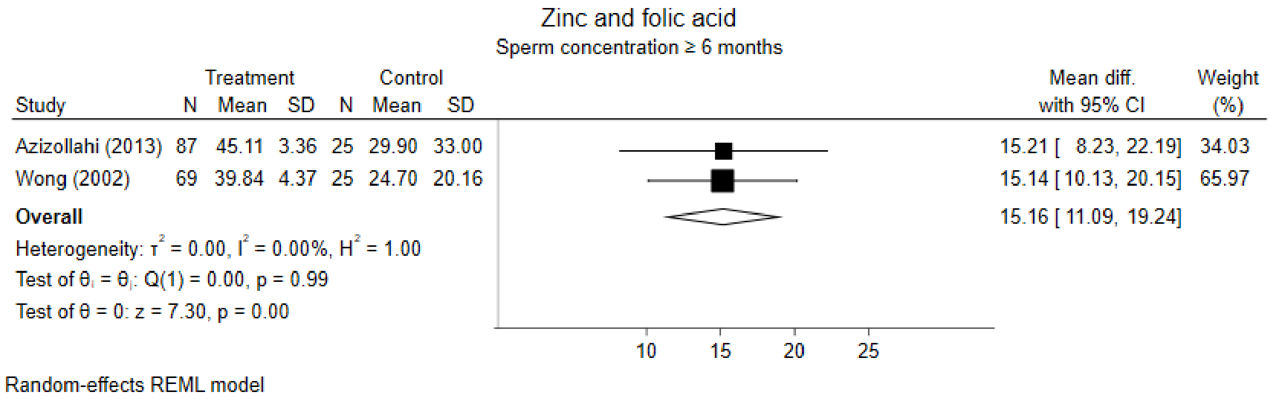

Figure S54. Forest plot of subgroup analysis on the effect of six or more months of zinc and folic acid use on sperm concentration.

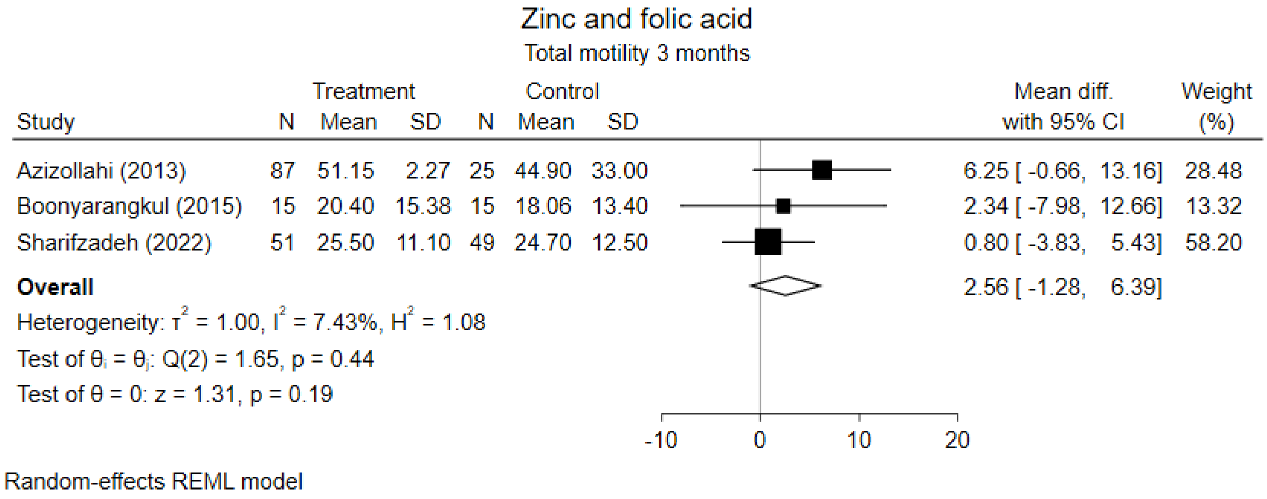

Figure S55. Forest plot of subgroup analysis on the effect of three months of zinc and folic acid use on total motility.

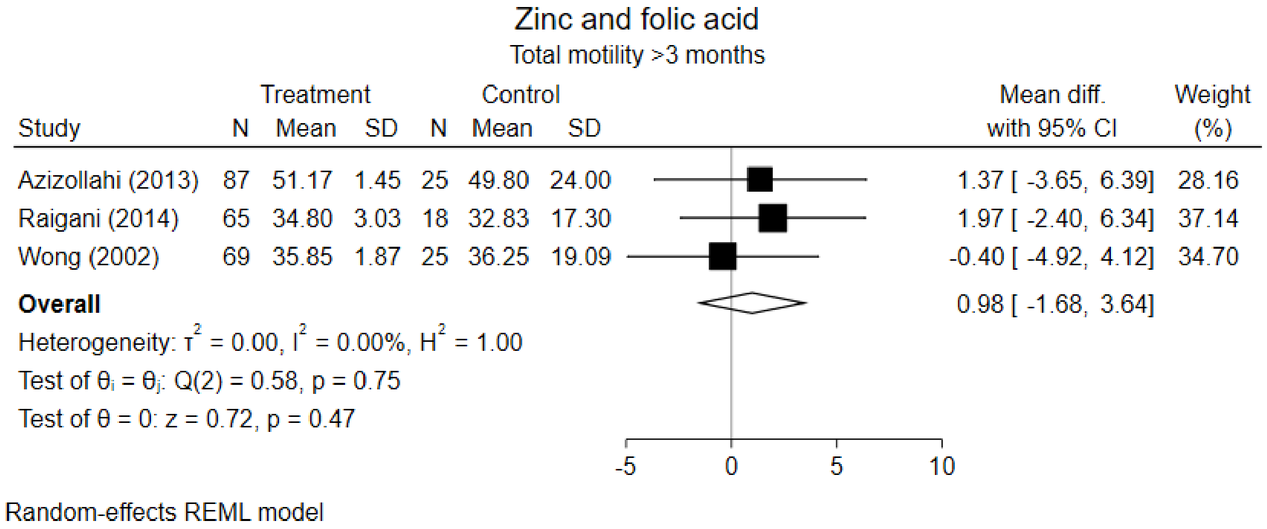

Figure S56. Forest plot of subgroup analysis on the effect of more than three months of zinc and folic acid use on total motility.

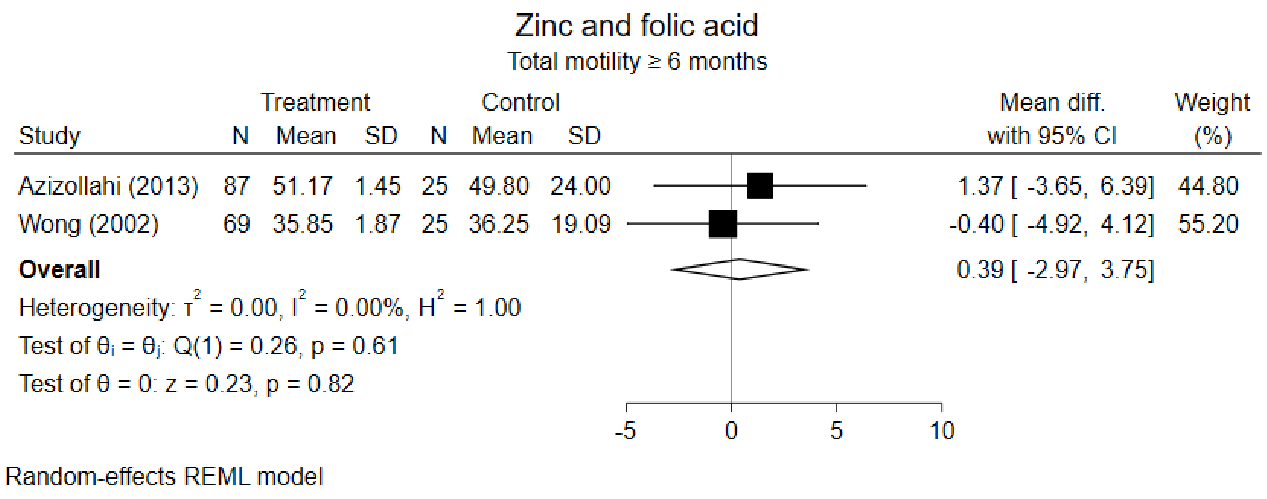

Figure S57. Forest plot of subgroup analysis on the effect of six or more months of zinc and folic acid use on total motility.

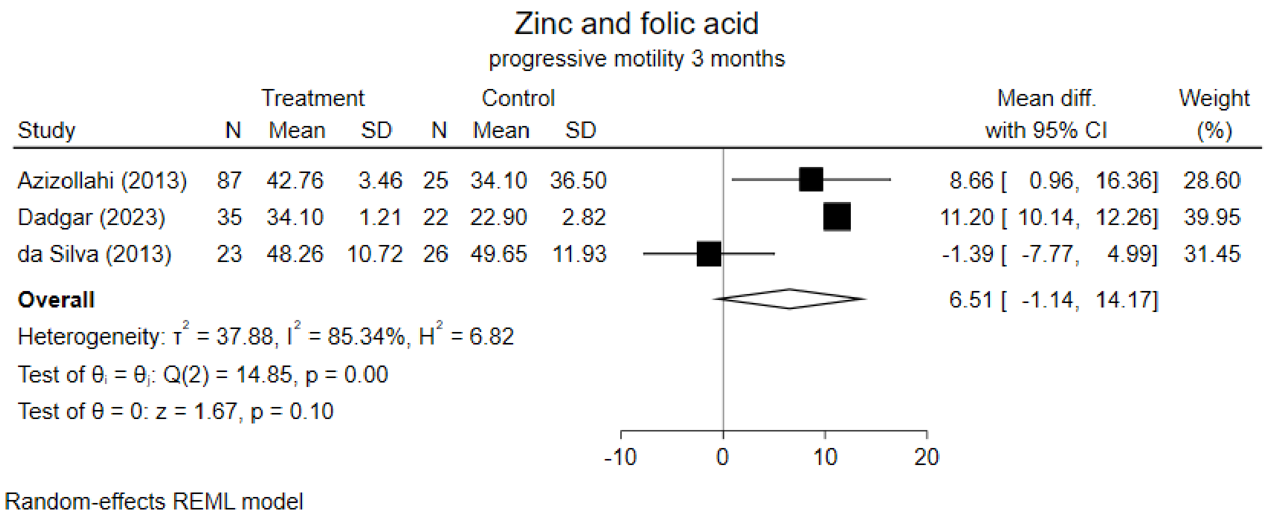

Figure S58. Forest plot of subgroup analysis on the effect of three months of zinc and folic acid use on progressive motility.

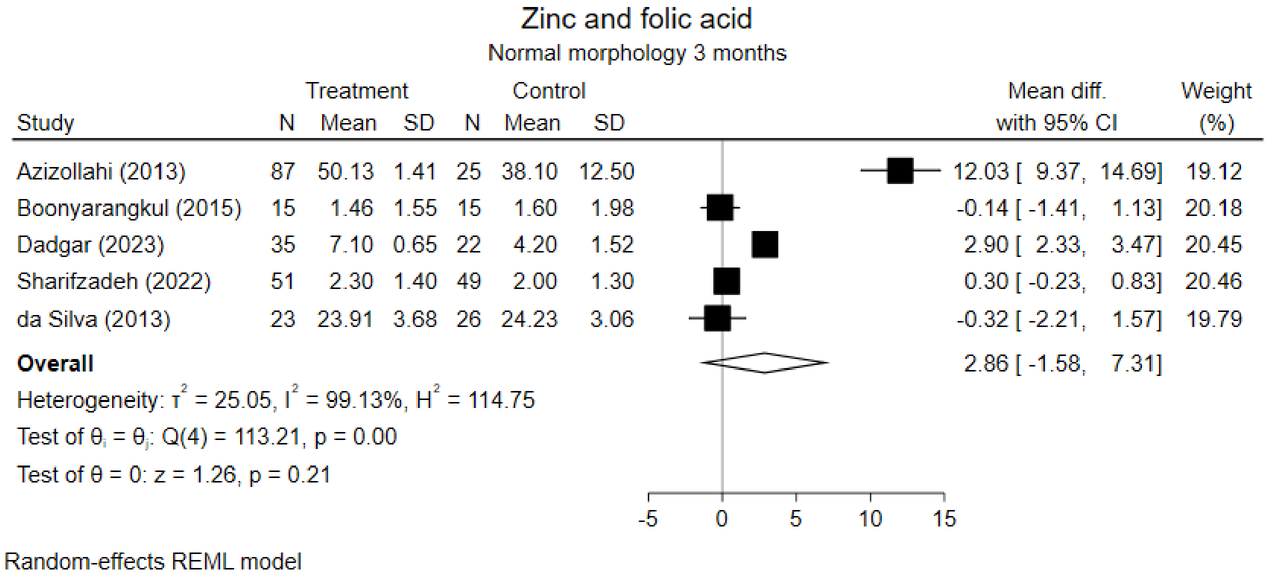

Figure S59. Forest plot of subgroup analysis on the effect of three months of zinc and folic acid use on normal morphology.

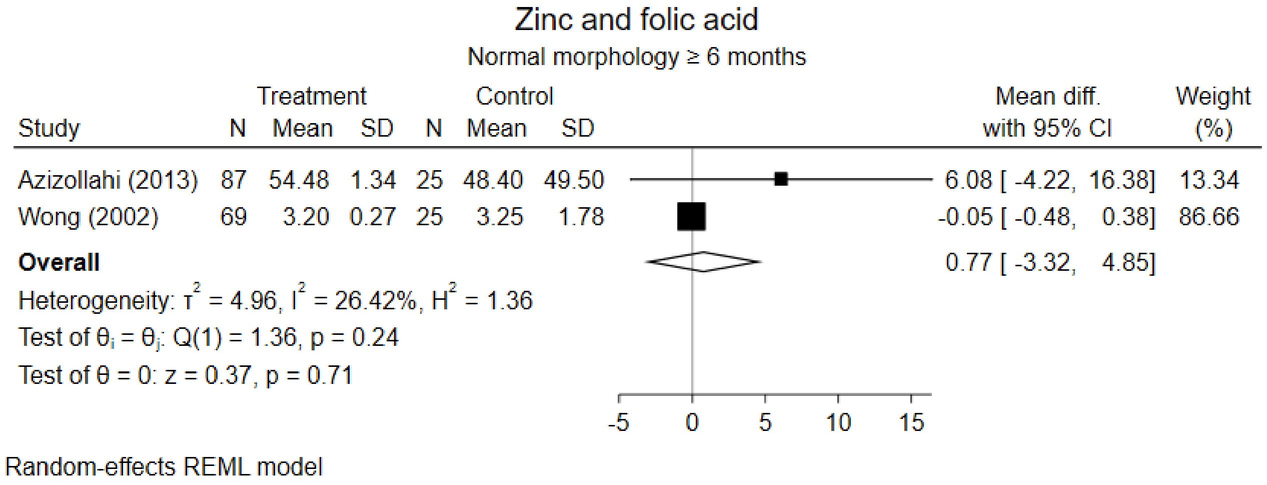

Figure S60. Forest plot of subgroup analysis on the effect of six or more months of zinc and folic acid use on normal morphology.

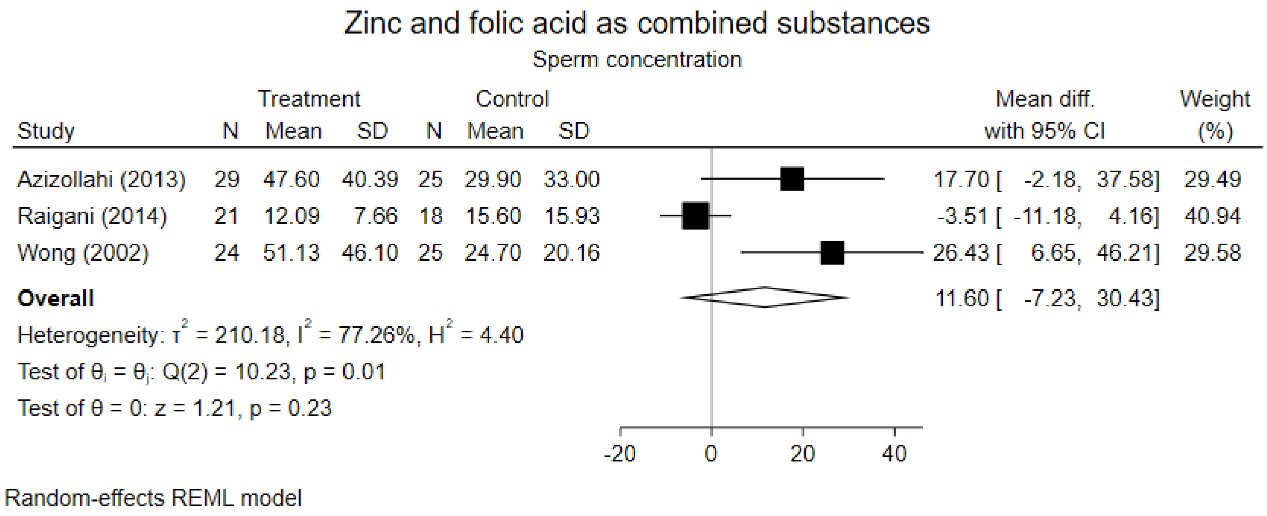

Figure S61. Forest plot of subgroup analysis on the effect of zinc and folic acid as combined substances on sperm concentration.

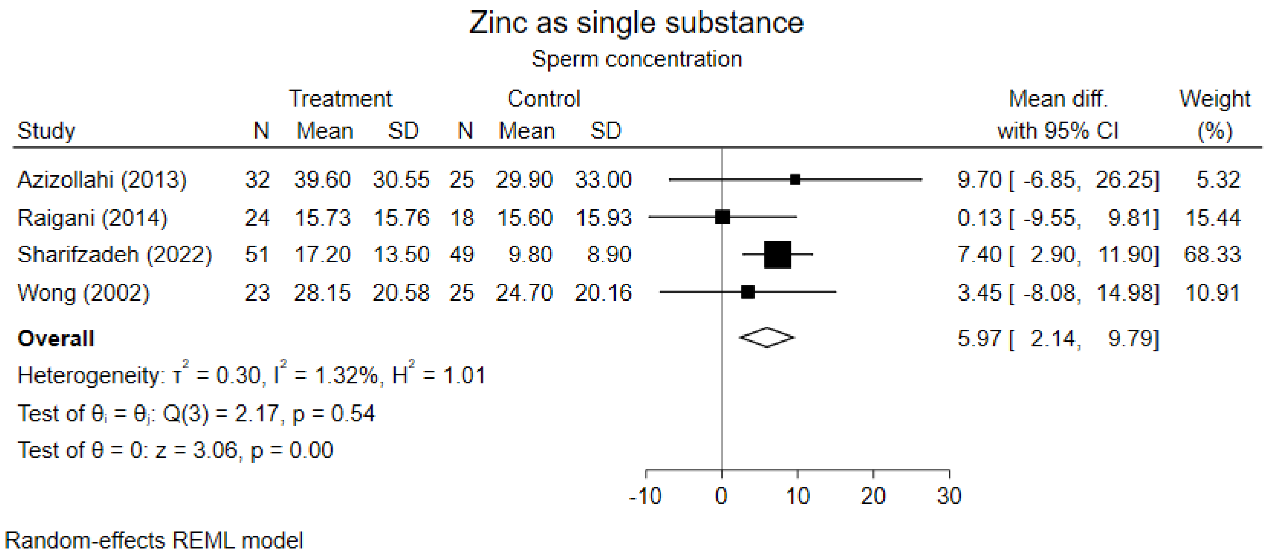

Figure S62. Forest plot of subgroup analysis on the effect of zinc as a single substance on sperm concentration.

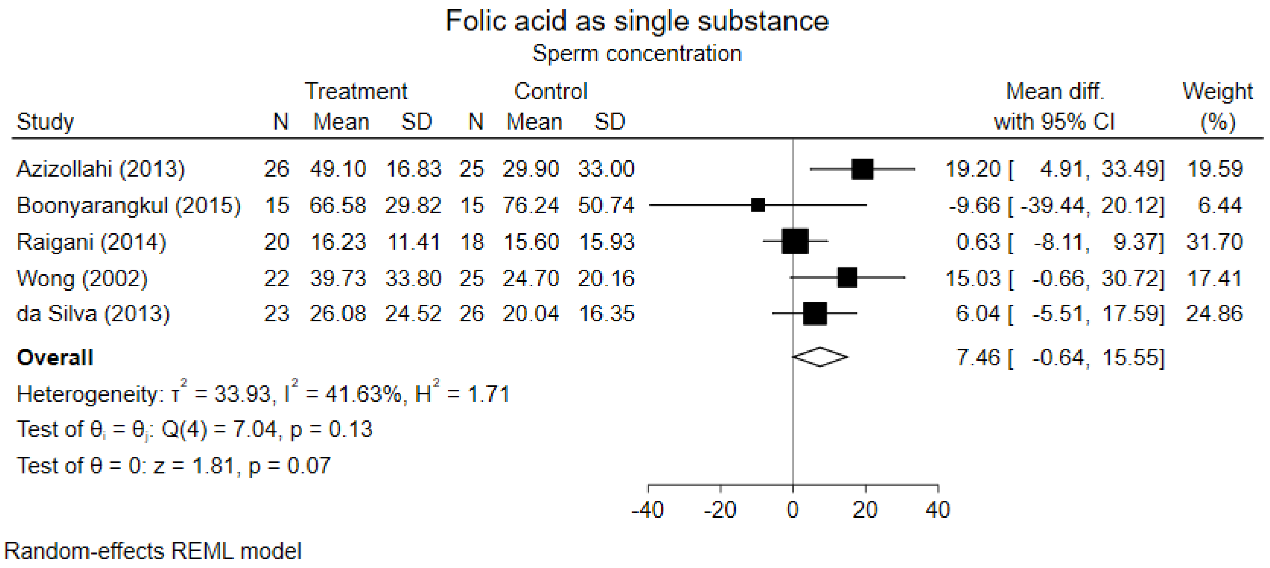

Figure S63. Forest plot of subgroup analysis on the effect of folic acid as a single substance on sperm concentration.

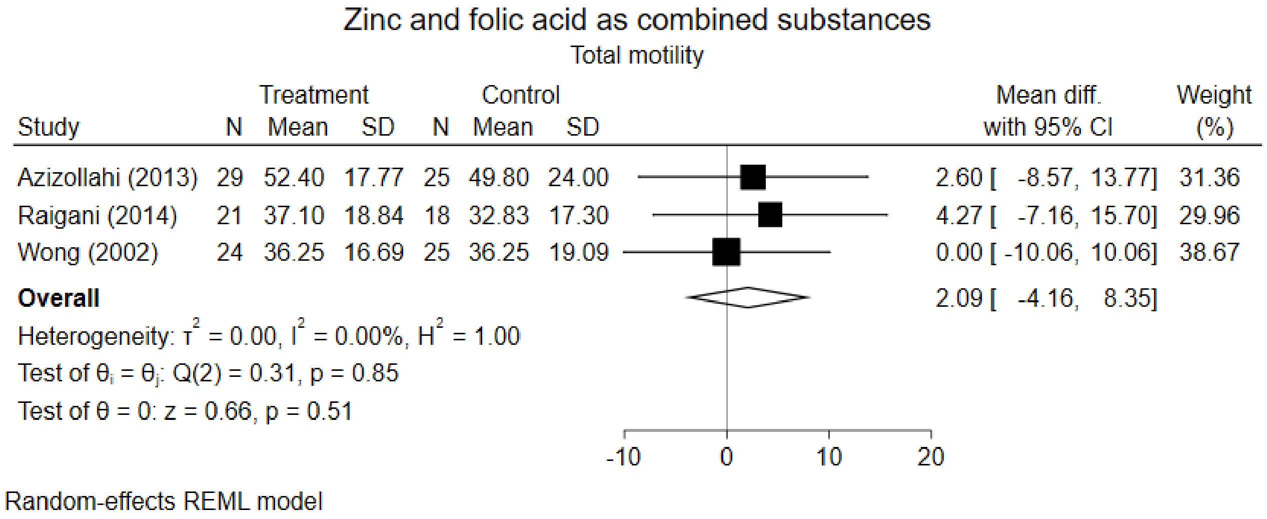

Figure S64. Forest plot of subgroup analysis on the effect of zinc and folic acid as combined substances on total motility.

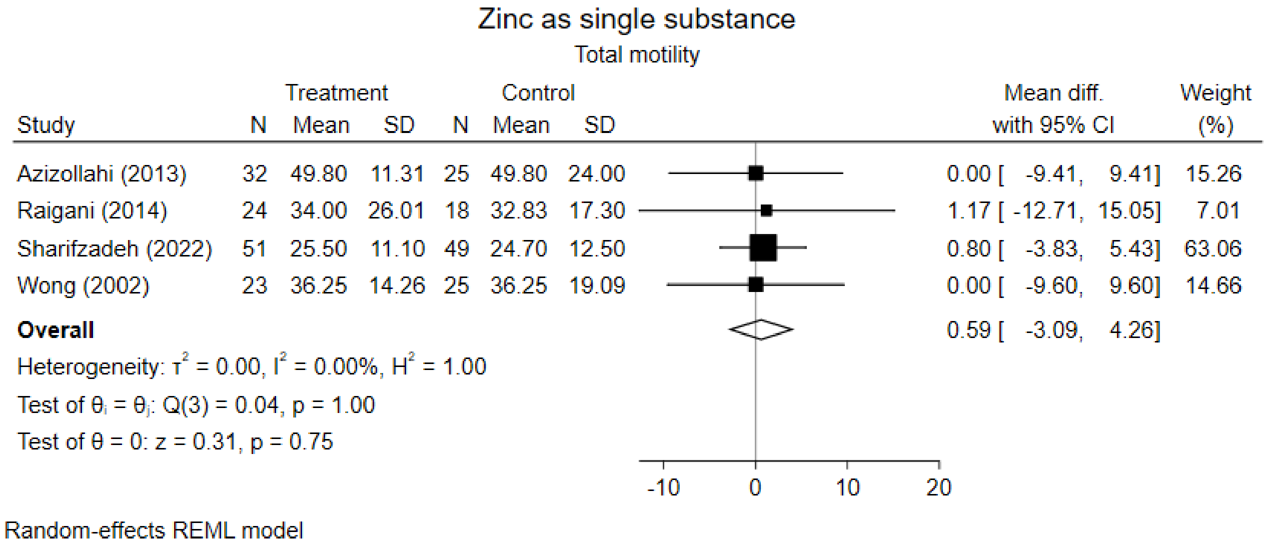

Figure S65. Forest plot of subgroup analysis on the effect of zinc as a single substance on total motility.

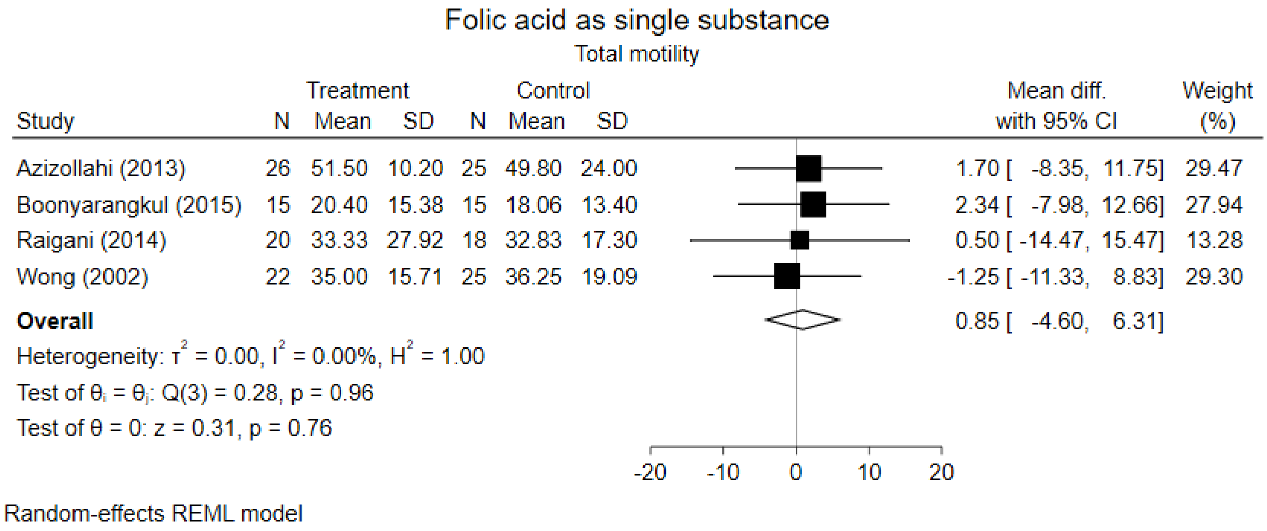

Figure S66. Forest plot of subgroup analysis on the effect of folic acid as a single substance on total motility.

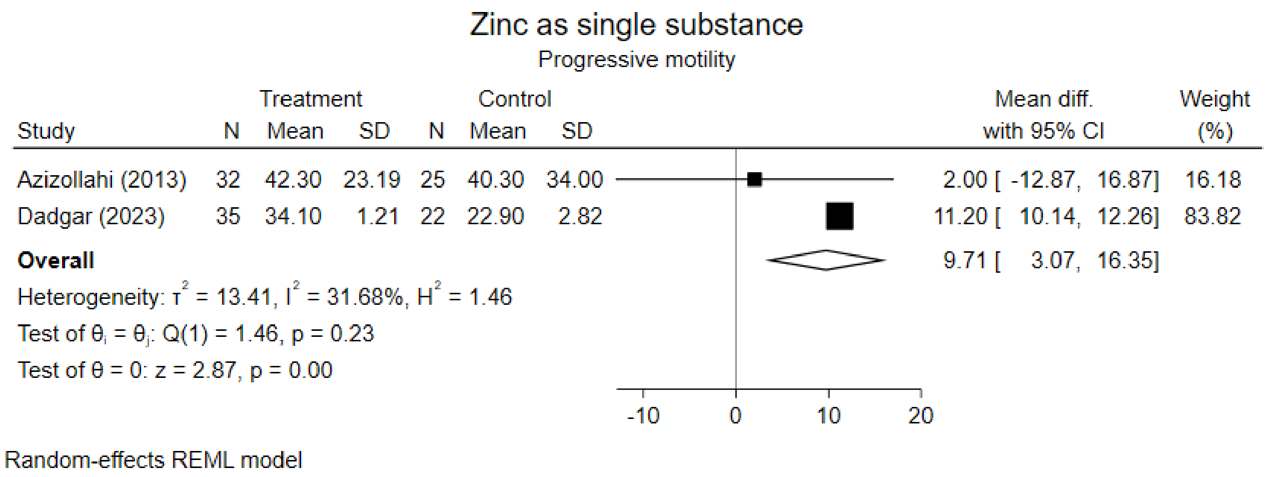

Figure S67. Forest plot of subgroup analysis on the effect of zinc as a single substance on progressive motility.

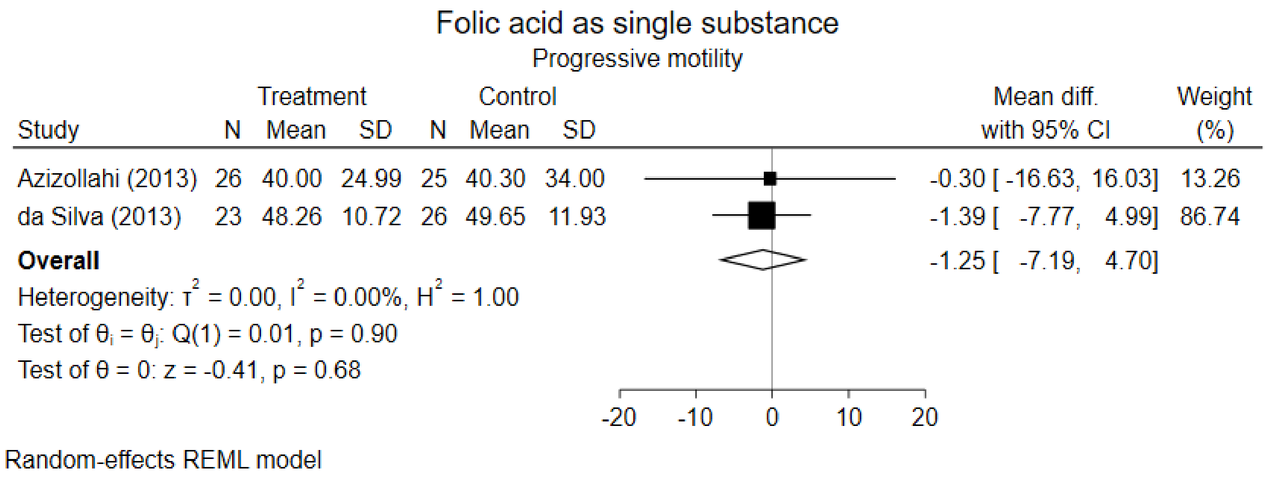

Figure S68. Forest plot of subgroup analysis on the effect of folic acid as a single substance on progressive motility.

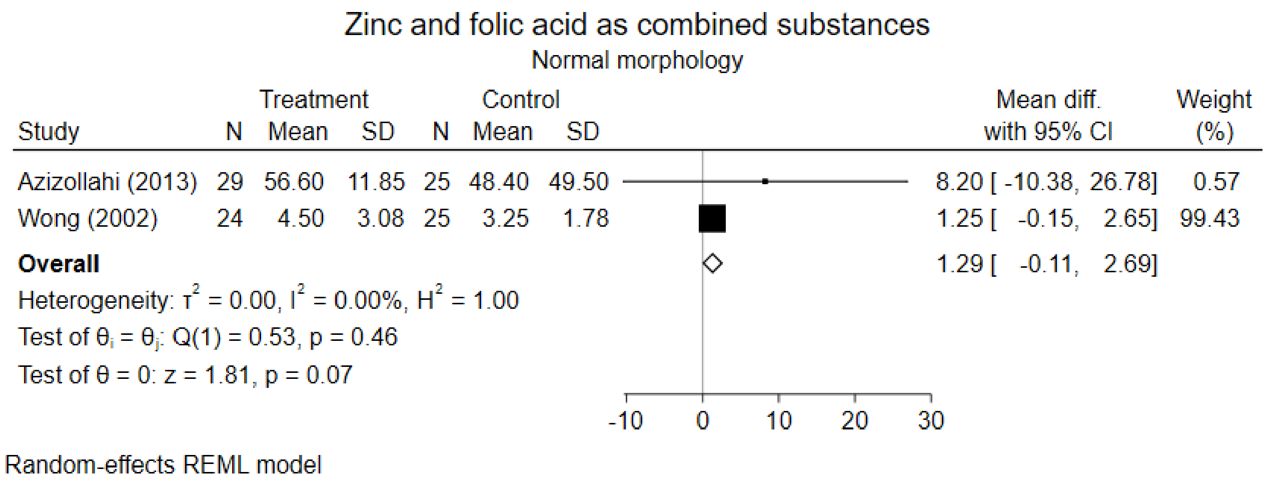

Figure S69. Forest plot of subgroup analysis on the effect of zinc and folic acid as combined substances on normal morphology.

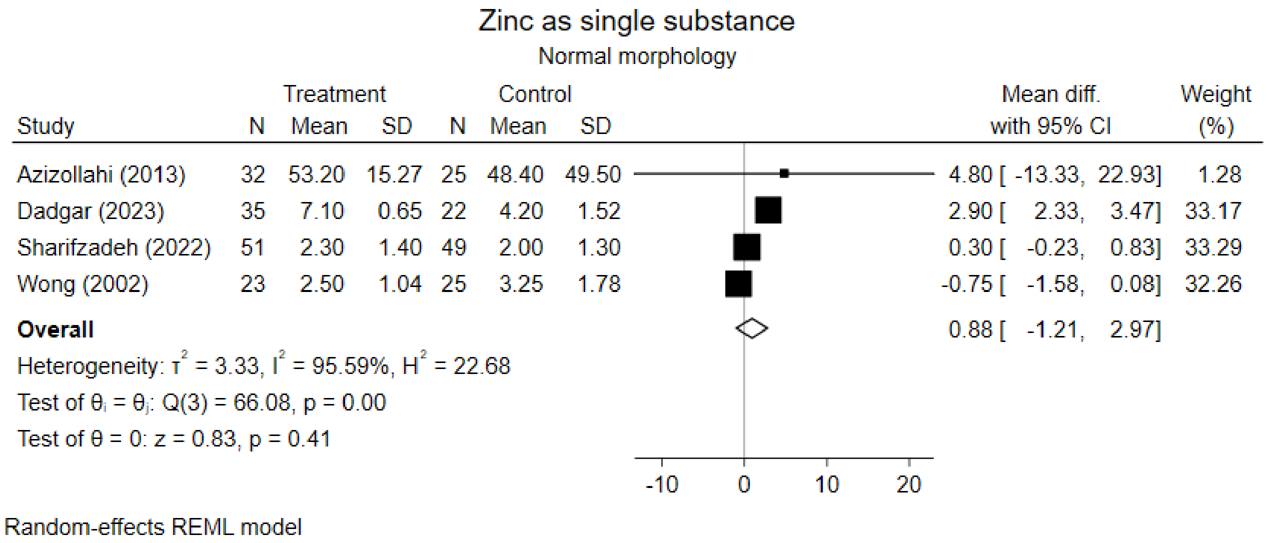

Figure S70. Forest plot of subgroup analysis on the effect of zinc as a single substance on normal morphology.

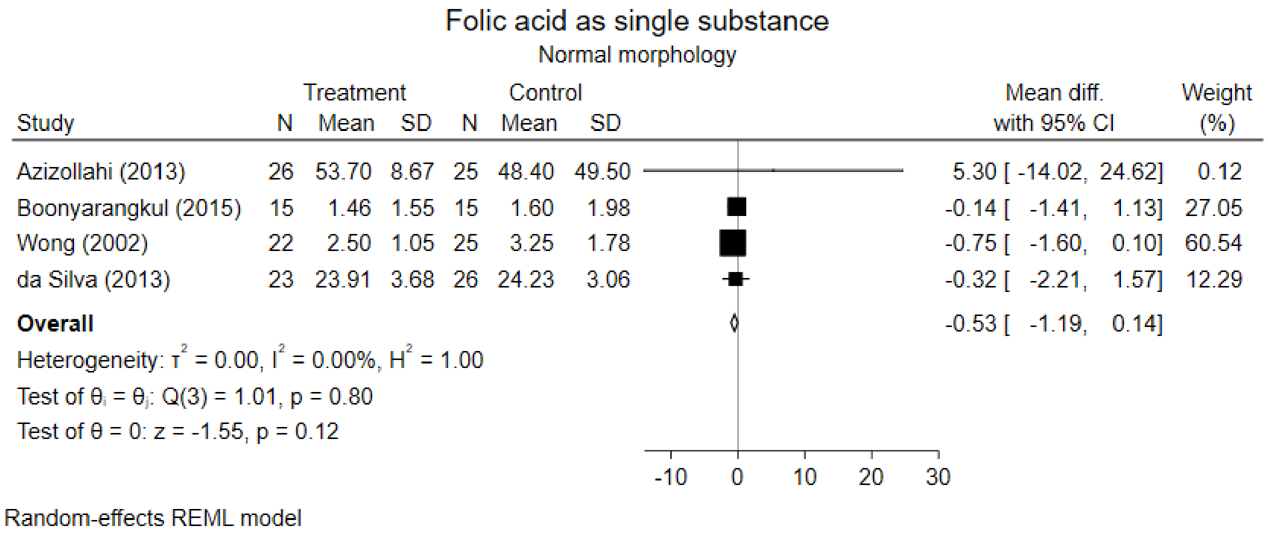

Figure S71. Forest plot of subgroup analysis on the effect of folic acid as a single substance on normal morphology.

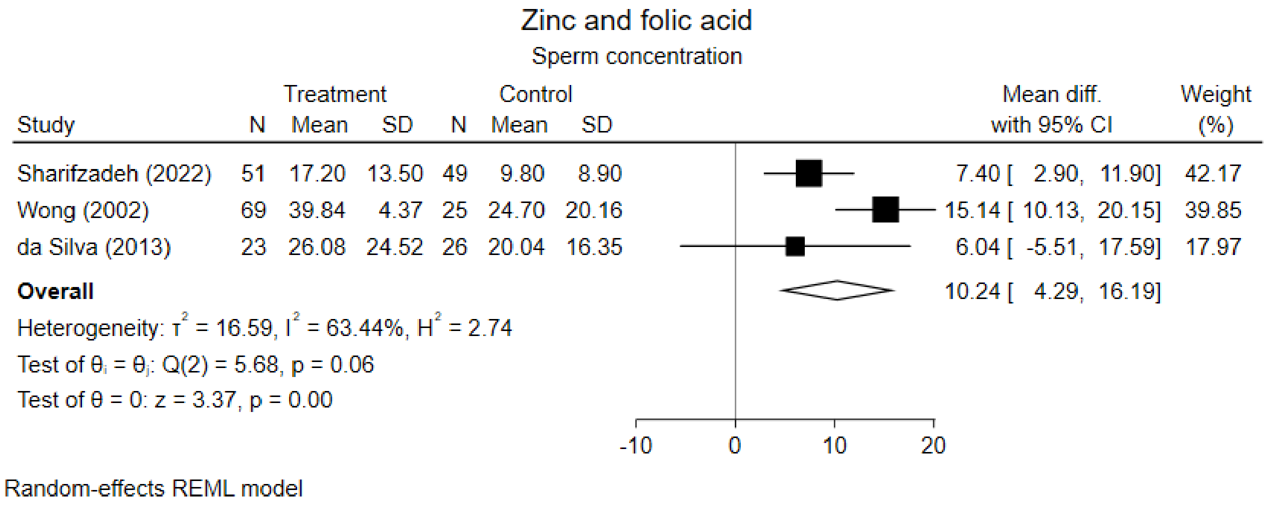

Figure S72. Forest plot on sensitivity analysis on the effect of zinc and folic acid on sperm concentration. Studies evaluated as having a high risk of bias have been excluded from the analysis.

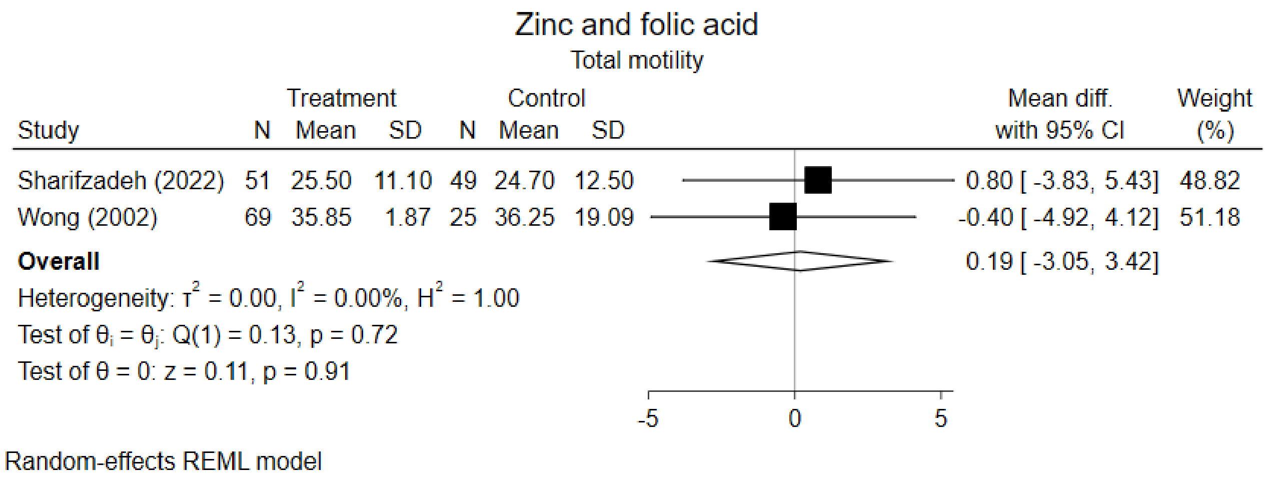

Figure S73. Forest plot on sensitivity analysis on the effect of zinc and folic acid on total motility. Studies evaluated as having a high risk of bias have been excluded from the analysis.

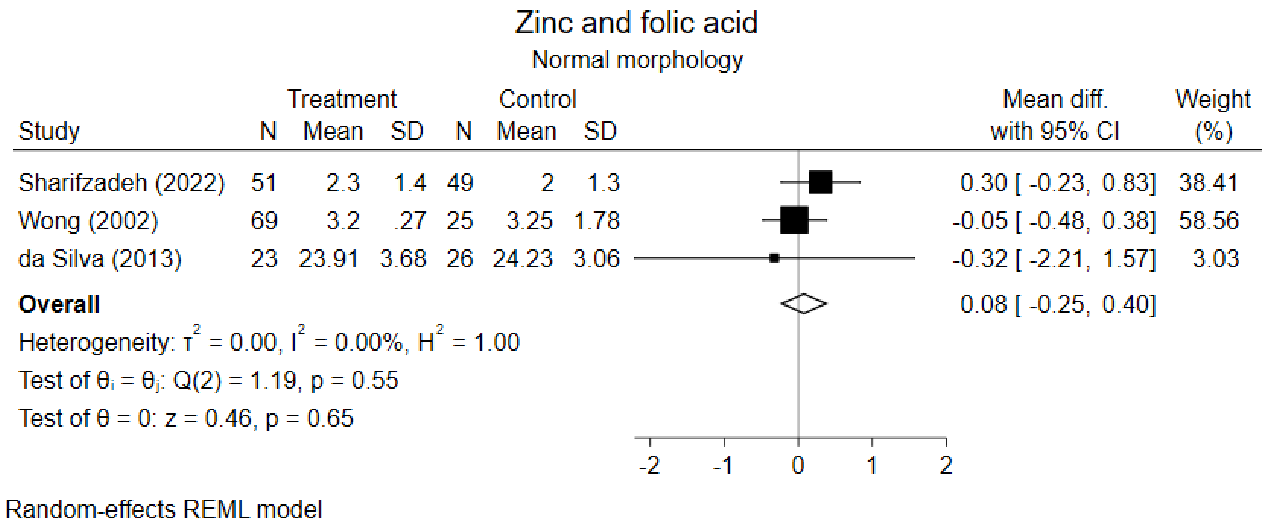

Figure S74. Forest plot on sensitivity analysis on the effect of zinc and folic acid on normal morphology. Studies evaluated as having a high risk of bias have been excluded from the analysis.

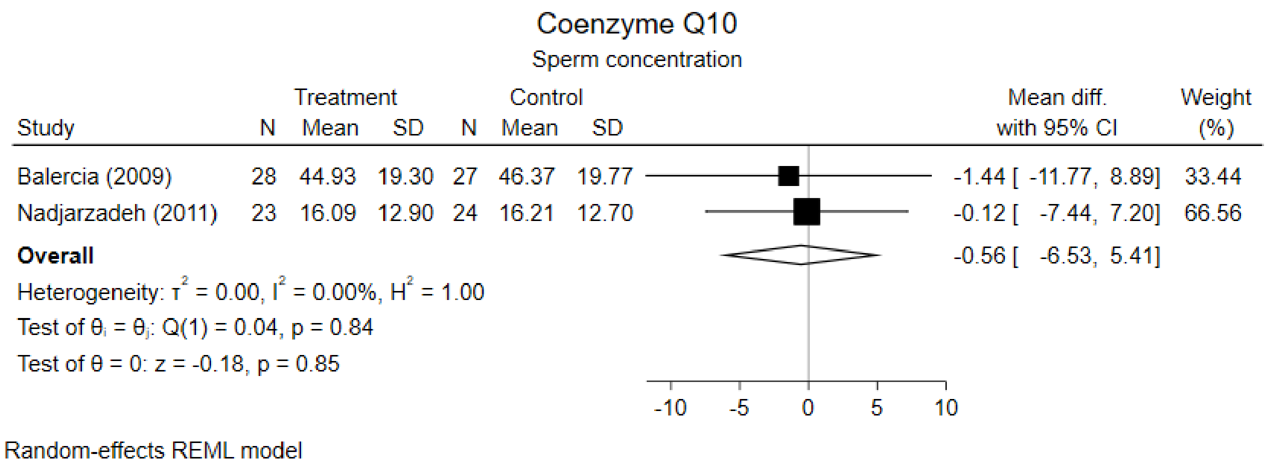

Figure S75. Forest plot of primary analysis on the effect of coenzyme Q10 on sperm concentration.

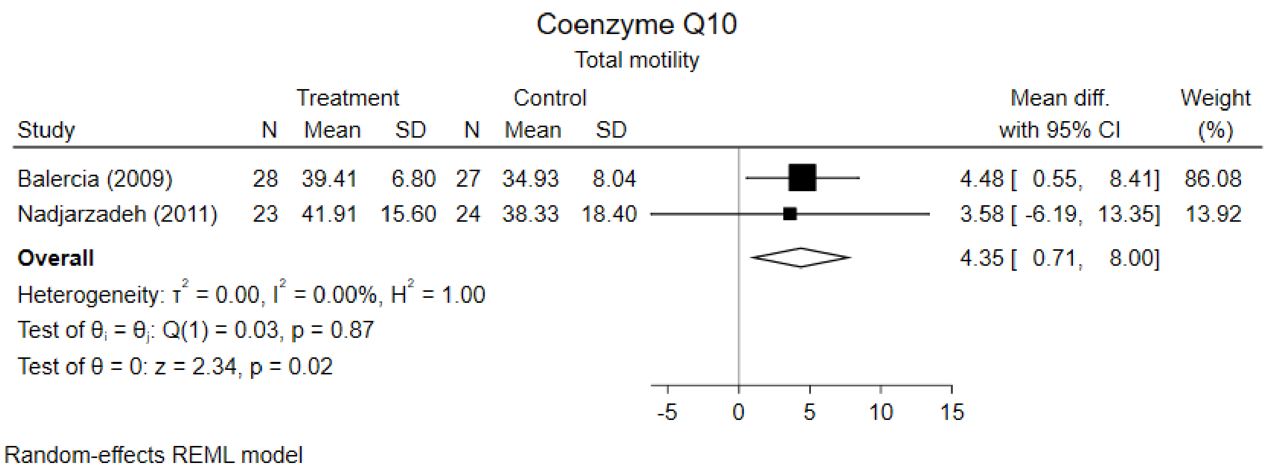

Figure S76. Forest plot of primary analysis on the effect of coenzyme Q10 on total motility.

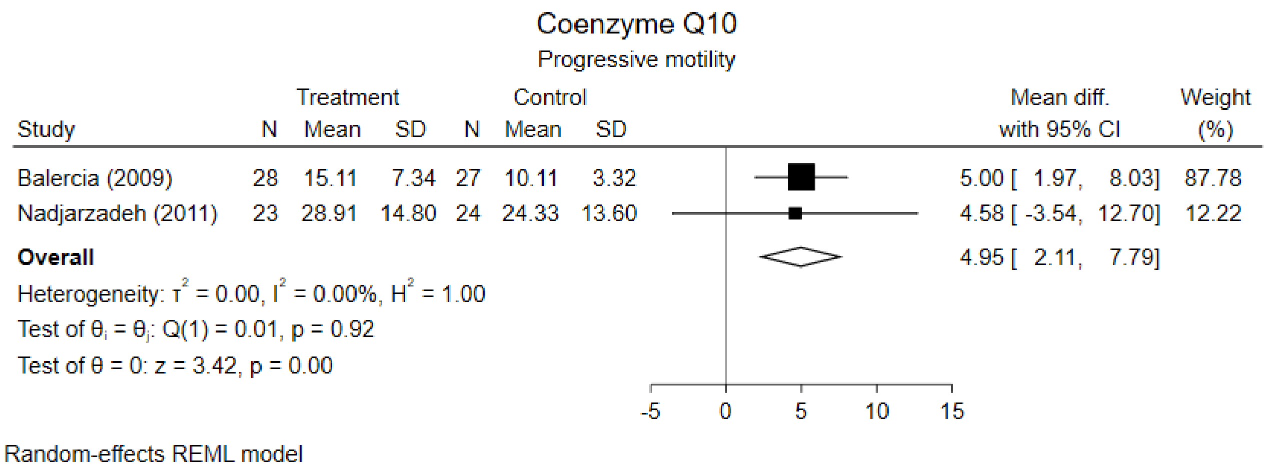

Figure S77. Forest plot of primary analysis on the effect of coenzyme Q10 on progressive motility.

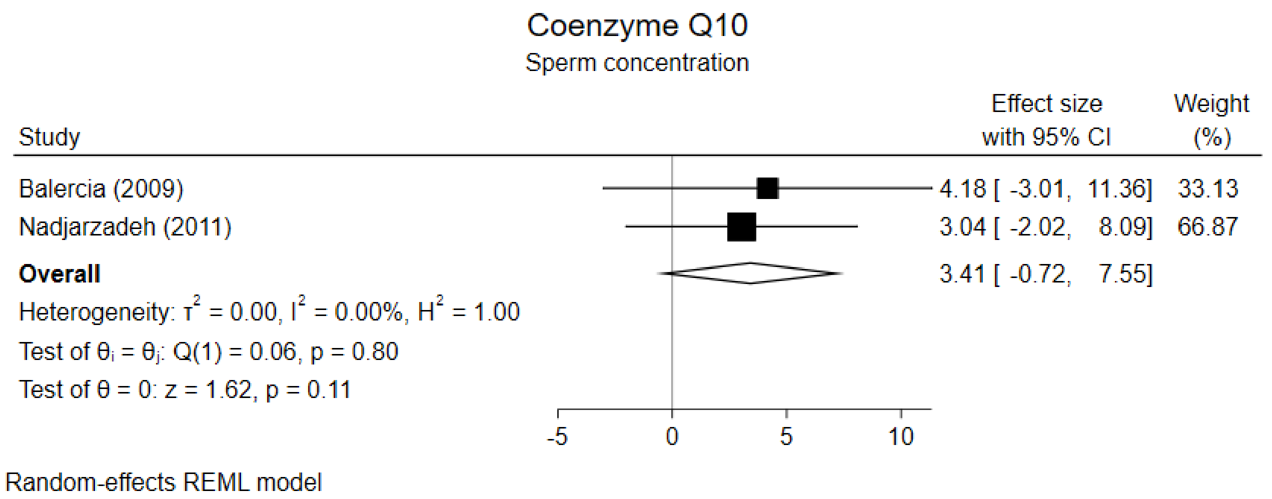

Figure S78. Forest plot of secondary analysis on the effect of coenzyme Q10 on sperm concentration. The analysis is adjusted for baseline using pseudo-individual participant data.

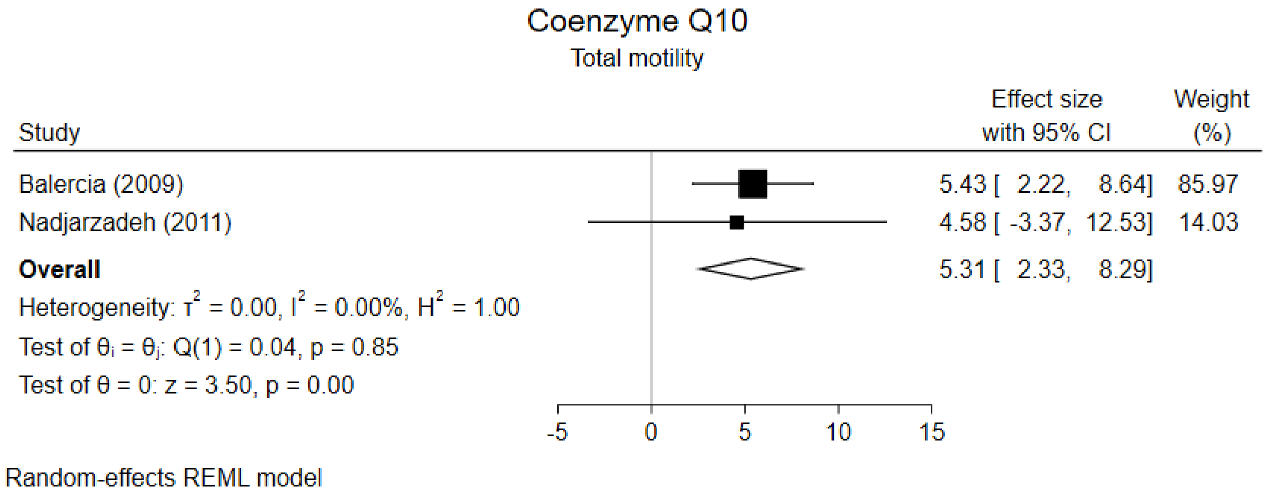

Figure S79. Forest plot of secondary analysis on the effect of coenzyme Q10 on total motility. The analysis is adjusted for baseline using pseudo-individual participant data.

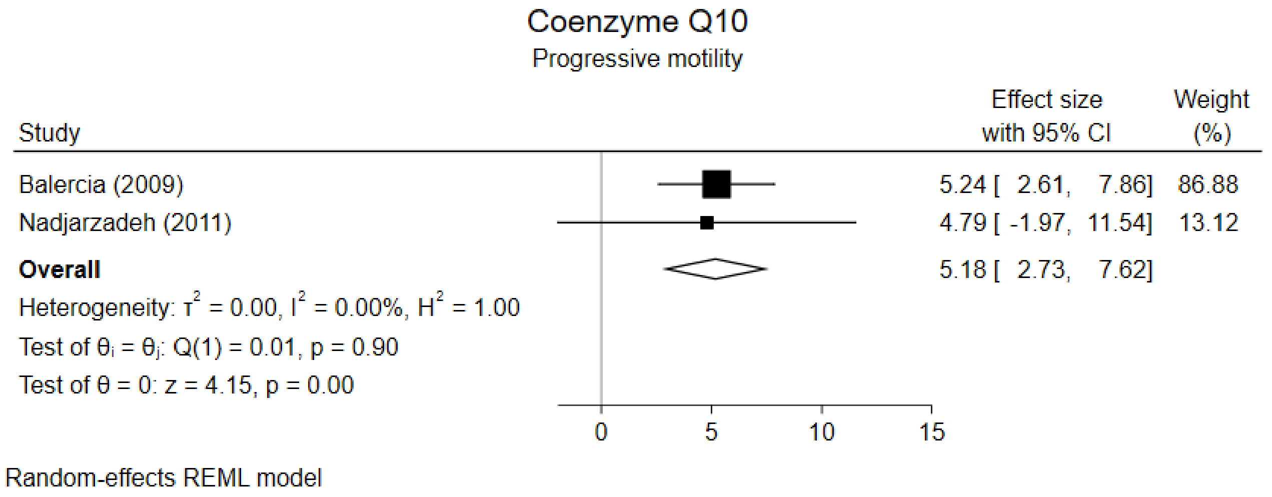

Figure S80. Forest plot of secondary analysis on the effect of coenzyme Q10 on progressive motility. The analysis is adjusted for baseline using pseudo-individual participant data.

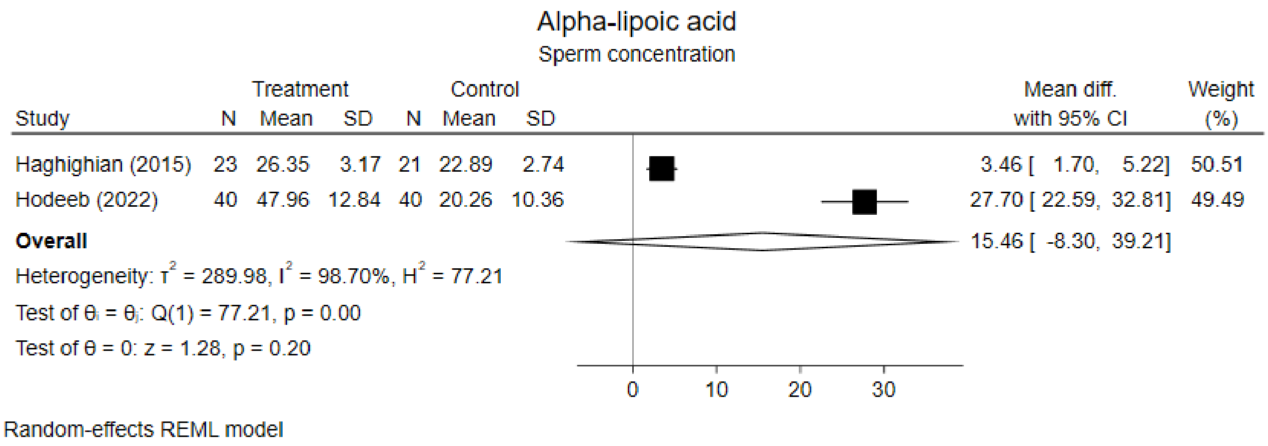

Figure S81. Forest plot of primary analysis on the effect of alpha-lipoic acid on sperm concentration.

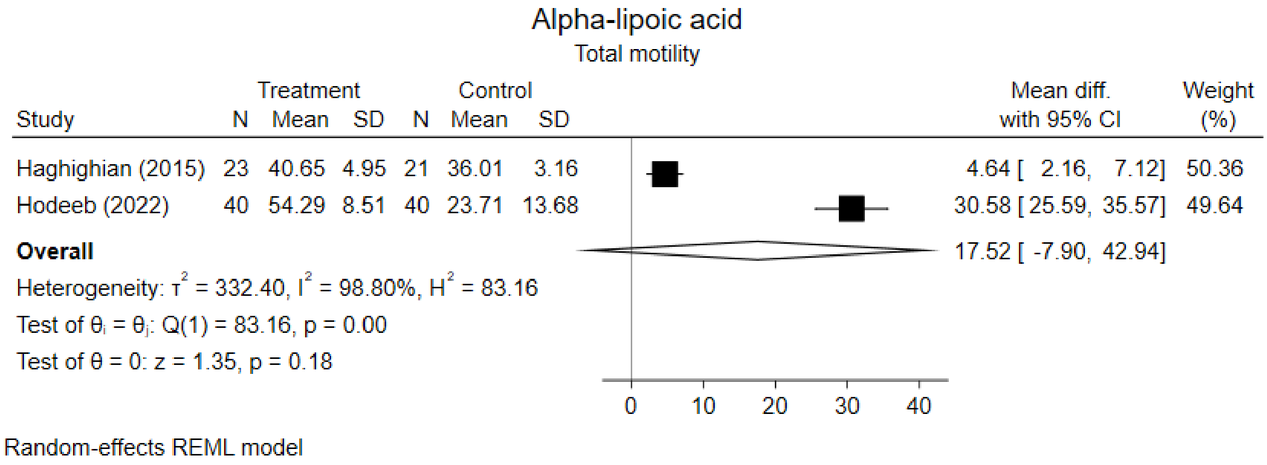

Figure S82. Forest plot of primary analysis on the effect of alpha-lipoic acid on total motility.

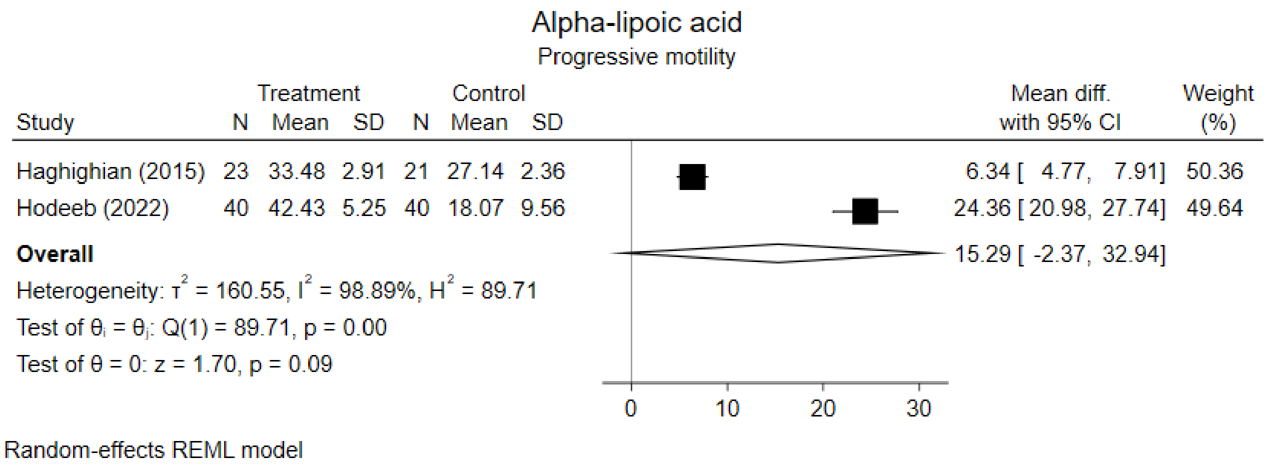

Figure S83. Forest plot of primary analysis on the effect of alpha-lipoic acid on progressive motility.

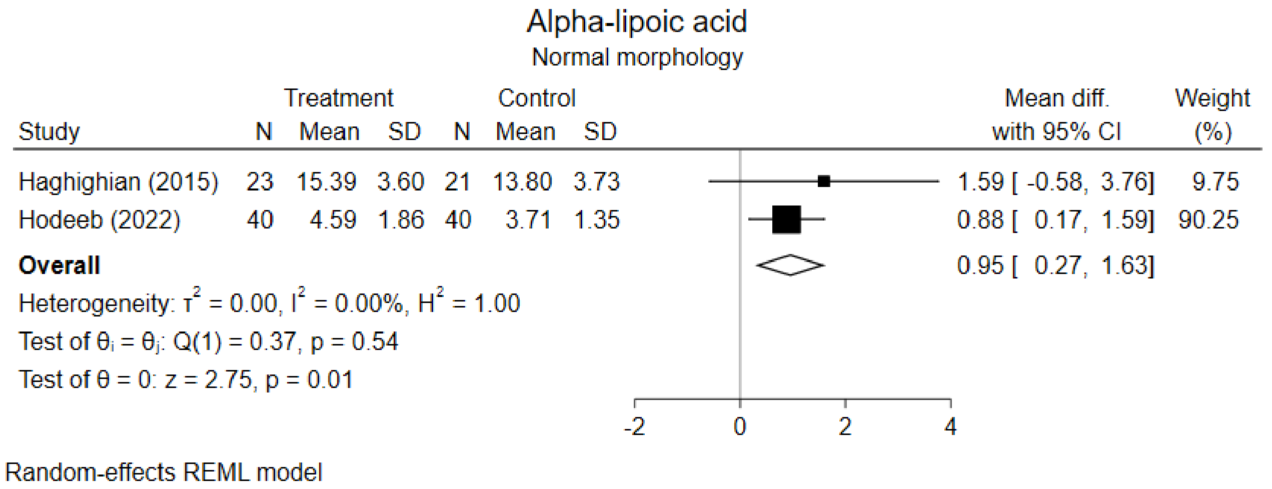

Figure S84. Forest plot of primary analysis on the effect of alpha-lipoic acid on normal morphology.

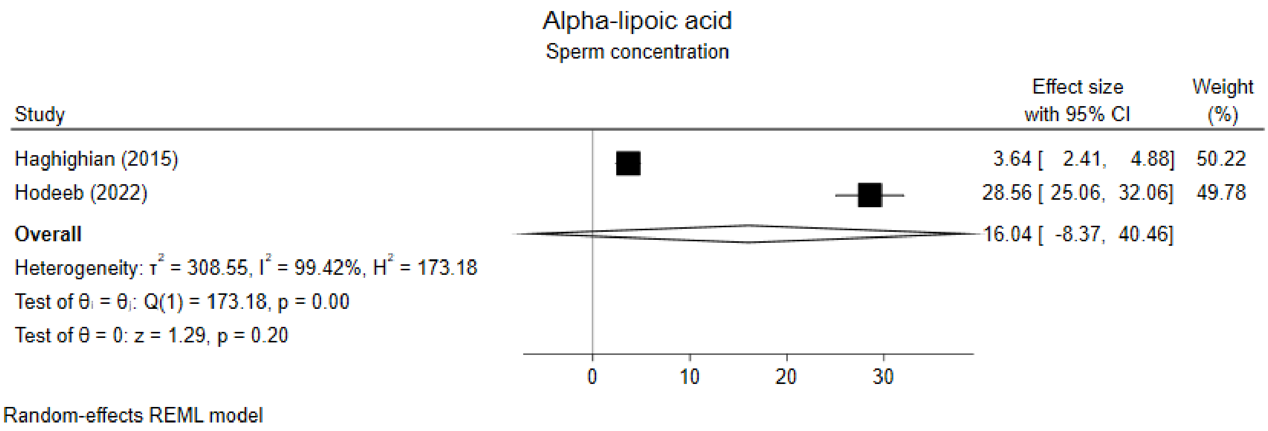

Figure S85. Forest plot of secondary analysis on the effect of alpha-lipoic acid on sperm concentration. The analysis is adjusted for baseline using pseudo-individual participant data.

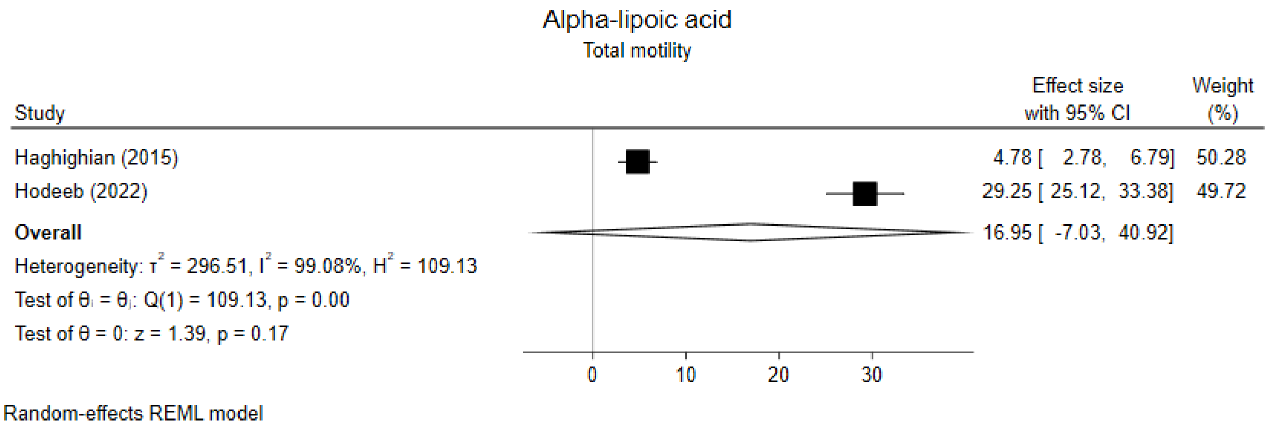

Figure S86. Forest plot of secondary analysis on the effect of alpha-lipoic acid on total motility. The analysis is adjusted for baseline using pseudo-individual participant data.

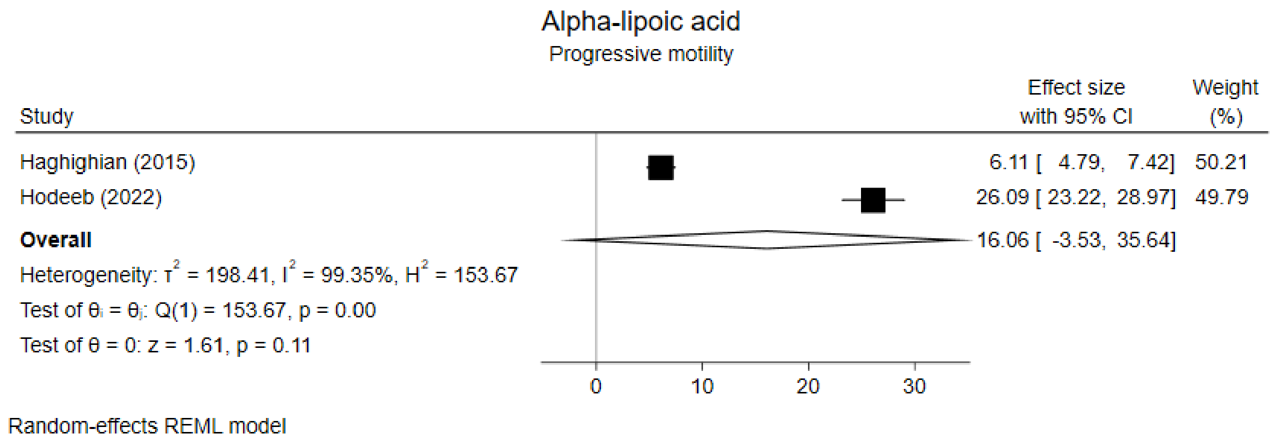

Figure S87. Forest plot of secondary analysis on the effect of alpha-lipoic acid on progressive motility. The analysis is adjusted for baseline using pseudo-individual participant data.

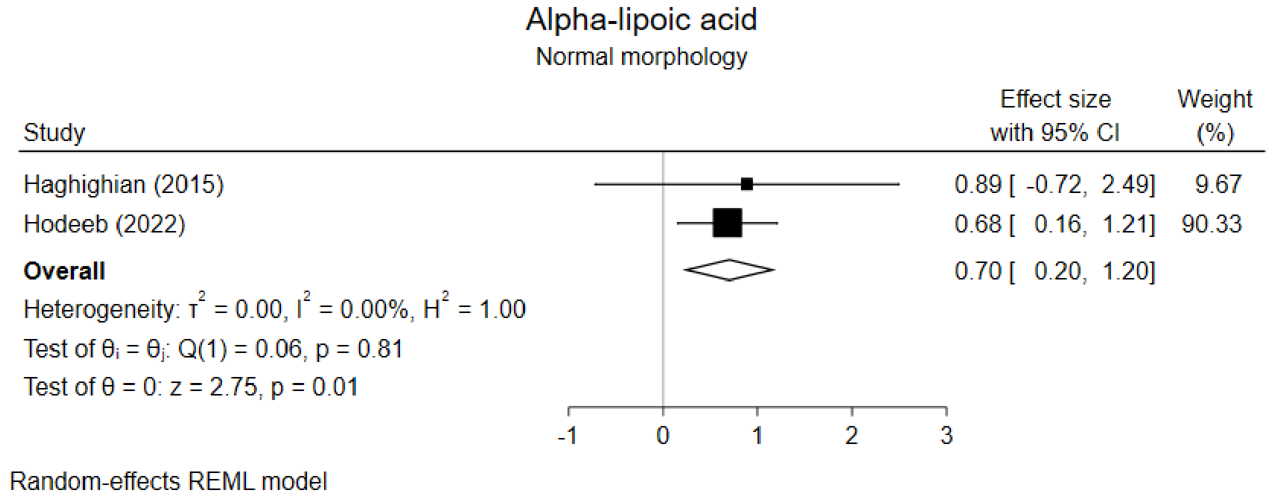

Figure S88. Forest plot of secondary analysis on the effect of alpha-lipoic acid on normal morphology. The analysis is adjusted for baseline using pseudo-individual participant data.

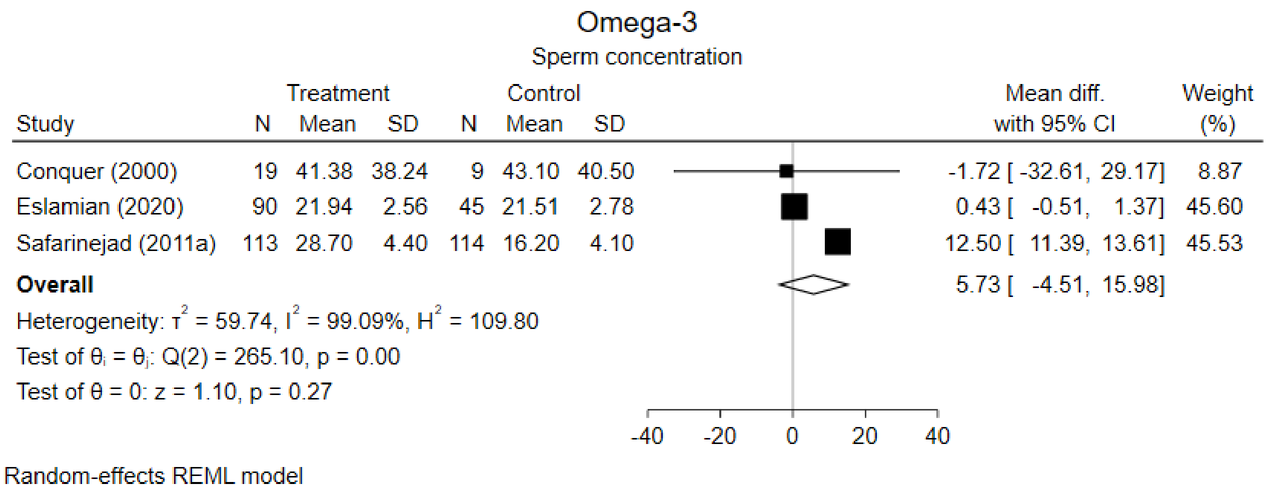

Figure S89. Forest plot of primary analysis on the effect of omega-3 fatty acids on sperm concentration.

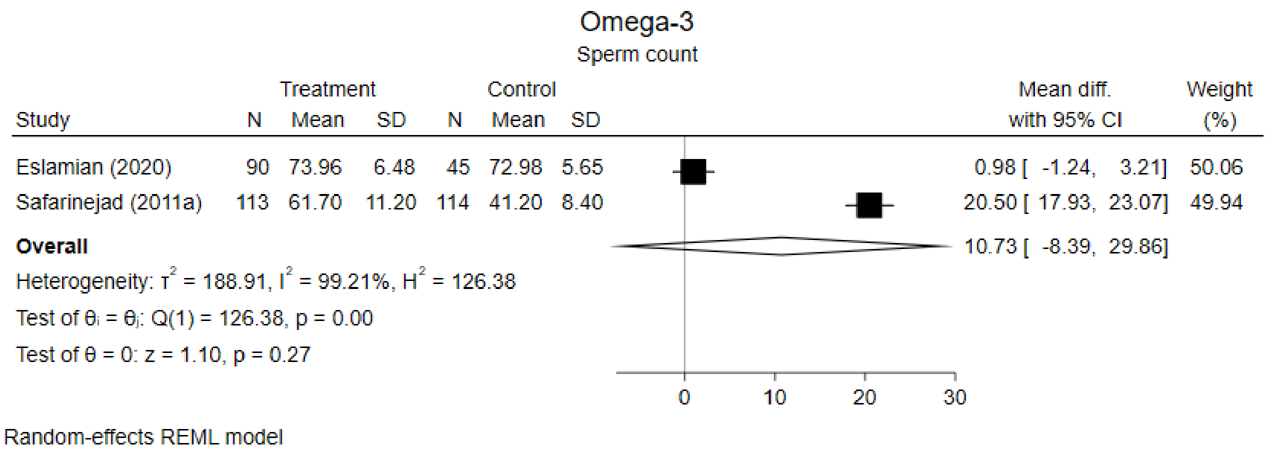

Figure S90. Forest plot of primary analysis on the effect of omega-3 fatty acids on sperm count.

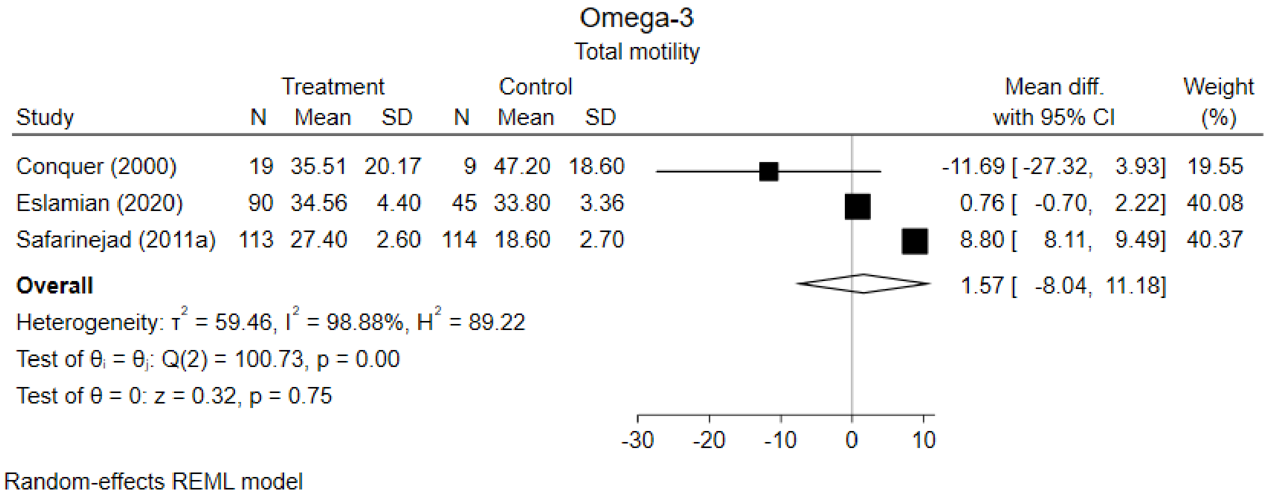

Figure S91. Forest plot of primary analysis on the effect of omega-3 fatty acids on total motility.

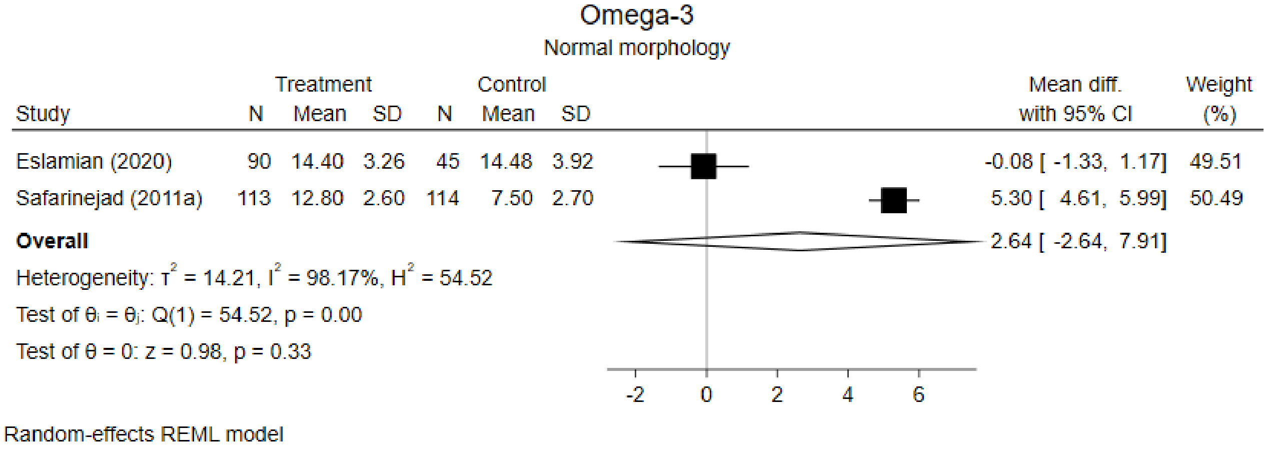

Figure S92. Forest plot of primary analysis on the effect of omega-3 fatty acids on normal morphology.

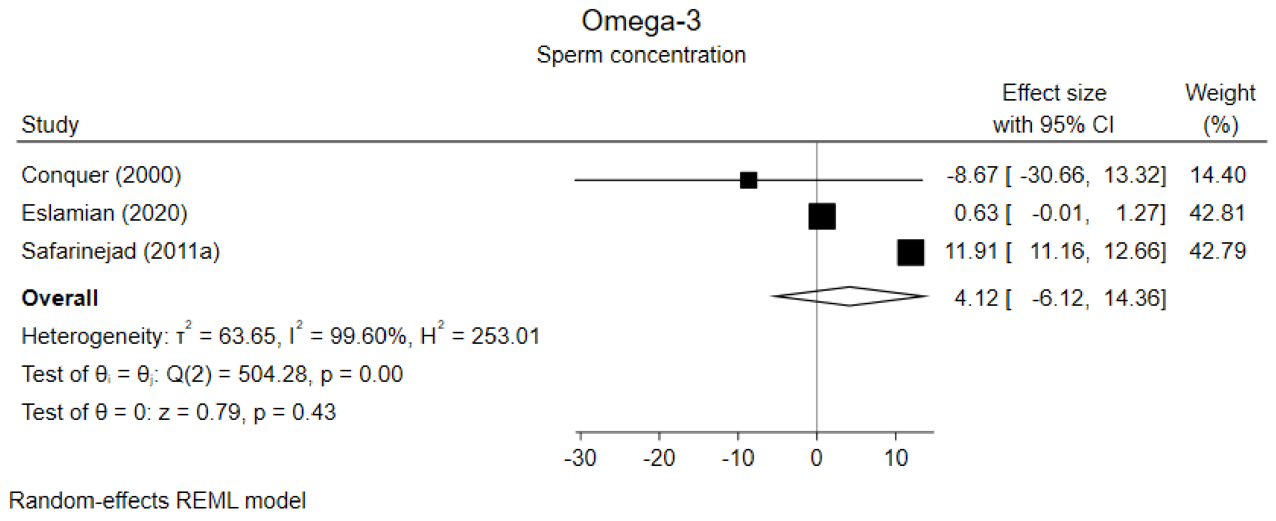

Figure S93. Forest plot of secondary analysis on the effect of omega-3 fatty acids on sperm concentration. The analysis is adjusted for baseline using pseudo-individual participant data.

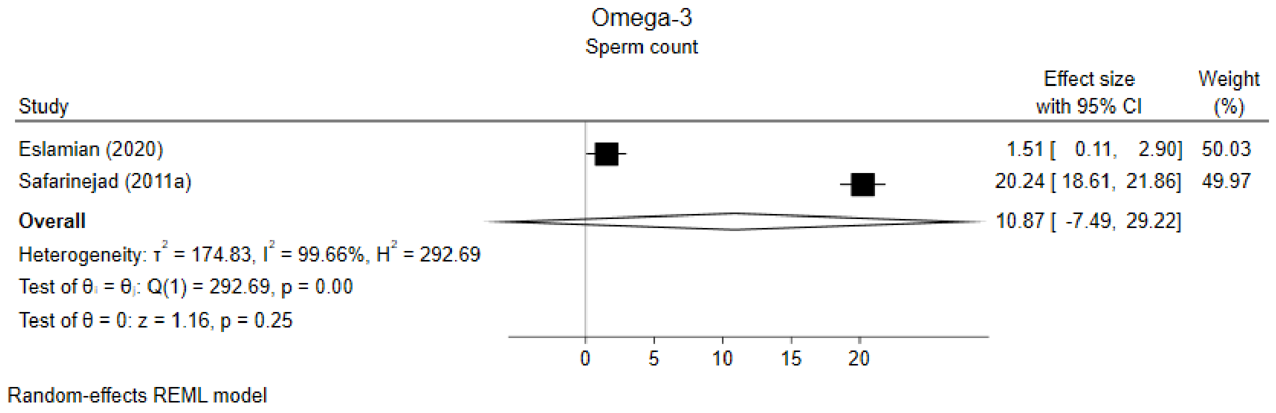

Figure S94. Forest plot of secondary analysis on the effect of omega-3 fatty acids on sperm count. The analysis is adjusted for baseline using pseudo-individual participant data.

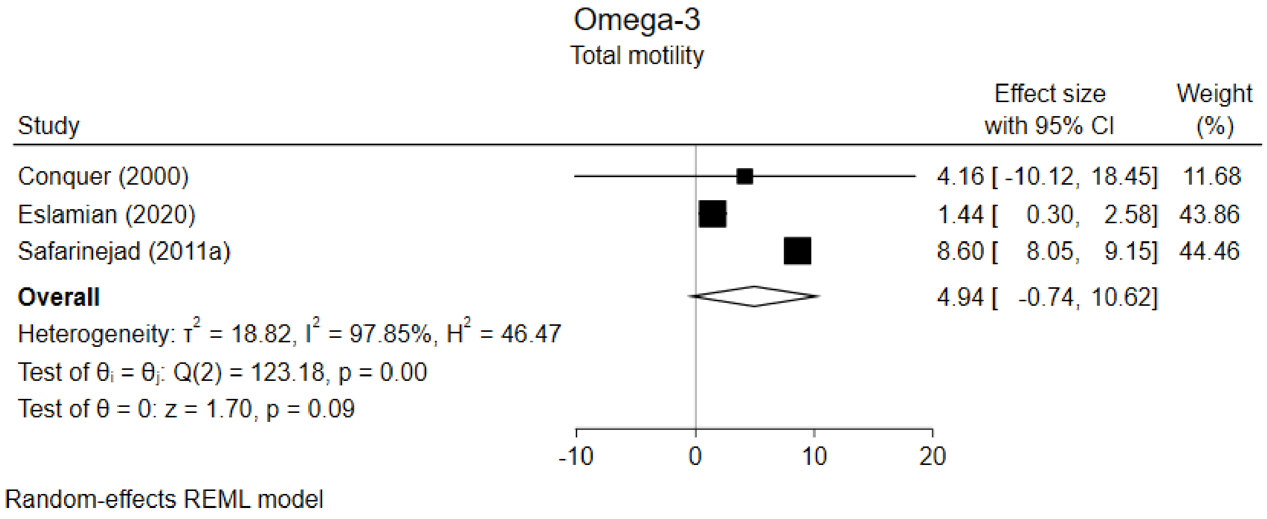

Figure S95. Forest plot of secondary analysis on the effect of omega-3 fatty acids on total motility. The analysis is adjusted for baseline using pseudo-individual participant data.

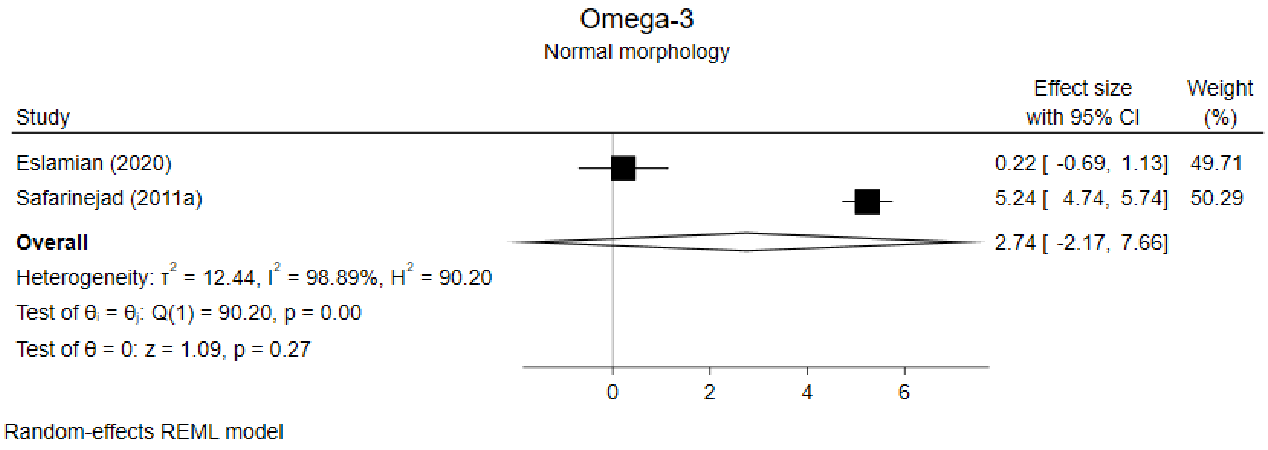

Figure S96. Forest plot of secondary analysis on the effect of omega-3 fatty acids on normal morphology. The analysis is adjusted for baseline using pseudo-individual participant data.

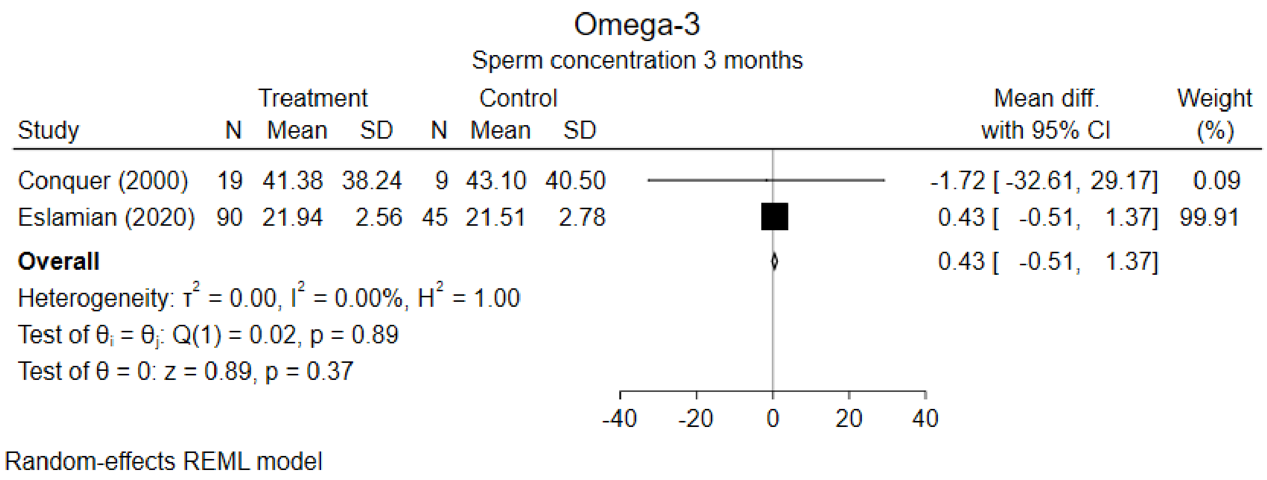

Figure S97. Forest plot of subgroup analysis on the effect of three months of omega-3 fatty acids use on sperm concentration.

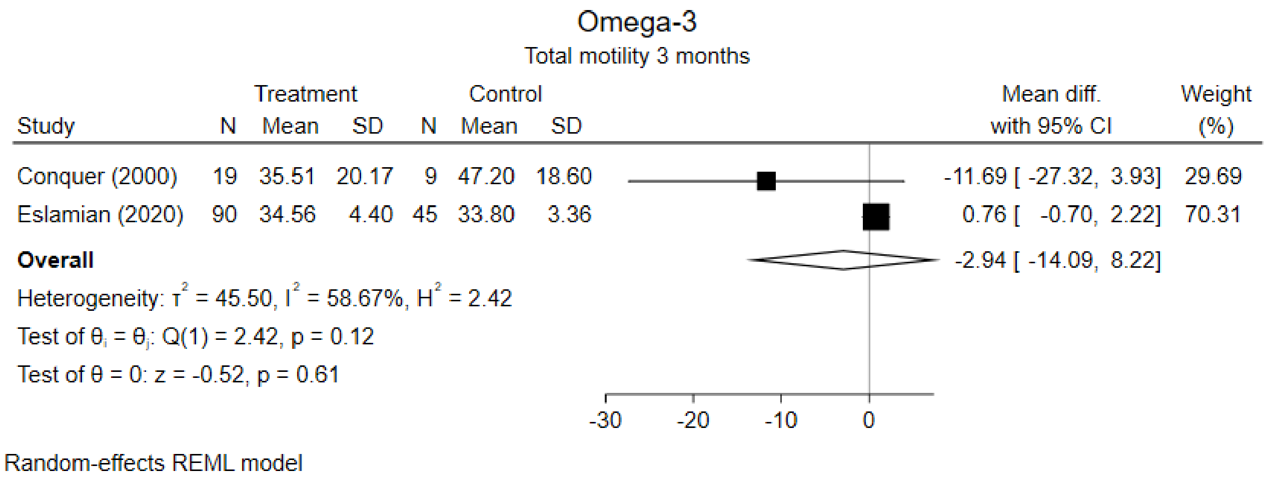

Figure S98. Forest plot of subgroup analysis on the effect of three months of omega-3 fatty acids use on total motility.

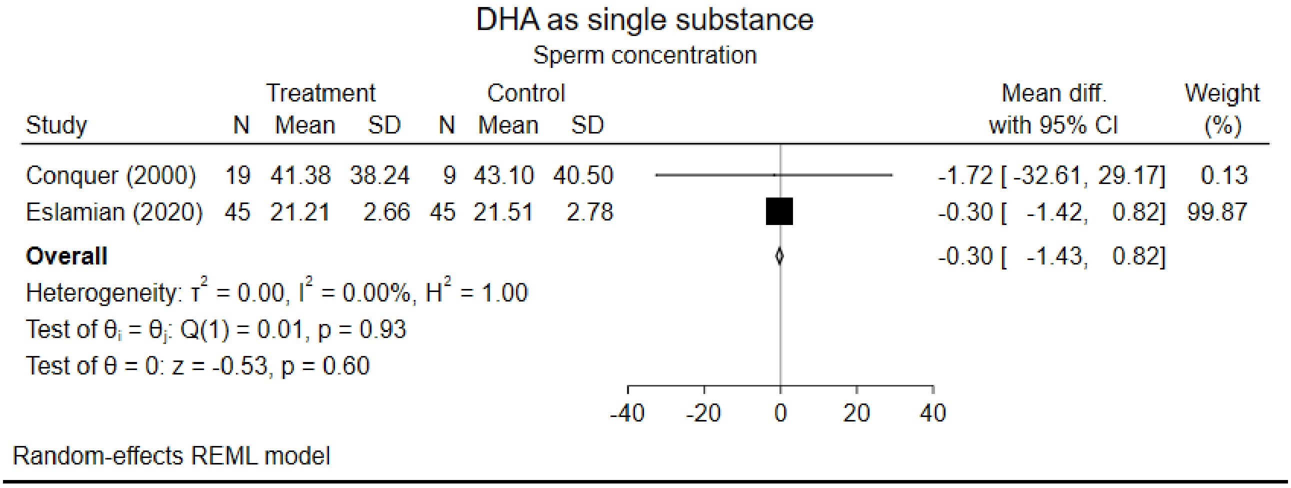

Figure S99. Forest plot of subgroup analysis on the effect of Docosahexaenoic acid (DHA) as a single substance on sperm concentration.

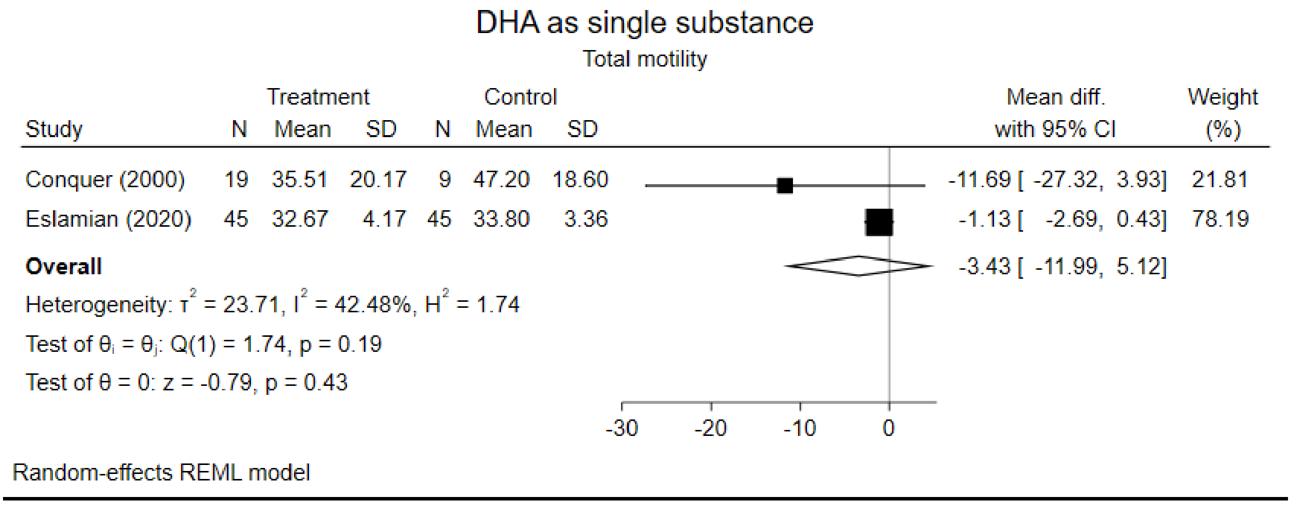

Figure S100. Forest plot of subgroup analysis on the effect of Docosahexaenoic acid (DHA) as a single substance on total motility.

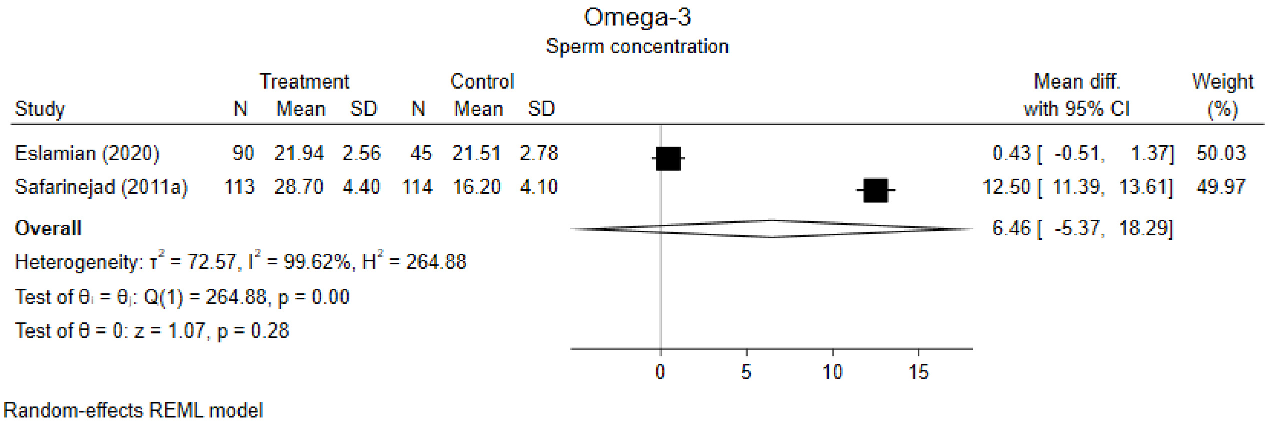

Figure S101. Forest plot on sensitivity analysis on the effect of omega-3 fatty acids on sperm concentration. Studies evaluated as having a high risk of bias have been excluded from the analysis.

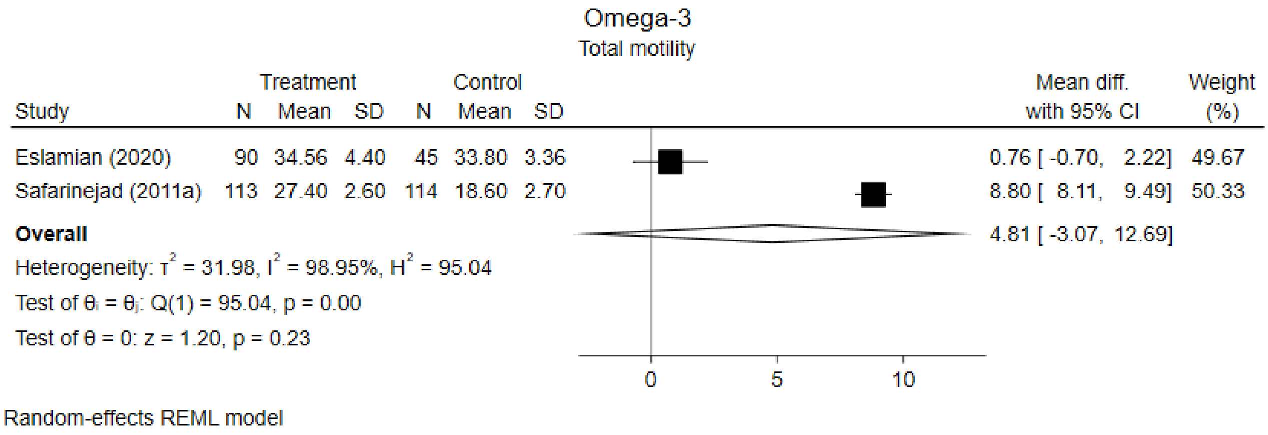

Figure S102. Forest plot on sensitivity analysis on the effect of omega-3 fatty acids on total motility. Studies evaluated as having a high risk of bias have been excluded from the analysis.

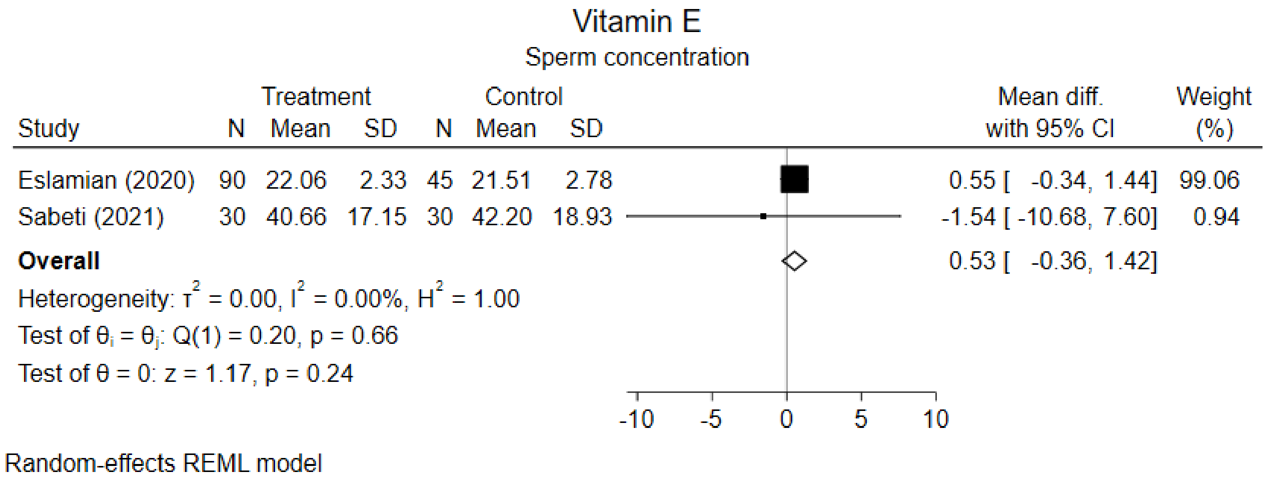

Figure S103. Forest plot of primary analysis on the effect of vitamin E on sperm concentration.

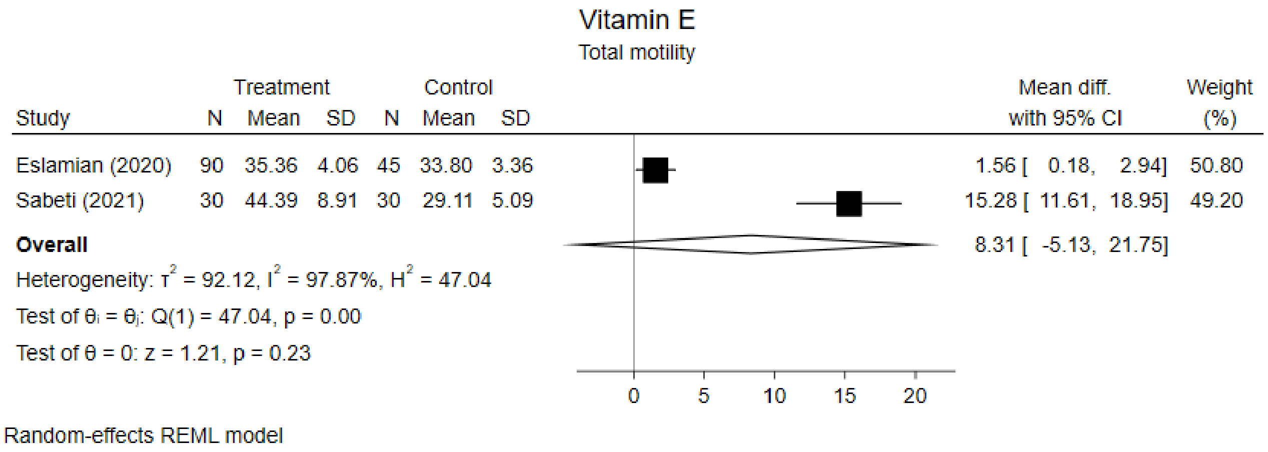

Figure S104. Forest plot of primary analysis on the effect of vitamin E on total motility.

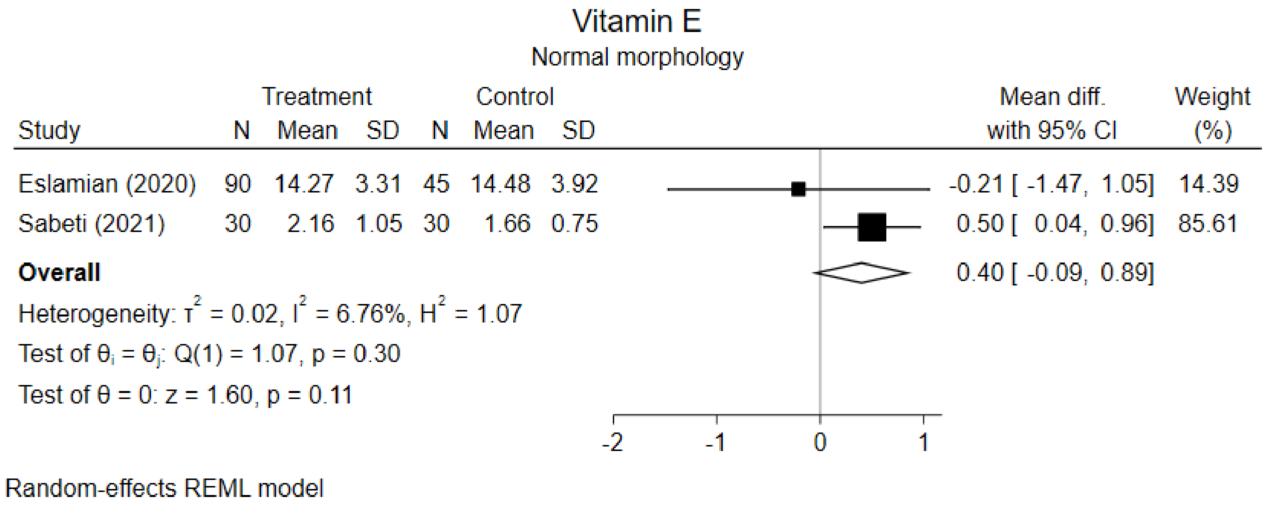

Figure S105. Forest plot of primary analysis on the effect of vitamin E on normal morphology.

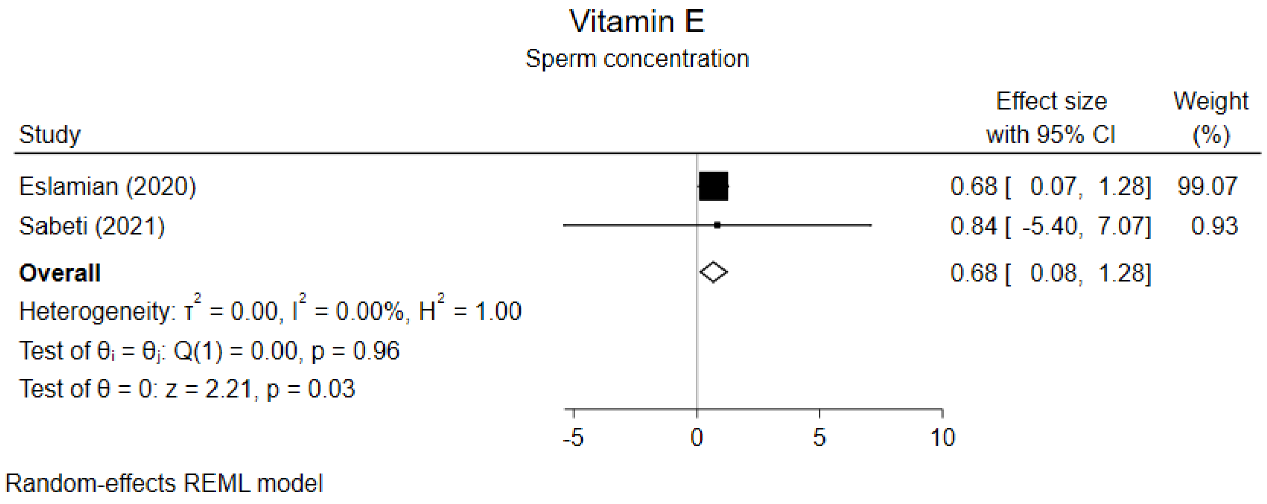

Figure S106. Forest plot of secondary analysis on the effect of vitamin E on sperm concentration. The analysis is adjusted for baseline using pseudo-individual participant data.

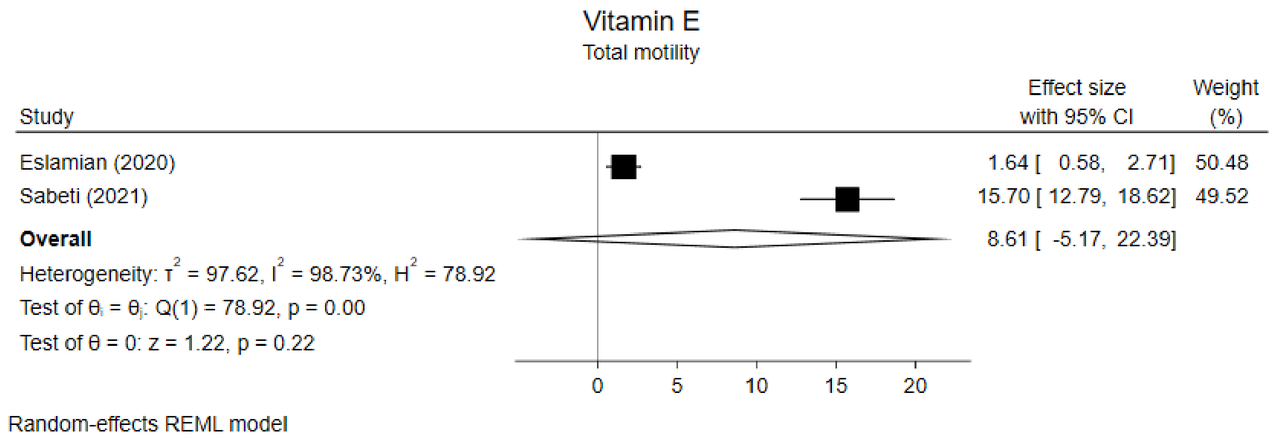

Figure S107. Forest plot of secondary analysis on the effect of vitamin E on total motility. The analysis is adjusted for baseline using pseudo-individual participant data.

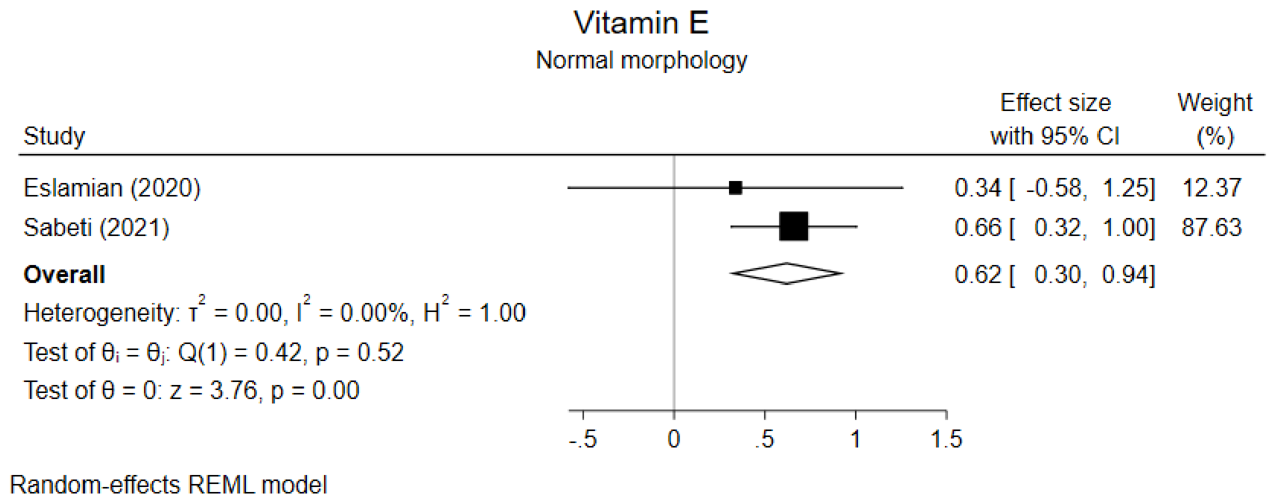

Figure S108. Forest plot of secondary analysis on the effect of vitamin E on normal morphology. The analysis is adjusted for baseline using pseudo-individual participant data.

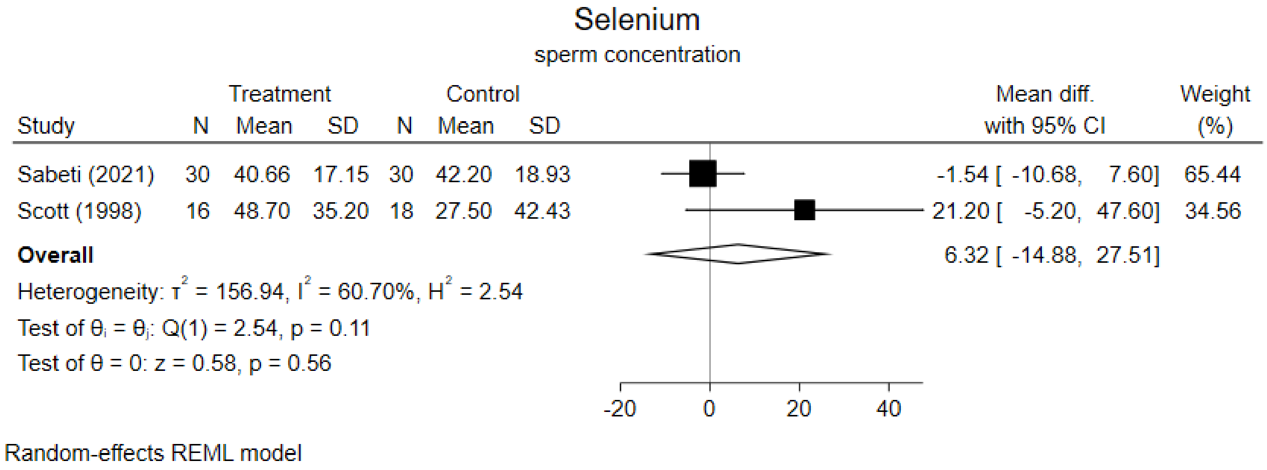

Figure S109. Forest plot of primary analysis on the effect of selenium on sperm concentration.

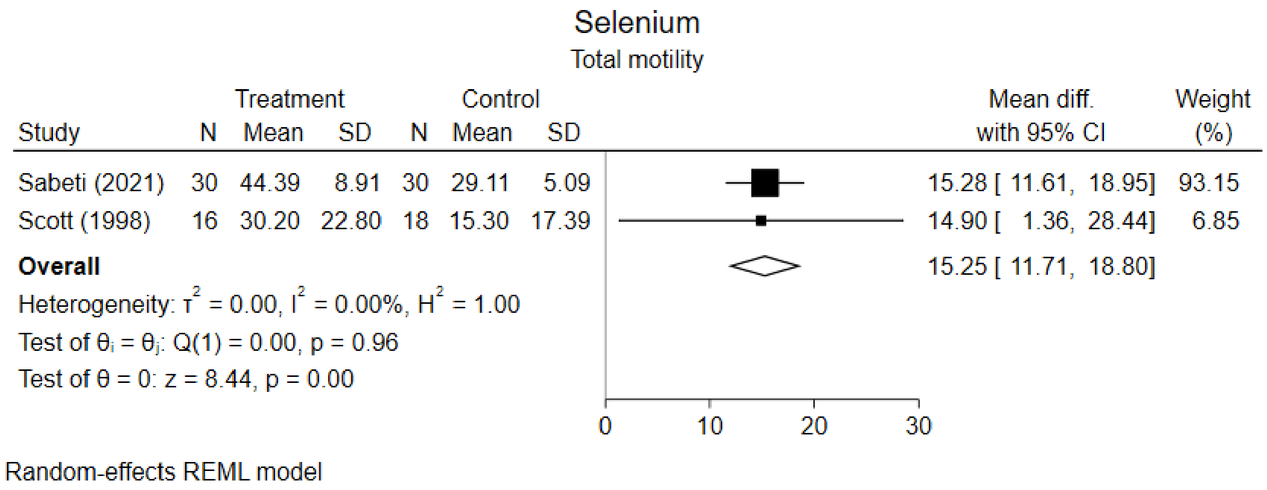

Figure S110. Forest plot of primary analysis on the effect of selenium on total motility.

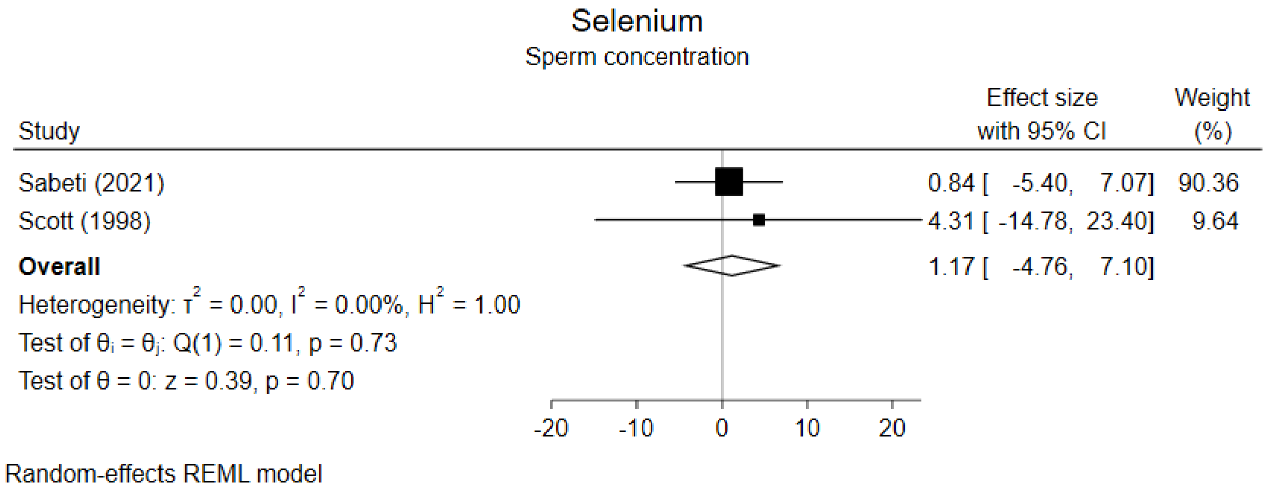

Figure S111. Forest plot of secondary analysis on the effect of selenium on sperm concentration. The analysis is adjusted for baseline using pseudo-individual participant data.

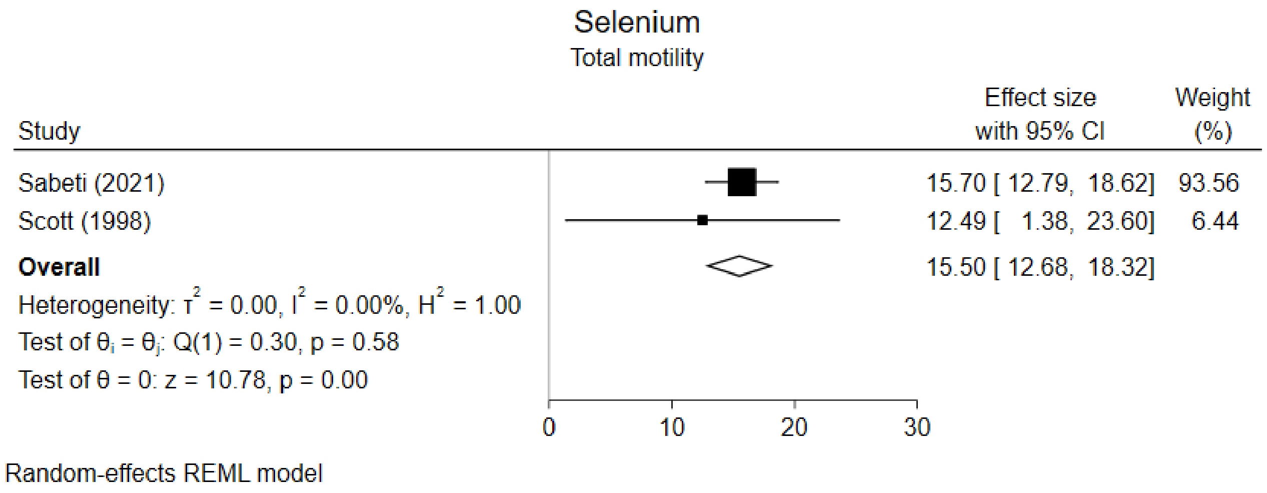

Figure S112. Forest plot of secondary analysis on the effect of selenium on total motility. The analysis is adjusted for baseline using pseudo-individual participant data.

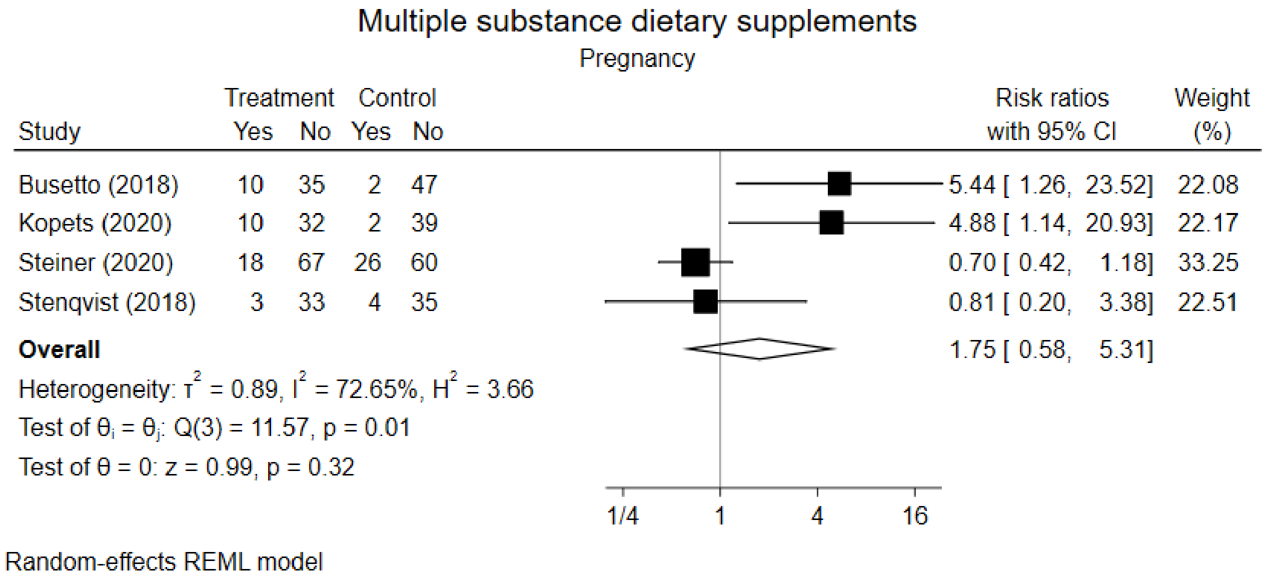

Figure S113. Forest plot of primary analysis on the effect of multiple substance dietary supplements on pregnancy.

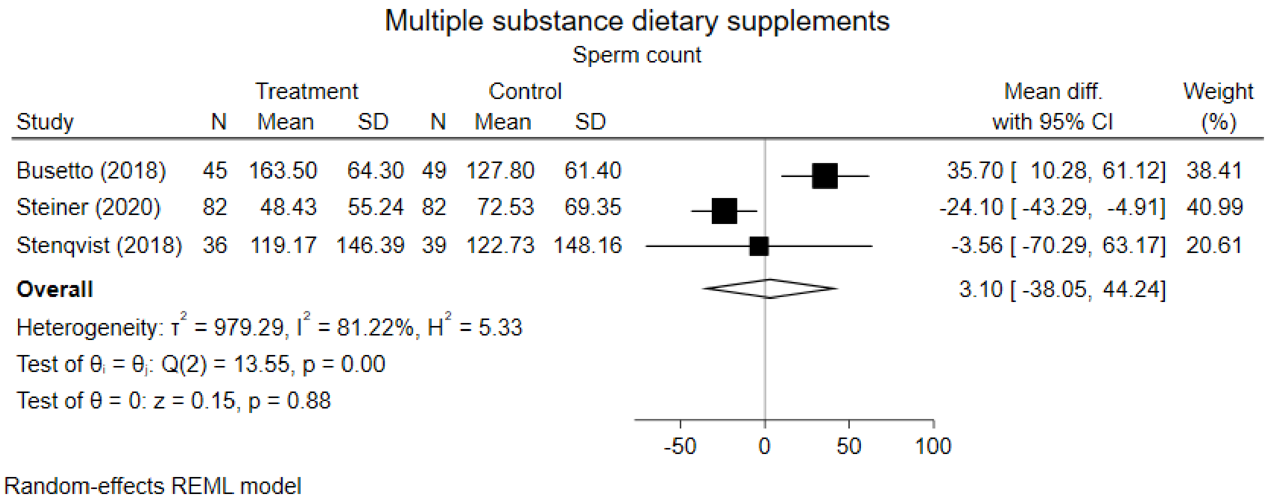

Figure S114. Forest plot of primary analysis on the effect of multiple substance dietary supplements on sperm count.

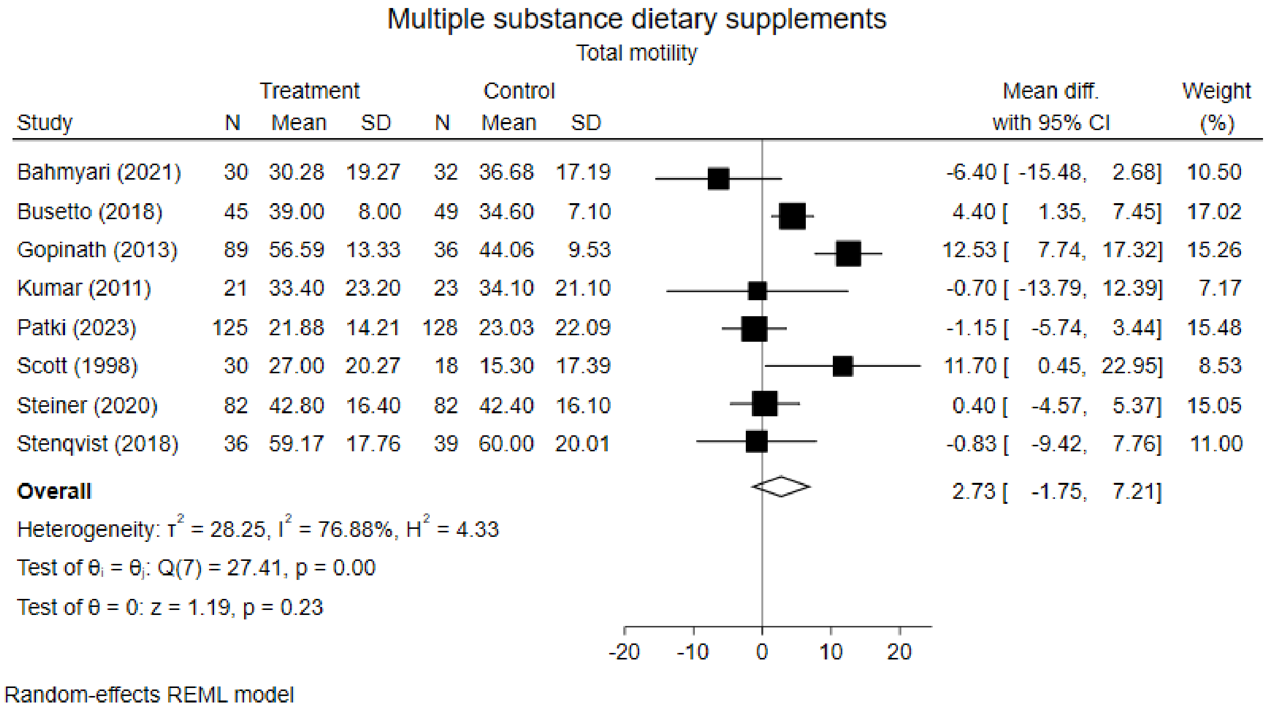

Figure S115. Forest plot of primary analysis on the effect of multiple substance dietary supplements on total motility.

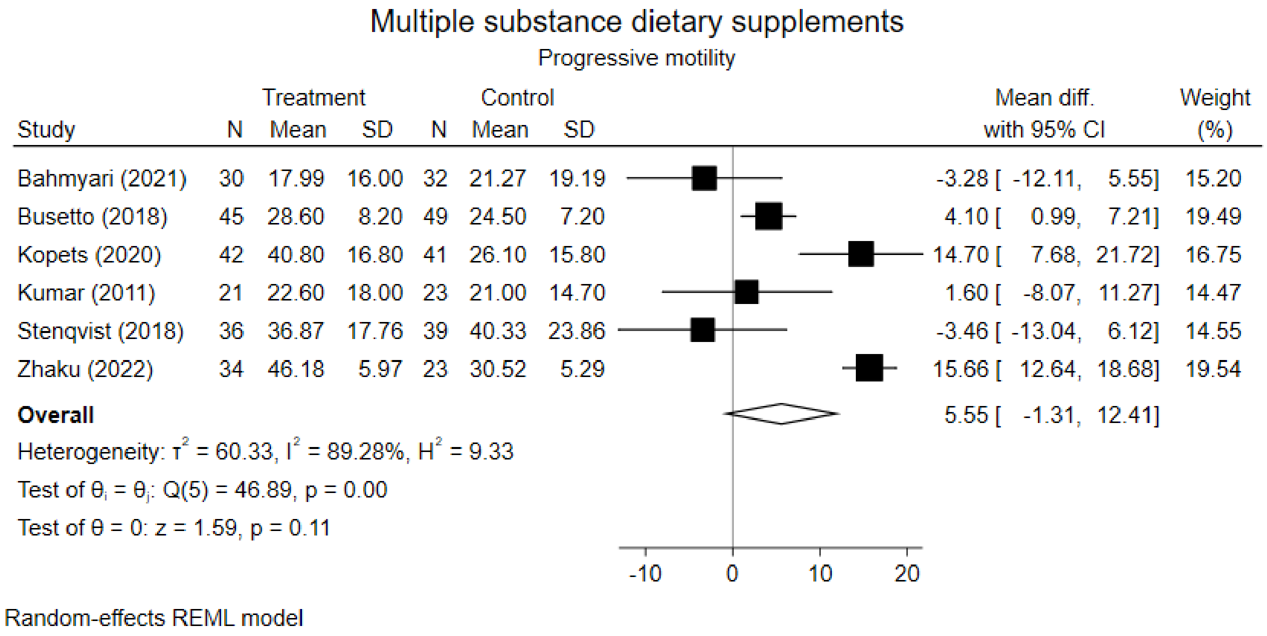

Figure S116. Forest plot of primary analysis on the effect of multiple substance dietary supplements on progressive motility.

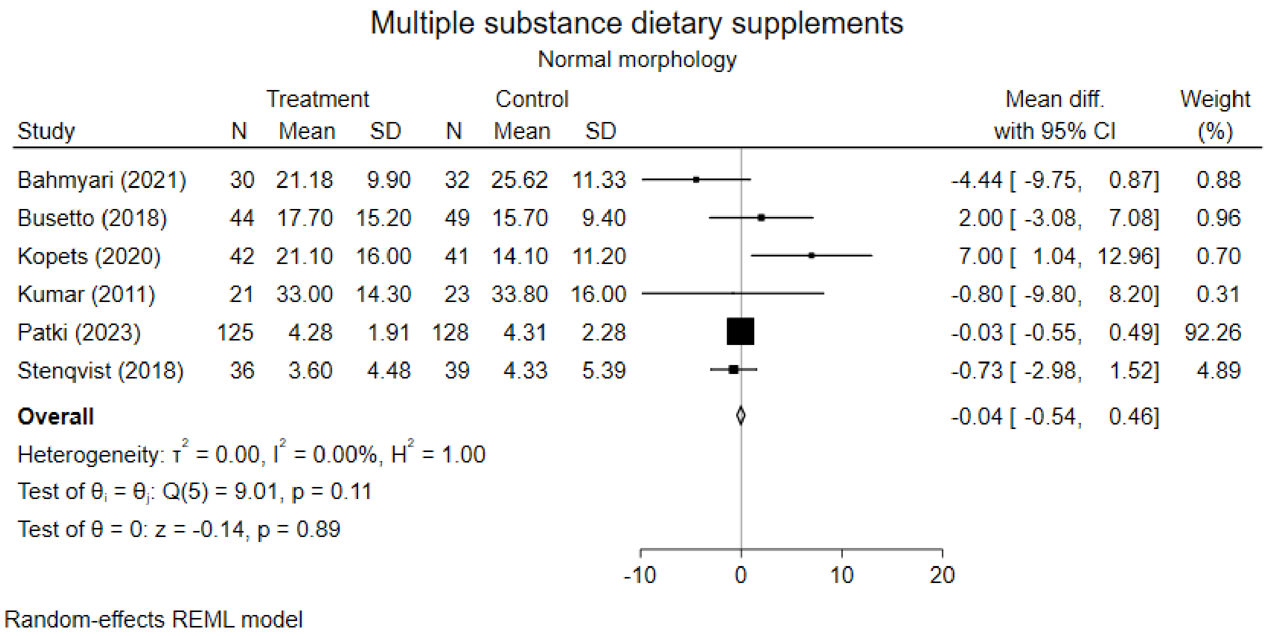

Figure S117. Forest plot of primary analysis on the effect of multiple substance dietary supplements on normal morphology.

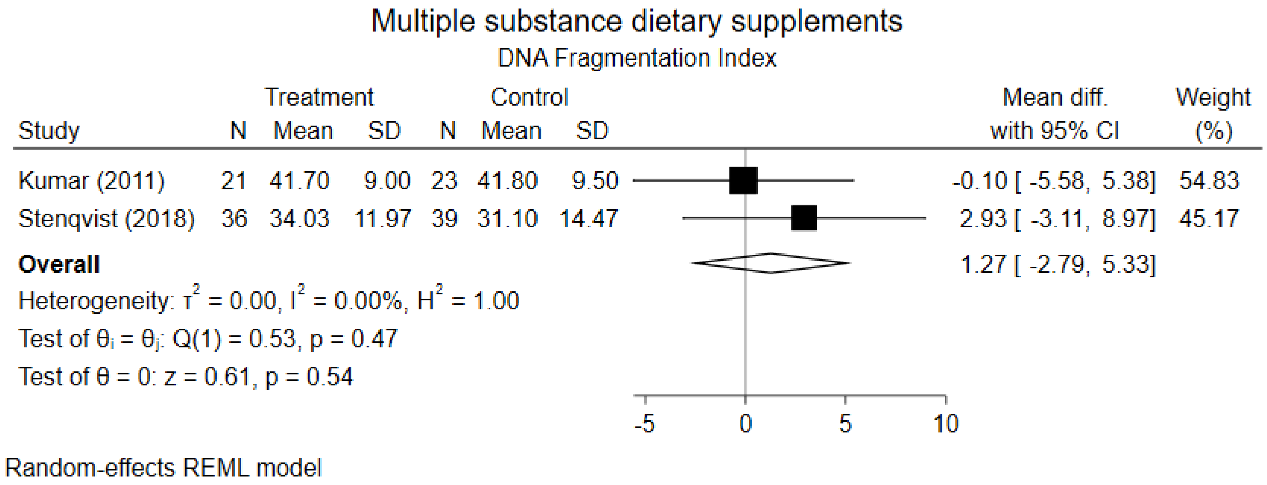

Figure S118. Forest plot of primary analysis on the effect of multiple substance dietary supplements on DNA Fragmentation Index.

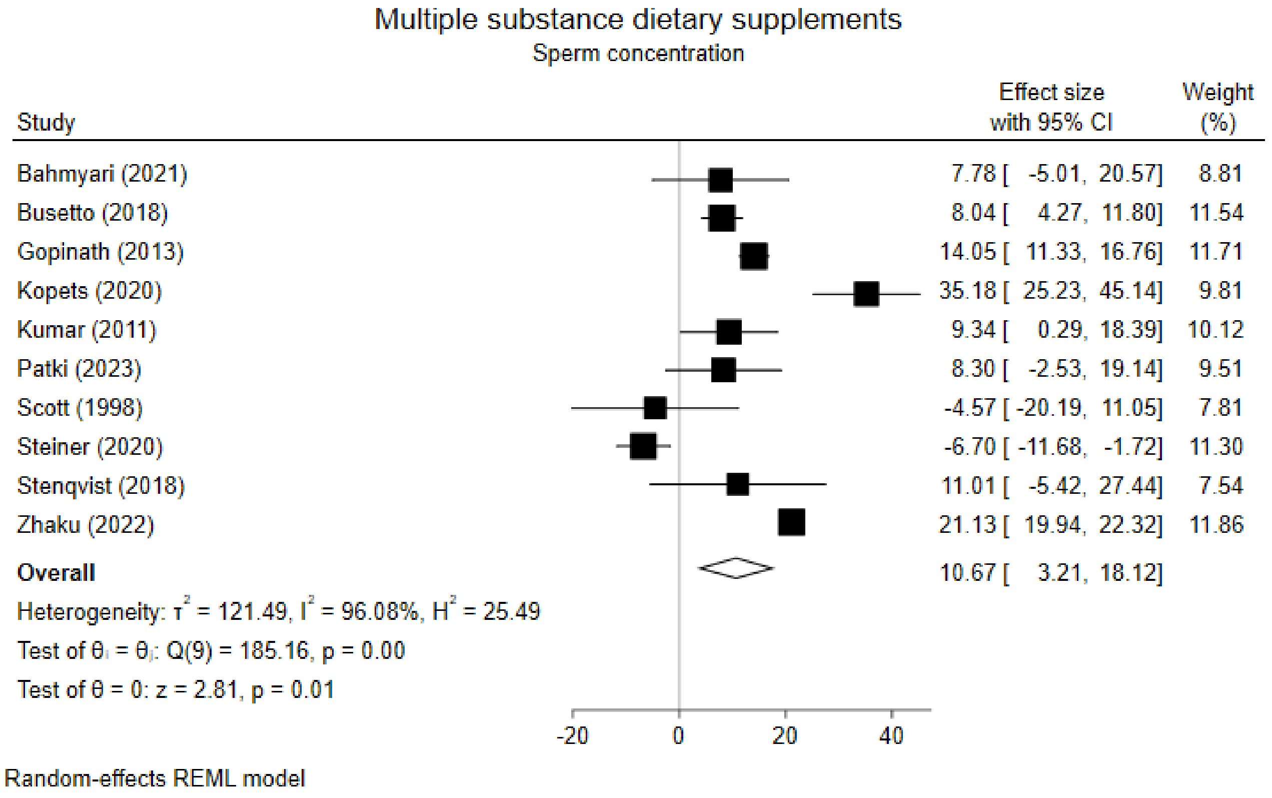

Figure S119. Forest plot of secondary analysis on the effect of multiple substance dietary supplements on sperm concentration. The analysis is adjusted for baseline using pseudo-individual participant data.

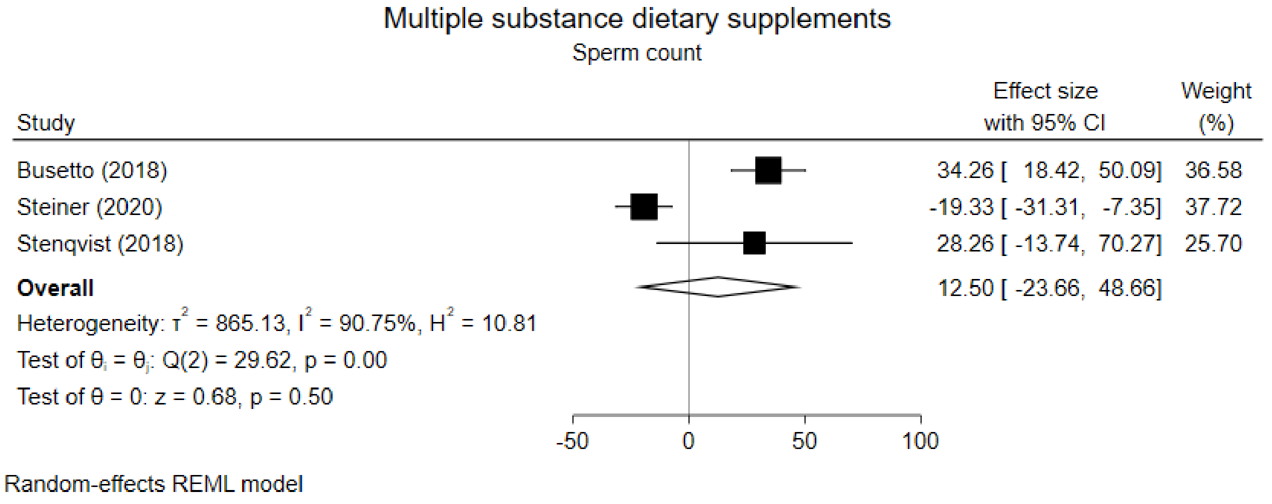

Figure S120. Forest plot of secondary analysis on the effect of multiple substance dietary supplements on sperm count. The analysis is adjusted for baseline using pseudo-individual participant data.

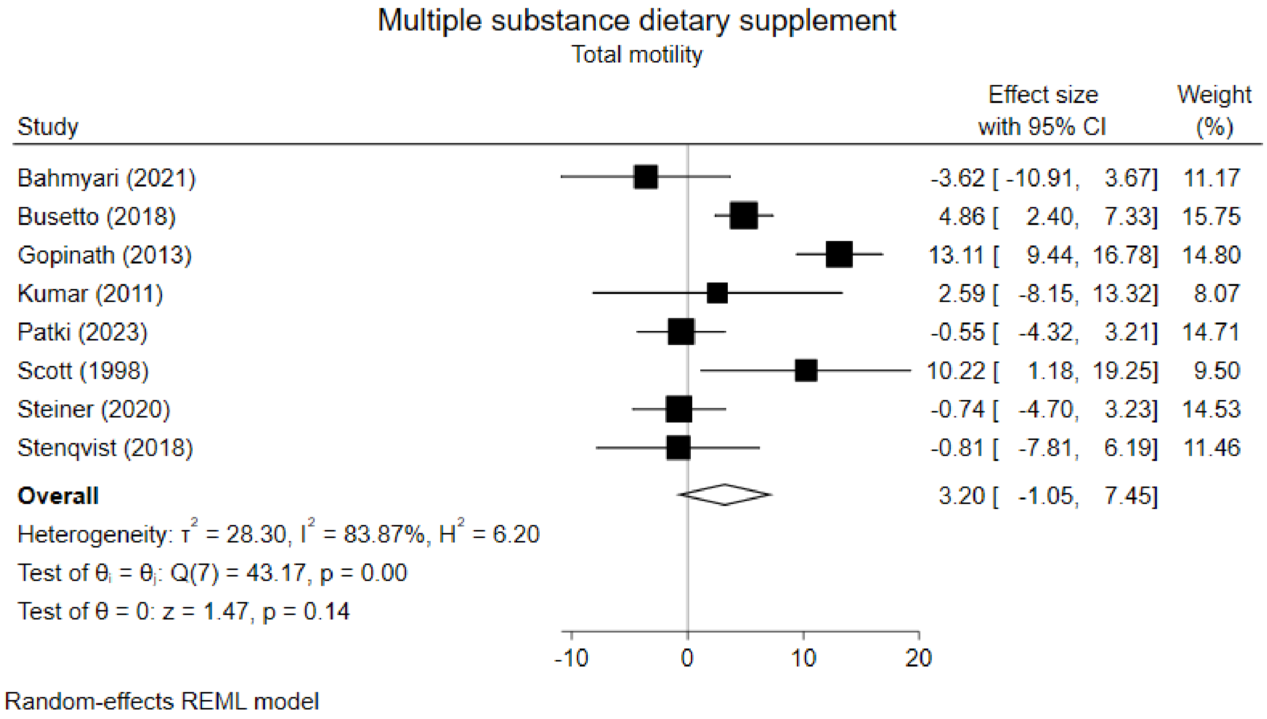

Figure S121. Forest plot of secondary analysis on the effect of multiple substance dietary supplements on total motility. The analysis is adjusted for baseline using pseudo-individual participant data.

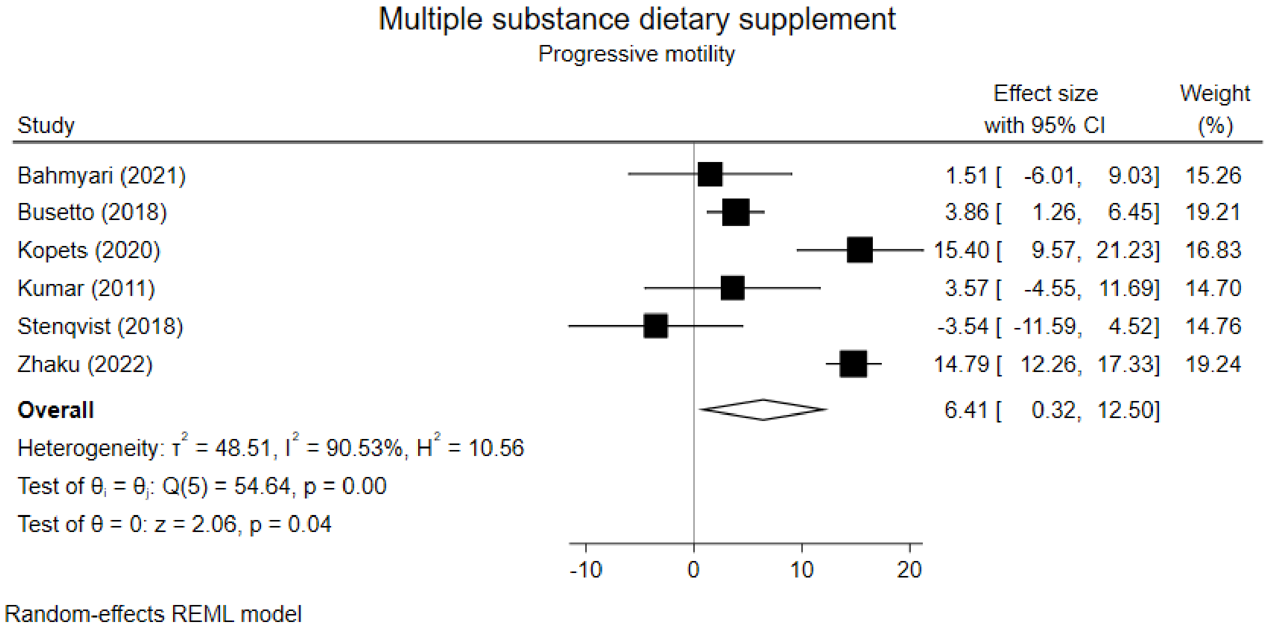

Figure S122. Forest plot of secondary analysis on the effect of multiple substance dietary supplements on progressive motility. The analysis is adjusted for baseline using pseudo-individual participant data.

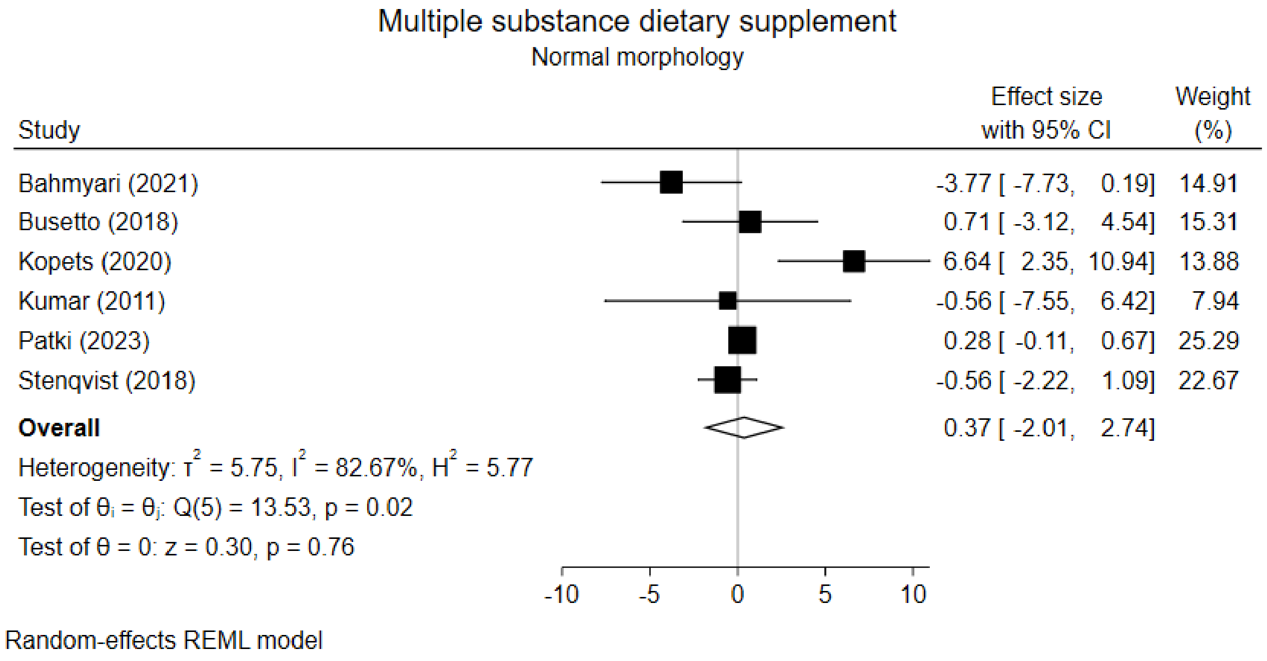

Figure S123. Forest plot of secondary analysis on the effect of multiple substance dietary supplements on normal morphology. The analysis is adjusted for baseline using pseudo-individual participant data.

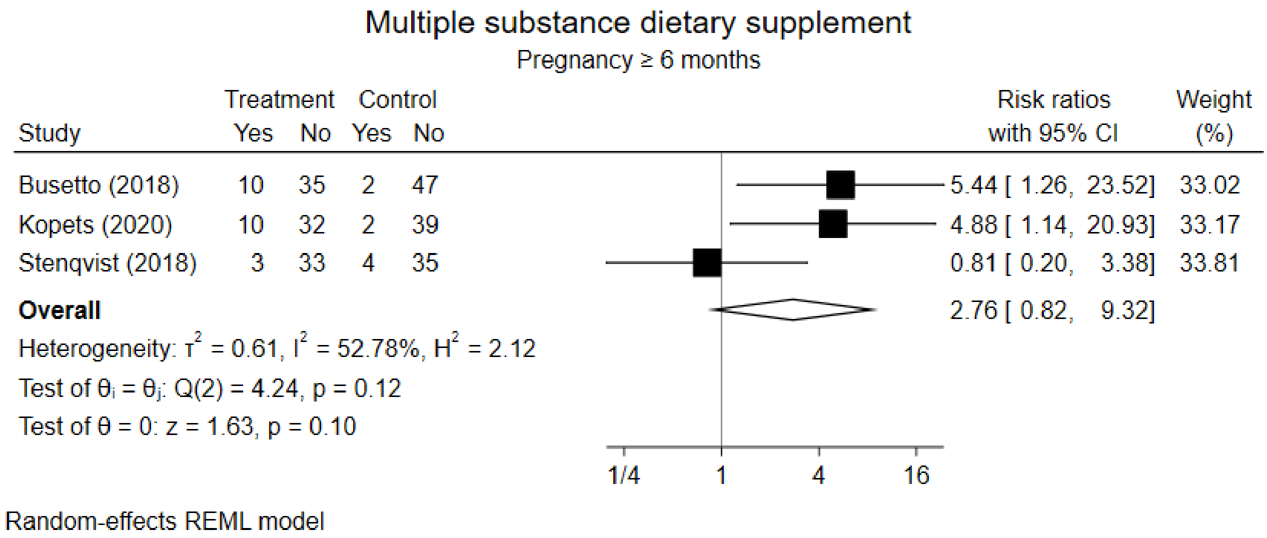

Figure S124. Forest plot of subgroup analysis on the effect of six or more months of multiple substance dietary supplements use on pregnancy.

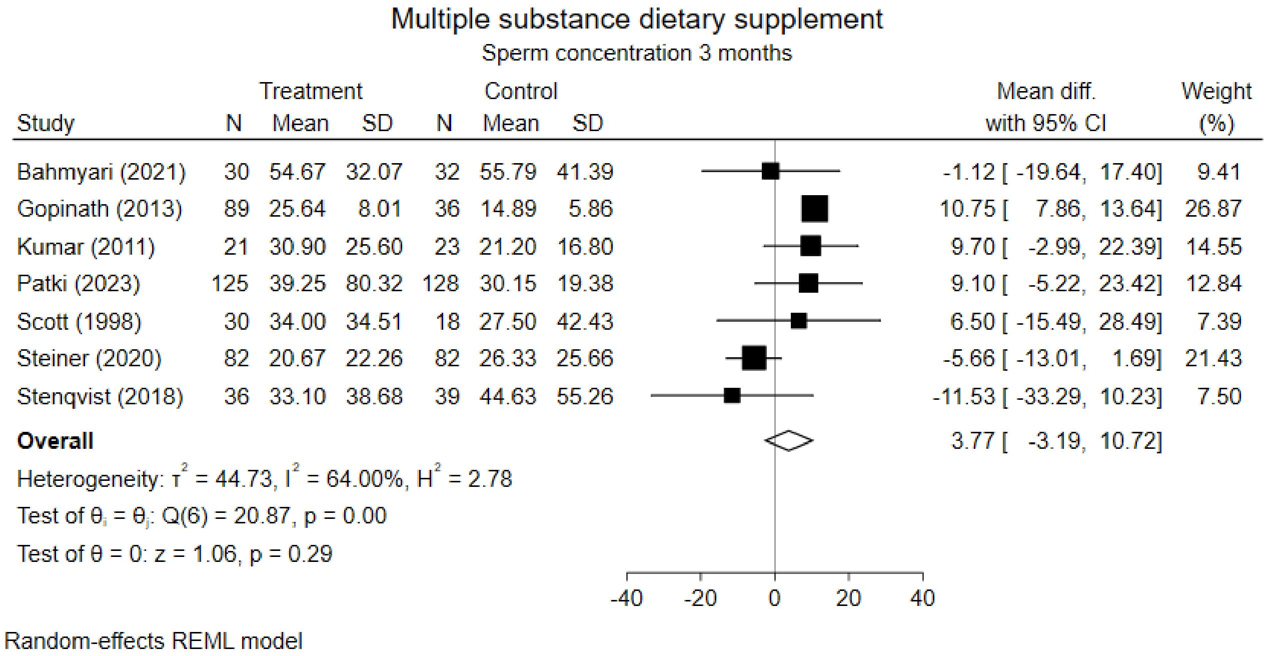

Figure S125. Forest plot of subgroup analysis on the effect of three months of multiple substance dietary supplements use on sperm concentration.

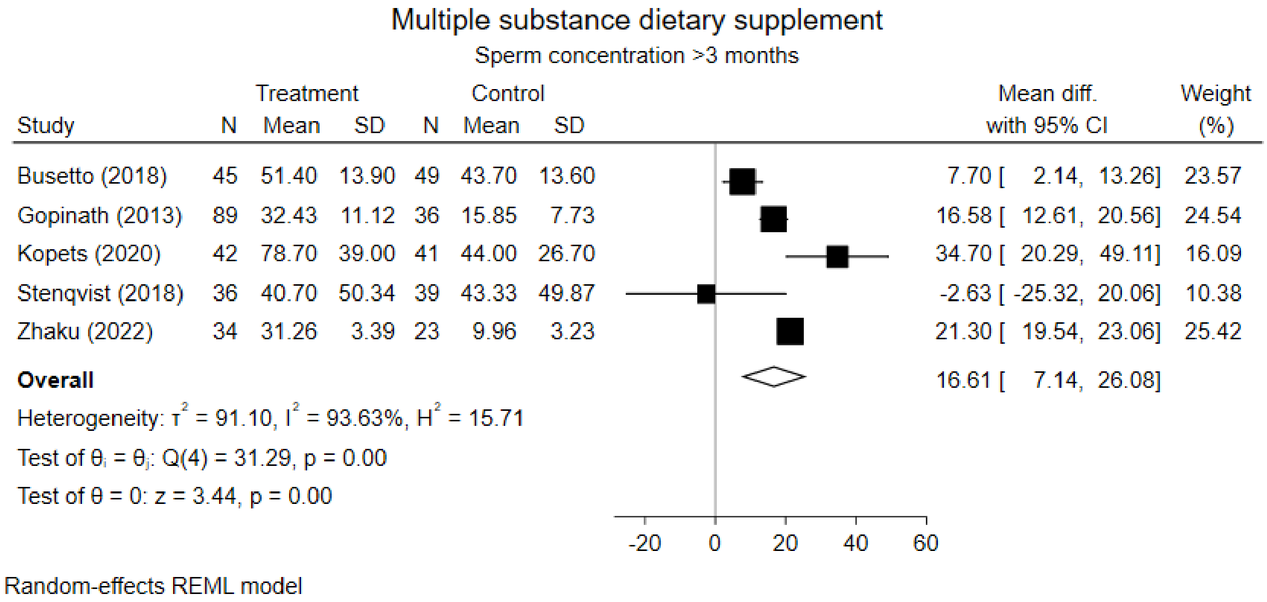

Figure S126. Forest plot of subgroup analysis on the effect of more than three months of multiple substance dietary supplements use on sperm concentration.

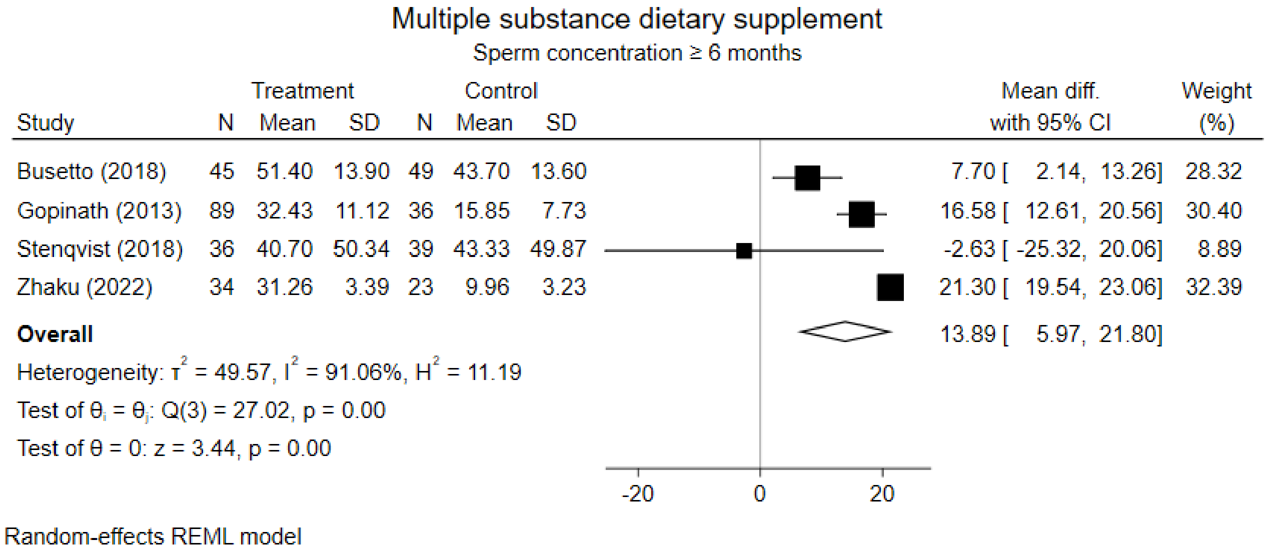

Figure S127. Forest plot of subgroup analysis on the effect of six or more months of multiple substance dietary supplements use on sperm concentration.

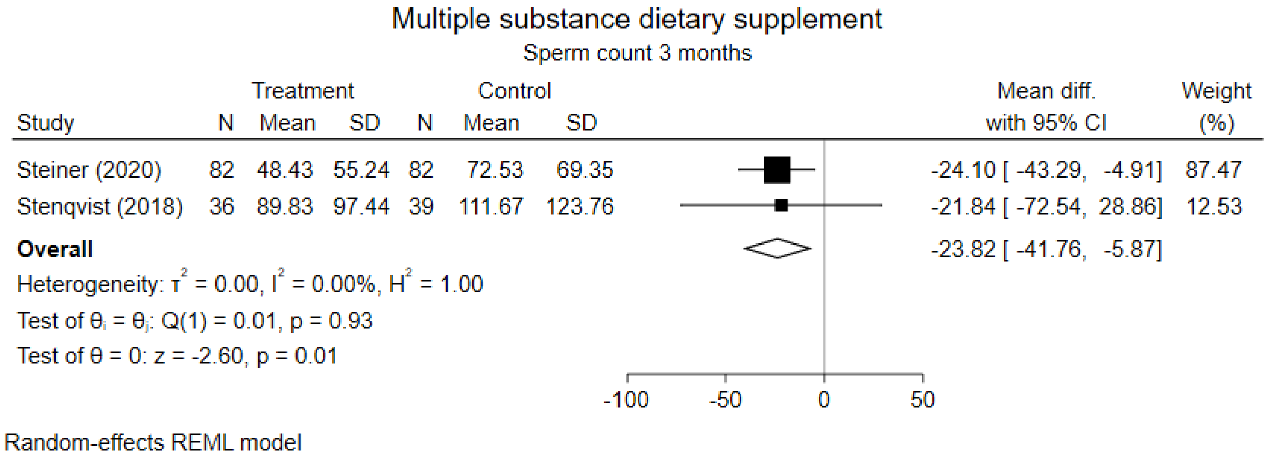

Figure S128. Forest plot of subgroup analysis on the effect of three months of multiple substance dietary supplements use on sperm count.

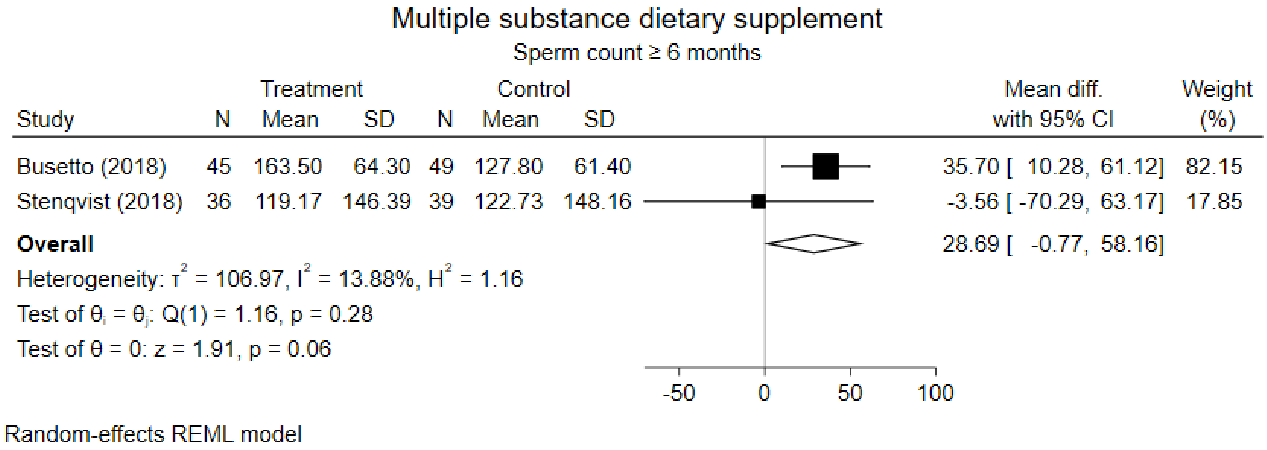

Figure S129. Forest plot of subgroup analysis on the effect of six or more months of multiple substance dietary supplements use on sperm count.

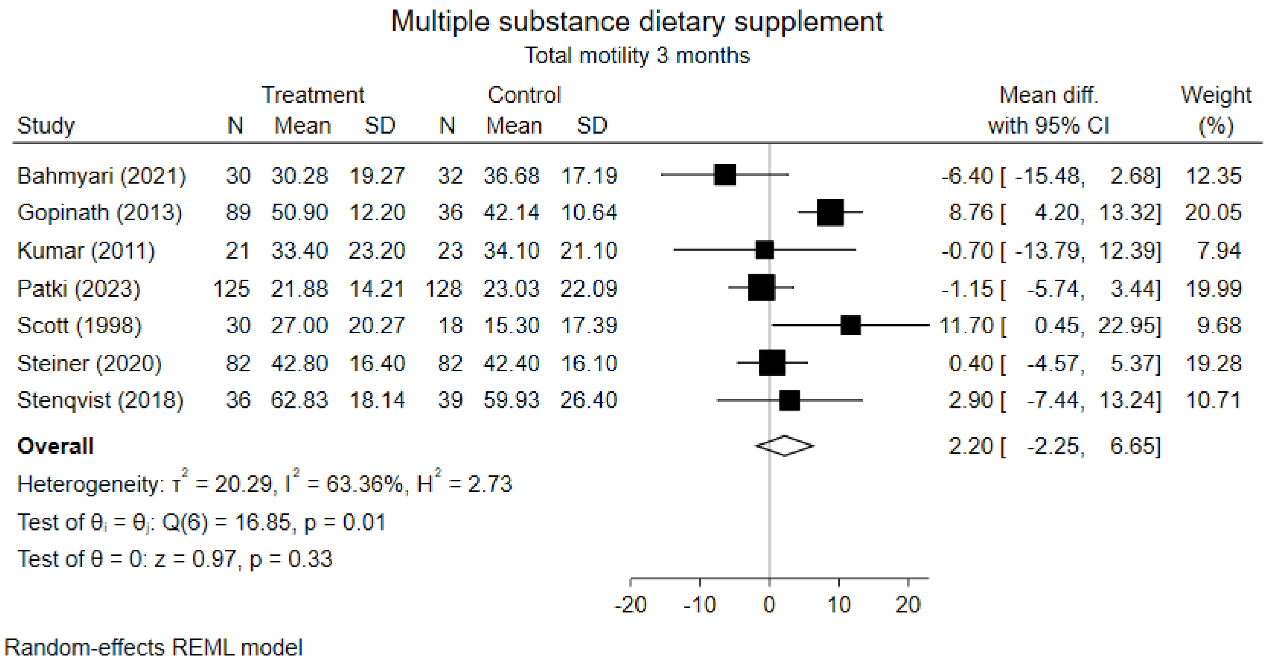

Figure S130. Forest plot of subgroup analysis on the effect of three months of multiple substance dietary supplements use on total motility.

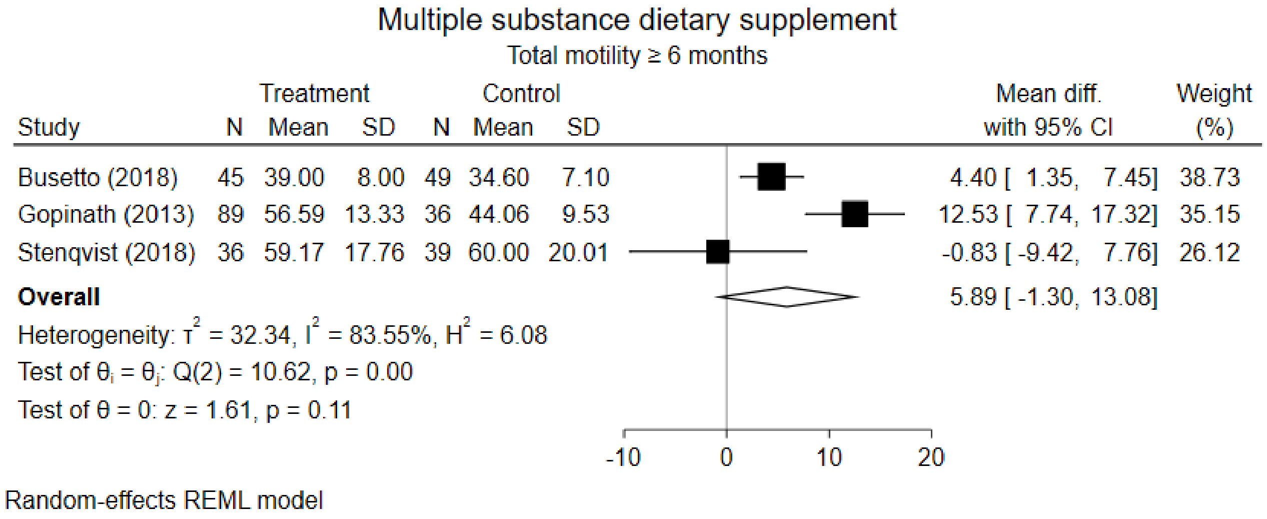

Figure S131. Forest plot of subgroup analysis on the effect of six or more months of multiple substance dietary supplements use on total motility.

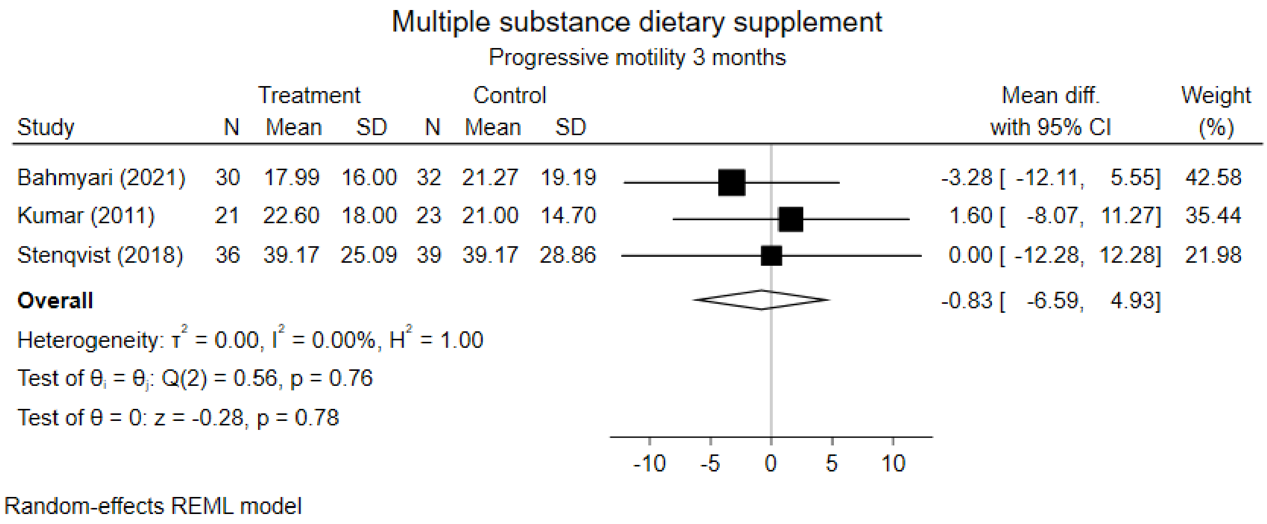

Figure S132. Forest plot of subgroup analysis on the effect of three months of multiple substance dietary supplements use on progressive motility.

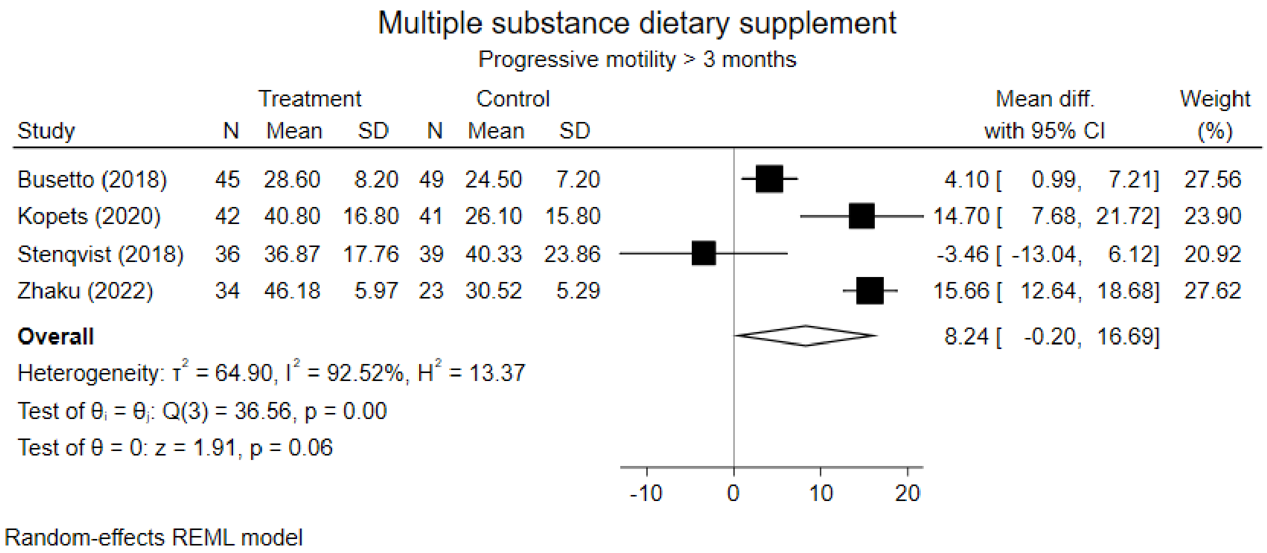

Figure S133. Forest plot of subgroup analysis on the effect of more than three months of multiple substance dietary supplements use on progressive motility.

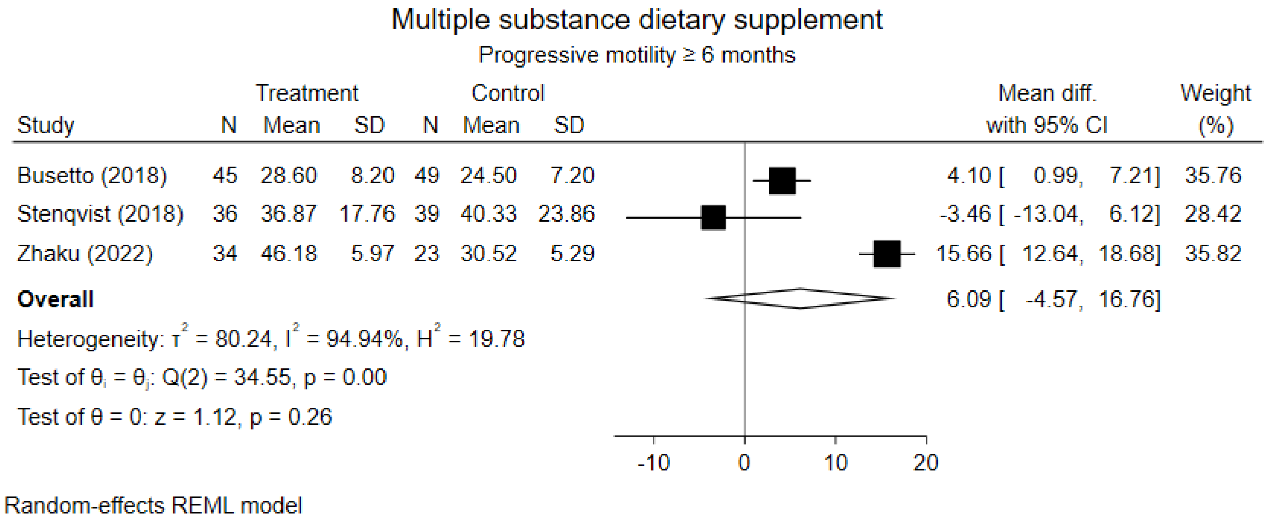

Figure S134. Forest plot of subgroup analysis on the effect of six or more months of multiple substance dietary supplements use on progressive motility.

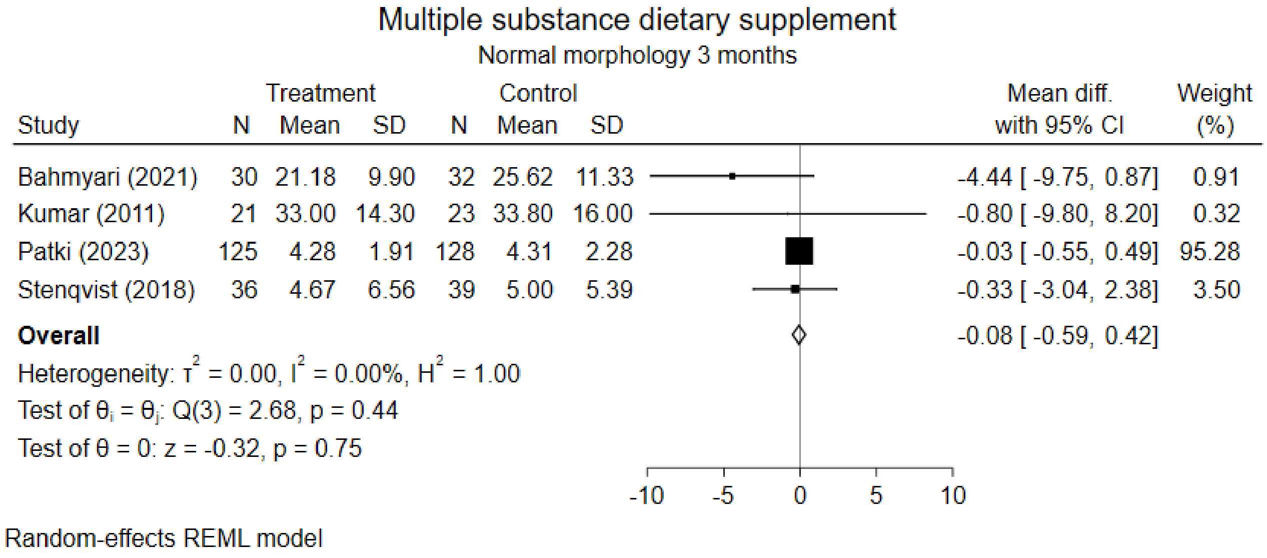

Figure S135. Forest plot of subgroup analysis on the effect of three months of multiple substance dietary supplements use on normal morphology.

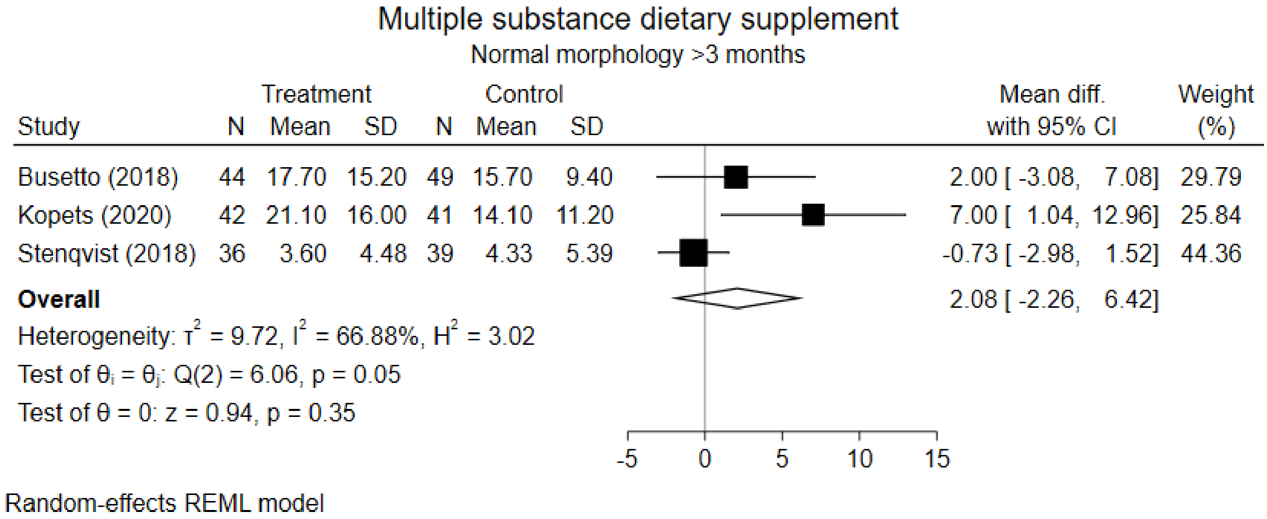

Figure S136. Forest plot of subgroup analysis on the effect of more than three months of multiple substance dietary supplements use on normal morphology.

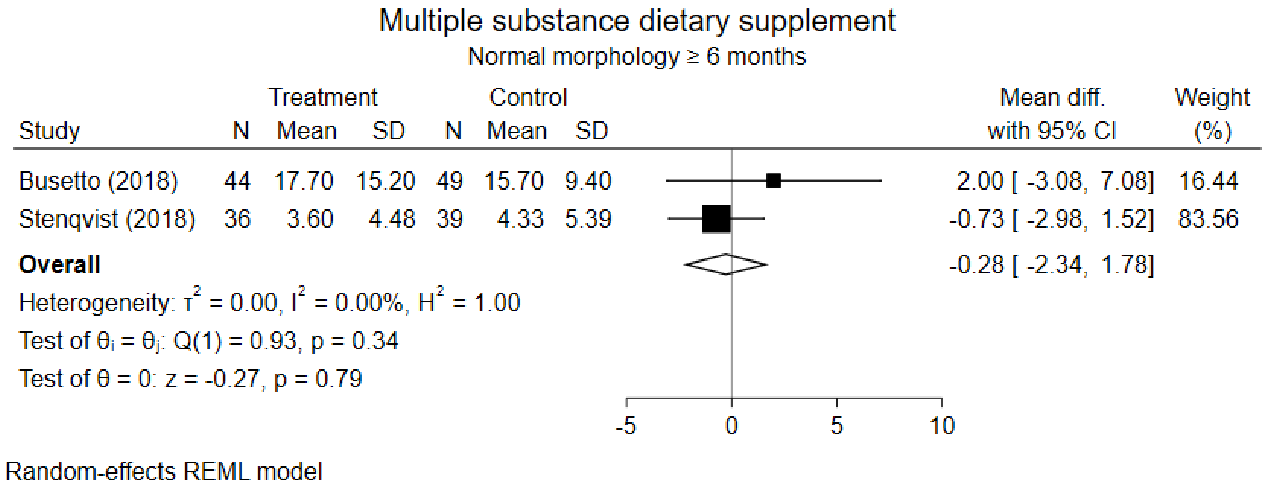

Figure S137. Forest plot of subgroup analysis on the effect of six or more months of multiple substance dietary supplements use on normal morphology.

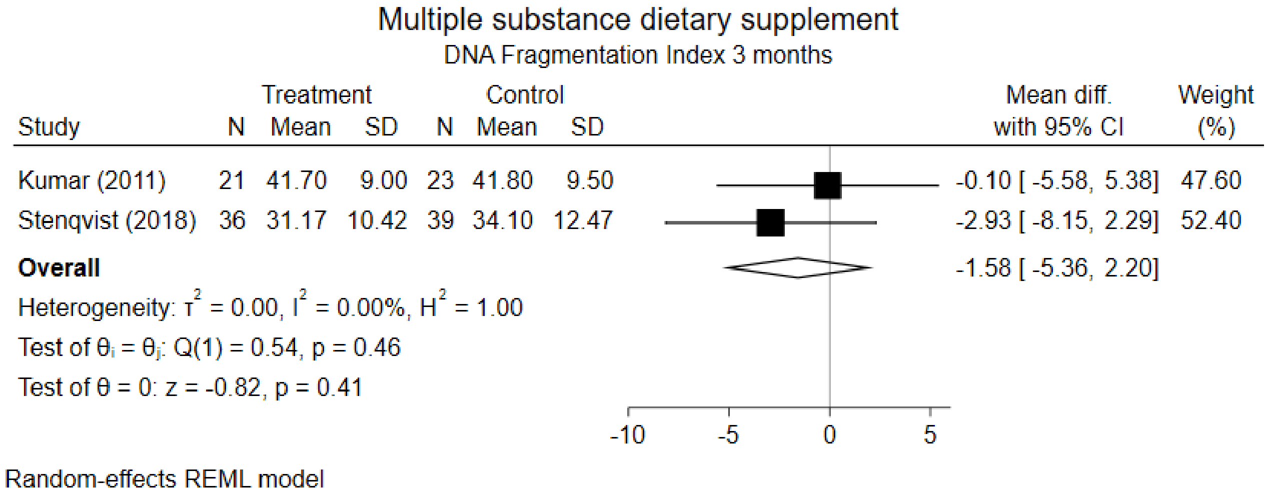

Figure S138. Forest plot of subgroup analysis on the effect of three months of multiple substance dietary supplements use on DNA Fragmentation Index.

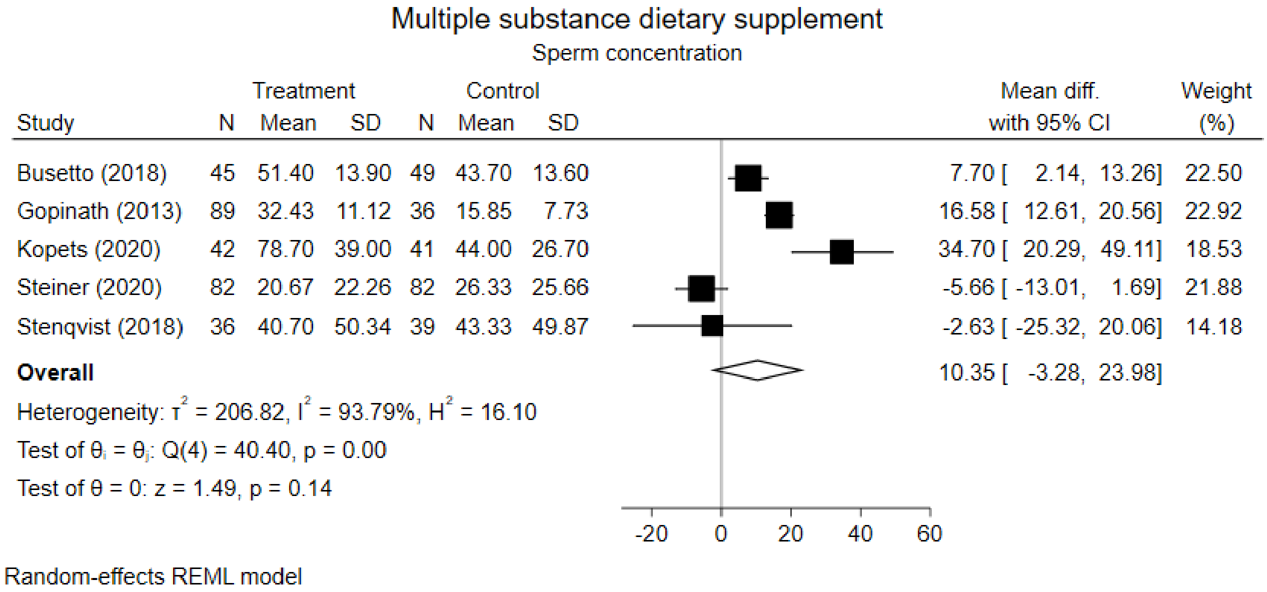

Figure S139. Forest plot on sensitivity analysis on the effect of multiple substance dietary supplements on sperm concentration. Studies evaluated as having a high risk of bias have been excluded from the analysis.

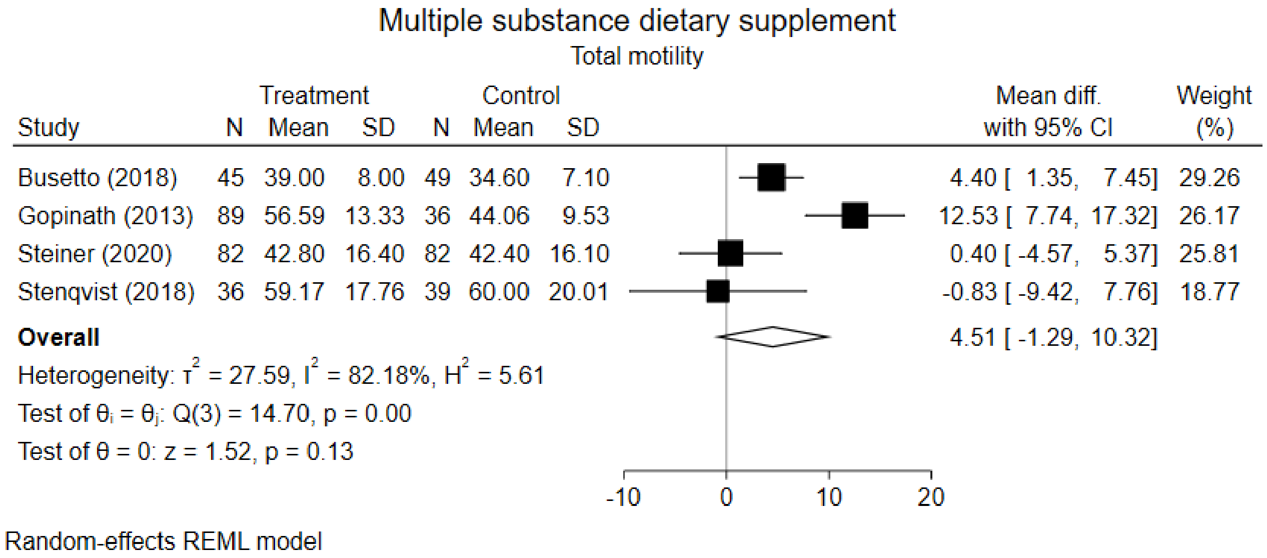

Figure S140. Forest plot on sensitivity analysis on the effect of multiple substance dietary supplements on total motility. Studies evaluated as having a high risk of bias have been excluded from the analysis.

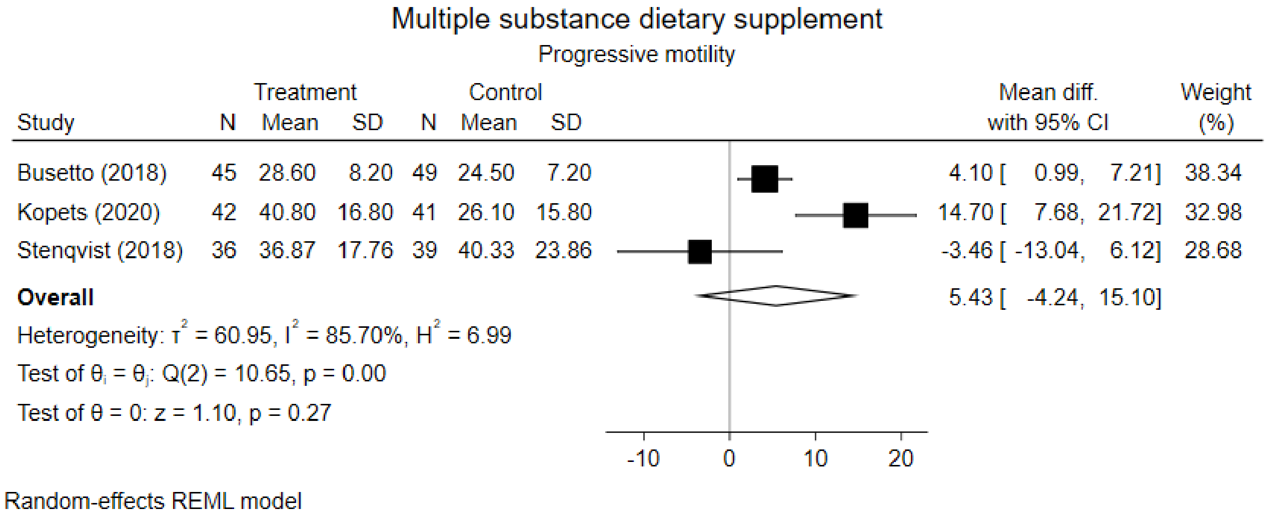

Figure S141. Forest plot on sensitivity analysis on the effect of multiple substance dietary supplements on progressive motility. Studies evaluated as having a high risk of bias have been excluded from the analysis.

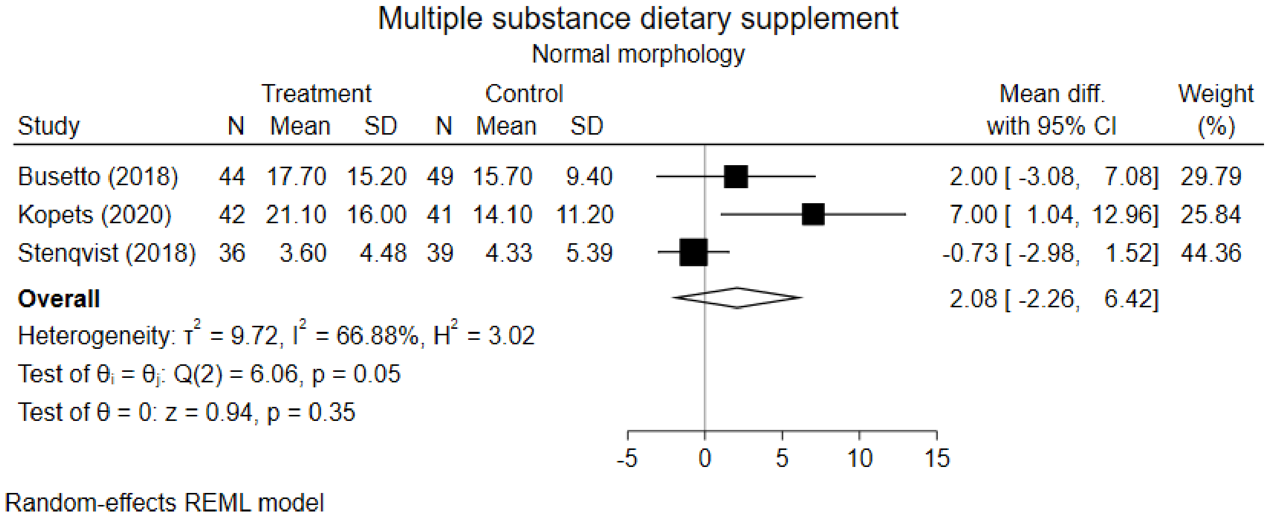

Figure S142. Forest plot on sensitivity analysis on the effect of multiple substance dietary supplements on normal morphology. Studies evaluated as having a high risk of bias have been excluded from the analysis.

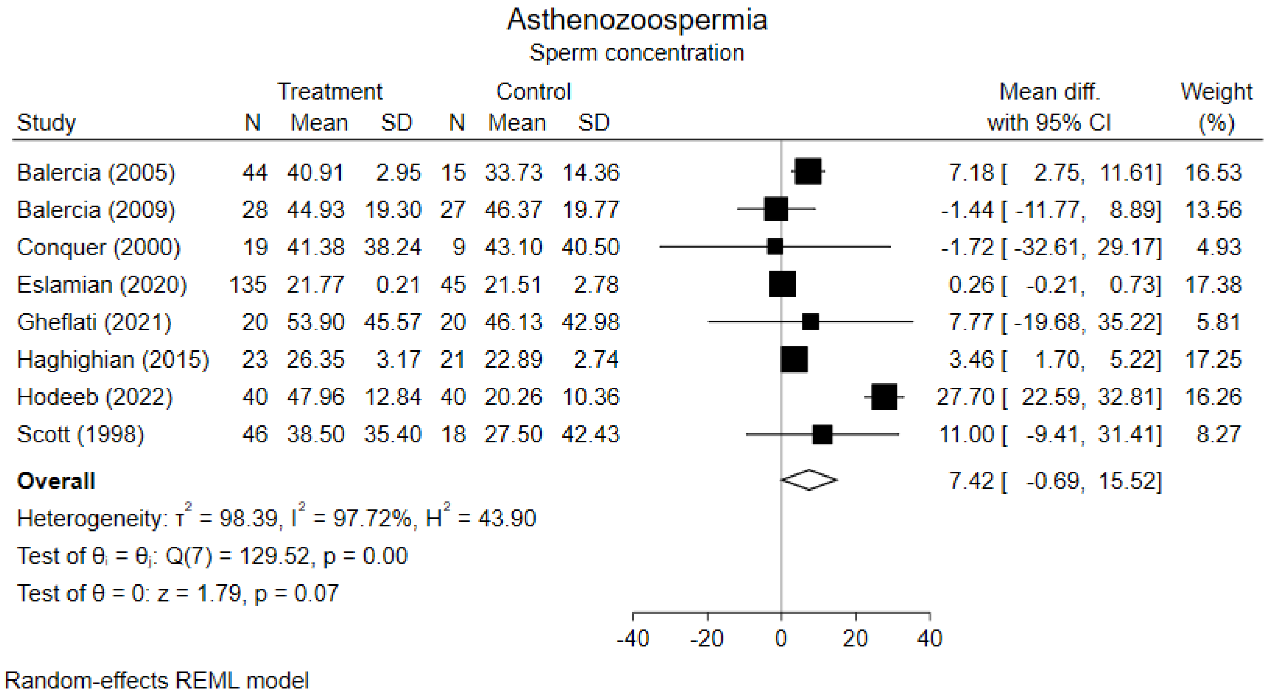

Figure S143. Forest plot on explorative subgroup analysis on the effect of dietary supplements on sperm concentration in men with asthenozoospermia.

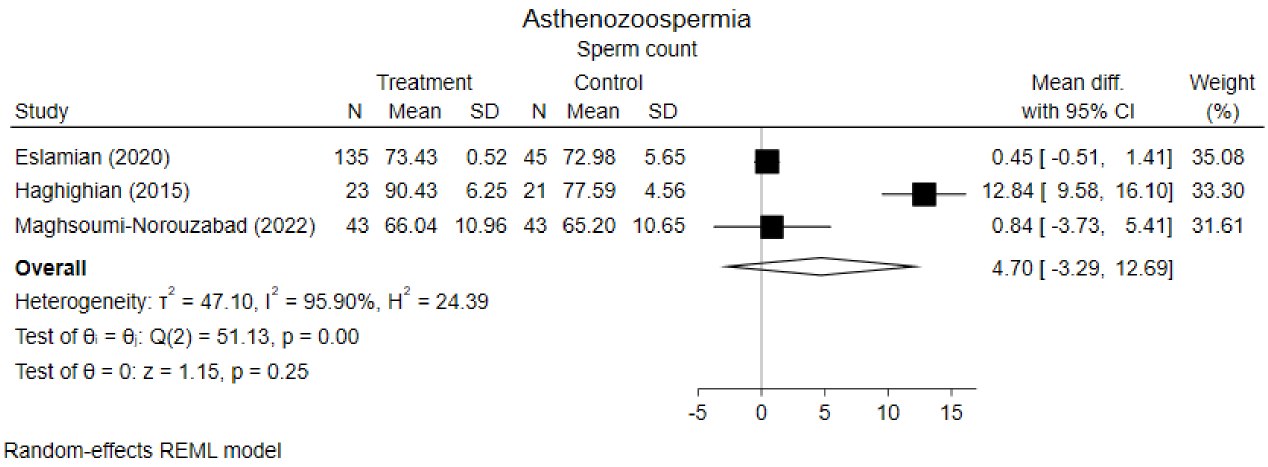

Figure S144. Forest plot on explorative subgroup analysis on the effect of dietary supplements on sperm count in men with asthenozoospermia.

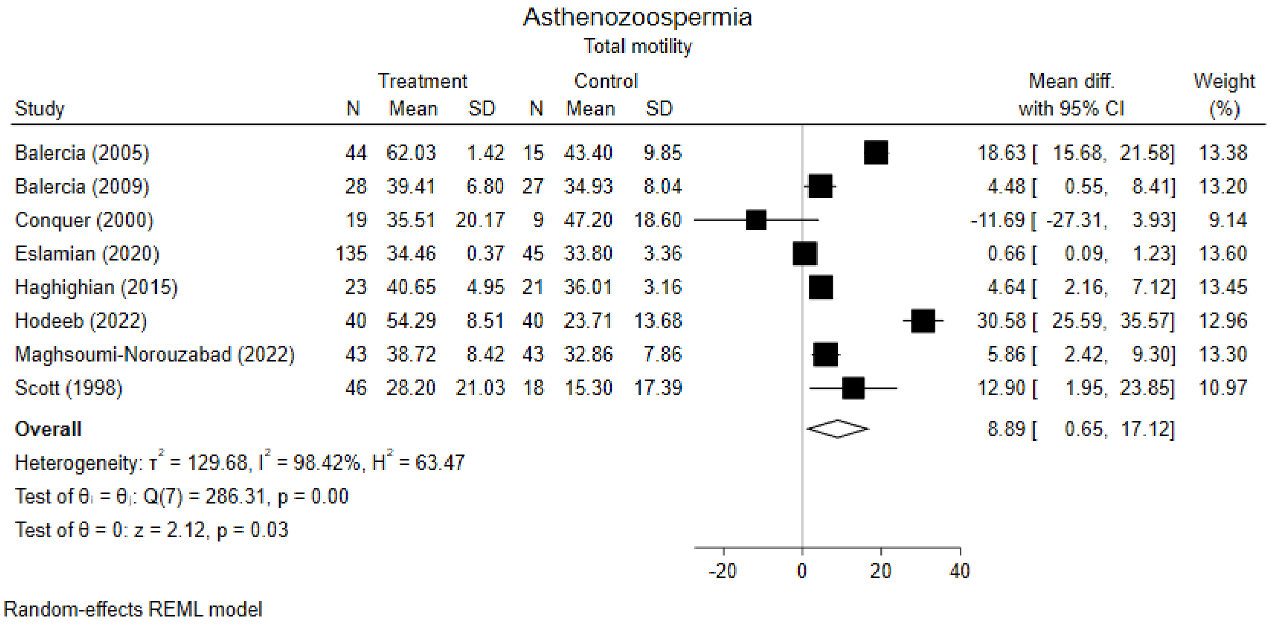

Figure S145. Forest plot on explorative subgroup analysis on the effect of dietary supplements on total motility in men with asthenozoospermia.

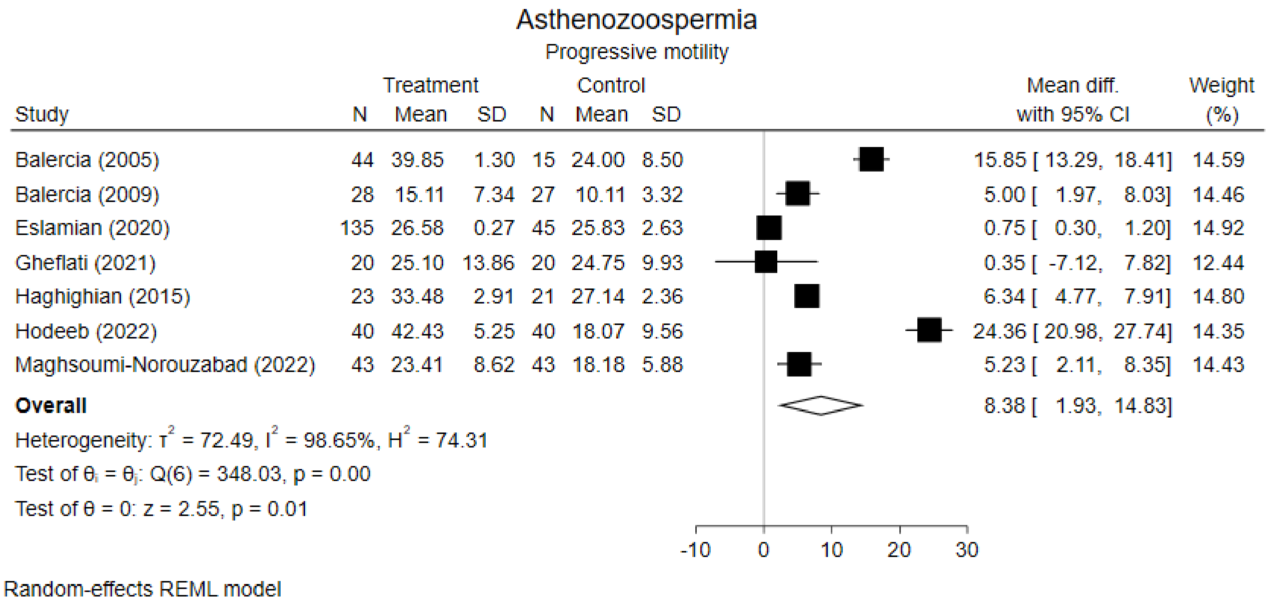

Figure S146. Forest plot on explorative subgroup analysis on the effect of dietary supplements on progressive motility in men with asthenozoospermia.

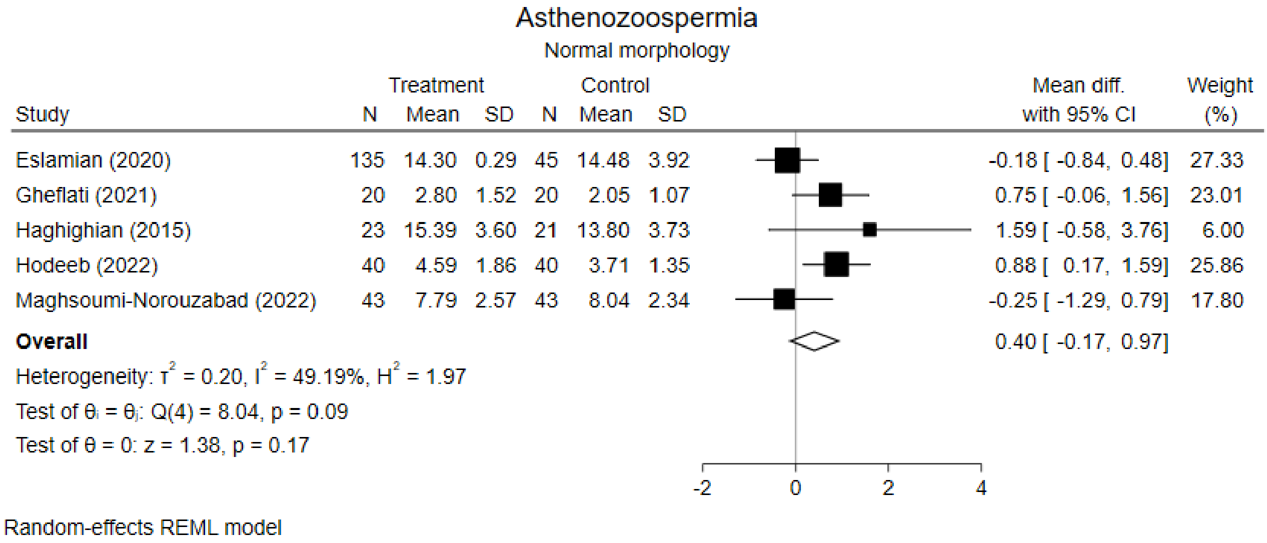

Figure S147. Forest plot on explorative subgroup analysis on the effect of dietary supplements on normal morphology in in men with asthenozoospermia.

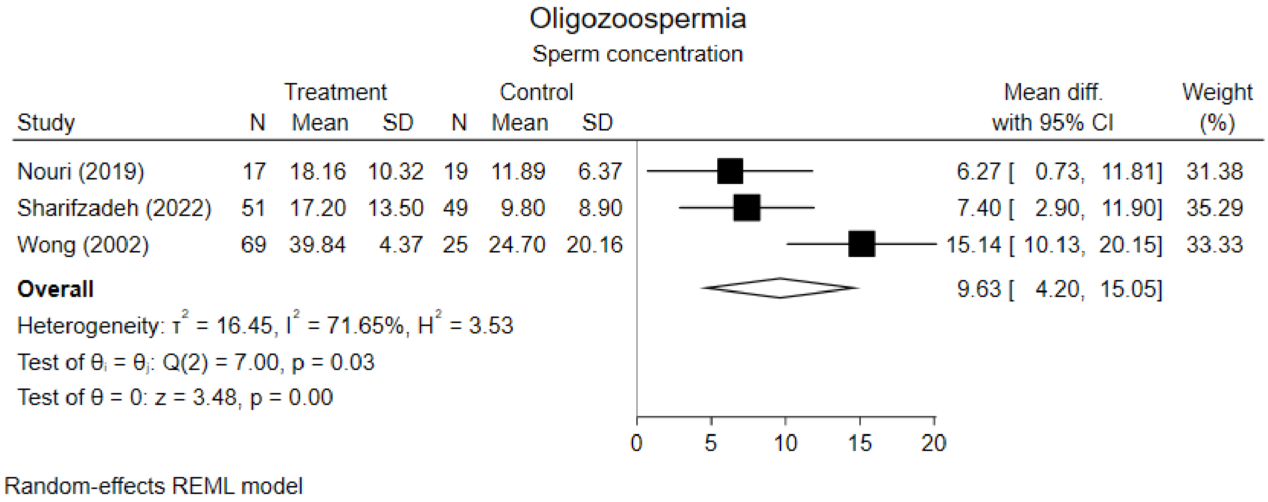

Figure S148. Forest plot on explorative subgroup analysis on the effect of dietary supplements on sperm concentration in men with oligozoospermia

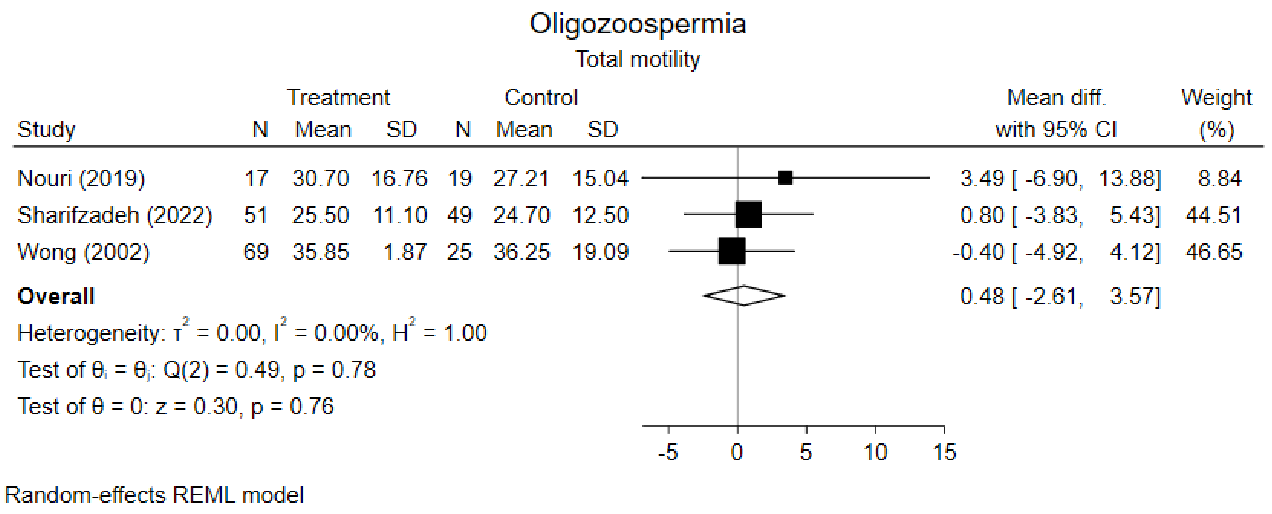

Figure S149. Forest plot on explorative subgroup analysis on the effect of dietary supplements on total motility in men with oligozoospermia

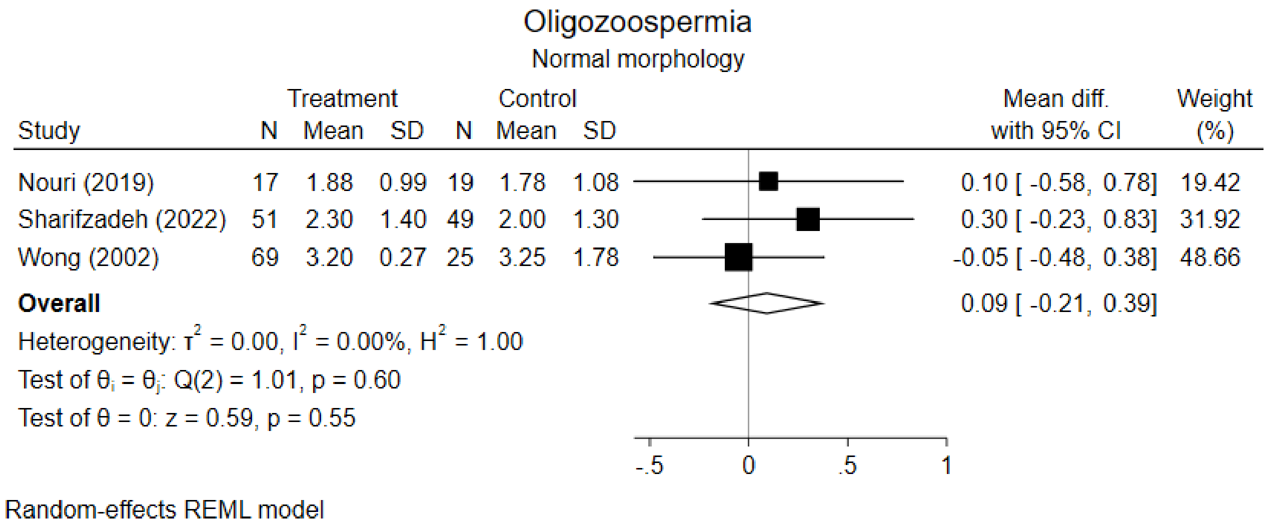

Figure S150. Forest plot on explorative subgroup analysis on the effect of dietary supplements on normal morphology in men with oligozoospermia

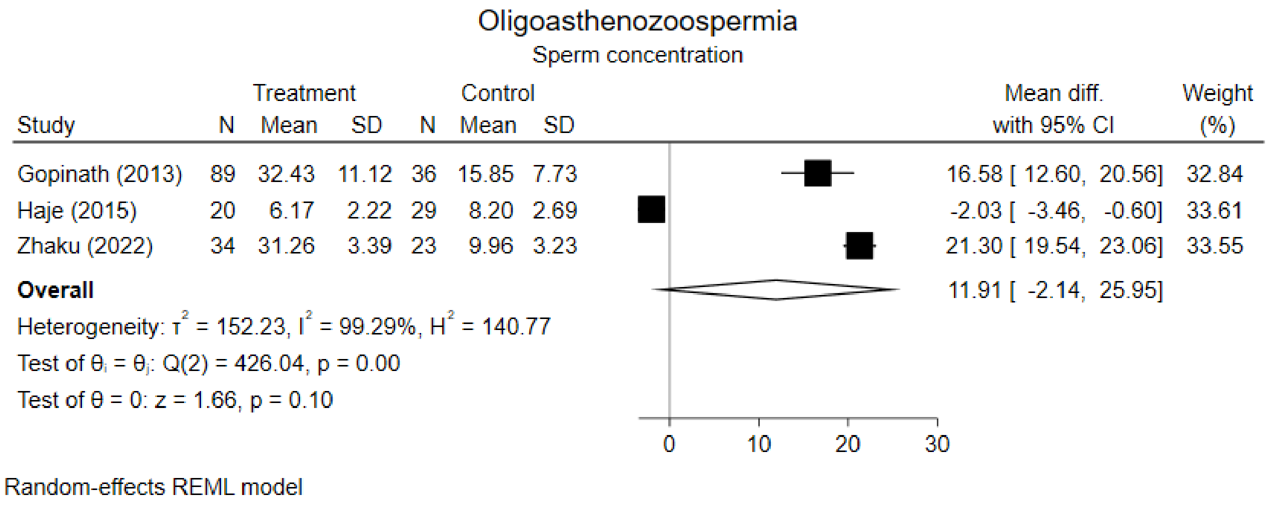

Figure S151. Forest plot on explorative subgroup analysis on the effect of dietary supplements on sperm concentration in men with oligoasthenozoospermia.

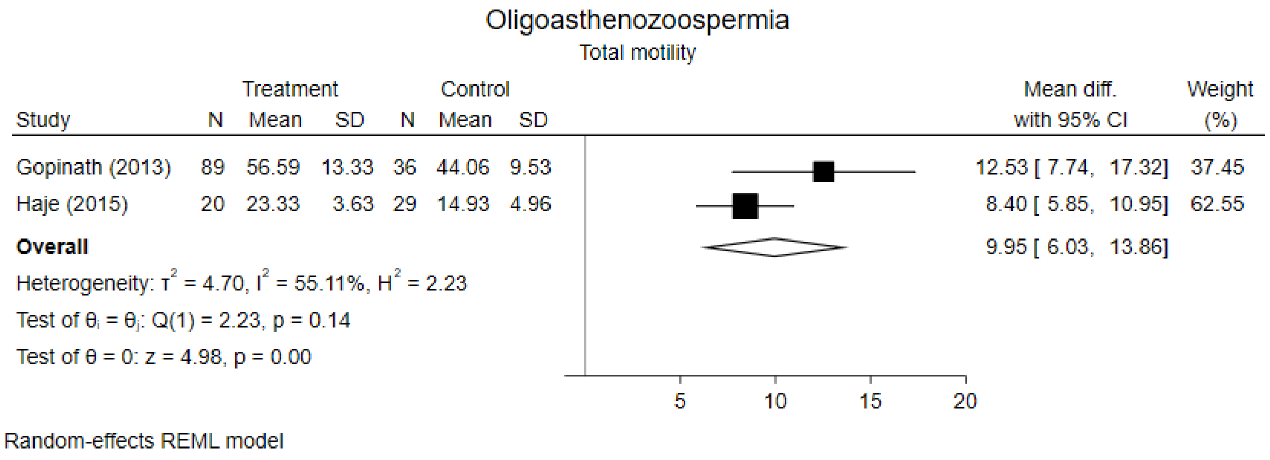

Figure S152. Forest plot on explorative subgroup analysis on the effect of dietary supplements on total motility in men with oligoasthenozoospermia.

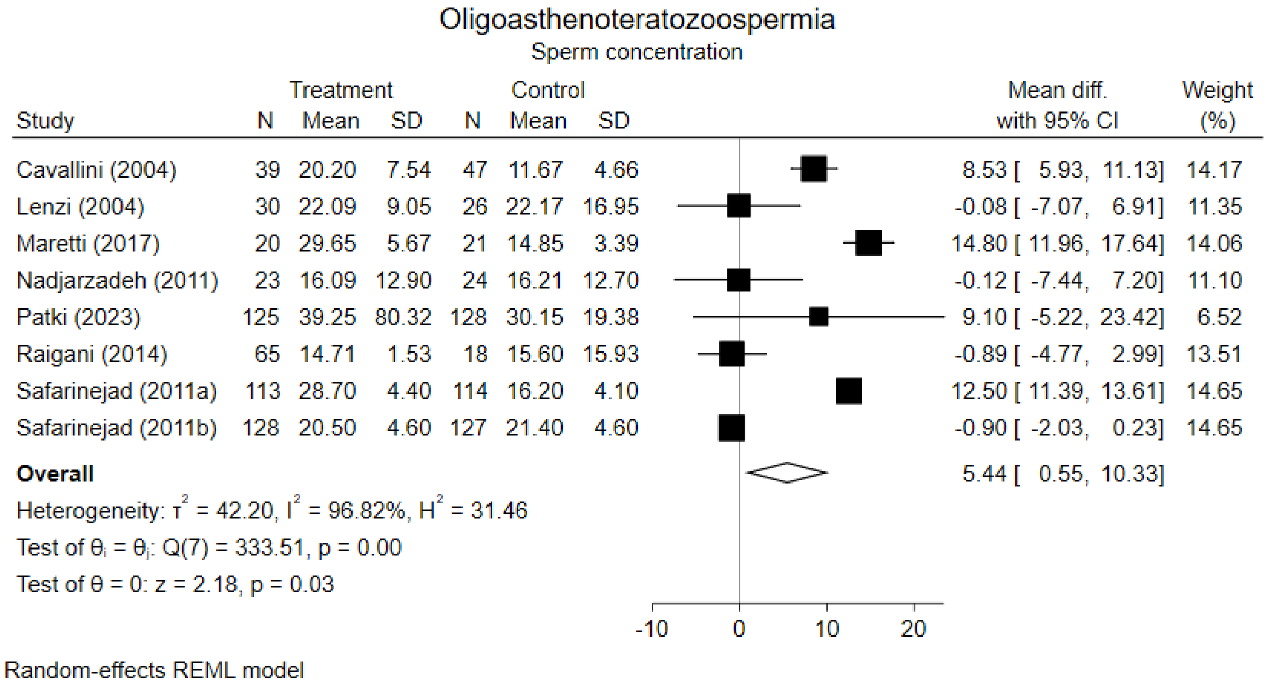

Figure S153. Forest plot on explorative subgroup analysis on the effect of dietary supplements on sperm concentration in men with oligoasthenoteratozoospermia.

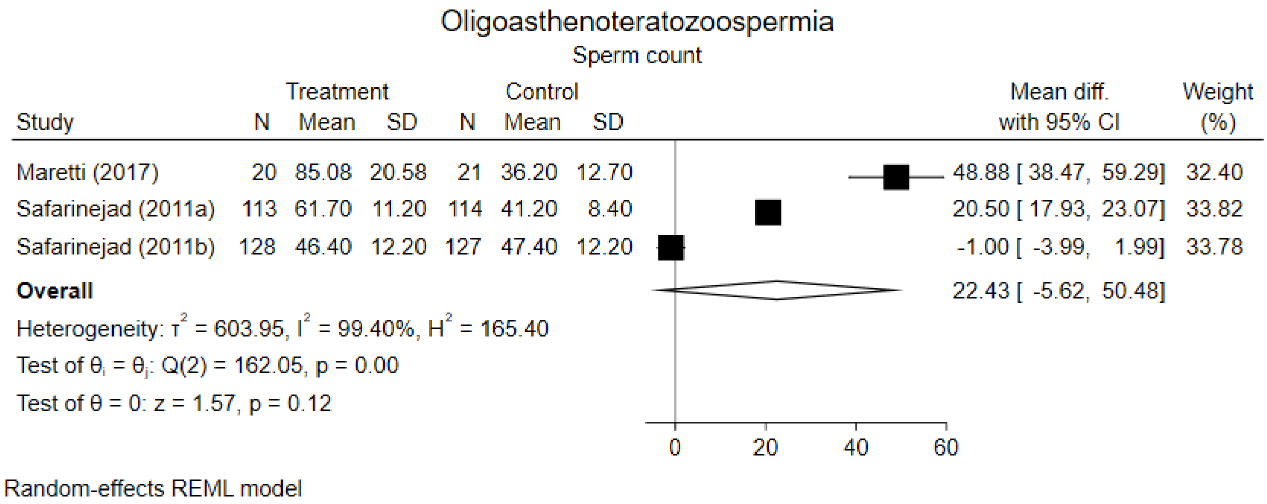

Figure S154. Forest plot on explorative subgroup analysis on the effect of dietary supplements on sperm count in men with oligoasthenoteratozoospermia.

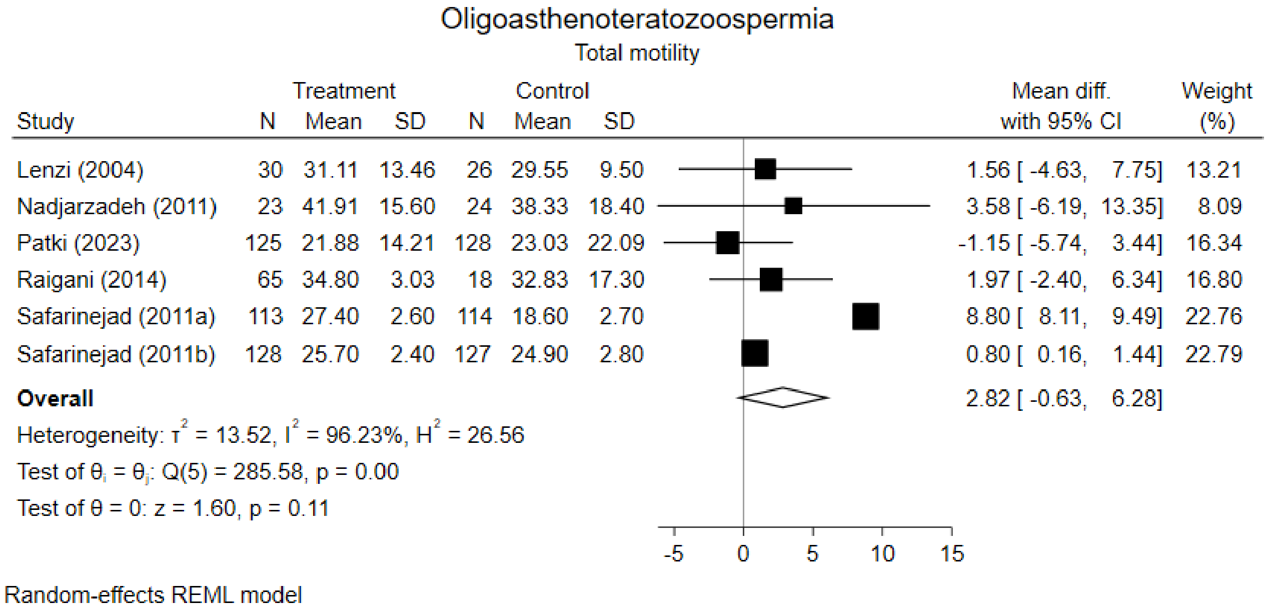

Figure S155. Forest plot on explorative subgroup analysis on the effect of dietary supplements on total motility in men with oligoasthenoteratozoospermia.

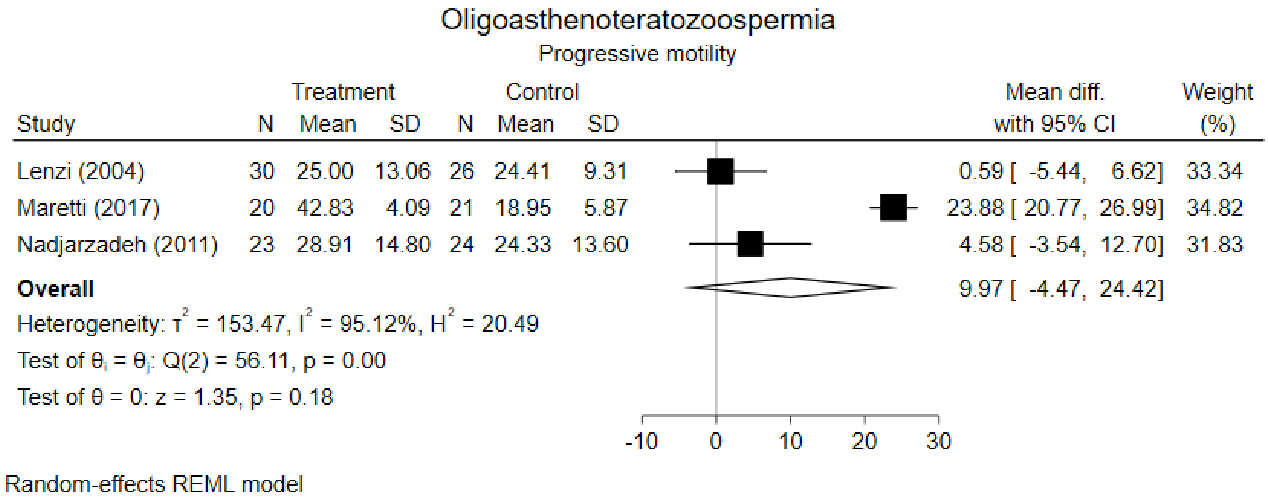

Figure S156. Forest plot on explorative subgroup analysis on the effect of dietary supplements on progressive motility in men with oligoasthenoteratozoospermia.

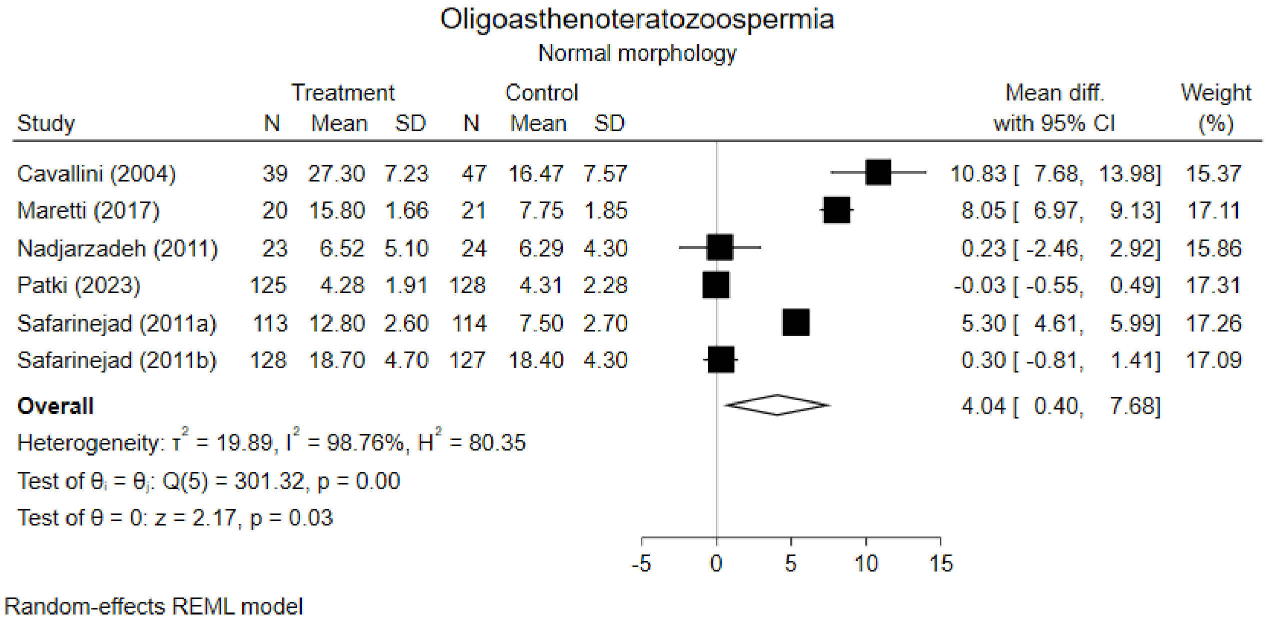

Figure S157. Forest plot on explorative subgroup analysis on the effect of dietary supplements on normal morphology in men with oligoasthenoteratozoospermia.

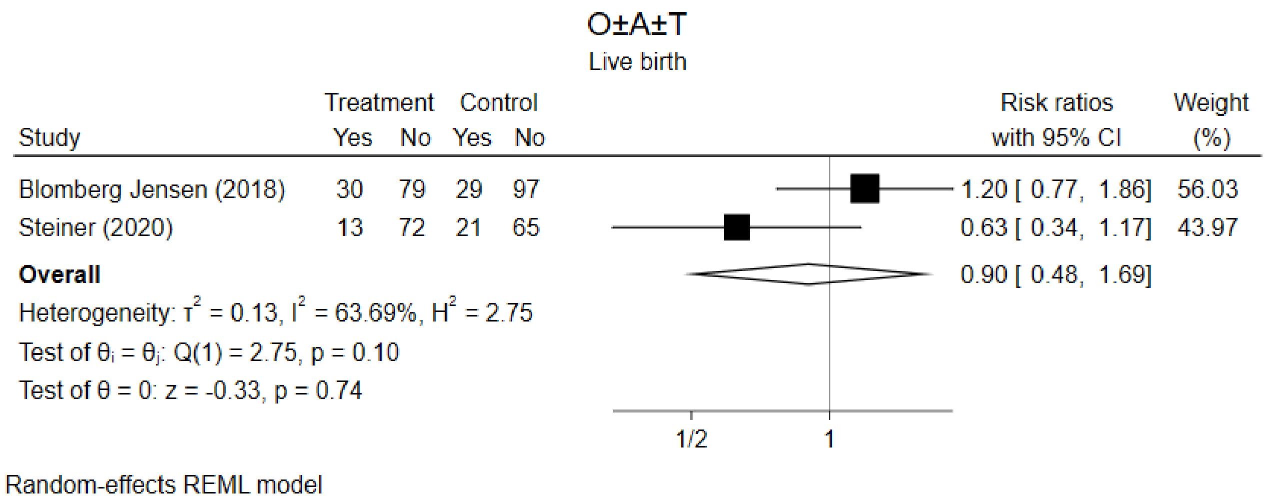

Figure S158. Forest plot on explorative subgroup analysis on the effect of dietary supplements on live birth in men with oligo- and/or astheno- and/or teratozoospermia.

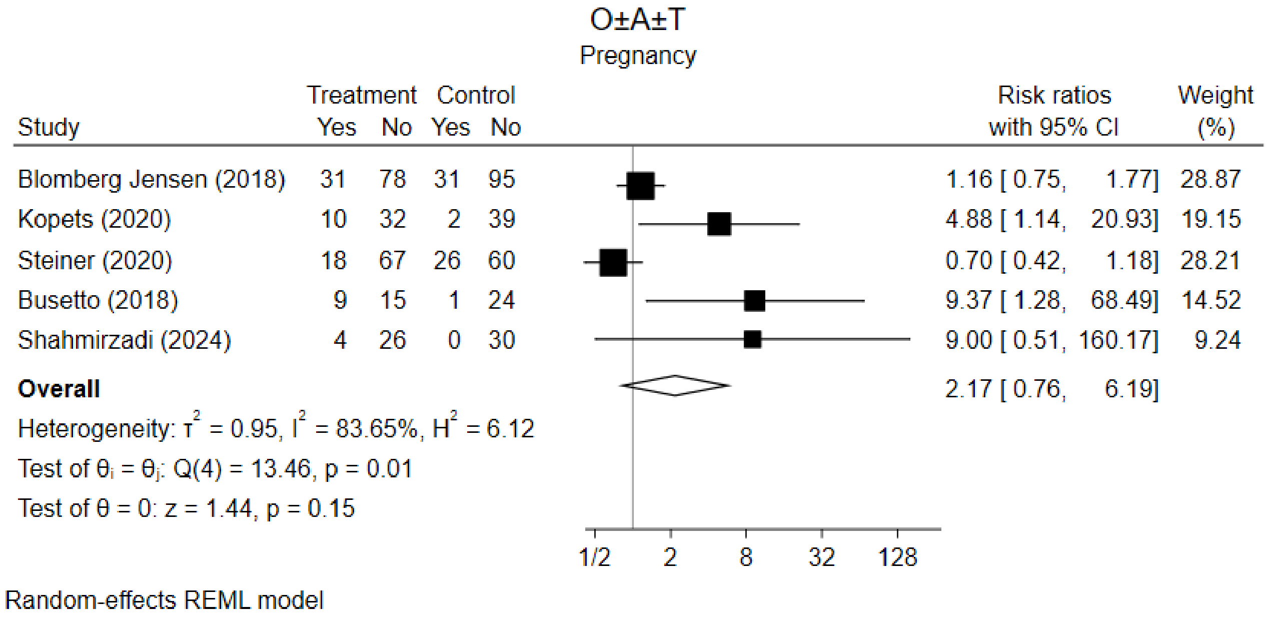

Figure S159. Forest plot on explorative subgroup analysis on the effect of dietary supplements on pregnancy in men with oligo- and/or astheno- and/or teratozoospermia.

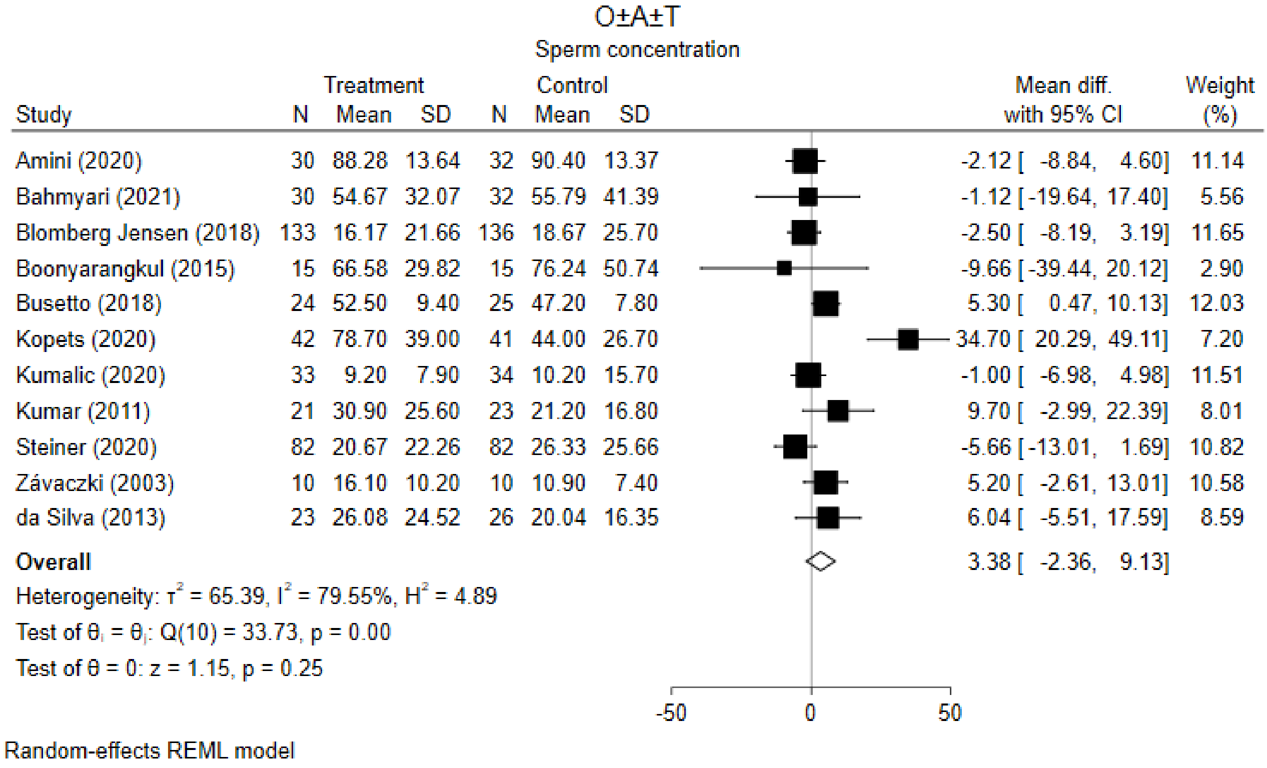

Figure S160. Forest plot on explorative subgroup analysis on the effect of dietary supplements on sperm concentration in men with oligo- and/or astheno- and/or teratozoospermia.

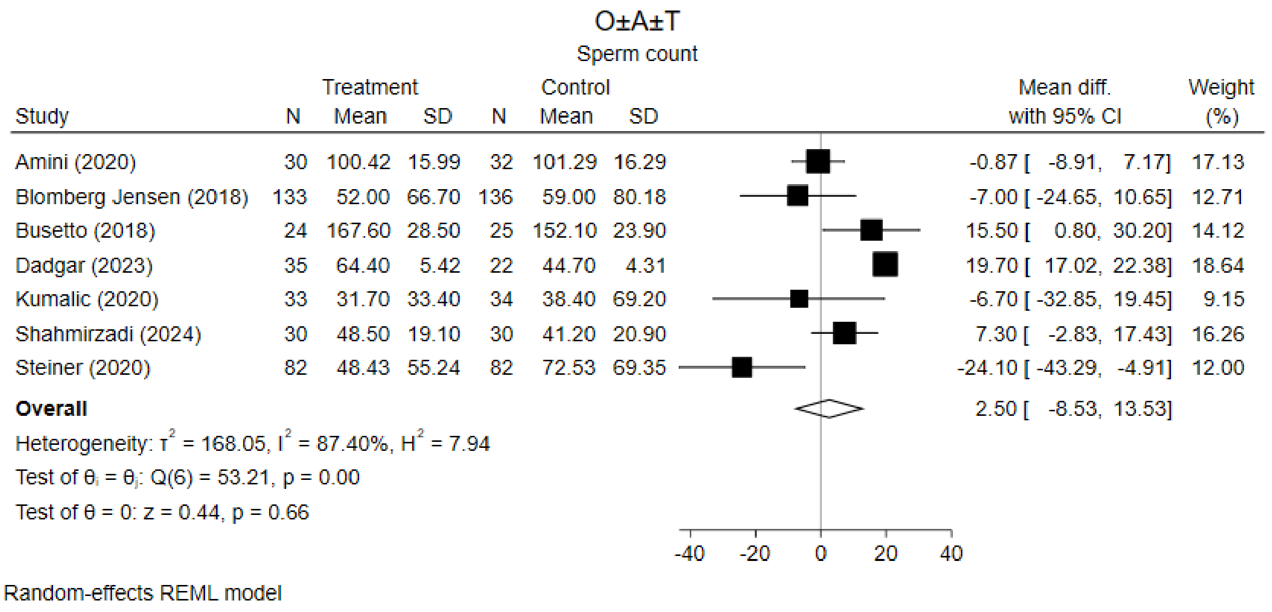

Figure S161. Forest plot on explorative subgroup analysis on the effect of dietary supplements on sperm count in men with oligo- and/or astheno- and/or teratozoospermia.

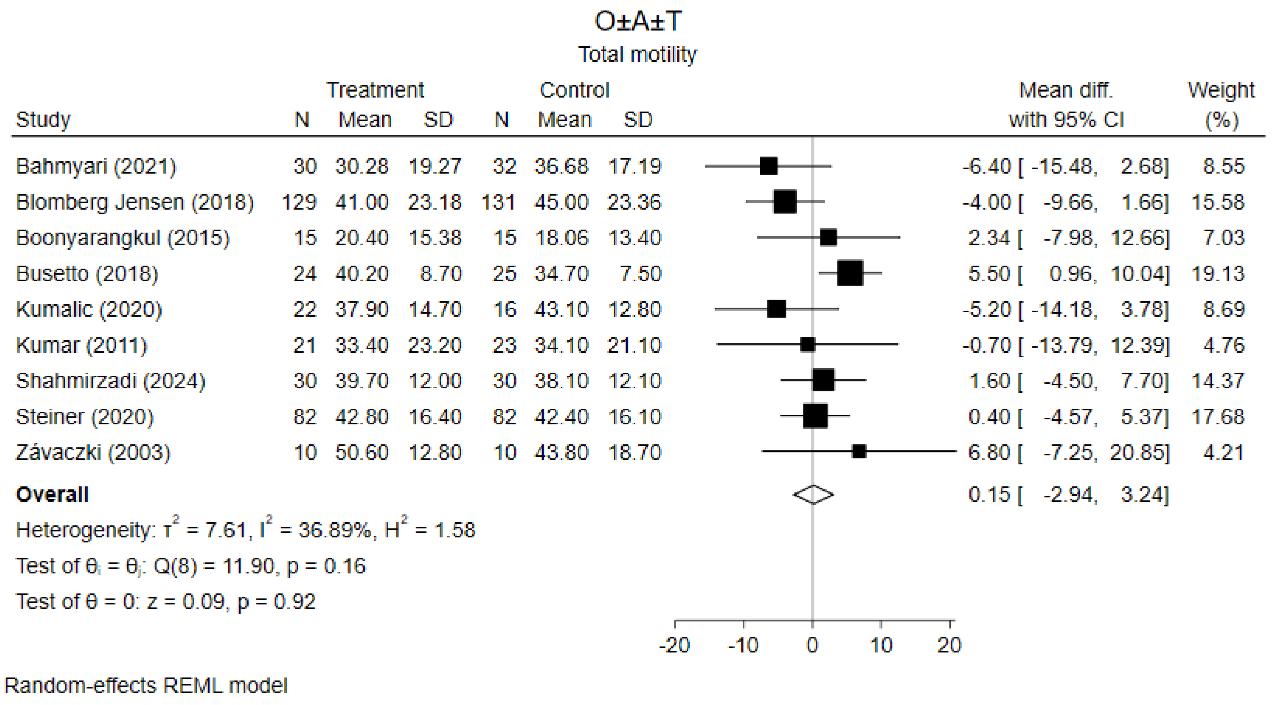

Figure S162. Forest plot on explorative subgroup analysis on the effect of dietary supplements on total motility in men with oligo- and/or astheno- and/or teratozoospermia.

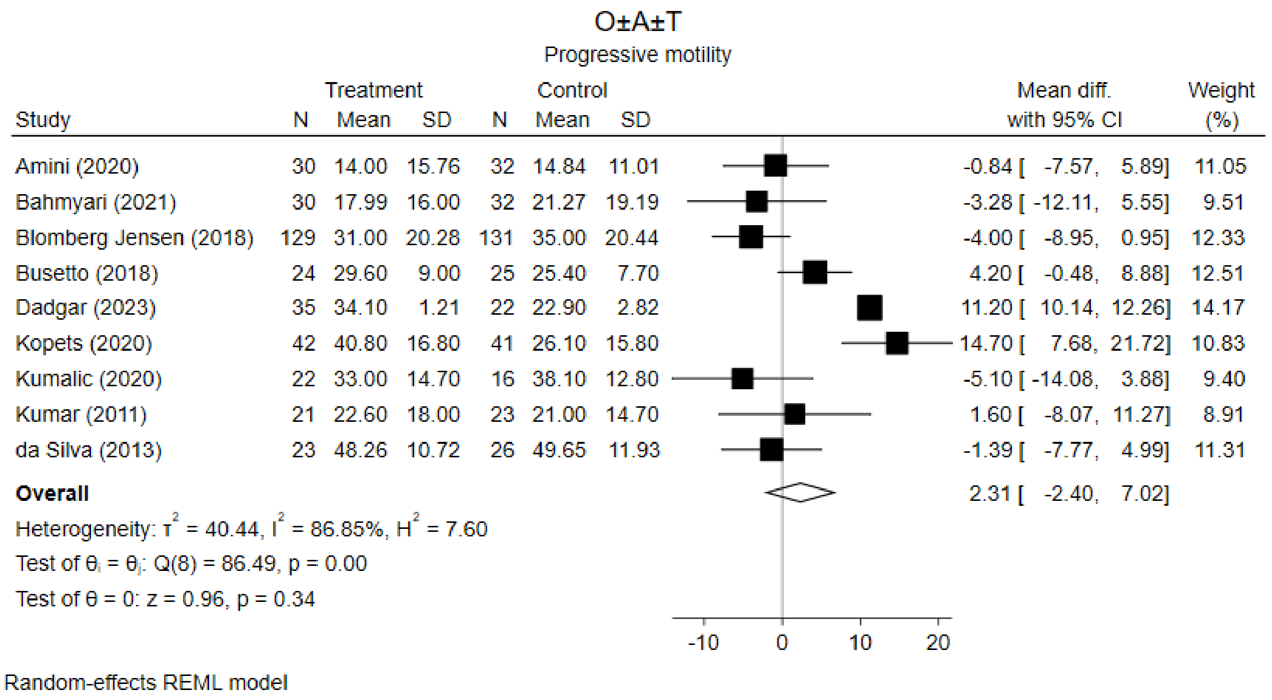

Figure S163. Forest plot on explorative subgroup analysis on the effect of dietary supplements on progressive motility in men with oligo- and/or astheno- and/or teratozoospermia.

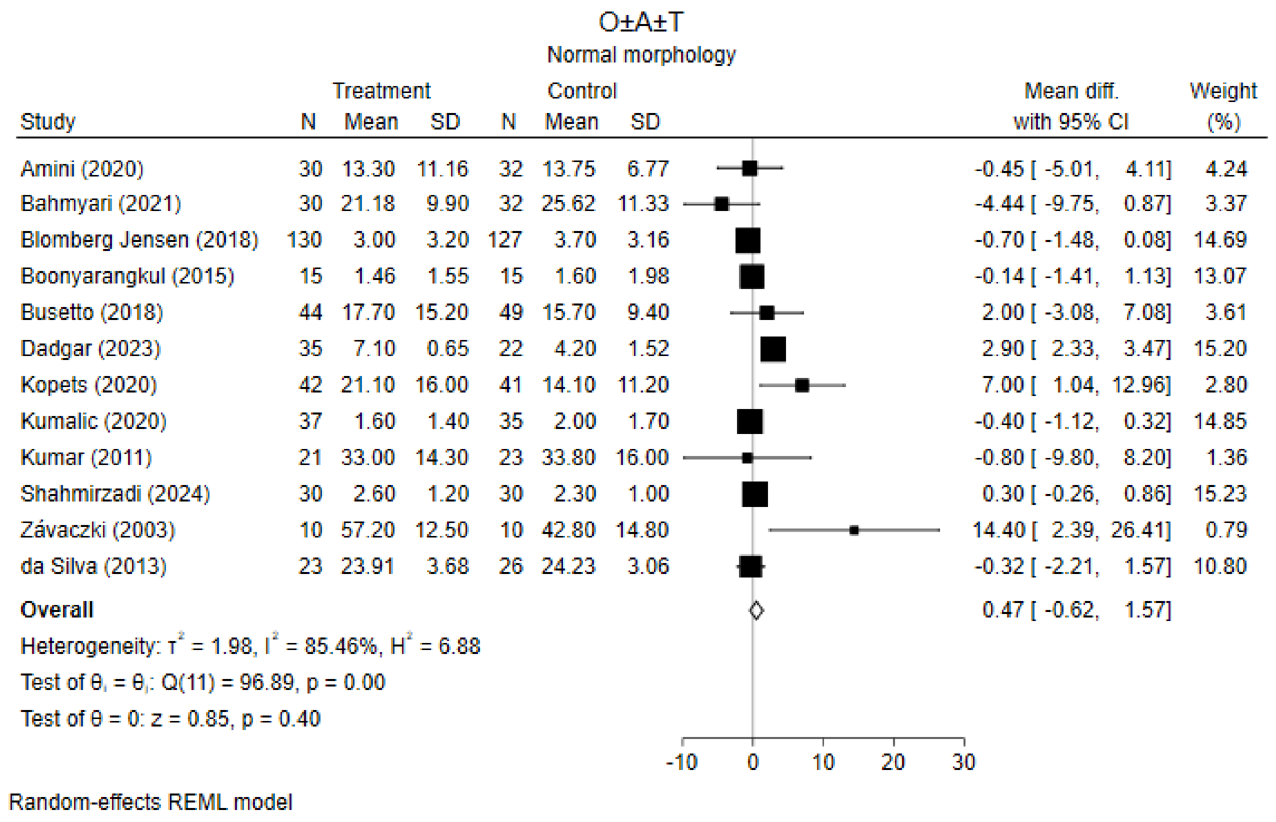

Figure S164. Forest plot on explorative subgroup analysis on the effect of dietary supplements on normal morphology in men with oligo- and/or astheno- and/or teratozoospermia.

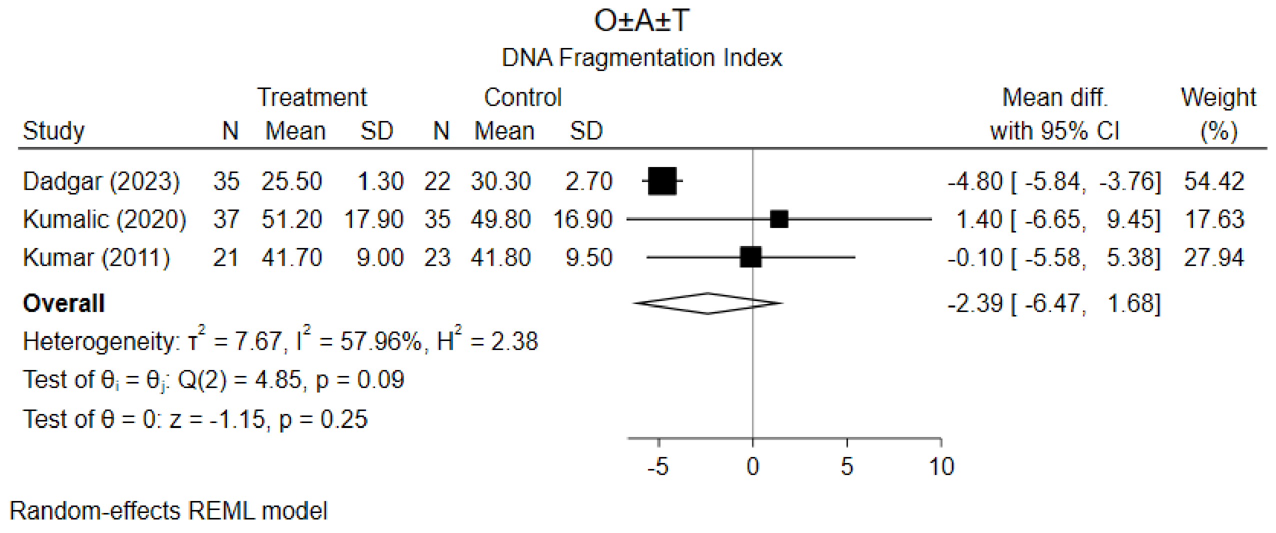

Figure S165. Forest plot on explorative subgroup analysis on the effect of dietary supplements on DNA Fragmentation Index in men with oligo- and/or astheno- and/or teratozoospermia.

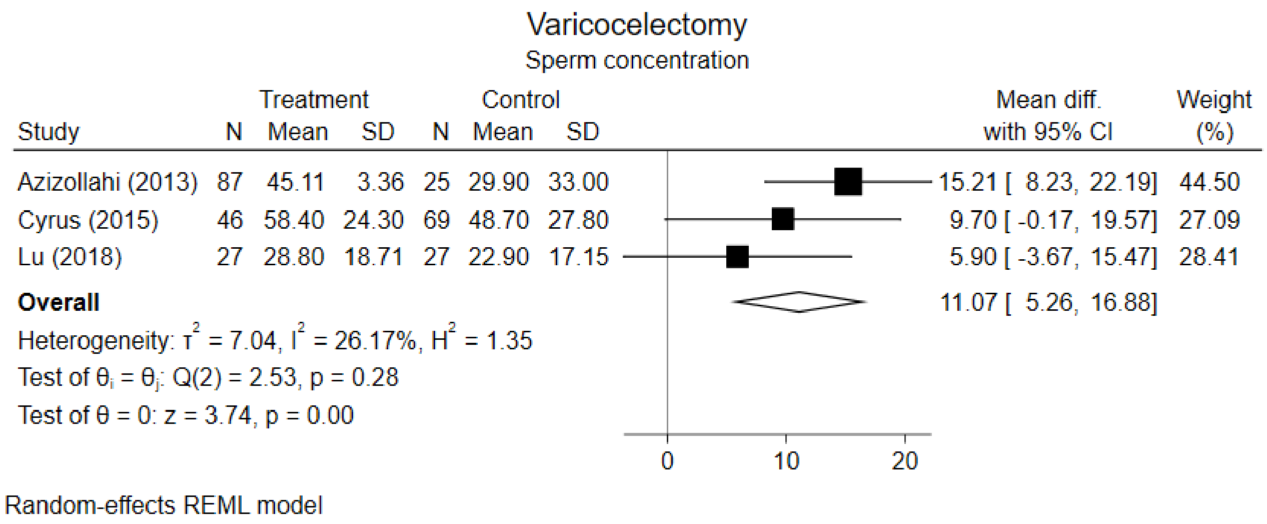

Figure S166. Forest plot on explorative subgroup analysis on the effect of dietary supplements on sperm concentration in men with varicocele undergoing varicocelectomy.

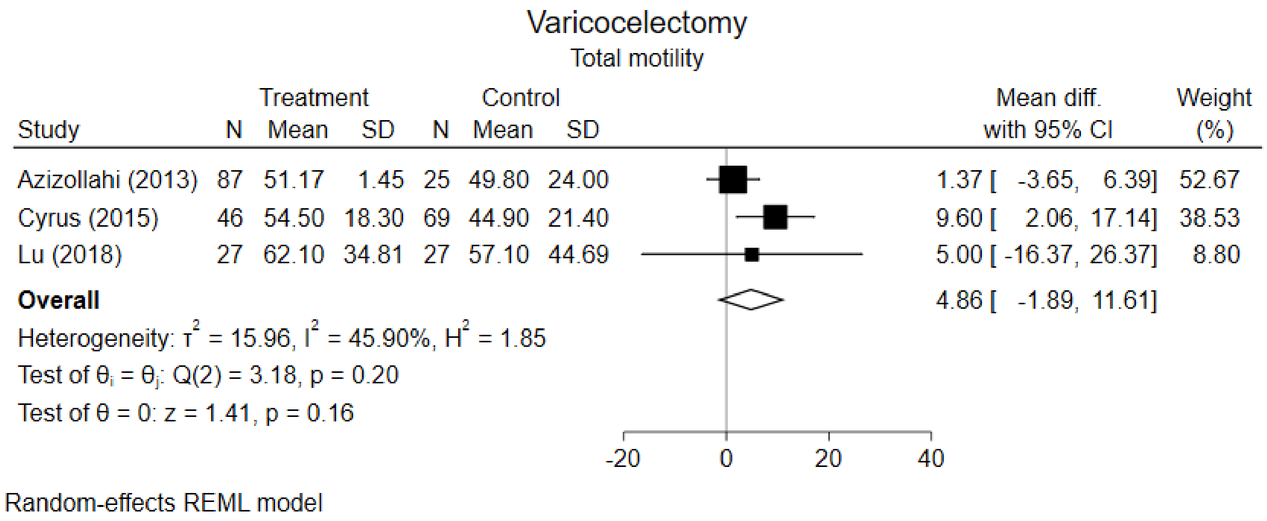

Figure S167. Forest plot on explorative subgroup analysis on the effect of dietary supplements on total motility in men with varicocele undergoing varicocelectomy.

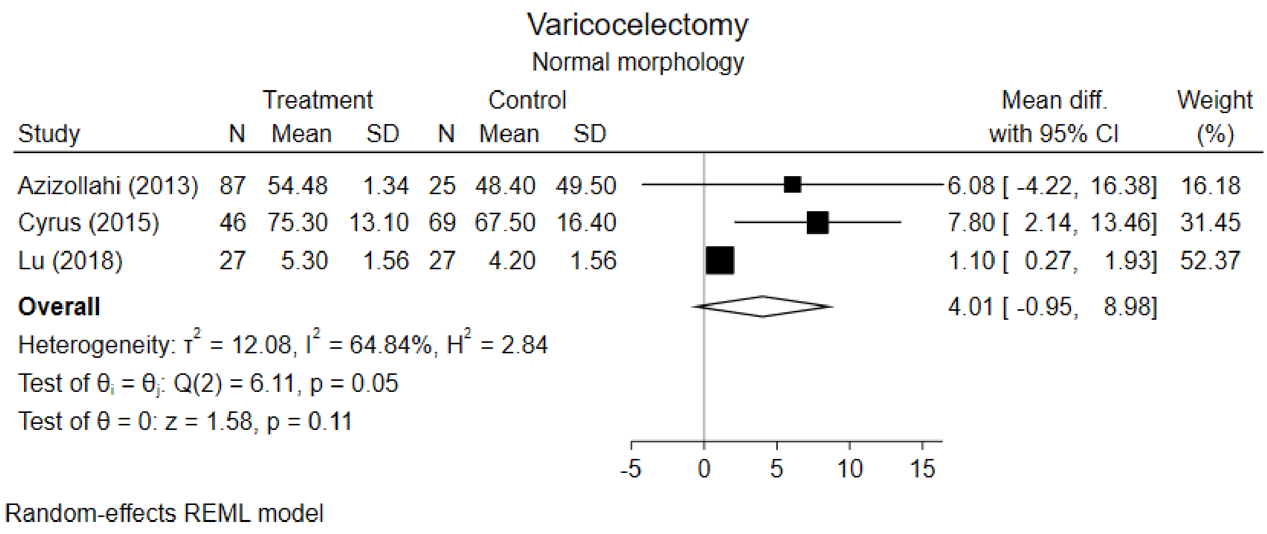

Figure S168. Forest plot on explorative subgroup analysis on the effect of dietary supplements on normal morphology in men with varicocele undergoing varicocelectomy.

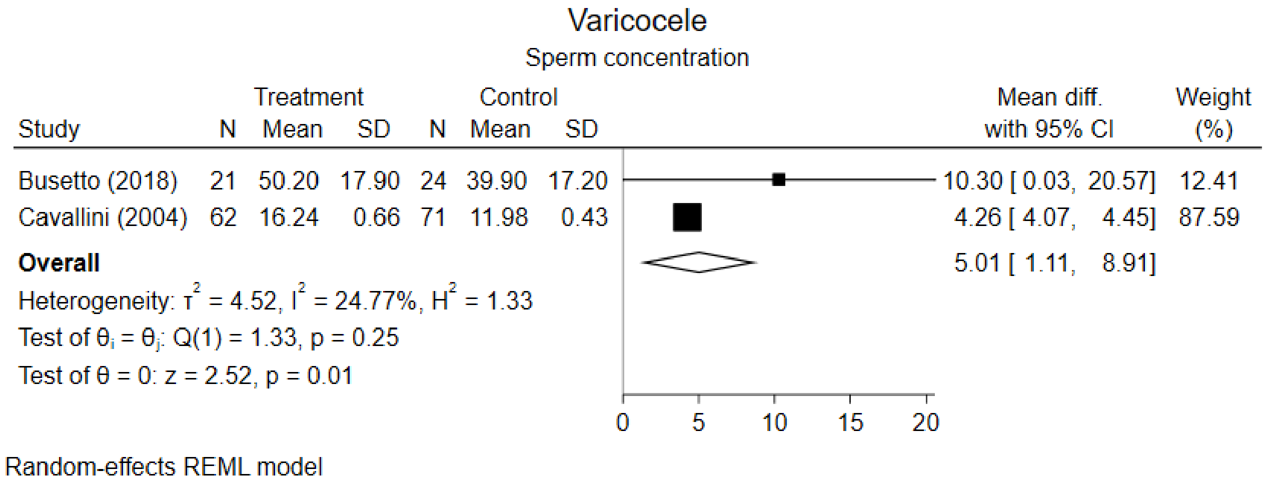

Figure S169. Forest plot on explorative subgroup analysis on the effect of dietary supplements on sperm concentration in men with varicocele.

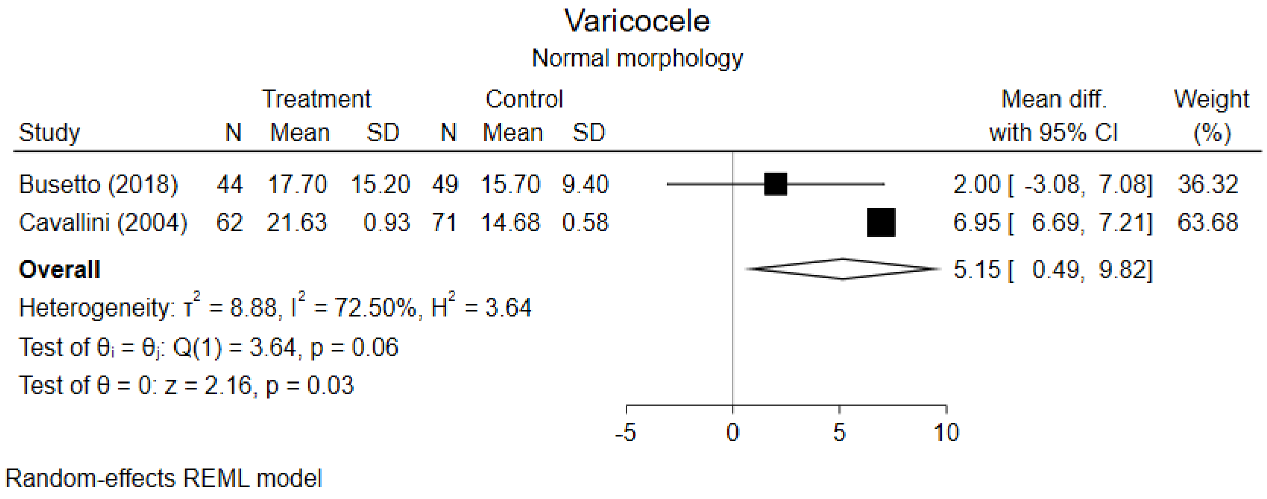

Figure S170. Forest plot on explorative subgroup analysis on the effect of dietary supplements on normal morphology in men with varicocele.

Vitamin D compared to placebo for male infertility

Patient or population: male infertility
Setting:
Intervention: Vitamin D
Comparison: placebo

Outcomes	№ of participants (studies) Follow-up	Certainty of the evidence (GRADE)	Relative effect (95% CI)	Anticipated absolute effects	
				Risk with placebo	Risk difference with Vitamin D
Sperm concentration	371 (3 RCTs)	⨁⨁◯◯ Low[a,b]	-		MD **2.09 mil/mL lower** (6.38 lower to 2.19 higher)
Sperm count	417 (3 RCTs)	⨁⨁◯◯ Low[a,b]	-		MD **0.06 mil/ejaculate higher** (3.81 lower to 3.94 higher)
Total motility	346 (2 RCTs)	⨁◯◯◯ Very low[a,c,d]	-		MD **1.2 % higher** (8.45 lower to 10.84 higher)
Progressive motility	448 (4 RCTs)	⨁◯◯◯ Very low[a,d,e]	-		MD **0.55 % higher** (4 lower to 5.1 higher)
Normal morphology	445 (4 RCTs)	⨁⨁◯◯ Low[a,b]	-		MD **0.07 % lower** (0.92 lower to 0.77 higher)

***The risk in the intervention group** (and its 95% confidence interval) is based on the assumed risk in the comparison group and the **relative effect** of the intervention (and its 95% CI).

CI: confidence interval; **MD:** mean difference

GRADE Working Group grades of evidence
High certainty: we are very confident that the true effect lies close to that of the estimate of the effect.
Moderate certainty: we are moderately confident in the effect estimate: the true effect is likely to be close to the estimate of the effect, but there is a possibility that it is substantially different.
Low certainty: our confidence in the effect estimate is limited: the true effect may be substantially different from the estimate of the effect.
Very low certainty: we have very little confidence in the effect estimate: the true effect is likely to be substantially different from the estimate of effect.

Explanations

a. Downgraded as one of study had some concerns in randomisation.
b. Downgraded due to a low number of included studies and a small sample size.
c. Downgraded due to high I^2. The two studies show different results.
d. Downgraded due to a low number of included studies, a small sample size and a wide confidence interval.
e. Downgraded due to a high I^2. The studies show different results.

Figure S171. GRADE assessment for outcomes with vitamin D as exposure.

L-carnitine and L-acetyl-carnitine compared to Placebo for Male infertility

Patient or population: Male infertility
Setting:
Intervention: L-carnitine and L-acetyl-carnitine
Comparison: Placebo

Outcomes	№ of participants (studies) Follow-up	Certainty of the evidence (GRADE)	Relative effect (95% CI)	Anticipated absolute effects	
				Risk with Placebo	Risk difference with L-carnitine and L-acetyl-carnitine
Sperm concentration	383 (4 RCTs)	⊕○○○ Very low[a,b,c]	-		MD **2.84 mil/mL higher** (1.79 lower to 7.47 higher)
Total motility	164 (3 RCTs)	⊕○○○ Very low[a,c,d]	-		MD **9.8 % higher** (0.21 higher to 19.39 higher)
Progressive motility	115 (2 RCTs)	⊕○○○ Very low[c,d,e]	-		MD **8.47 % higher** (6.47 lower to 23.42 higher)
Normal morphology	268 (2 RCTs)	⊕○○○ Very low[c,d,f]	-		MD **5.28 % higher** (0.88 lower to 11.44 higher)

*__The risk in the intervention group__ (and its 95% confidence interval) is based on the assumed risk in the comparison group and the **relative effect** of the intervention (and its 95% CI).

CI: confidence interval; **MD:** mean difference

GRADE Working Group grades of evidence
High certainty: we are very confident that the true effect lies close to that of the estimate of the effect.
Moderate certainty: we are moderately confident in the effect estimate: the true effect is likely to be close to the estimate of the effect, but there is a possibility that it is substantially different.
Low certainty: our confidence in the effect estimate is limited: the true effect may be substantially different from the estimate of the effect.
Very low certainty: we have very little confidence in the effect estimate: the true effect is likely to be substantially different from the estimate of effect.

Explanations

a. Downgraded as three studies had either some concerns or a high risk of bias in randomisation.
b. Downgraded due to a high I^2. Effect estimates of included studies shows different results.
c. Downgraded due to a low number of included studies and a small sample size.
d. Downgraded due to a high I^2.
e. Downgraded as both studies had some concerns in randomisation.
f. Downgraded as one study had a high risk of bias in randomisation. Thish study also had a weight of 50%.

Figure S172. GRADE assessment for outcomes with L-carnitine and L-acetyl-carnitine as exposure.

Zinc and folic acid compared to Placebo for male infertility

Patient or population: male infertility
Setting:
Intervention: Zinc and folic acid
Comparison: Placebo

Outcomes	№ of participants (studies) Follow-up	Certainty of the evidence (GRADE)	Relative effect (95% CI)	Anticipated absolute effects	
				Risk with Placebo	Risk difference with Zinc and folic acid
Sperm concentration	468 (6 RCTs)	⊕◯◯◯ Very low[a,b,c]	-		MD **7.81 mil/mL higher** (1.49 higher to 14.13 higher)
Total motility	419 (5 RCTs)	⊕⊕⊕◯ Moderate[a]	-		MD **1 % higher** (1.25 lower to 3.25 higher)
Progressive motility	218 (3 RCTs)	⊕◯◯◯ Very low[b,d,e]	-		MD **4.38 % higher** (3.61 lower to 12.37 higher)
Normal morphology	442 (6 RCTs)	⊕⊕◯◯ Low[a,b]	-		MD **0.69 % higher** (0.54 lower to 1.92 higher)

***The risk in the intervention group** (and its 95% confidence interval) is based on the assumed risk in the comparison group and the **relative effect** of the intervention (and its 95% CI).

CI: confidence interval; **MD:** mean difference

GRADE Working Group grades of evidence
High certainty: we are very confident that the true effect lies close to that of the estimate of the effect.
Moderate certainty: we are moderately confident in the effect estimate: the true effect is likely to be close to the estimate of the effect, but there is a possibility that it is substantially different.
Low certainty: our confidence in the effect estimate is limited: the true effect may be substantially different from the estimate of the effect.
Very low certainty: we have very little confidence in the effect estimate: the true effect is likely to be substantially different from the estimate of effect.

Explanations

a. Downgraded as all studies had either some concerns or high risk of bias in the overall assessment, and more of the studies had high risk of bias in randomisation.
b. Downgraded due to a high I^2.
c. Downgraded due to a small sample size.
d. Downgraded as one study had high risk of bias in randomisation. This study had a weight of 30%.
e. Downgraded to due a low number of included studies and a wide confidence interval.

Figure S173. GRADE assessment for outcomes with zinc and folic acid as exposure.

Coenzyme Q10 compared to Placebo for Male infertility

Patient or population: Male infertility
Setting:
Intervention: Coenzyme Q10
Comparison: Placebo

Outcomes	№ of participants (studies) Follow-up	Certainty of the evidence (GRADE)	Relative effect (95% CI)	Anticipated absolute effects	
				Risk with Placebo	Risk difference with Coenzyme Q10
Sperm concentration	102 (2 RCTs)	⊕⊕◯◯ Low[a,b]	-		MD **0.56 mil/mL lower** (6.53 lower to 5.41 higher)
Total motility	102 (2 RCTs)	⊕⊕◯◯ Low[a,c]	-		MD **4.35 % higher** (0.71 higher to 8 higher)
Progressive motility	102 (2 RCTs)	⊕⊕◯◯ Low[a,c]	-		MD **4.95 % higher** (2.11 higher to 7.79 higher)

***The risk in the intervention group** (and its 95% confidence interval) is based on the assumed risk in the comparison group and the **relative effect** of the intervention (and its 95% CI).

CI: confidence interval; **MD:** mean difference

GRADE Working Group grades of evidence
High certainty: we are very confident that the true effect lies close to that of the estimate of the effect.
Moderate certainty: we are moderately confident in the effect estimate: the true effect is likely to be close to the estimate of the effect, but there is a possibility that it is substantially different.
Low certainty: our confidence in the effect estimate is limited: the true effect may be substantially different from the estimate of the effect.
Very low certainty: we have very little confidence in the effect estimate: the true effect is likely to be substantially different from the estimate of effect.

Explanations

a. Downgraded as one study had some concerns in missing outcome data. Further, both studies had some concerns in selection of reported results.
b. Downgraded due to a low number of included studies, a small sample size and a wide confidence interval.
c. Downgraded due to a low number of included studies and a small sample size.

Figure S174. GRADE assessment for outcomes with coenzyme Q10 as exposure.

Alpha-lipoic acid compared to Placebo for male infertility					
**Patient or population:** male infertility<br>**Setting:**<br>**Intervention:** Alpha-lipoic acid<br>**Comparison:** Placebo					
**Outcomes**	**№ of participants (studies) Follow-up**	**Certainty of the evidence (GRADE)**	**Relative effect (95% CI)**	**Anticipated absolute effects**	
				**Risk with Placebo**	**Risk difference with Alpha-lipoic acid**
Sperm concentration	124 (2 RCTs)	⨁◯◯◯ Very low[a,b,c]	-		MD **15.46 mil/mL higher** (8.3 lower to 39.21 higher)
Total motility	124 (2 RCTs)	⨁◯◯◯ Very low[a,b,c]	-		MD **17.52 % higher** (7.9 lower to 42.94 higher)
Progressive motility	124 (2 RCTs)	⨁◯◯◯ Very low[a,b,c]	-		MD **15.29 % higher** (2.37 lower to 32.94 higher)
Normal morphology	124 (2 RCTs)	⨁⨁◯◯ Low[a,d]	-		MD **0.95 % higher** (0.27 higher to 1.63 higher)

*__The risk in the intervention group__ (and its 95% confidence interval) is based on the assumed risk in the comparison group and the **relative effect** of the intervention (and its 95% CI).

CI: confidence interval; **MD:** mean difference

GRADE Working Group grades of evidence
High certainty: we are very confident that the true effect lies close to that of the estimate of the effect.
Moderate certainty: we are moderately confident in the effect estimate: the true effect is likely to be close to the estimate of the effect, but there is a possibility that it is substantially different.
Low certainty: our confidence in the effect estimate is limited: the true effect may be substantially different from the estimate of the effect.
Very low certainty: we have very little confidence in the effect estimate: the true effect is likely to be substantially different from the estimate of effect.

Explanations

a. One study had some concerns in randomisation due to a significant difference in occupation. Both studies had some concerns in selection of reported results as no protocol was available and only one outcome was mentioned in both registration and method section, respectively.
b. Downgraded due to a high I^2.
c. Downgraded due to a low number of included studies, small sample size and a wide confidence interval.
d. Downgraded due to a low number of included studies and a small sample size.

Figure S175. GRADE assessment for outcomes with alpha-lipoic acid as exposure.

Omega-3 compared to Placebo for Male infertility					
**Patient or population:** Male infertility<br>**Setting:**<br>**Intervention:** Omega-3<br>**Comparison:** Placebo					
**Outcomes**	**№ of participants (studies) Follow-up**	**Certainty of the evidence (GRADE)**	**Relative effect (95% CI)**	**Anticipated absolute effects**	
				**Risk with Placebo**	**Risk difference with Omega-3**
Sperm concentration	390 (3 RCTs)	⊕◯◯◯ Very low[a,b,c]	-		MD **5.73 mil/mL higher** (4.51 lower to 15.98 higher)
Sperm count	362 (2 RCTs)	⊕⊕◯◯ Low[b,c]	-		MD **10.73 mil/ejaculate higher** (8.39 lower to 29.86 higher)
Total motility	390 (3 RCTs)	⊕◯◯◯ Very low[a,b,c]	-		MD **1.57 % higher** (8.04 lower to 11.18 higher)
Normal morphology	362 (2 RCTs)	⊕⊕◯◯ Low[b,d]	-		MD **2.64 % higher** (2.64 lower to 7.91 higher)

*__The risk in the intervention group__ (and its 95% confidence interval) is based on the assumed risk in the comparison group and the **relative effect** of the intervention (and its 95% CI).

CI: confidence interval; **MD:** mean difference

GRADE Working Group grades of evidence
High certainty: we are very confident that the true effect lies close to that of the estimate of the effect.
Moderate certainty: we are moderately confident in the effect estimate: the true effect is likely to be close to the estimate of the effect, but there is a possibility that it is substantially different.
Low certainty: our confidence in the effect estimate is limited: the true effect may be substantially different from the estimate of the effect.
Very low certainty: we have very little confidence in the effect estimate: the true effect is likely to be substantially different from the estimate of effect.

Explanations

a. Downgraded as one study had high risk of bias in randomisation.
b. Downgraded due to a high I^2.
c. Downgraded due to a low number of included studies, a small sample size and a wide confidence interval.
d. Downgraded due to a low number of included studies and a small sample size.

Figure S176. GRADE assessment for outcomes with omega-3 fatty acids as exposure.

Vitamin E compared to Placebo for Male infertility					
**Patient or population:** Male infertility<br>**Setting:**<br>**Intervention:** Vitamin E<br>**Comparison:** Placebo					
**Outcomes**	**№ of participants (studies) Follow-up**	**Certainty of the evidence (GRADE)**	**Relative effect (95% CI)**	**Anticipated absolute effects**	
				**Risk with Placebo**	**Risk difference with Vitamin E**
Sperm concentration	195 (2 RCTs)	⊕⊕◯◯ Low[a,b]	-		MD **0.53 mil/mL higher** (0.36 lower to 1.42 higher)
Total motility	195 (2 RCTs)	⊕◯◯◯ Very low[a,b,c]	-		MD **8.31 % higher** (5.14 lower to 21.75 higher)
Normal morphology	195 (2 RCTs)	⊕⊕◯◯ Low[a,b]	-		MD **0.4 % higher** (0.09 lower to 0.89 higher)

***The risk in the intervention group** (and its 95% confidence interval) is based on the assumed risk in the comparison group and the **relative effect** of the intervention (and its 95% CI).

CI: confidence interval; **MD:** mean difference

GRADE Working Group grades of evidence
High certainty: we are very confident that the true effect lies close to that of the estimate of the effect.
Moderate certainty: we are moderately confident in the effect estimate: the true effect is likely to be close to the estimate of the effect, but there is a possibility that it is substantially different.
Low certainty: our confidence in the effect estimate is limited: the true effect may be substantially different from the estimate of the effect.
Very low certainty: we have very little confidence in the effect estimate: the true effect is likely to be substantially different from the estimate of effect.

Explanations

a. Downgraded as one study had some concerns in missing outcome data. Further, both studies had some concerns in selection of reported results.
b. Downgraded due to a low number of included studies and a small sample size.
c. Downgraded due to a high I^2.

Figure S177. GRADE assessment for outcomes with vitamin E as exposure.

Selenium compared to Placebo for Male infertility					
**Patient or population:** Male infertility<br>**Setting:**<br>**Intervention:** Selenium<br>**Comparison:** Placebo					
**Outcomes**	**№ of participants (studies) Follow-up**	**Certainty of the evidence (GRADE)**	**Relative effect (95% CI)**	**Anticipated absolute effects**	
				**Risk with Placebo**	**Risk difference with Selenium**
Sperm concentration	124 (2 RCTs)	⨁⨁◯◯ Low[a,b]	-		MD **6.32 mil/mL higher** (14.88 lower to 27.51 higher)
Total motility	124 (2 RCTs)	⨁⨁◯◯ Low[a,b]	-		MD **15.25 % higher** (11.71 higher to 18.8 higher)

***The risk in the intervention group** (and its 95% confidence interval) is based on the assumed risk in the comparison group and the **relative effect** of the intervention (and its 95% CI).

CI: confidence interval; **MD:** mean difference

GRADE Working Group grades of evidence
High certainty: we are very confident that the true effect lies close to that of the estimate of the effect.
Moderate certainty: we are moderately confident in the effect estimate: the true effect is likely to be close to the estimate of the effect, but there is a possibility that it is substantially different.
Low certainty: our confidence in the effect estimate is limited: the true effect may be substantially different from the estimate of the effect.
Very low certainty: we have very little confidence in the effect estimate: the true effect is likely to be substantially different from the estimate of effect.

Explanations

a. Downgraded as one study had a high risk of bias in randomisation, while the other study had some concerns in missing outcome data.
b. Downgraded due to a low number of included studies and a small sample size.

Figure S178. GRADE assessment for outcomes with selenium as exposure.

Multiple substance dietary supplements compared to Placebo for Male infertility

Patient or population: Male infertility
Setting:
Intervention: Multiple substance dietary supplements
Comparison: Placebo

Outcomes	№ of participants (studies) Follow-up	Certainty of the evidence (GRADE)	Relative effect (95% CI)	Anticipated absolute effects	
				Risk with Placebo	Risk difference with Multiple substance dietary supplements
Pregnancy	423 (4 RCTs)	⊕◯◯◯ Very low[a,b,c]	**RR 1.75** (0.58 to 5.31)	16 per 100	**12 more per 100** (7 fewer to 68 more)
Sperm concentration	1005 (10 RCTs)	⊕◯◯◯ Very low[b,d,e]	-		MD **10.53 mil/mL higher** (3 higher to 18.07 higher)
Sperm count	333 (3 RCTs)	⊕◯◯◯ Very low[b,f,g]	-		MD **3.1 mil/ejaculate higher** (38.05 lower to 44.24 higher)
Total motility	865 (8 RCTs)	⊕⊕◯◯ Low[b,d]	-		MD **2.73 % higher** (1.75 lower to 7.21 higher)
Progressive motility	415 (6 RCTs)	⊕◯◯◯ Very low[b,c,d]	-		MD **5.55 % higher** (1.31 lower to 12.41 higher)
Normal morphology	610 (6 RCTs)	⊕⊕◯◯ Low[c,h]	-		MD **0.04 % lower** (0.54 higher to 0.46 higher)
DNA Fragmentation Index	119 (2 RCTs)	⊕⊕◯◯ Low[c,i]	-		MD **1.27 % higher** (2.79 lower to 5.33 higher)

***The risk in the intervention group** (and its 95% confidence interval) is based on the assumed risk in the comparison group and the **relative effect** of the intervention (and its 95% CI).

CI: confidence interval; **MD:** mean difference; **RR:** risk ratio

GRADE Working Group grades of evidence
High certainty: we are very confident that the true effect lies close to that of the estimate of the effect.
Moderate certainty: we are moderately confident in the effect estimate: the true effect is likely to be close to the estimate of the effect, but there is a possibility that it is substantially different.
Low certainty: our confidence in the effect estimate is limited: the true effect may be substantially different from the estimate of the effect.
Very low certainty: we have very little confidence in the effect estimate: the true effect is likely to be substantially different from the estimate of effect.

Explanations

a. Downgraded as two of the four studies had some concerns in randomisation. Further, all studies had some concerns in selection of reported results.
b. Downgraded due to a high I^2.
c. Downgraded due to a low number of studies included and a small sample size. Further, results from the studies are in different directions.
d. Downgraded as multiple studies had some concerns or high risk of bias in randomisation and other domains.
e. Funnel plot suggests publication bias.
f. Downgraded as two studies had some concerns in randomisation.
g. Downgraded due to a low number of studies included, a small sample size and wide confidence intervals.
h. Downgraded as one study had a high risk of bias in deviations from the intended intervention and some concerns in missing outocme data. This study had a weight of 92%.
i. Downgraded as both studies had either some concerns or high risk of bias in selection of reported results due to discepancies between trial registration and reported measures in the studies.

Figure S179. GRADE assessment for outcomes with multiple substance dietary supplements as exposure.
